# Measurement of the transverse momentum and $\phi ^*_{\eta }$ distributions of Drell–Yan lepton pairs in proton–proton collisions at $\sqrt{s}=8$ TeV with the ATLAS detector

**DOI:** 10.1140/epjc/s10052-016-4070-4

**Published:** 2016-05-23

**Authors:** Atlas Collaboration, G. Aad, B. Abbott, J. Abdallah, O. Abdinov, R. Aben, M. Abolins, O. S. AbouZeid, H. Abramowicz, H. Abreu, R. Abreu, Y. Abulaiti, B. S. Acharya, L. Adamczyk, D. L. Adams, J. Adelman, S. Adomeit, T. Adye, A. A. Affolder, T. Agatonovic-Jovin, J. Agricola, J. A. Aguilar-Saavedra, S. P. Ahlen, F. Ahmadov, G. Aielli, H. Akerstedt, T. P. A. Åkesson, A. V. Akimov, G. L. Alberghi, J. Albert, S. Albrand, M. J. Alconada Verzini, M. Aleksa, I. N. Aleksandrov, C. Alexa, G. Alexander, T. Alexopoulos, M. Alhroob, G. Alimonti, L. Alio, J. Alison, S. P. Alkire, B. M. M. Allbrooke, P. P. Allport, A. Aloisio, A. Alonso, F. Alonso, C. Alpigiani, A. Altheimer, B. Alvarez Gonzalez, D. Álvarez Piqueras, M. G. Alviggi, B. T. Amadio, K. Amako, Y. Amaral Coutinho, C. Amelung, D. Amidei, S. P. Amor Dos Santos, A. Amorim, S. Amoroso, N. Amram, G. Amundsen, C. Anastopoulos, L. S. Ancu, N. Andari, T. Andeen, C. F. Anders, G. Anders, J. K. Anders, K. J. Anderson, A. Andreazza, V. Andrei, S. Angelidakis, I. Angelozzi, P. Anger, A. Angerami, F. Anghinolfi, A. V. Anisenkov, N. Anjos, A. Annovi, M. Antonelli, A. Antonov, J. Antos, F. Anulli, M. Aoki, L. Aperio Bella, G. Arabidze, Y. Arai, J. P. Araque, A. T. H. Arce, F. A. Arduh, J.-F. Arguin, S. Argyropoulos, M. Arik, A. J. Armbruster, O. Arnaez, H. Arnold, M. Arratia, O. Arslan, A. Artamonov, G. Artoni, S. Artz, S. Asai, N. Asbah, A. Ashkenazi, B. Åsman, L. Asquith, K. Assamagan, R. Astalos, M. Atkinson, N. B. Atlay, K. Augsten, M. Aurousseau, G. Avolio, B. Axen, M. K. Ayoub, G. Azuelos, M. A. Baak, A. E. Baas, M. J. Baca, C. Bacci, H. Bachacou, K. Bachas, M. Backes, M. Backhaus, P. Bagiacchi, P. Bagnaia, Y. Bai, T. Bain, J. T. Baines, O. K. Baker, E. M. Baldin, P. Balek, T. Balestri, F. Balli, W. K. Balunas, E. Banas, Sw. Banerjee, A. A. E. Bannoura, L. Barak, E. L. Barberio, D. Barberis, M. Barbero, T. Barillari, M. Barisonzi, T. Barklow, N. Barlow, S. L. Barnes, B. M. Barnett, R. M. Barnett, Z. Barnovska, A. Baroncelli, G. Barone, A. J. Barr, F. Barreiro, J. Barreiro Guimarães da Costa, R. Bartoldus, A. E. Barton, P. Bartos, A. Basalaev, A. Bassalat, A. Basye, R. L. Bates, S. J. Batista, J. R. Batley, M. Battaglia, M. Bauce, F. Bauer, H. S. Bawa, J. B. Beacham, M. D. Beattie, T. Beau, P. H. Beauchemin, R. Beccherle, P. Bechtle, H. P. Beck, K. Becker, M. Becker, M. Beckingham, C. Becot, A. J. Beddall, A. Beddall, V. A. Bednyakov, C. P. Bee, L. J. Beemster, T. A. Beermann, M. Begel, J. K. Behr, C. Belanger-Champagne, W. H. Bell, G. Bella, L. Bellagamba, A. Bellerive, M. Bellomo, K. Belotskiy, O. Beltramello, O. Benary, D. Benchekroun, M. Bender, K. Bendtz, N. Benekos, Y. Benhammou, E. Benhar Noccioli, J. A. Benitez Garcia, D. P. Benjamin, J. R. Bensinger, S. Bentvelsen, L. Beresford, M. Beretta, D. Berge, E. Bergeaas Kuutmann, N. Berger, F. Berghaus, J. Beringer, C. Bernard, N. R. Bernard, C. Bernius, F. U. Bernlochner, T. Berry, P. Berta, C. Bertella, G. Bertoli, F. Bertolucci, C. Bertsche, D. Bertsche, M. I. Besana, G. J. Besjes, O. Bessidskaia Bylund, M. Bessner, N. Besson, C. Betancourt, S. Bethke, A. J. Bevan, W. Bhimji, R. M. Bianchi, L. Bianchini, M. Bianco, O. Biebel, D. Biedermann, N. V. Biesuz, M. Biglietti, J. Bilbao De Mendizabal, H. Bilokon, M. Bindi, S. Binet, A. Bingul, C. Bini, S. Biondi, D. M. Bjergaard, C. W. Black, J. E. Black, K. M. Black, D. Blackburn, R. E. Blair, J.-B. Blanchard, J. E. Blanco, T. Blazek, I. Bloch, C. Blocker, W. Blum, U. Blumenschein, S. Blunier, G. J. Bobbink, V. S. Bobrovnikov, S. S. Bocchetta, A. Bocci, C. Bock, M. Boehler, J. A. Bogaerts, D. Bogavac, A. G. Bogdanchikov, C. Bohm, V. Boisvert, T. Bold, V. Boldea, A. S. Boldyrev, M. Bomben, M. Bona, M. Boonekamp, A. Borisov, G. Borissov, S. Borroni, J. Bortfeldt, V. Bortolotto, K. Bos, D. Boscherini, M. Bosman, J. Boudreau, J. Bouffard, E. V. Bouhova-Thacker, D. Boumediene, C. Bourdarios, N. Bousson, S. K. Boutle, A. Boveia, J. Boyd, I. R. Boyko, I. Bozic, J. Bracinik, A. Brandt, G. Brandt, O. Brandt, U. Bratzler, B. Brau, J. E. Brau, H. M. Braun, W. D. Breaden Madden, K. Brendlinger, A. J. Brennan, L. Brenner, R. Brenner, S. Bressler, T. M. Bristow, D. Britton, D. Britzger, F. M. Brochu, I. Brock, R. Brock, J. Bronner, G. Brooijmans, T. Brooks, W. K. Brooks, J. Brosamer, E. Brost, P. A. Bruckman de Renstrom, D. Bruncko, R. Bruneliere, A. Bruni, G. Bruni, M. Bruschi, N. Bruscino, L. Bryngemark, T. Buanes, Q. Buat, P. Buchholz, A. G. Buckley, I. A. Budagov, F. Buehrer, L. Bugge, M. K. Bugge, O. Bulekov, D. Bullock, H. Burckhart, S. Burdin, C. D. Burgard, B. Burghgrave, S. Burke, I. Burmeister, E. Busato, D. Büscher, V. Büscher, P. Bussey, J. M. Butler, A. I. Butt, C. M. Buttar, J. M. Butterworth, P. Butti, W. Buttinger, A. Buzatu, A. R. Buzykaev, S. Cabrera Urbán, D. Caforio, V. M. Cairo, O. Cakir, N. Calace, P. Calafiura, A. Calandri, G. Calderini, P. Calfayan, L. P. Caloba, D. Calvet, S. Calvet, R. Camacho Toro, S. Camarda, P. Camarri, D. Cameron, R. Caminal Armadans, S. Campana, M. Campanelli, A. Campoverde, V. Canale, A. Canepa, M. Cano Bret, J. Cantero, R. Cantrill, T. Cao, M. D. M. Capeans Garrido, I. Caprini, M. Caprini, M. Capua, R. Caputo, R. M. Carbone, R. Cardarelli, F. Cardillo, T. Carli, G. Carlino, L. Carminati, S. Caron, E. Carquin, G. D. Carrillo-Montoya, J. R. Carter, J. Carvalho, D. Casadei, M. P. Casado, M. Casolino, D. W. Casper, E. Castaneda-Miranda, A. Castelli, V. Castillo Gimenez, N. F. Castro, P. Catastini, A. Catinaccio, J. R. Catmore, A. Cattai, J. Caudron, V. Cavaliere, D. Cavalli, M. Cavalli-Sforza, V. Cavasinni, F. Ceradini, L. Cerda Alberich, B. C. Cerio, K. Cerny, A. S. Cerqueira, A. Cerri, L. Cerrito, F. Cerutti, M. Cerv, A. Cervelli, S. A. Cetin, A. Chafaq, D. Chakraborty, I. Chalupkova, Y. L. Chan, P. Chang, J. D. Chapman, D. G. Charlton, C. C. Chau, C. A. Chavez Barajas, S. Cheatham, A. Chegwidden, S. Chekanov, S. V. Chekulaev, G. A. Chelkov, M. A. Chelstowska, C. Chen, H. Chen, K. Chen, L. Chen, S. Chen, S. Chen, X. Chen, Y. Chen, H. C. Cheng, Y. Cheng, A. Cheplakov, E. Cheremushkina, R. Cherkaoui El Moursli, V. Chernyatin, E. Cheu, L. Chevalier, V. Chiarella, G. Chiarelli, G. Chiodini, A. S. Chisholm, R. T. Chislett, A. Chitan, M. V. Chizhov, K. Choi, S. Chouridou, B. K. B. Chow, V. Christodoulou, D. Chromek-Burckhart, J. Chudoba, A. J. Chuinard, J. J. Chwastowski, L. Chytka, G. Ciapetti, A. K. Ciftci, D. Cinca, V. Cindro, I. A. Cioara, A. Ciocio, F. Cirotto, Z. H. Citron, M. Ciubancan, A. Clark, B. L. Clark, P. J. Clark, R. N. Clarke, C. Clement, Y. Coadou, M. Cobal, A. Coccaro, J. Cochran, L. Coffey, L. Colasurdo, B. Cole, S. Cole, A. P. Colijn, J. Collot, T. Colombo, G. Compostella, P. Conde Muiño, E. Coniavitis, S. H. Connell, I. A. Connelly, V. Consorti, S. Constantinescu, C. Conta, G. Conti, F. Conventi, M. Cooke, B. D. Cooper, A. M. Cooper-Sarkar, T. Cornelissen, M. Corradi, F. Corriveau, A. Corso-Radu, A. Cortes-Gonzalez, G. Cortiana, G. Costa, M. J. Costa, D. Costanzo, D. Côté, G. Cottin, G. Cowan, B. E. Cox, K. Cranmer, G. Cree, S. Crépé-Renaudin, F. Crescioli, W. A. Cribbs, M. Crispin Ortuzar, M. Cristinziani, V. Croft, G. Crosetti, T. Cuhadar Donszelmann, J. Cummings, M. Curatolo, J. Cúth, C. Cuthbert, H. Czirr, P. Czodrowski, S. D’Auria, M. D’Onofrio, M. J. Da Cunha Sargedas De Sousa, C. Da Via, W. Dabrowski, A. Dafinca, T. Dai, O. Dale, F. Dallaire, C. Dallapiccola, M. Dam, J. R. Dandoy, N. P. Dang, A. C. Daniells, M. Danninger, M. Dano Hoffmann, V. Dao, G. Darbo, S. Darmora, J. Dassoulas, A. Dattagupta, W. Davey, C. David, T. Davidek, E. Davies, M. Davies, P. Davison, Y. Davygora, E. Dawe, I. Dawson, R. K. Daya-Ishmukhametova, K. De, R. de Asmundis, A. De Benedetti, S. De Castro, S. De Cecco, N. De Groot, P. de Jong, H. De la Torre, F. De Lorenzi, D. De Pedis, A. De Salvo, U. De Sanctis, A. De Santo, J. B. De Vivie De Regie, W. J. Dearnaley, R. Debbe, C. Debenedetti, D. V. Dedovich, I. Deigaard, J. Del Peso, T. Del Prete, D. Delgove, F. Deliot, C. M. Delitzsch, M. Deliyergiyev, A. Dell’Acqua, L. Dell’Asta, M. Dell’Orso, M. Della Pietra, D. della Volpe, M. Delmastro, P. A. Delsart, C. Deluca, D. A. DeMarco, S. Demers, M. Demichev, A. Demilly, S. P. Denisov, D. Derendarz, J. E. Derkaoui, F. Derue, P. Dervan, K. Desch, C. Deterre, K. Dette, P. O. Deviveiros, A. Dewhurst, S. Dhaliwal, A. Di Ciaccio, L. Di Ciaccio, A. Di Domenico, C. Di Donato, A. Di Girolamo, B. Di Girolamo, A. Di Mattia, B. Di Micco, R. Di Nardo, A. Di Simone, R. Di Sipio, D. Di Valentino, C. Diaconu, M. Diamond, F. A. Dias, M. A. Diaz, E. B. Diehl, J. Dietrich, S. Diglio, A. Dimitrievska, J. Dingfelder, P. Dita, S. Dita, F. Dittus, F. Djama, T. Djobava, J. I. Djuvsland, M. A. B. do Vale, D. Dobos, M. Dobre, C. Doglioni, T. Dohmae, J. Dolejsi, Z. Dolezal, B. A. Dolgoshein, M. Donadelli, S. Donati, P. Dondero, J. Donini, J. Dopke, A. Doria, M. T. Dova, A. T. Doyle, E. Drechsler, M. Dris, Y. Du, E. Dubreuil, E. Duchovni, G. Duckeck, O. A. Ducu, D. Duda, A. Dudarev, L. Duflot, L. Duguid, M. Dührssen, M. Dunford, H. Duran Yildiz, M. Düren, A. Durglishvili, D. Duschinger, B. Dutta, M. Dyndal, C. Eckardt, K. M. Ecker, R. C. Edgar, W. Edson, N. C. Edwards, W. Ehrenfeld, T. Eifert, G. Eigen, K. Einsweiler, T. Ekelof, M. El Kacimi, M. Ellert, S. Elles, F. Ellinghaus, A. A. Elliot, N. Ellis, J. Elmsheuser, M. Elsing, D. Emeliyanov, Y. Enari, O. C. Endner, M. Endo, J. Erdmann, A. Ereditato, G. Ernis, J. Ernst, M. Ernst, S. Errede, E. Ertel, M. Escalier, H. Esch, C. Escobar, B. Esposito, A. I. Etienvre, E. Etzion, H. Evans, A. Ezhilov, L. Fabbri, G. Facini, R. M. Fakhrutdinov, S. Falciano, R. J. Falla, J. Faltova, Y. Fang, M. Fanti, A. Farbin, A. Farilla, T. Farooque, S. Farrell, S. M. Farrington, P. Farthouat, F. Fassi, P. Fassnacht, D. Fassouliotis, M. Faucci Giannelli, A. Favareto, L. Fayard, O. L. Fedin, W. Fedorko, S. Feigl, L. Feligioni, C. Feng, E. J. Feng, H. Feng, A. B. Fenyuk, L. Feremenga, P. Fernandez Martinez, S. Fernandez Perez, J. Ferrando, A. Ferrari, P. Ferrari, R. Ferrari, D. E. Ferreira de Lima, A. Ferrer, D. Ferrere, C. Ferretti, A. Ferretto Parodi, M. Fiascaris, F. Fiedler, A. Filipčič, M. Filipuzzi, F. Filthaut, M. Fincke-Keeler, K. D. Finelli, M. C. N. Fiolhais, L. Fiorini, A. Firan, A. Fischer, C. Fischer, J. Fischer, W. C. Fisher, N. Flaschel, I. Fleck, P. Fleischmann, G. T. Fletcher, G. Fletcher, R. R. M. Fletcher, T. Flick, A. Floderus, L. R. Flores Castillo, M. J. Flowerdew, A. Formica, A. Forti, D. Fournier, H. Fox, S. Fracchia, P. Francavilla, M. Franchini, D. Francis, L. Franconi, M. Franklin, M. Frate, M. Fraternali, D. Freeborn, S. T. French, S. M. Fressard-Batraneanu, F. Friedrich, D. Froidevaux, J. A. Frost, C. Fukunaga, E. Fullana Torregrosa, B. G. Fulsom, T. Fusayasu, J. Fuster, C. Gabaldon, O. Gabizon, A. Gabrielli, A. Gabrielli, G. P. Gach, S. Gadatsch, S. Gadomski, G. Gagliardi, P. Gagnon, C. Galea, B. Galhardo, E. J. Gallas, B. J. Gallop, P. Gallus, G. Galster, K. K. Gan, J. Gao, Y. Gao, Y. S. Gao, F. M. Garay Walls, F. Garberson, C. García, J. E. García Navarro, M. Garcia-Sciveres, R. W. Gardner, N. Garelli, V. Garonne, C. Gatti, A. Gaudiello, G. Gaudio, B. Gaur, L. Gauthier, P. Gauzzi, I. L. Gavrilenko, C. Gay, G. Gaycken, E. N. Gazis, P. Ge, Z. Gecse, C. N. P. Gee, Ch. Geich-Gimbel, M. P. Geisler, C. Gemme, M. H. Genest, C. Geng, S. Gentile, M. George, S. George, D. Gerbaudo, A. Gershon, S. Ghasemi, H. Ghazlane, B. Giacobbe, S. Giagu, V. Giangiobbe, P. Giannetti, B. Gibbard, S. M. Gibson, M. Gignac, M. Gilchriese, T. P. S. Gillam, D. Gillberg, G. Gilles, D. M. Gingrich, N. Giokaris, M. P. Giordani, F. M. Giorgi, F. M. Giorgi, P. F. Giraud, P. Giromini, D. Giugni, C. Giuliani, M. Giulini, B. K. Gjelsten, S. Gkaitatzis, I. Gkialas, E. L. Gkougkousis, L. K. Gladilin, C. Glasman, J. Glatzer, P. C. F. Glaysher, A. Glazov, M. Goblirsch-Kolb, J. R. Goddard, J. Godlewski, S. Goldfarb, T. Golling, D. Golubkov, A. Gomes, R. Gonçalo, J. Goncalves Pinto Firmino Da Costa, L. Gonella, S. González de la Hoz, G. Gonzalez Parra, S. Gonzalez-Sevilla, L. Goossens, P. A. Gorbounov, H. A. Gordon, I. Gorelov, B. Gorini, E. Gorini, A. Gorišek, E. Gornicki, A. T. Goshaw, C. Gössling, M. I. Gostkin, D. Goujdami, A. G. Goussiou, N. Govender, E. Gozani, L. Graber, I. Grabowska-Bold, P. O. J. Gradin, P. Grafström, J. Gramling, E. Gramstad, S. Grancagnolo, V. Gratchev, H. M. Gray, E. Graziani, Z. D. Greenwood, C. Grefe, K. Gregersen, I. M. Gregor, P. Grenier, J. Griffiths, A. A. Grillo, K. Grimm, S. Grinstein, Ph. Gris, J.-F. Grivaz, S. Groh, J. P. Grohs, A. Grohsjean, E. Gross, J. Grosse-Knetter, G. C. Grossi, Z. J. Grout, L. Guan, J. Guenther, F. Guescini, D. Guest, O. Gueta, E. Guido, T. Guillemin, S. Guindon, U. Gul, C. Gumpert, J. Guo, Y. Guo, S. Gupta, G. Gustavino, P. Gutierrez, N. G. Gutierrez Ortiz, C. Gutschow, C. Guyot, C. Gwenlan, C. B. Gwilliam, A. Haas, C. Haber, H. K. Hadavand, N. Haddad, P. Haefner, S. Hageböck, Z. Hajduk, H. Hakobyan, M. Haleem, J. Haley, D. Hall, G. Halladjian, G. D. Hallewell, K. Hamacher, P. Hamal, K. Hamano, A. Hamilton, G. N. Hamity, P. G. Hamnett, L. Han, K. Hanagaki, K. Hanawa, M. Hance, B. Haney, P. Hanke, R. Hanna, J. B. Hansen, J. D. Hansen, M. C. Hansen, P. H. Hansen, K. Hara, A. S. Hard, T. Harenberg, F. Hariri, S. Harkusha, R. D. Harrington, P. F. Harrison, F. Hartjes, M. Hasegawa, Y. Hasegawa, A. Hasib, S. Hassani, S. Haug, R. Hauser, L. Hauswald, M. Havranek, C. M. Hawkes, R. J. Hawkings, A. D. Hawkins, T. Hayashi, D. Hayden, C. P. Hays, J. M. Hays, H. S. Hayward, S. J. Haywood, S. J. Head, T. Heck, V. Hedberg, L. Heelan, S. Heim, T. Heim, B. Heinemann, L. Heinrich, J. Hejbal, L. Helary, S. Hellman, C. Helsens, J. Henderson, R. C. W. Henderson, Y. Heng, C. Hengler, S. Henkelmann, A. Henrichs, A. M. Henriques Correia, S. Henrot-Versille, G. H. Herbert, Y. Hernández Jiménez, G. Herten, R. Hertenberger, L. Hervas, G. G. Hesketh, N. P. Hessey, J. W. Hetherly, R. Hickling, E. Higón-Rodriguez, E. Hill, J. C. Hill, K. H. Hiller, S. J. Hillier, I. Hinchliffe, E. Hines, R. R. Hinman, M. Hirose, D. Hirschbuehl, J. Hobbs, N. Hod, M. C. Hodgkinson, P. Hodgson, A. Hoecker, M. R. Hoeferkamp, F. Hoenig, M. Hohlfeld, D. Hohn, T. R. Holmes, M. Homann, T. M. Hong, B. H. Hooberman, W. H. Hopkins, Y. Horii, A. J. Horton, J.-Y. Hostachy, S. Hou, A. Hoummada, J. Howard, J. Howarth, M. Hrabovsky, I. Hristova, J. Hrivnac, T. Hryn’ova, A. Hrynevich, C. Hsu, P. J. Hsu, S.-C. Hsu, D. Hu, Q. Hu, X. Hu, Y. Huang, Z. Hubacek, F. Hubaut, F. Huegging, T. B. Huffman, E. W. Hughes, G. Hughes, M. Huhtinen, T. A. Hülsing, N. Huseynov, J. Huston, J. Huth, G. Iacobucci, G. Iakovidis, I. Ibragimov, L. Iconomidou-Fayard, E. Ideal, Z. Idrissi, P. Iengo, O. Igonkina, T. Iizawa, Y. Ikegami, M. Ikeno, Y. Ilchenko, D. Iliadis, N. Ilic, T. Ince, G. Introzzi, P. Ioannou, M. Iodice, K. Iordanidou, V. Ippolito, A. Irles Quiles, C. Isaksson, M. Ishino, M. Ishitsuka, R. Ishmukhametov, C. Issever, S. Istin, J. M. Iturbe Ponce, R. Iuppa, J. Ivarsson, W. Iwanski, H. Iwasaki, J. M. Izen, V. Izzo, S. Jabbar, B. Jackson, M. Jackson, P. Jackson, M. R. Jaekel, V. Jain, K. B. Jakobi, K. Jakobs, S. Jakobsen, T. Jakoubek, J. Jakubek, D. O. Jamin, D. K. Jana, E. Jansen, R. Jansky, J. Janssen, M. Janus, G. Jarlskog, N. Javadov, T. Javůrek, L. Jeanty, J. Jejelava, G.-Y. Jeng, D. Jennens, P. Jenni, J. Jentzsch, C. Jeske, S. Jézéquel, H. Ji, J. Jia, Y. Jiang, S. Jiggins, J. Jimenez Pena, S. Jin, A. Jinaru, O. Jinnouchi, M. D. Joergensen, P. Johansson, K. A. Johns, W. J. Johnson, K. Jon-And, G. Jones, R. W. L. Jones, T. J. Jones, J. Jongmanns, P. M. Jorge, K. D. Joshi, J. Jovicevic, X. Ju, A. Juste Rozas, M. Kaci, A. Kaczmarska, M. Kado, H. Kagan, M. Kagan, S. J. Kahn, E. Kajomovitz, C. W. Kalderon, A. Kaluza, S. Kama, A. Kamenshchikov, N. Kanaya, S. Kaneti, V. A. Kantserov, J. Kanzaki, B. Kaplan, L. S. Kaplan, A. Kapliy, D. Kar, K. Karakostas, A. Karamaoun, N. Karastathis, M. J. Kareem, E. Karentzos, M. Karnevskiy, S. N. Karpov, Z. M. Karpova, K. Karthik, V. Kartvelishvili, A. N. Karyukhin, K. Kasahara, L. Kashif, R. D. Kass, A. Kastanas, Y. Kataoka, C. Kato, A. Katre, J. Katzy, K. Kawade, K. Kawagoe, T. Kawamoto, G. Kawamura, S. Kazama, V. F. Kazanin, R. Keeler, R. Kehoe, J. S. Keller, J. J. Kempster, H. Keoshkerian, O. Kepka, B. P. Kerševan, S. Kersten, R. A. Keyes, F. Khalil-zada, H. Khandanyan, A. Khanov, A. G. Kharlamov, T. J. Khoo, V. Khovanskiy, E. Khramov, J. Khubua, S. Kido, H. Y. Kim, S. H. Kim, Y. K. Kim, N. Kimura, O. M. Kind, B. T. King, M. King, S. B. King, J. Kirk, A. E. Kiryunin, T. Kishimoto, D. Kisielewska, F. Kiss, K. Kiuchi, O. Kivernyk, E. Kladiva, M. H. Klein, M. Klein, U. Klein, K. Kleinknecht, P. Klimek, A. Klimentov, R. Klingenberg, J. A. Klinger, T. Klioutchnikova, E.-E. Kluge, P. Kluit, S. Kluth, J. Knapik, E. Kneringer, E. B. F. G. Knoops, A. Knue, A. Kobayashi, D. Kobayashi, T. Kobayashi, M. Kobel, M. Kocian, P. Kodys, T. Koffas, E. Koffeman, L. A. Kogan, S. Kohlmann, Z. Kohout, T. Kohriki, T. Koi, H. Kolanoski, M. Kolb, I. Koletsou, A. A. Komar, Y. Komori, T. Kondo, N. Kondrashova, K. Köneke, A. C. König, T. Kono, R. Konoplich, N. Konstantinidis, R. Kopeliansky, S. Koperny, L. Köpke, A. K. Kopp, K. Korcyl, K. Kordas, A. Korn, A. A. Korol, I. Korolkov, E. V. Korolkova, O. Kortner, S. Kortner, T. Kosek, V. V. Kostyukhin, V. M. Kotov, A. Kotwal, A. Kourkoumeli-Charalampidi, C. Kourkoumelis, V. Kouskoura, A. Koutsman, R. Kowalewski, T. Z. Kowalski, W. Kozanecki, A. S. Kozhin, V. A. Kramarenko, G. Kramberger, D. Krasnopevtsev, M. W. Krasny, A. Krasznahorkay, J. K. Kraus, A. Kravchenko, S. Kreiss, M. Kretz, J. Kretzschmar, K. Kreutzfeldt, P. Krieger, K. Krizka, K. Kroeninger, H. Kroha, J. Kroll, J. Kroseberg, J. Krstic, U. Kruchonak, H. Krüger, N. Krumnack, A. Kruse, M. C. Kruse, M. Kruskal, T. Kubota, H. Kucuk, S. Kuday, S. Kuehn, A. Kugel, F. Kuger, A. Kuhl, T. Kuhl, V. Kukhtin, R. Kukla, Y. Kulchitsky, S. Kuleshov, M. Kuna, T. Kunigo, A. Kupco, H. Kurashige, Y. A. Kurochkin, V. Kus, E. S. Kuwertz, M. Kuze, J. Kvita, T. Kwan, D. Kyriazopoulos, A. La Rosa, J. L. La Rosa Navarro, L. La Rotonda, C. Lacasta, F. Lacava, J. Lacey, H. Lacker, D. Lacour, V. R. Lacuesta, E. Ladygin, R. Lafaye, B. Laforge, T. Lagouri, S. Lai, L. Lambourne, S. Lammers, C. L. Lampen, W. Lampl, E. Lançon, U. Landgraf, M. P. J. Landon, V. S. Lang, J. C. Lange, A. J. Lankford, F. Lanni, K. Lantzsch, A. Lanza, S. Laplace, C. Lapoire, J. F. Laporte, T. Lari, F. Lasagni Manghi, M. Lassnig, P. Laurelli, W. Lavrijsen, A. T. Law, P. Laycock, T. Lazovich, O. Le Dortz, E. Le Guirriec, E. Le Menedeu, M. LeBlanc, T. LeCompte, F. Ledroit-Guillon, C. A. Lee, S. C. Lee, L. Lee, G. Lefebvre, M. Lefebvre, F. Legger, C. Leggett, A. Lehan, G. Lehmann Miotto, X. Lei, W. A. Leight, A. Leisos, A. G. Leister, M. A. L. Leite, R. Leitner, D. Lellouch, B. Lemmer, K. J. C. Leney, T. Lenz, B. Lenzi, R. Leone, S. Leone, C. Leonidopoulos, S. Leontsinis, C. Leroy, C. G. Lester, M. Levchenko, J. Levêque, D. Levin, L. J. Levinson, M. Levy, A. Lewis, A. M. Leyko, M. Leyton, B. Li, H. Li, H. L. Li, L. Li, L. Li, S. Li, X. Li, Y. Li, Z. Liang, H. Liao, B. Liberti, A. Liblong, P. Lichard, K. Lie, J. Liebal, W. Liebig, C. Limbach, A. Limosani, S. C. Lin, T. H. Lin, F. Linde, B. E. Lindquist, J. T. Linnemann, E. Lipeles, A. Lipniacka, M. Lisovyi, T. M. Liss, D. Lissauer, A. Lister, A. M. Litke, B. Liu, D. Liu, H. Liu, J. Liu, J. B. Liu, K. Liu, L. Liu, M. Liu, M. Liu, Y. Liu, M. Livan, A. Lleres, J. Llorente Merino, S. L. Lloyd, F. Lo Sterzo, E. Lobodzinska, P. Loch, W. S. Lockman, F. K. Loebinger, A. E. Loevschall-Jensen, K. M. Loew, A. Loginov, T. Lohse, K. Lohwasser, M. Lokajicek, B. A. Long, J. D. Long, R. E. Long, K. A. Looper, L. Lopes, D. Lopez Mateos, B. Lopez Paredes, I. Lopez Paz, J. Lorenz, N. Lorenzo Martinez, M. Losada, P. J. Lösel, X. Lou, A. Lounis, J. Love, P. A. Love, H. Lu, N. Lu, H. J. Lubatti, C. Luci, A. Lucotte, C. Luedtke, F. Luehring, W. Lukas, L. Luminari, O. Lundberg, B. Lund-Jensen, D. Lynn, R. Lysak, E. Lytken, H. Ma, L. L. Ma, G. Maccarrone, A. Macchiolo, C. M. Macdonald, B. Maček, J. Machado Miguens, D. Macina, D. Madaffari, R. Madar, H. J. Maddocks, W. F. Mader, A. Madsen, J. Maeda, S. Maeland, T. Maeno, A. Maevskiy, E. Magradze, K. Mahboubi, J. Mahlstedt, C. Maiani, C. Maidantchik, A. A. Maier, T. Maier, A. Maio, S. Majewski, Y. Makida, N. Makovec, B. Malaescu, Pa. Malecki, V. P. Maleev, F. Malek, U. Mallik, D. Malon, C. Malone, S. Maltezos, V. M. Malyshev, S. Malyukov, J. Mamuzic, G. Mancini, B. Mandelli, L. Mandelli, I. Mandić, R. Mandrysch, J. Maneira, L. Manhaes de Andrade Filho, J. Manjarres Ramos, A. Mann, A. Manousakis-Katsikakis, B. Mansoulie, R. Mantifel, M. Mantoani, L. Mapelli, L. March, G. Marchiori, M. Marcisovsky, C. P. Marino, M. Marjanovic, D. E. Marley, F. Marroquim, S. P. Marsden, Z. Marshall, L. F. Marti, S. Marti-Garcia, B. Martin, T. A. Martin, V. J. Martin, B. Martin dit Latour, M. Martinez, S. Martin-Haugh, V. S. Martoiu, A. C. Martyniuk, M. Marx, F. Marzano, A. Marzin, L. Masetti, T. Mashimo, R. Mashinistov, J. Masik, A. L. Maslennikov, I. Massa, L. Massa, P. Mastrandrea, A. Mastroberardino, T. Masubuchi, P. Mättig, J. Mattmann, J. Maurer, S. J. Maxfield, D. A. Maximov, R. Mazini, S. M. Mazza, G. Mc Goldrick, S. P. Mc Kee, A. McCarn, R. L. McCarthy, T. G. McCarthy, N. A. McCubbin, K. W. McFarlane, J. A. Mcfayden, G. Mchedlidze, S. J. McMahon, R. A. McPherson, M. Medinnis, S. Meehan, S. Mehlhase, A. Mehta, K. Meier, C. Meineck, B. Meirose, B. R. Mellado Garcia, F. Meloni, A. Mengarelli, S. Menke, E. Meoni, K. M. Mercurio, S. Mergelmeyer, P. Mermod, L. Merola, C. Meroni, F. S. Merritt, A. Messina, J. Metcalfe, A. S. Mete, C. Meyer, C. Meyer, J.-P. Meyer, J. Meyer, H. Meyer Zu Theenhausen, R. P. Middleton, S. Miglioranzi, L. Mijović, G. Mikenberg, M. Mikestikova, M. Mikuž, M. Milesi, A. Milic, D. W. Miller, C. Mills, A. Milov, D. A. Milstead, A. A. Minaenko, Y. Minami, I. A. Minashvili, A. I. Mincer, B. Mindur, M. Mineev, Y. Ming, L. M. Mir, K. P. Mistry, T. Mitani, J. Mitrevski, V. A. Mitsou, A. Miucci, P. S. Miyagawa, J. U. Mjörnmark, T. Moa, K. Mochizuki, S. Mohapatra, W. Mohr, S. Molander, R. Moles-Valls, R. Monden, M. C. Mondragon, K. Mönig, C. Monini, J. Monk, E. Monnier, A. Montalbano, J. Montejo Berlingen, F. Monticelli, S. Monzani, R. W. Moore, N. Morange, D. Moreno, M. Moreno Llácer, P. Morettini, D. Mori, T. Mori, M. Morii, M. Morinaga, V. Morisbak, S. Moritz, A. K. Morley, G. Mornacchi, J. D. Morris, S. S. Mortensen, A. Morton, L. Morvaj, M. Mosidze, J. Moss, K. Motohashi, R. Mount, E. Mountricha, S. V. Mouraviev, E. J. W. Moyse, S. Muanza, R. D. Mudd, F. Mueller, J. Mueller, R. S. P. Mueller, T. Mueller, D. Muenstermann, P. Mullen, G. A. Mullier, F. J. Munoz Sanchez, J. A. Murillo Quijada, W. J. Murray, H. Musheghyan, E. Musto, A. G. Myagkov, M. Myska, B. P. Nachman, O. Nackenhorst, J. Nadal, K. Nagai, R. Nagai, Y. Nagai, K. Nagano, A. Nagarkar, Y. Nagasaka, K. Nagata, M. Nagel, E. Nagy, A. M. Nairz, Y. Nakahama, K. Nakamura, T. Nakamura, I. Nakano, H. Namasivayam, R. F. Naranjo Garcia, R. Narayan, D. I. Narrias Villar, T. Naumann, G. Navarro, R. Nayyar, H. A. Neal, P. Yu. Nechaeva, T. J. Neep, P. D. Nef, A. Negri, M. Negrini, S. Nektarijevic, C. Nellist, A. Nelson, S. Nemecek, P. Nemethy, A. A. Nepomuceno, M. Nessi, M. S. Neubauer, M. Neumann, R. M. Neves, P. Nevski, P. R. Newman, D. H. Nguyen, R. B. Nickerson, R. Nicolaidou, B. Nicquevert, J. Nielsen, N. Nikiforou, A. Nikiforov, V. Nikolaenko, I. Nikolic-Audit, K. Nikolopoulos, J. K. Nilsen, P. Nilsson, Y. Ninomiya, A. Nisati, R. Nisius, T. Nobe, L. Nodulman, M. Nomachi, I. Nomidis, T. Nooney, S. Norberg, M. Nordberg, O. Novgorodova, S. Nowak, M. Nozaki, L. Nozka, K. Ntekas, G. Nunes Hanninger, T. Nunnemann, E. Nurse, F. Nuti, F. O’grady, D. C. O’Neil, V. O’Shea, F. G. Oakham, H. Oberlack, T. Obermann, J. Ocariz, A. Ochi, I. Ochoa, J. P. Ochoa-Ricoux, S. Oda, S. Odaka, H. Ogren, A. Oh, S. H. Oh, C. C. Ohm, H. Ohman, H. Oide, W. Okamura, H. Okawa, Y. Okumura, T. Okuyama, A. Olariu, S. A. Olivares Pino, D. Oliveira Damazio, A. Olszewski, J. Olszowska, A. Onofre, K. Onogi, P. U. E. Onyisi, C. J. Oram, M. J. Oreglia, Y. Oren, D. Orestano, N. Orlando, C. Oropeza Barrera, R. S. Orr, B. Osculati, R. Ospanov, G. Otero y Garzon, H. Otono, M. Ouchrif, F. Ould-Saada, A. Ouraou, K. P. Oussoren, Q. Ouyang, A. Ovcharova, M. Owen, R. E. Owen, V. E. Ozcan, N. Ozturk, K. Pachal, A. Pacheco Pages, C. Padilla Aranda, M. Pagáčová, S. Pagan Griso, E. Paganis, F. Paige, P. Pais, K. Pajchel, G. Palacino, S. Palestini, M. Palka, D. Pallin, A. Palma, Y. B. Pan, E. St. Panagiotopoulou, C. E. Pandini, J. G. Panduro Vazquez, P. Pani, S. Panitkin, D. Pantea, L. Paolozzi, Th. D. Papadopoulou, K. Papageorgiou, A. Paramonov, D. Paredes Hernandez, M. A. Parker, K. A. Parker, F. Parodi, J. A. Parsons, U. Parzefall, E. Pasqualucci, S. Passaggio, F. Pastore, Fr. Pastore, G. Pásztor, S. Pataraia, N. D. Patel, J. R. Pater, T. Pauly, J. Pearce, B. Pearson, L. E. Pedersen, M. Pedersen, S. Pedraza Lopez, R. Pedro, S. V. Peleganchuk, D. Pelikan, O. Penc, C. Peng, H. Peng, B. Penning, J. Penwell, D. V. Perepelitsa, E. Perez Codina, M. T. Pérez García-Estañ, L. Perini, H. Pernegger, S. Perrella, R. Peschke, V. D. Peshekhonov, K. Peters, R. F. Y. Peters, B. A. Petersen, T. C. Petersen, E. Petit, A. Petridis, C. Petridou, P. Petroff, E. Petrolo, F. Petrucci, N. E. Pettersson, R. Pezoa, P. W. Phillips, G. Piacquadio, E. Pianori, A. Picazio, E. Piccaro, M. Piccinini, M. A. Pickering, R. Piegaia, D. T. Pignotti, J. E. Pilcher, A. D. Pilkington, A. W. J. Pin, J. Pina, M. Pinamonti, J. L. Pinfold, A. Pingel, S. Pires, H. Pirumov, M. Pitt, C. Pizio, L. Plazak, M.-A. Pleier, V. Pleskot, E. Plotnikova, P. Plucinski, D. Pluth, R. Poettgen, L. Poggioli, D. Pohl, G. Polesello, A. Poley, A. Policicchio, R. Polifka, A. Polini, C. S. Pollard, V. Polychronakos, K. Pommès, L. Pontecorvo, B. G. Pope, G. A. Popeneciu, D. S. Popovic, A. Poppleton, S. Pospisil, K. Potamianos, I. N. Potrap, C. J. Potter, C. T. Potter, G. Poulard, J. Poveda, V. Pozdnyakov, M. E. Pozo Astigarraga, P. Pralavorio, A. Pranko, S. Prasad, S. Prell, D. Price, L. E. Price, M. Primavera, S. Prince, M. Proissl, K. Prokofiev, F. Prokoshin, E. Protopapadaki, S. Protopopescu, J. Proudfoot, M. Przybycien, E. Ptacek, D. Puddu, E. Pueschel, D. Puldon, M. Purohit, P. Puzo, J. Qian, G. Qin, Y. Qin, A. Quadt, D. R. Quarrie, W. B. Quayle, M. Queitsch-Maitland, D. Quilty, S. Raddum, V. Radeka, V. Radescu, S. K. Radhakrishnan, P. Radloff, P. Rados, F. Ragusa, G. Rahal, S. Rajagopalan, M. Rammensee, C. Rangel-Smith, F. Rauscher, S. Rave, T. Ravenscroft, M. Raymond, A. L. Read, N. P. Readioff, D. M. Rebuzzi, A. Redelbach, G. Redlinger, R. Reece, K. Reeves, L. Rehnisch, J. Reichert, H. Reisin, C. Rembser, H. Ren, A. Renaud, M. Rescigno, S. Resconi, O. L. Rezanova, P. Reznicek, R. Rezvani, R. Richter, S. Richter, E. Richter-Was, O. Ricken, M. Ridel, P. Rieck, C. J. Riegel, J. Rieger, O. Rifki, M. Rijssenbeek, A. Rimoldi, L. Rinaldi, B. Ristić, E. Ritsch, I. Riu, F. Rizatdinova, E. Rizvi, S. H. Robertson, A. Robichaud-Veronneau, D. Robinson, J. E. M. Robinson, A. Robson, C. Roda, S. Roe, O. Røhne, A. Romaniouk, M. Romano, S. M. Romano Saez, E. Romero Adam, N. Rompotis, M. Ronzani, L. Roos, E. Ros, S. Rosati, K. Rosbach, P. Rose, O. Rosenthal, V. Rossetti, E. Rossi, L. P. Rossi, J. H. N. Rosten, R. Rosten, M. Rotaru, I. Roth, J. Rothberg, D. Rousseau, C. R. Royon, A. Rozanov, Y. Rozen, X. Ruan, F. Rubbo, I. Rubinskiy, V. I. Rud, C. Rudolph, M. S. Rudolph, F. Rühr, A. Ruiz-Martinez, Z. Rurikova, N. A. Rusakovich, A. Ruschke, H. L. Russell, J. P. Rutherfoord, N. Ruthmann, Y. F. Ryabov, M. Rybar, G. Rybkin, N. C. Ryder, A. Ryzhov, A. F. Saavedra, G. Sabato, S. Sacerdoti, A. Saddique, H. F.-W. Sadrozinski, R. Sadykov, F. Safai Tehrani, P. Saha, M. Sahinsoy, M. Saimpert, T. Saito, H. Sakamoto, Y. Sakurai, G. Salamanna, A. Salamon, J. E. Salazar Loyola, M. Saleem, D. Salek, P. H. Sales De Bruin, D. Salihagic, A. Salnikov, J. Salt, D. Salvatore, F. Salvatore, A. Salvucci, A. Salzburger, D. Sammel, D. Sampsonidis, A. Sanchez, J. Sánchez, V. Sanchez Martinez, H. Sandaker, R. L. Sandbach, H. G. Sander, M. P. Sanders, M. Sandhoff, C. Sandoval, R. Sandstroem, D. P. C. Sankey, M. Sannino, A. Sansoni, C. Santoni, R. Santonico, H. Santos, I. Santoyo Castillo, K. Sapp, A. Sapronov, J. G. Saraiva, B. Sarrazin, O. Sasaki, Y. Sasaki, K. Sato, G. Sauvage, E. Sauvan, G. Savage, P. Savard, C. Sawyer, L. Sawyer, J. Saxon, C. Sbarra, A. Sbrizzi, T. Scanlon, D. A. Scannicchio, M. Scarcella, V. Scarfone, J. Schaarschmidt, P. Schacht, D. Schaefer, R. Schaefer, J. Schaeffer, S. Schaepe, S. Schaetzel, U. Schäfer, A. C. Schaffer, D. Schaile, R. D. Schamberger, V. Scharf, V. A. Schegelsky, D. Scheirich, M. Schernau, C. Schiavi, C. Schillo, M. Schioppa, S. Schlenker, K. Schmieden, C. Schmitt, S. Schmitt, S. Schmitt, S. Schmitz, B. Schneider, Y. J. Schnellbach, U. Schnoor, L. Schoeffel, A. Schoening, B. D. Schoenrock, E. Schopf, A. L. S. Schorlemmer, M. Schott, D. Schouten, J. Schovancova, S. Schramm, M. Schreyer, N. Schuh, M. J. Schultens, H.-C. Schultz-Coulon, H. Schulz, M. Schumacher, B. A. Schumm, Ph. Schune, C. Schwanenberger, A. Schwartzman, T. A. Schwarz, Ph. Schwegler, H. Schweiger, Ph. Schwemling, R. Schwienhorst, J. Schwindling, T. Schwindt, E. Scifo, G. Sciolla, F. Scuri, F. Scutti, J. Searcy, G. Sedov, E. Sedykh, P. Seema, S. C. Seidel, A. Seiden, F. Seifert, J. M. Seixas, G. Sekhniaidze, K. Sekhon, S. J. Sekula, D. M. Seliverstov, N. Semprini-Cesari, C. Serfon, L. Serin, L. Serkin, T. Serre, M. Sessa, R. Seuster, H. Severini, T. Sfiligoj, F. Sforza, A. Sfyrla, E. Shabalina, M. Shamim, L. Y. Shan, R. Shang, J. T. Shank, M. Shapiro, P. B. Shatalov, K. Shaw, S. M. Shaw, A. Shcherbakova, C. Y. Shehu, P. Sherwood, L. Shi, S. Shimizu, C. O. Shimmin, M. Shimojima, M. Shiyakova, A. Shmeleva, D. Shoaleh Saadi, M. J. Shochet, S. Shojaii, S. Shrestha, E. Shulga, M. A. Shupe, P. Sicho, P. E. Sidebo, O. Sidiropoulou, D. Sidorov, A. Sidoti, F. Siegert, Dj. Sijacki, J. Silva, Y. Silver, S. B. Silverstein, V. Simak, O. Simard, Lj. Simic, S. Simion, E. Simioni, B. Simmons, D. Simon, M. Simon, P. Sinervo, N. B. Sinev, M. Sioli, G. Siragusa, A. N. Sisakyan, S. Yu. Sivoklokov, J. Sjölin, T. B. Sjursen, M. B. Skinner, H. P. Skottowe, P. Skubic, M. Slater, T. Slavicek, M. Slawinska, K. Sliwa, V. Smakhtin, B. H. Smart, L. Smestad, S. Yu. Smirnov, Y. Smirnov, L. N. Smirnova, O. Smirnova, M. N. K. Smith, R. W. Smith, M. Smizanska, K. Smolek, A. A. Snesarev, G. Snidero, S. Snyder, R. Sobie, F. Socher, A. Soffer, D. A. Soh, G. Sokhrannyi, C. A. Solans, M. Solar, J. Solc, E. Yu. Soldatov, U. Soldevila, A. A. Solodkov, A. Soloshenko, O. V. Solovyanov, V. Solovyev, P. Sommer, H. Y. Song, N. Soni, A. Sood, A. Sopczak, B. Sopko, V. Sopko, V. Sorin, D. Sosa, M. Sosebee, C. L. Sotiropoulou, R. Soualah, A. M. Soukharev, D. South, B. C. Sowden, S. Spagnolo, M. Spalla, M. Spangenberg, F. Spanò, W. R. Spearman, D. Sperlich, F. Spettel, R. Spighi, G. Spigo, L. A. Spiller, M. Spousta, R. D. St. Denis, A. Stabile, S. Staerz, J. Stahlman, R. Stamen, S. Stamm, E. Stanecka, R. W. Stanek, C. Stanescu, M. Stanescu-Bellu, M. M. Stanitzki, S. Stapnes, E. A. Starchenko, J. Stark, P. Staroba, P. Starovoitov, R. Staszewski, P. Steinberg, B. Stelzer, H. J. Stelzer, O. Stelzer-Chilton, H. Stenzel, G. A. Stewart, J. A. Stillings, M. C. Stockton, M. Stoebe, G. Stoicea, P. Stolte, S. Stonjek, A. R. Stradling, A. Straessner, M. E. Stramaglia, J. Strandberg, S. Strandberg, A. Strandlie, E. Strauss, M. Strauss, P. Strizenec, R. Ströhmer, D. M. Strom, R. Stroynowski, A. Strubig, S. A. Stucci, B. Stugu, N. A. Styles, D. Su, J. Su, R. Subramaniam, A. Succurro, S. Suchek, Y. Sugaya, M. Suk, V. V. Sulin, S. Sultansoy, T. Sumida, S. Sun, X. Sun, J. E. Sundermann, K. Suruliz, G. Susinno, M. R. Sutton, S. Suzuki, M. Svatos, M. Swiatlowski, I. Sykora, T. Sykora, D. Ta, C. Taccini, K. Tackmann, J. Taenzer, A. Taffard, R. Tafirout, N. Taiblum, H. Takai, R. Takashima, H. Takeda, T. Takeshita, Y. Takubo, M. Talby, A. A. Talyshev, J. Y. C. Tam, K. G. Tan, J. Tanaka, R. Tanaka, S. Tanaka, B. B. Tannenwald, S. Tapia Araya, S. Tapprogge, S. Tarem, F. Tarrade, G. F. Tartarelli, P. Tas, M. Tasevsky, T. Tashiro, E. Tassi, A. Tavares Delgado, Y. Tayalati, A. C. Taylor, F. E. Taylor, G. N. Taylor, P. T. E. Taylor, W. Taylor, F. A. Teischinger, P. Teixeira-Dias, K. K. Temming, D. Temple, H. Ten Kate, P. K. Teng, J. J. Teoh, F. Tepel, S. Terada, K. Terashi, J. Terron, S. Terzo, M. Testa, R. J. Teuscher, T. Theveneaux-Pelzer, J. P. Thomas, J. Thomas-Wilsker, E. N. Thompson, P. D. Thompson, R. J. Thompson, A. S. Thompson, L. A. Thomsen, E. Thomson, M. Thomson, R. P. Thun, M. J. Tibbetts, R. E. Ticse Torres, V. O. Tikhomirov, Yu. A. Tikhonov, S. Timoshenko, E. Tiouchichine, P. Tipton, S. Tisserant, K. Todome, T. Todorov, S. Todorova-Nova, J. Tojo, S. Tokár, K. Tokushuku, K. Tollefson, E. Tolley, L. Tomlinson, M. Tomoto, L. Tompkins, K. Toms, E. Torrence, H. Torres, E. Torró Pastor, J. Toth, F. Touchard, D. R. Tovey, T. Trefzger, L. Tremblet, A. Tricoli, I. M. Trigger, S. Trincaz-Duvoid, M. F. Tripiana, W. Trischuk, B. Trocmé, C. Troncon, M. Trottier-McDonald, M. Trovatelli, L. Truong, M. Trzebinski, A. Trzupek, C. Tsarouchas, J. C.-L. Tseng, P. V. Tsiareshka, D. Tsionou, G. Tsipolitis, N. Tsirintanis, S. Tsiskaridze, V. Tsiskaridze, E. G. Tskhadadze, K. M. Tsui, I. I. Tsukerman, V. Tsulaia, S. Tsuno, D. Tsybychev, A. Tudorache, V. Tudorache, A. N. Tuna, S. A. Tupputi, S. Turchikhin, D. Turecek, R. Turra, A. J. Turvey, P. M. Tuts, A. Tykhonov, M. Tylmad, M. Tyndel, I. Ueda, R. Ueno, M. Ughetto, F. Ukegawa, G. Unal, A. Undrus, G. Unel, F. C. Ungaro, Y. Unno, C. Unverdorben, J. Urban, P. Urquijo, P. Urrejola, G. Usai, A. Usanova, L. Vacavant, V. Vacek, B. Vachon, C. Valderanis, N. Valencic, S. Valentinetti, A. Valero, L. Valery, S. Valkar, S. Vallecorsa, J. A. Valls Ferrer, W. Van Den Wollenberg, P. C. Van Der Deijl, R. van der Geer, H. van der Graaf, N. van Eldik, P. van Gemmeren, J. Van Nieuwkoop, I. van Vulpen, M. C. van Woerden, M. Vanadia, W. Vandelli, R. Vanguri, A. Vaniachine, F. Vannucci, G. Vardanyan, R. Vari, E. W. Varnes, T. Varol, D. Varouchas, A. Vartapetian, K. E. Varvell, F. Vazeille, T. Vazquez Schroeder, J. Veatch, L. M. Veloce, F. Veloso, T. Velz, S. Veneziano, A. Ventura, D. Ventura, M. Venturi, N. Venturi, A. Venturini, V. Vercesi, M. Verducci, W. Verkerke, J. C. Vermeulen, A. Vest, M. C. Vetterli, O. Viazlo, I. Vichou, T. Vickey, O. E. Vickey Boeriu, G. H. A. Viehhauser, S. Viel, R. Vigne, M. Villa, M. Villaplana Perez, E. Vilucchi, M. G. Vincter, V. B. Vinogradov, I. Vivarelli, S. Vlachos, D. Vladoiu, M. Vlasak, M. Vogel, P. Vokac, G. Volpi, M. Volpi, H. von der Schmitt, H. von Radziewski, E. von Toerne, V. Vorobel, K. Vorobev, M. Vos, R. Voss, J. H. Vossebeld, N. Vranjes, M. Vranjes Milosavljevic, V. Vrba, M. Vreeswijk, R. Vuillermet, I. Vukotic, Z. Vykydal, P. Wagner, W. Wagner, H. Wahlberg, S. Wahrmund, J. Wakabayashi, J. Walder, R. Walker, W. Walkowiak, C. Wang, F. Wang, H. Wang, H. Wang, J. Wang, J. Wang, K. Wang, R. Wang, S. M. Wang, T. Wang, T. Wang, X. Wang, C. Wanotayaroj, A. Warburton, C. P. Ward, D. R. Wardrope, A. Washbrook, C. Wasicki, P. M. Watkins, A. T. Watson, I. J. Watson, M. F. Watson, G. Watts, S. Watts, B. M. Waugh, S. Webb, M. S. Weber, S. W. Weber, J. S. Webster, A. R. Weidberg, B. Weinert, J. Weingarten, C. Weiser, H. Weits, P. S. Wells, T. Wenaus, T. Wengler, S. Wenig, N. Wermes, M. Werner, P. Werner, M. Wessels, J. Wetter, K. Whalen, A. M. Wharton, A. White, M. J. White, R. White, S. White, D. Whiteson, F. J. Wickens, W. Wiedenmann, M. Wielers, P. Wienemann, C. Wiglesworth, L. A. M. Wiik-Fuchs, A. Wildauer, H. G. Wilkens, H. H. Williams, S. Williams, C. Willis, S. Willocq, A. Wilson, J. A. Wilson, I. Wingerter-Seez, F. Winklmeier, B. T. Winter, M. Wittgen, J. Wittkowski, S. J. Wollstadt, M. W. Wolter, H. Wolters, B. K. Wosiek, J. Wotschack, M. J. Woudstra, K. W. Wozniak, M. Wu, M. Wu, S. L. Wu, X. Wu, Y. Wu, T. R. Wyatt, B. M. Wynne, S. Xella, D. Xu, L. Xu, B. Yabsley, S. Yacoob, R. Yakabe, M. Yamada, D. Yamaguchi, Y. Yamaguchi, A. Yamamoto, S. Yamamoto, T. Yamanaka, K. Yamauchi, Y. Yamazaki, Z. Yan, H. Yang, H. Yang, Y. Yang, W.-M. Yao, Y. C. Yap, Y. Yasu, E. Yatsenko, K. H. Yau Wong, J. Ye, S. Ye, I. Yeletskikh, A. L. Yen, E. Yildirim, K. Yorita, R. Yoshida, K. Yoshihara, C. Young, C. J. S. Young, S. Youssef, D. R. Yu, J. Yu, J. M. Yu, J. Yu, L. Yuan, S. P. Y. Yuen, A. Yurkewicz, I. Yusuff, B. Zabinski, R. Zaidan, A. M. Zaitsev, J. Zalieckas, A. Zaman, S. Zambito, L. Zanello, D. Zanzi, C. Zeitnitz, M. Zeman, A. Zemla, J. C. Zeng, Q. Zeng, K. Zengel, O. Zenin, T. Ženiš, D. Zerwas, D. Zhang, F. Zhang, G. Zhang, H. Zhang, J. Zhang, L. Zhang, R. Zhang, X. Zhang, Z. Zhang, X. Zhao, Y. Zhao, Z. Zhao, A. Zhemchugov, J. Zhong, B. Zhou, C. Zhou, L. Zhou, L. Zhou, M. Zhou, N. Zhou, C. G. Zhu, H. Zhu, J. Zhu, Y. Zhu, X. Zhuang, K. Zhukov, A. Zibell, D. Zieminska, N. I. Zimine, C. Zimmermann, S. Zimmermann, Z. Zinonos, M. Zinser, M. Ziolkowski, L. Živković, G. Zobernig, A. Zoccoli, M. zur Nedden, G. Zurzolo, L. Zwalinski

**Affiliations:** 1 Department of Physics, University of Adelaide, Adelaide, Australia; 2 Physics Department, SUNY Albany, Albany, NY USA; 3 Department of Physics, University of Alberta, Edmonton, AB Canada; 4 Department of Physics, Ankara University, Ankara, Turkey; 5 Istanbul Aydin University, Istanbul, Turkey; 6 Division of Physics, TOBB University of Economics and Technology, Ankara, Turkey; 7 LAPP, CNRS/IN2P3 and Université Savoie Mont Blanc, Annecy-le-Vieux, France; 8 High Energy Physics Division, Argonne National Laboratory, Argonne, IL USA; 9 Department of Physics, University of Arizona, Tucson, AZ USA; 10 Department of Physics, The University of Texas at Arlington, Arlington, TX USA; 11 Physics Department, University of Athens, Athens, Greece; 12 Physics Department, National Technical University of Athens, Zografou, Greece; 13 Institute of Physics, Azerbaijan Academy of Sciences, Baku, Azerbaijan; 14 Institut de Física d’Altes Energies and Departament de Física de la Universitat Autònoma de Barcelona, Barcelona, Spain; 15 Institute of Physics, University of Belgrade, Belgrade, Serbia; 16 Department for Physics and Technology, University of Bergen, Bergen, Norway; 17 Physics Division, Lawrence Berkeley National Laboratory and University of California, Berkeley, CA USA; 18 Department of Physics, Humboldt University, Berlin, Germany; 19 Albert Einstein Center for Fundamental Physics and Laboratory for High Energy Physics, University of Bern, Bern, Switzerland; 20 School of Physics and Astronomy, University of Birmingham, Birmingham, UK; 21 Department of Physics, Bogazici University, Istanbul, Turkey; 22 Department of Physics Engineering, Gaziantep University, Gaziantep, Turkey; 23 Department of Physics, Dogus University, Istanbul, Turkey; 24 INFN Sezione di Bologna, Bologna, Italy; 25 Dipartimento di Fisica e Astronomia, Università di Bologna, Bologna, Italy; 26 Physikalisches Institut, University of Bonn, Bonn, Germany; 27 Department of Physics, Boston University, Boston, MA USA; 28 Department of Physics, Brandeis University, Waltham, MA USA; 29 Universidade Federal do Rio De Janeiro COPPE/EE/IF, Rio de Janeiro, Brazil; 30 Electrical Circuits Department, Federal University of Juiz de Fora (UFJF), Juiz de Fora, Brazil; 31 Federal University of Sao Joao del Rei (UFSJ), Sao Joao del Rei, Brazil; 32 Instituto de Fisica, Universidade de Sao Paulo, São Paulo, Brazil; 33 Physics Department, Brookhaven National Laboratory, Upton, NY USA; 34 Transilvania University of Brasov, National Institute of Physics and Nuclear Engineering, Brasov, Romania; 35 National Institute of Physics and Nuclear Engineering, Bucharest, Romania; 36 Physics Department, National Institute for Research and Development of Isotopic and Molecular Technologies, Cluj Napoca, Romania; 37 University Politehnica Bucharest, Bucharest, Romania; 38 West University in Timisoara, Timisoara, Romania; 39 Departamento de Física, Universidad de Buenos Aires, Buenos Aires, Argentina; 40 Cavendish Laboratory, University of Cambridge, Cambridge, UK; 41 Department of Physics, Carleton University, Ottawa, ON Canada; 42 CERN, Geneva, Switzerland; 43 Enrico Fermi Institute, University of Chicago, Chicago, IL USA; 44 Departamento de Física, Pontificia Universidad Católica de Chile, Santiago, Chile; 45 Departamento de Física, Universidad Técnica Federico Santa María, Valparaiso, Chile; 46 Institute of High Energy Physics, Chinese Academy of Sciences, Beijing, China; 47 Department of Modern Physics, University of Science and Technology of China, Hefei, Anhui China; 48 Department of Physics, Nanjing University, Nanjing, Jiangsu China; 49 School of Physics, Shandong University, Jinan, Shandong China; 50 Shanghai Key Laboratory for Particle Physics and Cosmology, Department of Physics and Astronomy, Shanghai Jiao Tong University, Shanghai, China; 51 Physics Department, Tsinghua University, Beijing, 100084 China; 52 Laboratoire de Physique Corpusculaire, Clermont Université and Université Blaise Pascal and CNRS/IN2P3, Clermont-Ferrand, France; 53 Nevis Laboratory, Columbia University, Irvington, NY USA; 54 Niels Bohr Institute, University of Copenhagen, Copenhagen, Denmark; 55 INFN Gruppo Collegato di Cosenza, Laboratori Nazionali di Frascati, Frascati, Italy; 56 Dipartimento di Fisica, Università della Calabria, Rende, Italy; 57 AGH University of Science and Technology, Faculty of Physics and Applied Computer Science, Kraków, Poland; 58 Marian Smoluchowski Institute of Physics, Jagiellonian University, Kraków, Poland; 59 Institute of Nuclear Physics, Polish Academy of Sciences, Kraków, Poland; 60 Physics Department, Southern Methodist University, Dallas, TX USA; 61 Physics Department, University of Texas at Dallas, Richardson, TX USA; 62 DESY, Hamburg and Zeuthen, Germany; 63 Institut für Experimentelle Physik IV, Technische Universität Dortmund, Dortmund, Germany; 64 Institut für Kern- und Teilchenphysik, Technische Universität Dresden, Dresden, Germany; 65 Department of Physics, Duke University, Durham, NC USA; 66 SUPA-School of Physics and Astronomy, University of Edinburgh, Edinburgh, UK; 67 INFN Laboratori Nazionali di Frascati, Frascati, Italy; 68 Fakultät für Mathematik und Physik, Albert-Ludwigs-Universität, Freiburg, Germany; 69 Section de Physique, Université de Genève, Geneva, Switzerland; 70 INFN Sezione di Genova, Genoa, Italy; 71 Dipartimento di Fisica, Università di Genova, Genoa, Italy; 72 E. Andronikashvili Institute of Physics, Iv. Javakhishvili Tbilisi State University, Tbilisi, Georgia; 73 High Energy Physics Institute, Tbilisi State University, Tbilisi, Georgia; 74 II Physikalisches Institut, Justus-Liebig-Universität Giessen, Giessen, Germany; 75 SUPA-School of Physics and Astronomy, University of Glasgow, Glasgow, UK; 76 II Physikalisches Institut, Georg-August-Universität, Göttingen, Germany; 77 Laboratoire de Physique Subatomique et de Cosmologie, Université Grenoble-Alpes, CNRS/IN2P3, Grenoble, France; 78 Department of Physics, Hampton University, Hampton, VA USA; 79 Laboratory for Particle Physics and Cosmology, Harvard University, Cambridge, MA USA; 80 Kirchhoff-Institut für Physik, Ruprecht-Karls-Universität Heidelberg, Heidelberg, Germany; 81 Physikalisches Institut, Ruprecht-Karls-Universität Heidelberg, Heidelberg, Germany; 82 ZITI Institut für technische Informatik, Ruprecht-Karls-Universität Heidelberg, Mannheim, Germany; 83 Faculty of Applied Information Science, Hiroshima Institute of Technology, Hiroshima, Japan; 84 Department of Physics, The Chinese University of Hong Kong, Shatin, NT Hong Kong; 85 Department of Physics, The University of Hong Kong, Pokfulam, Hong Kong; 86 Department of Physics, The Hong Kong University of Science and Technology, Clear Water Bay, Kowloon, Hong Kong China; 87 Department of Physics, Indiana University, Bloomington, IN USA; 88 Institut für Astro- und Teilchenphysik, Leopold-Franzens-Universität, Innsbruck, Austria; 89 University of Iowa, Iowa City, IA USA; 90 Department of Physics and Astronomy, Iowa State University, Ames, IA USA; 91 Joint Institute for Nuclear Research, JINR Dubna, Dubna, Russia; 92 KEK, High Energy Accelerator Research Organization, Tsukuba, Japan; 93 Graduate School of Science, Kobe University, Kobe, Japan; 94 Faculty of Science, Kyoto University, Kyoto, Japan; 95 Kyoto University of Education, Kyoto, Japan; 96 Department of Physics, Kyushu University, Fukuoka, Japan; 97 Instituto de Física La Plata, Universidad Nacional de La Plata and CONICET, La Plata, Argentina; 98 Physics Department, Lancaster University, Lancaster, UK; 99 INFN Sezione di Lecce, Lecce, Italy; 100 Dipartimento di Matematica e Fisica, Università del Salento, Lecce, Italy; 101 Oliver Lodge Laboratory, University of Liverpool, Liverpool, UK; 102 Department of Physics, Jožef Stefan Institute and University of Ljubljana, Ljubljana, Slovenia; 103 School of Physics and Astronomy, Queen Mary University of London, London, UK; 104 Department of Physics, Royal Holloway University of London, Surrey, UK; 105 Department of Physics and Astronomy, University College London, London, UK; 106 Louisiana Tech University, Ruston, LA USA; 107 Laboratoire de Physique Nucléaire et de Hautes Energies, UPMC and Université Paris-Diderot and CNRS/IN2P3, Paris, France; 108 Fysiska institutionen, Lunds universitet, Lund, Sweden; 109 Departamento de Fisica Teorica C-15, Universidad Autonoma de Madrid, Madrid, Spain; 110 Institut für Physik, Universität Mainz, Mainz, Germany; 111 School of Physics and Astronomy, University of Manchester, Manchester, UK; 112 CPPM, Aix-Marseille Université and CNRS/IN2P3, Marseille, France; 113 Department of Physics, University of Massachusetts, Amherst, MA USA; 114 Department of Physics, McGill University, Montreal, QC Canada; 115 School of Physics, University of Melbourne, Melbourne, VIC Australia; 116 Department of Physics, The University of Michigan, Ann Arbor, MI USA; 117 Department of Physics and Astronomy, Michigan State University, East Lansing, MI USA; 118 INFN Sezione di Milano, Milan, Italy; 119 Dipartimento di Fisica, Università di Milano, Milan, Italy; 120 B.I. Stepanov Institute of Physics, National Academy of Sciences of Belarus, Minsk, Republic of Belarus; 121 National Scientific and Educational Centre for Particle and High Energy Physics, Minsk, Republic of Belarus; 122 Department of Physics, Massachusetts Institute of Technology, Cambridge, MA USA; 123 Group of Particle Physics, University of Montreal, Montreal, QC Canada; 124 P.N. Lebedev Institute of Physics, Academy of Sciences, Moscow, Russia; 125 Institute for Theoretical and Experimental Physics (ITEP), Moscow, Russia; 126 National Research Nuclear University MEPhI, Moscow, Russia; 127 D.V. Skobeltsyn Institute of Nuclear Physics, M.V. Lomonosov Moscow State University, Moscow, Russia; 128 Fakultät für Physik, Ludwig-Maximilians-Universität München, Munich, Germany; 129 Max-Planck-Institut für Physik (Werner-Heisenberg-Institut), Munich, Germany; 130 Nagasaki Institute of Applied Science, Nagasaki, Japan; 131 Graduate School of Science and Kobayashi-Maskawa Institute, Nagoya University, Nagoya, Japan; 132 INFN Sezione di Napoli, Naples, Italy; 133 Dipartimento di Fisica, Università di Napoli, Naples, Italy; 134 Department of Physics and Astronomy, University of New Mexico, Albuquerque, NM USA; 135 Institute for Mathematics, Astrophysics and Particle Physics, Radboud University Nijmegen/Nikhef, Nijmegen, The Netherlands; 136 Nikhef National Institute for Subatomic Physics and University of Amsterdam, Amsterdam, The Netherlands; 137 Department of Physics, Northern Illinois University, De Kalb, IL USA; 138 Budker Institute of Nuclear Physics, SB RAS, Novosibirsk, Russia; 139 Department of Physics, New York University, New York, NY USA; 140 Ohio State University, Columbus, OH USA; 141 Faculty of Science, Okayama University, Okayama, Japan; 142 Homer L. Dodge Department of Physics and Astronomy, University of Oklahoma, Norman, OK USA; 143 Department of Physics, Oklahoma State University, Stillwater, OK USA; 144 Palacký University, RCPTM, Olomouc, Czech Republic; 145 Center for High Energy Physics, University of Oregon, Eugene, OR USA; 146 LAL, Université Paris-Sud and CNRS/IN2P3, Orsay, France; 147 Graduate School of Science, Osaka University, Osaka, Japan; 148 Department of Physics, University of Oslo, Oslo, Norway; 149 Department of Physics, Oxford University, Oxford, UK; 150 INFN Sezione di Pavia, Pavia, Italy; 151 Dipartimento di Fisica, Università di Pavia, Pavia, Italy; 152 Department of Physics, University of Pennsylvania, Philadelphia, PA USA; 153 National Research Centre “Kurchatov Institute” B.P.Konstantinov Petersburg Nuclear Physics Institute, St. Petersburg, Russia; 154 INFN Sezione di Pisa, Pisa, Italy; 155 Dipartimento di Fisica E. Fermi, Università di Pisa, Pisa, Italy; 156 Department of Physics and Astronomy, University of Pittsburgh, Pittsburgh, PA USA; 157 Laboratório de Instrumentação e Física Experimental de Partículas-LIP, Lisbon, Portugal; 158 Faculdade de Ciências, Universidade de Lisboa, Lisbon, Portugal; 159 Department of Physics, University of Coimbra, Coimbra, Portugal; 160 Centro de Física Nuclear da Universidade de Lisboa, Lisbon, Portugal; 161 Departamento de Fisica, Universidade do Minho, Braga, Portugal; 162 Departamento de Fisica Teorica y del Cosmos and CAFPE, Universidad de Granada, Granada, Spain; 163 Dep Fisica and CEFITEC of Faculdade de Ciencias e Tecnologia, Universidade Nova de Lisboa, Caparica, Portugal; 164 Institute of Physics, Academy of Sciences of the Czech Republic, Prague, Czech Republic; 165 Czech Technical University in Prague, Prague, Czech Republic; 166 Faculty of Mathematics and Physics, Charles University in Prague, Prague, Czech Republic; 167 State Research Center Institute for High Energy Physics, Protvino, NRC KI Russia; 168 Particle Physics Department, Rutherford Appleton Laboratory, Didcot, UK; 169 INFN Sezione di Roma, Rome, Italy; 170 Dipartimento di Fisica, Sapienza Università di Roma, Rome, Italy; 171 INFN Sezione di Roma Tor Vergata, Rome, Italy; 172 Dipartimento di Fisica, Università di Roma Tor Vergata, Rome, Italy; 173 INFN Sezione di Roma Tre, Rome, Italy; 174 Dipartimento di Matematica e Fisica, Università Roma Tre, Rome, Italy; 175 Faculté des Sciences Ain Chock, Réseau Universitaire de Physique des Hautes Energies-Université Hassan II, Casablanca, Morocco; 176 Centre National de l’Energie des Sciences Techniques Nucleaires, Rabat, Morocco; 177 Faculté des Sciences Semlalia, Université Cadi Ayyad, LPHEA-Marrakech, Marrakech, Morocco; 178 Faculté des Sciences, Université Mohamed Premier and LPTPM, Oujda, Morocco; 179 Faculté des Sciences, Université Mohammed V, Rabat, Morocco; 180 DSM/IRFU (Institut de Recherches sur les Lois Fondamentales de l’Univers), CEA Saclay (Commissariat à l’Energie Atomique et aux Energies Alternatives), Gif-sur-Yvette, France; 181 Santa Cruz Institute for Particle Physics, University of California Santa Cruz, Santa Cruz, CA USA; 182 Department of Physics, University of Washington, Seattle, WA USA; 183 Department of Physics and Astronomy, University of Sheffield, Sheffield, UK; 184 Department of Physics, Shinshu University, Nagano, Japan; 185 Fachbereich Physik, Universität Siegen, Siegen, Germany; 186 Department of Physics, Simon Fraser University, Burnaby, BC Canada; 187 SLAC National Accelerator Laboratory, Stanford, CA USA; 188 Faculty of Mathematics, Physics and Informatics, Comenius University, Bratislava, Slovak Republic; 189 Department of Subnuclear Physics, Institute of Experimental Physics of the Slovak Academy of Sciences, Kosice, Slovak Republic; 190 Department of Physics, University of Cape Town, Cape Town, South Africa; 191 Department of Physics, University of Johannesburg, Johannesburg, South Africa; 192 School of Physics, University of the Witwatersrand, Johannesburg, South Africa; 193 Department of Physics, Stockholm University, Stockholm, Sweden; 194 The Oskar Klein Centre, Stockholm, Sweden; 195 Physics Department, Royal Institute of Technology, Stockholm, Sweden; 196 Departments of Physics and Astronomy and Chemistry, Stony Brook University, Stony Brook, NY USA; 197 Department of Physics and Astronomy, University of Sussex, Brighton, UK; 198 School of Physics, University of Sydney, Sydney, Australia; 199 Institute of Physics, Academia Sinica, Taipei, Taiwan; 200 Department of Physics, Technion: Israel Institute of Technology, Haifa, Israel; 201 Raymond and Beverly Sackler School of Physics and Astronomy, Tel Aviv University, Tel Aviv, Israel; 202 Department of Physics, Aristotle University of Thessaloniki, Thessaloníki, Greece; 203 International Center for Elementary Particle Physics and Department of Physics, The University of Tokyo, Tokyo, Japan; 204 Graduate School of Science and Technology, Tokyo Metropolitan University, Tokyo, Japan; 205 Department of Physics, Tokyo Institute of Technology, Tokyo, Japan; 206 Department of Physics, University of Toronto, Toronto, ON Canada; 207 TRIUMF, Vancouver, BC Canada; 208 Department of Physics and Astronomy, York University, Toronto, ON Canada; 209 Faculty of Pure and Applied Sciences,and Center for Integrated Research in Fundamental Science and Engineering, University of Tsukuba, Tsukuba, Japan; 210 Department of Physics and Astronomy, Tufts University, Medford, MA USA; 211 Centro de Investigaciones, Universidad Antonio Narino, Bogotá, Colombia; 212 Department of Physics and Astronomy, University of California Irvine, Irvine, CA USA; 213 INFN Gruppo Collegato di Udine, Sezione di Trieste, Udine, Italy; 214 ICTP, Trieste, Italy; 215 Dipartimento di Chimica Fisica e Ambiente, Università di Udine, Udine, Italy; 216 Department of Physics, University of Illinois, Urbana, IL USA; 217 Department of Physics and Astronomy, University of Uppsala, Uppsala, Sweden; 218 Instituto de Física Corpuscular (IFIC) and Departamento de Física Atómica, Molecular y Nuclear and Departamento de Ingeniería Electrónica and Instituto de Microelectrónica de Barcelona (IMB-CNM), University of Valencia and CSIC, Valencia, Spain; 219 Department of Physics, University of British Columbia, Vancouver, BC Canada; 220 Department of Physics and Astronomy, University of Victoria, Victoria, BC Canada; 221 Department of Physics, University of Warwick, Coventry, UK; 222 Waseda University, Tokyo, Japan; 223 Department of Particle Physics, The Weizmann Institute of Science, Rehovot, Israel; 224 Department of Physics, University of Wisconsin, Madison, WI USA; 225 Fakultät für Physik und Astronomie, Julius-Maximilians-Universität, Würzburg, Germany; 226 Fachbereich C Physik, Bergische Universität Wuppertal, Wuppertal, Germany; 227 Department of Physics, Yale University, New Haven, CT USA; 228 Yerevan Physics Institute, Yerevan, Armenia; 229 Centre de Calcul de l’Institut National de Physique Nucléaire et de Physique des Particules (IN2P3), Villeurbanne, France; 230 CERN, Geneva, Switzerland

## Abstract

Distributions of transverse momentum $p_T^{\ell \ell }$ and the related angular variable $\phi ^*_\eta$ of DrellΓÇôYan lepton pairs are measured in 20.3┬áfb$^{-1}$ of protonΓÇôproton collisions at $\sqrt{s}=8$┬áTeV with the ATLAS detector at the LHC. Measurements in electron-pair and muon-pair final states are corrected for detector effects and combined. Compared to previous measurements in protonΓÇôproton collisions at $\sqrt{s}=7$┬áTeV, these new measurements benefit from a larger data sample and improved control of systematic uncertainties. Measurements are performed in bins of lepton-pair mass above, around and below the *Z*-boson mass peak. The data are compared to predictions from perturbative and resummed QCD calculations. For values of $\phi ^*_\eta < 1$ the predictions from the Monte Carlo generator ResBos are generally consistent with the data within the theoretical uncertainties. However, at larger values of $\phi ^*_\eta$ this is not the case. Monte Carlo generators based on the parton-shower approach are unable to describe the data over the full range of $p_T^{\ell \ell }$ while the fixed-order prediction of Dynnlo falls below the data at high values of $p_T^{\ell \ell }$. ResBos and the parton-shower Monte Carlo generators provide a much better description of the evolution of the $\phi ^*_\eta$ and $p_T^{\ell \ell }$ distributions as a function of lepton-pair mass and rapidity than the basic shape of the data.

## Introduction

In high-energy hadronΓÇôhadron collisions the vector bosons *W* and $Z/\gamma ^*$ are produced via quarkΓÇôantiquark annihilation, and may be observed with very small backgrounds in their leptonic decay modes. The vector bosons may have non-zero momentum transverse to the beam direction $p_\mathrm {T}^{(W,Z)}$ due to the emission of quarks and gluons from the initial-state partons as well as to the intrinsic transverse momentum of the initial-state partons in the proton. Phenomenologically, the spectrum at low $p_\mathrm {T}^{(W,Z)}$ can be described using soft-gluon resummation [1] together with a non-perturbative contribution from the parton intrinsic transverse momentum. At high $p_\mathrm {T}^{(W,Z)}$ the spectrum may be described by fixed-order perturbative QCD predictions [2–4]. Parton-shower models [5, 6] may be used to compensate for missing higher-order corrections in the fixed-order QCD predictions.

Measurements of $p_\mathrm {T}^{(W,Z)}$ thus test several aspects of QCD. The correct modelling of $p_\mathrm {T}^{(W,Z)}$ is also important in many physics analyses at the LHC for which the production of *W* and/or *Z* bosons constitutes a background. Moreover, it is a crucial ingredient for a precise measurement of the *W*-boson mass, at both the LHC and the Tevatron. Measurements of the dependence of $p_\mathrm {T}^{(W,Z)}$ on the boson rapidity^1^ are sensitive to the gluon distribution function of the proton [7]. High-precision measurements at large values of $p_\mathrm {T}^{(W,Z)}$ could be sensitive to electroweak (EW) corrections [8].

DrellΓÇôYan events with final states including $e^+ e^-$ or $\mu ^+ \mu ^-$ (ΓÇÿDrellΓÇôYan lepton pairsΓÇÖ) allow the transverse momentum $p_\mathrm {T}^{\ell \ell }$ of $Z/\gamma ^*$ bosons to be measured with greater precision than is possible in the case of *W* bosons, because of the unobserved neutrino produced in *W* leptonic decays. Measurements of $p_\mathrm {T}^{\ell \ell }$ for lepton-pair masses, $m_{\ell \ell }$, around the *Z*-boson mass peak have been made by the CDF Collaboration [9] and the D0 Collaboration [10–12] at the Tevatron, and the ATLAS Collaboration [13, 14], the CMS Collaboration [15, 16] and the LHCb Collaboration [17–19] at the LHC. Measurements of $p_\mathrm {T}^{\ell \ell }$ require a precise understanding of the transverse momentum $p_{\text {T}}$ calibration and resolution of the final-state leptons. Associated systematic uncertainties affect the resolution in $p_\mathrm {T}^{\ell \ell }$ and limit the ultimate precision of the measurements, particularly in the low-$p_\mathrm {T}^{\ell \ell }$ domain. To minimise the impact of these uncertainties, the $\phi _\eta ^*$ observable was introduced [20] as an alternative probe of $p_\mathrm {T}^{\ell \ell }$. It is defined as

1 $$\begin{aligned} \phi _\eta ^* = \tan \left( \frac{\pi -\Delta \phi }{2}\right) \cdot \sin (\theta ^*_\eta ), \end{aligned}$$

where $\Delta \phi$ is the azimuthal angle in radians between the two leptons. The angle $\theta _\eta ^*$ is a measure of the scattering angle of the leptons with respect to the proton beam direction in the rest frame of the dilepton system and is defined by $\cos (\theta _\eta ^*) = \tanh [(\eta ^- - \eta ^+)/2]$, where $\eta ^-$ and $\eta ^+$ are the pseudorapidities of the negatively and positively charged lepton, respectively [20]. Therefore, $\phi ^*_{\eta }$ depends exclusively on the directions of the two leptons, which are more precisely measured than their momenta. Measurements of $\phi ^*_{\eta }$ for $m_{\ell \ell }$ around the *Z*-boson mass peak were first made by the D0 Collaboration [21] at the Tevatron and subsequently by the ATLAS Collaboration [22] for $\sqrt{s}\!=\!7 ~{\mathrm {TeV}}$ and the LHCb Collaboration for $\sqrt{s}\!=\!7 ~{\mathrm {TeV}}$ [17, 18] and┬á$8 ~{\mathrm {TeV}}$ [19] at the LHC. First measurements of $\phi ^*_\eta$ for ranges of $m_{\ell \ell }$ above and below the *Z*-boson mass peak were recently presented by the D0 Collaboration [23].

Measurements are presented here of $\phi ^*_{\eta }$ and $p_\mathrm {T}^{\ell \ell }$ for DrellΓÇôYan lepton-pair events using the complete $\sqrt{s}=8 ~{\mathrm {TeV}}$ data set of the ATLAS experiment at the LHC, corresponding to an integrated luminosity of 20.3┬á$\mathrm{fb}^{-1}$. The data are corrected for detector effects. The measurements are presented for $e^+ e^-$ and $\mu ^+ \mu ^-$ final states, in bins of $m_{\ell \ell }$, above and below, as well as at the *Z*-boson mass peak, and in bins of the $Z/\gamma ^*$-boson rapidity $|y_{\ell \ell }|$. In addition, integrated fiducial cross sections are provided for six regions of $m_{\ell \ell }$.

The ATLAS experiment is briefly described in Sect.┬á2. A general overview of the measurement methods is given in Sect.┬á3, which has specific sections on the event simulation, event reconstruction, event selection, background estimation, corrections for detector effects, and the evaluation of the systematic uncertainties. The combination of the measurements in the $e^+ e^-$ and $\mu ^+ \mu ^-$ final states is described in Sect.┬á4. The corrected differential cross sections are compared to various theoretical predictions in Sect.┬á5. A short summary and conclusion are given in Sect.┬á6. The values of the normalised differential cross sections $(1/\sigma )\, {\mathrm {d}}\sigma / {\mathrm {d}}\phi ^*_{\eta }$ and $(1/\sigma )\, {\mathrm {d}}\sigma / {\mathrm {d}}p_\mathrm {T}^{\ell \ell }$ are given in tables in the Appendix for each region of $m_{\ell \ell }$ and $|y_{\ell \ell }|$ considered.

## The ATLAS detector

The ATLAS detector [24] at the LHC covers nearly the entire solid angle around the collision point. It consists of an inner tracking detector (ID) surrounded by a thin superconducting solenoid, electromagnetic and hadronic calorimeters, and a muon spectrometer (MS) incorporating three large superconducting toroid magnets. The ID is immersed in a 2 T axial magnetic field and provides charged-particle tracking in the range $|\eta | < 2.5$. A high-granularity silicon pixel detector typically provides three measurements per track, and is followed by a silicon microstrip tracker, which usually provides four three-dimensional measurement points per track. These silicon detectors are complemented by a transition radiation tracker, which enables radially extended track reconstruction up to $|\eta | = 2.0$. The transition radiation tracker also provides electron identification information based on the fraction of hits (typically 30 in total) above a higher energy-deposit threshold corresponding to transition radiation.

The calorimeter system covers the pseudorapidity range $|\eta | < 4.9$. Within the region $|\eta |< 3.2$, electromagnetic calorimetry is provided by barrel and endcap high-granularity lead/liquid-argon (LAr) electromagnetic calorimeters, with an additional thin LAr presampler covering $|\eta | < 1.8$, to correct for energy loss in material upstream of the calorimeters. Hadronic calorimetry is provided by the steel/scintillator-tile calorimeter, segmented into three barrel structures within $|\eta | < 1.7$, and two copper/LAr hadronic endcap calorimeters. The solid angle coverage is completed with forward copper/LAr and tungsten/LAr calorimeter modules optimised for electromagnetic and hadronic measurements, respectively.

The MS comprises separate trigger and precision tracking chambers measuring the deflection of muons in a magnetic field generated by superconducting air-core toroids. The precision chamber system covers the region $|\eta | < 2.7$ with three layers of monitored drift tubes, complemented by cathode-strip chambers in the forward region, where the background is highest. The muon trigger system covers the range $|\eta | < 2.4$ with resistive-plate chambers in the barrel, and thin-gap chambers in the endcap regions.

**Table 1:** Synopsis of the $\phi ^*_{\eta }$ and $p_\mathrm {T}^{\ell \ell }$ measurements, and of the fiducial region definitions used. Full details including the definition of the Born, bare and dressed particle levels are provided in the text. Unless otherwise stated criteria apply to both $\phi ^*_{\eta }$ and $p_\mathrm {T}^{\ell \ell }$ measurements

*Particle-level definitions* (*treatment of final-state photon radiation*)	
Electron pairs	Dressed; Born
Muon pairs	Bare; dressed; Born
Combined	Born
*Fiducial region*	
Leptons	$p_T > 20 ~{\mathrm {GeV}}$ and $|\eta |< 2.4$
Lepton pairs	$|y_{\ell \ell }|< 2.4$
*Mass and rapidity regions*	
$46 ~{\mathrm {GeV}}<m_{\ell \ell }<66 ~{\mathrm {GeV}}$	$|y_{\ell \ell }|<0.8$; $0.8<|y_{\ell \ell }|<1.6$; $1.6<|y_{\ell \ell }|<2.4$ ($\phi ^*_{\eta }$ measurements only)
	$$|y_{\ell \ell }|<2.4$$
$66 ~{\mathrm {GeV}}<m_{\ell \ell }<116 ~{\mathrm {GeV}}$	$|y_{\ell \ell }|<0.4$; $0.4<|y_{\ell \ell }|<0.8$; $0.8<|y_{\ell \ell }|<1.2$; $1.2<|y_{\ell \ell }|<1.6$; $1.6<|y_{\ell \ell }|<2.0$; $2.0<|y_{\ell \ell }|<2.4$; $|y_{\ell \ell }|<2.4$
$116 ~{\mathrm {GeV}}<m_{\ell \ell }<150 ~{\mathrm {GeV}}$	$|y_{\ell \ell }|<0.8$; $0.8<|y_{\ell \ell }|<1.6$; $1.6<|y_{\ell \ell }|<2.4$ ($\phi ^*_{\eta }$ measurements only)
	$$|y_{\ell \ell }|<2.4$$
*Very-low mass regions*	
$$\left. \begin{array}{l} 12 ~{\mathrm {GeV}}<m_{\ell \ell }<20 ~{\mathrm {GeV}}\\ 20 ~{\mathrm {GeV}}<m_{\ell \ell }<30 ~{\mathrm {GeV}}\\ 30 ~{\mathrm {GeV}}<m_{\ell \ell }<46 ~{\mathrm {GeV}}\\ \end{array} \right\} $$	$|y_{\ell \ell }|<2.4$, $p_\mathrm {T}^{\ell \ell }>45 ~{\mathrm {GeV}}$, $p_\mathrm {T}^{\ell \ell }$ measurements only

A three-level trigger system is used to select interesting events [25]. The Level-1 trigger is implemented in hardware and uses a subset of detector information to reduce the event rate to a design value of at most 75┬ákHz. This is followed by two software-based trigger levels which together reduce the event rate to about 400 Hz.

## Analysis methods

This section describes the particle-level measurements presented in this paper (Sect.┬á3.1), the simulation of signal and background Monte Carlo (MC) samples (Sect.┬á3.2), the event reconstruction and selection criteria (Sect.┬á3.3), the estimation of backgrounds (Sect.┬á3.4), corrections to the distributions of $\phi ^*_{\eta }$ and $p_\mathrm {T}^{\ell \ell }$ for detector effects and final-state radiation (Sect.┬á3.5), and the estimation of systematic uncertainties (Sect.┬á3.6).

### Description of the particle-level measurements

DrellΓÇôYan signal MC simulation is used to correct the background-subtracted data for detector resolution and inefficiency. Three different ΓÇÿparticle-levelΓÇÖ definitions are employed, which differ in their treatment of final-state photon radiation (FSR). The Born and bare levels are defined from the lepton kinematics before and after FSR, respectively. The dressed level is defined by combining the bare four-momentum of each lepton with that of photons radiated within a cone defined by $\Delta R = 0.1$ (See footnote 1) around the lepton. The muon-pair data are corrected to the bare, dressed, and Born levels. The electron-pair data are corrected to the dressed and Born levels. The two lepton-pair channels are combined at the Born level. The bare and dressed particle-level definitions reduce the dependence on the MC FSR model used to correct the data, which results (particularly for events with $m_{\ell \ell }$ below the *Z*-boson mass peak) in a lower systematic uncertainty. Corrections to a common particle-level definition (Born level) for the combination of the two channels allow comparisons to calculations that do not account for the effects of FSR, albeit at the cost of an increased systematic uncertainty on the corrected data.

The data are corrected to the particle level within fiducial regions in lepton $p_{\text {T}}$ and $|\eta |$, and in lepton-pair $m_{\ell \ell }$ and $|y_{\ell \ell }|$ that correspond closely to the selection criteria applied to the data. The fiducial regions common to the measurements of $\phi ^*_{\eta }$ and $p_\mathrm {T}^{\ell \ell }$ are described first. The two leptons are required to have $p_{\text {T}} {} > 20 ~{\mathrm {GeV}}$ and $|\eta |<2.4$. Measurements of the normalised differential cross sections $(1/\sigma )\, {\mathrm {d}}\sigma / {\mathrm {d}}\phi ^*_{\eta }$ and $(1/\sigma )\, {\mathrm {d}}\sigma / {\mathrm {d}}p_\mathrm {T}^{\ell \ell }$, and of the absolute differential cross section ${\mathrm {d}}\sigma / {\mathrm {d}}p_\mathrm {T}^{\ell \ell }$, are made in three $m_{\ell \ell }$ regions within $46 ~{\mathrm {GeV}}< m_{\ell \ell }< 150 ~{\mathrm {GeV}}$ for $|y_{\ell \ell }|<2.4$. In the mass region $66 ~{\mathrm {GeV}}< m_{\ell \ell }< 116 ~{\mathrm {GeV}}$, measurements are made in six equally sized regions of $|y_{\ell \ell }|$. The distributions of $(1/\sigma )\, {\mathrm {d}}\sigma / {\mathrm {d}}\phi ^*_{\eta }$ and $(1/\sigma )\, {\mathrm {d}}\sigma / {\mathrm {d}}p_\mathrm {T}^{\ell \ell }$ are individually normalised in each region of $|y_{\ell \ell }|$. Measurements of $(1/\sigma )\, {\mathrm {d}}\sigma / {\mathrm {d}}\phi ^*_{\eta }$ in the regions of $m_{\ell \ell }$ above and below the *Z*-boson mass peak, $46 ~{\mathrm {GeV}}< m_{\ell \ell }< 66 ~{\mathrm {GeV}}$ and $116 ~{\mathrm {GeV}}< m_{\ell \ell }< 150 ~{\mathrm {GeV}}$, are made in three equally-sized regions of $|y_{\ell \ell }|$. For $p_\mathrm {T}^{\ell \ell }>45 ~{\mathrm {GeV}}$, measurements of $p_\mathrm {T}^{\ell \ell }$ are made in three additional mass regions below $46 ~{\mathrm {GeV}}$.

A synopsis of the $\phi ^*_{\eta }$ and $p_\mathrm {T}^{\ell \ell }$ measurements, and of the fiducial-region definitions used is given in Table┬á1.

### Event simulation

MC simulation is used to estimate backgrounds and to correct the data for detector resolution and inefficiencies, as well as for the effects of FSR.

Three generators are used to produce samples of DrellΓÇôYan lepton-pair signal events. The first is Powheg [26, 27] which uses the CT10 set of parton distribution functions (PDFs) [28] and is interfaced to Pythia 8.170 [6, 29] with the AU2 set of tuned parameters (tune) [30] to simulate the parton shower, hadronisation and underlying event, and to Photos [31] to simulate FSR. This is referred to as Powheg+Pythia in the text. The second is Powheg interfaced to Herwig 6.520.2 [5] for the parton shower and hadronisation, Jimmy [32] for the underlying event, and Photos for FSR (referred to as Powheg+Herwig). The Sherpa 1.4.1 [33] generator is also used, which has its own implementation of the parton shower, hadronisation, underlying event and FSR, and which again uses the CT10 PDF set. Differences between the results obtained using these three generators are used to estimate systematic uncertainties related to the choice of generator.

Background events from the process $Z\rightarrow \tau \tau$ are produced using Alpgen [34] interfaced to Herwig to simulate the parton shower and Jimmy to simulate the underlying event. Single *W*-boson decays to electrons, muons and $\tau$ leptons are produced with Sherpa, and the diboson processes *WW*, *WZ* and *ZZ* are produced with Herwig. The $t\bar{t}$ process is simulated with MC@NLO [35] interfaced to Jimmy, as is the single-top process in the *s*-channel and *Wt*-channel. The *t*-channel is generated with AcerMC [36] interfaced to Pythia. Exclusive $\gamma \gamma \rightarrow \ell \ell$ production is generated using the Herwig++ 2.6.3 generator [37]. Photon-induced single-dissociative dilepton production, is simulated using Lpair 4.0 [38] with the Brasse [39] and SuriΓÇôYennie [40] structure functions for proton dissociation. For double-dissociative $\gamma \gamma \rightarrow \ell \ell$ reactions, Pythia 8.175 [29] is used with the MRST2004QED [41] PDFs.

The effect of multiple interactions per bunch crossing (pile-up) is simulated by overlaying MC-generated minimum bias events [42]. The simulated event samples are reweighted to describe the distribution of the number of pile-up events in the data. The Geant4 [43] program is used to simulate the passage of particles through the ATLAS detector. Differences in reconstruction, trigger, identification and isolation efficiencies between MC simulation and data are evaluated using a tag-and-probe method [44, 45] and are corrected for by reweighting the MC simulated events. Corrections are also applied to MC events for the description of the lepton energy and momentum scales and resolution, which are determined from fits to the observed $Z$ -boson line shapes in data and MC simulation [45, 46]. The MC simulation is also reweighted to better describe the distribution of the longitudinal position of the primary *pp* collision vertex [47] in data.

Three additional samples of DrellΓÇôYan lepton-pair signal events are produced without detector simulation, for the purpose of comparison with the corrected data in Sect.┬á5. The MC generators used are ResBos, Dynnlo, and Powheg+Pythia (AZNLO tune).

ResBos [48] simulates vector-boson production and decay, but does not include a description of the hadronic activity in the event nor of FSR. Initial-state QCD corrections to *Z*-boson production are simulated at approximately next-to-next-to-leading-order (NNLO) accuracy using approximate NNLO (i.e. $\mathcal {O}(\alpha _s^2)$) Wilson coefficient functions [49].^2^ The contributions from $\gamma ^{*}$ and from $Z/\gamma ^{*}$ interference are simulated at next-to-leading-order (NLO) accuracy (i.e. $\mathcal {O}(\alpha _s)$). ResBos uses a resummed treatment of soft-gluon emissions at next-to-next-to-leading-logarithm (NNLL) accuracy. It uses the GNW parameterisation [49, 50] of non-perturbative effects at small $p_\mathrm {T}^{\ell \ell }$, as optimised using the D0 $\phi ^*_{\eta }$ measurements in Ref. [21]. The CT14 NNLO PDF sets [51] are used and the corresponding 90┬á% confidence-level PDF uncertainties are evaluated and rescaled to 68┬á% confidence level. The choices^3^ of central values and range of systematic uncertainty variations for QCD scales and the non-perturbative parameter $a_Z$ are made following Ref. [49]. These differ from the choices made for the ATLAS $7 ~{\mathrm {TeV}}$ $p_\mathrm {T}^{\ell \ell }$ and $\phi ^*_{\eta }$ papers [14, 22].

Dynnlo1.3 [4] simulates initial-state QCD corrections to NNLO accuracy. The CT10 NNLO PDF sets are used. The Dynnlo calculation is performed in the $G_{\mu }$ electroweak parameter scheme [52]. Additional NLO electroweak virtual corrections^4^ are provided by the authors of Ref. [53]. Dynnlo does not account for the effects of multiple soft-gluon emission and therefore is not able to make accurate predictions at low $\phi ^*_{\eta }$ and $p_\mathrm {T}^{\ell \ell }$.

An additional Powheg+Pythia sample is produced which uses the AZNLO tune [14]. This tune includes the ATLAS $7 ~{\mathrm {TeV}}$ $\phi ^*_{\eta }$ and $p_\mathrm {T}^{\ell \ell }$ results in a mass region around the $Z$ peak. The sample uses Pythia version 8.175 and the CTEQ6L1 PDF set [54] for the parton shower, while CT10 is used for the Powheg calculation.

### Event reconstruction and selection

The measurements are performed using protonΓÇôproton collision data recorded at $\sqrt{s} = 8\,~{\mathrm {TeV}}$. The data were collected between April and December 2012 and correspond to an integrated luminosity of $20.3\,\mathrm{fb}^{-1}$. Selected events are required to be in a data-taking period in which there were stable beams and the detector was fully operational.

**Table 2:** The number of events in data satisfying the selection criteria in the electron-pair channel for six different regions of $m_{\ell \ell }$ and the estimated contribution to this value from the various background sources considered. The uncertainties quoted on the background samples include contributions from statistical and systematic sources

$m_{\ell \ell }$ [GeV]	Data	Total Bkg	Multi-jet	$t\bar{t}$, single top	$Z\rightarrow \tau \tau$	$W\rightarrow \ell \nu$	*WW*┬á/┬á*WZ*┬á/┬á*ZZ*	$\gamma \gamma \rightarrow \ell \ell$
12ΓÇô20	17┬á729	2┬á220 $\pm$ 470	1┬á370 $\pm$ 460	509 $\pm$ 27	7 $\pm$ 1	215 $\pm$ 44	81 $\pm$ 7	41 $\pm$ 16
20ΓÇô30	13┬á322	1┬á860 $\pm$ 210	600 $\pm$ 200	873 $\pm$ 46	33 $\pm$ 3	144 $\pm$ 36	158 $\pm$ 11	54 $\pm$ 21
30ΓÇô46	14┬á798	3┬á290 $\pm$ 260	570 $\pm$ 230	1┬á920 $\pm$ 100	228 $\pm$ 23	192 $\pm$ 48	314 $\pm$ 25	75 $\pm$ 30
46ΓÇô66	201┬á613	25┬á600 $\pm$ 3┬á900	6┬á200 $\pm$ 3┬á400	3┬á990 $\pm$ 210	9┬á360 $\pm$ 940	670 $\pm$ 170	1┬á060 $\pm$ 88	4┬á300 $\pm$ 1┬á700
66ΓÇô116	6┬á671┬á873	59┬á400 $\pm$ 9┬á500	23┬á500 $\pm$ 9┬á200	13┬á040 $\pm$ 680	3┬á560 $\pm$ 360	3┬á860 $\pm$ 930	10┬á450 $\pm$ 320	5┬á000 $\pm$ 2┬á000
116ΓÇô150	77┬á919	8┬á280 $\pm$ 170	910 $\pm$ 170	4┬á590 $\pm$ 240	82 $\pm$ 8	530 $\pm$ 130	1┬á097 $\pm$ 90	1┬á070 $\pm$ 430

**Table 3:** The number of events in data satisfying the selection criteria in the muon-pair channel for six different regions of $m_{\ell \ell }$ and the estimated contribution to this value from the various background sources considered. The uncertainties quoted on the background samples include contributions from statistical and systematic sources

$m_{\ell \ell }$ [GeV]	Data	Total Bkg	Multi-jet	$t\bar{t}$, single top	$Z\rightarrow \tau \tau$	$W\rightarrow \ell \nu$	*WW*┬á/┬á*WZ*┬á/┬á*ZZ*	$\gamma \gamma \rightarrow \ell \ell$
12ΓÇô20	25┬á297	1┬á220 $\pm$ 180	440 $\pm$ 170	605 $\pm$ 32	1 $\pm$ 0	9 $\pm$ 2	107 $\pm$ 10	64 $\pm$ 26
20ΓÇô30	19┬á485	2┬á100 $\pm$ 250	590 $\pm$ 240	1┬á156 $\pm$ 61	20 $\pm$ 2	8 $\pm$ 2	241 $\pm$ 19	84 $\pm$ 33
30ΓÇô46	20┬á731	3┬á980 $\pm$ 330	730 $\pm$ 290	2┬á540 $\pm$ 130	156 $\pm$ 16	12 $\pm$ 3	429 $\pm$ 36	114 $\pm$ 45
46ΓÇô66	318┬á117	30┬á900 $\pm$ 4┬á100	7┬á400 $\pm$ 3┬á000	5┬á370 $\pm$ 280	9┬á940 $\pm$ 990	174 $\pm$ 35	1┬á460 $\pm$ 120	6┬á600 $\pm$ 2┬á600
66ΓÇô116	9┬á084┬á639	46┬á500 $\pm$ 4┬á200	7┬á400 $\pm$ 3┬á000	13┬á730 $\pm$ 720	4┬á150 $\pm$ 420	870 $\pm$ 170	13┬á640 $\pm$ 420	6┬á700 $\pm$ 2┬á700
116ΓÇô150	100┬á697	9┬á960 $\pm$ 520	1┬á270 $\pm$ 520	5┬á790 $\pm$ 300	58 $\pm$ 6	153 $\pm$ 38	1┬á310 $\pm$ 110	1┬á380 $\pm$ 550

For measurements of $\phi ^*_{\eta }$, candidate electron-pair events were obtained using a dielectron trigger, whilst for measurements of $p_\mathrm {T}^{\ell \ell }$, a combination of a single-electron trigger (to select events with the leading reconstructed electron $p_{\text {T}} > 60 ~{\mathrm {GeV}}$ and the sub-leading electron $p_{\text {T}} > 25 ~{\mathrm {GeV}}$) and a dielectron trigger (to select all other events) was used. The motivation for using a slightly different trigger selection for measurements of the $p_\mathrm {T}^{\ell \ell }$ observable is to obtain a higher efficiency for electron pairs with $\Delta R< 0.35$, which is relevant to maintain a high acceptance for $m_{\ell \ell }<46 ~{\mathrm {GeV}}$. Electron candidates are reconstructed from clusters of energy in the electromagnetic calorimeter matched to ID tracks [55]. They are required to have $p_{\text {T}} > 20 ~{\mathrm {GeV}}$ and $|\eta | < 2.4$, but excluding the transition regions between the barrel and the endcap electromagnetic calorimeters, $1.37< |\eta | < 1.52$. The electron candidates must satisfy a set of ΓÇÿmediumΓÇÖ selection criteria [55] that have been reoptimised for the larger number of protonΓÇôproton collisions per beam crossing observed in the 2012 data. Events are required to contain exactly two electron candidates. Except for the $m_{\ell \ell }$ region around the *Z*-boson mass peak, the electron candidates are required to be isolated, satisfying $I_e$┬á<┬á0.2, where $I_e$ is the scalar sum of the $p_{\text {T}}$ of tracks with $\Delta R < 0.4$ around the electron track divided by the $p_{\text {T}}$ of the electron. For measurements of $p_\mathrm {T}^{\ell \ell }$, this requirement is not applied when the two electrons are separated by $\Delta R < 0.5$. For measurements of $p_\mathrm {T}^{\ell \ell }$ the two electron candidates must satisfy $\Delta R > 0.15$.

**Fig. 1:**
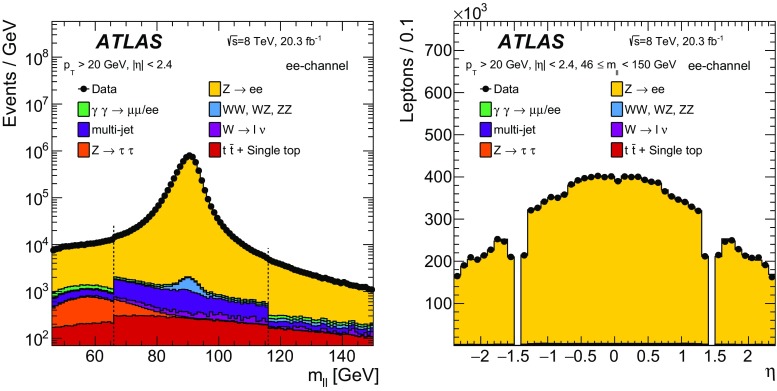
The distribution of events passing the selection requirements in the electron-pair channel as a function of dilepton invariant mass $m_{\ell \ell }$ (*left*) and electron pseudorapidity $\eta$ (*right*). Events are shown for the $m_{\ell \ell }$ range 46 to $150 ~{\mathrm {GeV}}$. The MC signal sample (*yellow*) is simulated using Powheg+Pythia. The statistical uncertainties on the data points are smaller than the size of the markers and the systematic uncertainties are not plotted. The prediction is normalised to the integral of the data. *The vertical dashed lines* on the *left-hand plot* at $m_{\ell \ell }$ values of 66 and $116 ~{\mathrm {GeV}}$ indicate the boundaries between the three principal $m_{\ell \ell }$ regions employed in the analysis. The small discontinuities in the $m_{\ell \ell }$ distribution at 66 and $116 ~{\mathrm {GeV}}$ are due to the absence of the isolation requirement around the $Z$ -boson mass peak

**Fig. 2:**
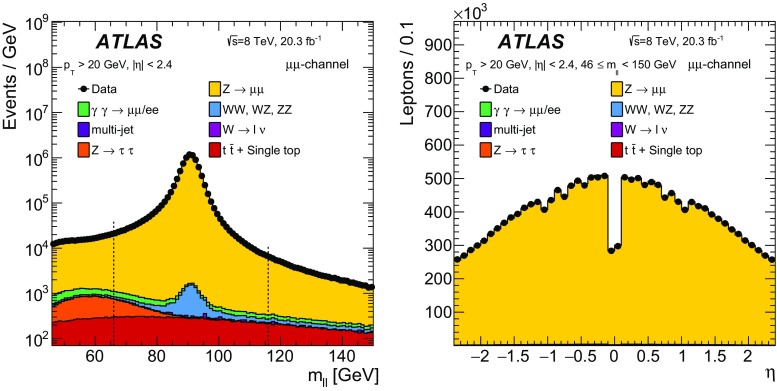
The distribution of events passing the selection requirements in the muon-pair channel as a function of dilepton invariant mass $m_{\ell \ell }$ (*left*) and muon pseudorapidity $\eta$ (*right*). Events are shown for the $m_{\ell \ell }$ range 46 to $150 ~{\mathrm {GeV}}$. The MC signal sample (*yellow*) is simulated using Powheg+Pythia. The statistical uncertainties on the data points are smaller than the size of the markers and the systematic uncertainties are not plotted. The prediction is normalised to the integral of the data. *The vertical dashed lines* on the *left hand plot* at $m_{\ell \ell }$ values of 66 and $116 ~{\mathrm {GeV}}$ indicate the boundaries between the three principal $m_{\ell \ell }$ regions employed in the analysis

Candidate muon-pair events are retained for further analysis using a combination of a single-muon trigger (for $p_{\text {T}} > 25$┬áGeV) and a dimuon trigger (for $20< p_{\text {T}} < 25$┬áGeV). Muon candidates are reconstructed by combining tracks reconstructed in both the inner detector and the MS [45]. They are required to have $p_{\text {T}} > 20 ~{\mathrm {GeV}}$ and $|\eta | < 2.4$. In order to suppress backgrounds, track-quality requirements are imposed for muon identification, and longitudinal and transverse impact-parameter requirements ensure that the muon candidates originate from a common primary protonΓÇôproton interaction vertex. The muon candidates are also required to be isolated, satisfying $I_\mu$┬á<┬á0.1, where $I_\mu$ is the scalar sum of the $p_{\text {T}}$ of tracks within a cone of size $\Delta R = 0.2$ around the muon divided by the $p_{\text {T}}$ of the muon. Events are required to contain exactly two muon candidates of opposite charge satisfying the above criteria.

Precise knowledge of the lepton directions is particularly important for the $\phi ^*_{\eta }$ measurements. These are determined for electron candidates by the track direction in the ID, and for muon candidates from a combination of the track direction in the ID and in the MS.

Tables┬á2 and┬á3 show the number of events satisfying the above selection criteria in the electron-pair and muon-pair channels, respectively, for six regions of $m_{\ell \ell }$. Also given is the estimated contribution to the data from the various background sources considered (described in Sect.┬á3.4).

Figure┬á1 shows the distributions of $m_{\ell \ell }$ and $\eta$ for electron-pair events passing the selection requirements described above. Figure┬á2 shows the equivalent distributions for the dimuon channel. The MC signal sample is simulated using Powheg+Pythia. The predictions from the model are in qualitative agreement with the data.

**Fig. 3:**
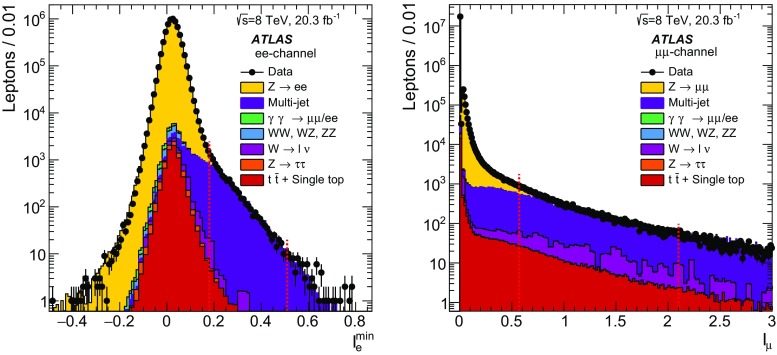
*Left* The distribution of the smallest of the isolation variables of the two electrons $I_e^{\text {min}}$. *Right* The distribution of the muon isolation variable $I_\mu$. The data for $66 ~{\mathrm {GeV}}< m_{\ell \ell }< 116 ~{\mathrm {GeV}}$ are compared to the sum of the estimated multi-jet background and all other processes, which are estimated from MC simulation. *The red dashed lines* indicate the range over which the fit is performed

### Estimation of backgrounds

The number and properties of the background events arising from multi-jet processes are estimated using a data-driven technique. A background-dominated sample is selected using a modified version of the signal-selection criteria. In the electron-pair channel, both electrons are required to satisfy the ΓÇÿlooseΓÇÖ identification criteria [55], but not the ΓÇÿmediumΓÇÖ criteria, and are also required to have the same charge. For the muon-pair channel, two samples of lepton pairs are used: the light-flavour background is estimated by requiring a pair of muons with the same charge, whilst the heavy-flavour background is estimated by requiring one electron and one muon with opposite charge. The electron is required to be identified as ΓÇÿlooseΓÇÖ and the electron isolation cut is inverted. It is assumed that in all other variables the shape of the distribution of the multi-jet events is the same in both the signal- and background-dominated samples.

The normalisation of the multi-jet background is determined by performing a $\chi ^2$ minimisation in a variable that discriminates between the signal and multi-jet background. The contribution from all sources other than the multi-jet background is taken from MC simulation. Two independent fits are performed, using lepton isolation and $m_{\ell \ell }$ as discriminating variables. The signal event-selection criteria are applied, except that the selection criteria on the isolation variables are removed for the fit that uses lepton isolation. In the muon-pair final state, the fit using isolation is performed using the values of $I_\mu$. In the electron-pair final state, the isolation variable $I_e^*$ is defined as the scalar sum of the $E_\mathrm {T}$ of energy deposits in the calorimeter within a cone of size $\Delta R = 0.2$ around the electron cluster divided by the $p_{\text {T}}$ of the electron. The $E_\mathrm {T}$ sum excludes cells assigned to the electron cluster and can be negative due to cell noise and negative signal contribution from pile-up in neighbouring bunches [56]. The fit is performed using the quantity $I_e^{\text {min}}$, where $I_e^{\text {min}}$ is the smaller of the $I_e^*$ values of the two electrons in an event. Example results of fits to the isolation variables for the electron- and muon-pair channels are shown in Fig.┬á3 for the $m_{\ell \ell }$ region around the $Z$ -boson mass peak. The difference in the results of the fits to isolation and $m_{\ell \ell }$ is taken as the systematic uncertainty on the normalisation of the multi-jet background. As a cross-check the procedure is repeated in bins of $|y_{\ell \ell }|$ and gives results consistent with the fit performed inclusively in $|y_{\ell \ell }|$.

The backgrounds from all sources other than multi-jet processes are estimated using the MC samples detailed in Sect.┬á3.2. These estimates are cross-checked by comparing MC simulation to data in control regions, selected using criteria that increase the fraction of background. The $Z \rightarrow \tau \tau$ and $t\bar{t}$ backgrounds are enhanced by requiring exactly one electron and one muon candidate per event according to the criteria described in Sect.┬á3.3. The MC simulation is found to be consistent with the data within the assigned uncertainties on the cross sections (see Sect.┬á3.6). In addition, a subset of these events is studied in which two jets with $p_{\text {T}} {} > 25 ~{\mathrm {GeV}}$ are identified, which significantly enhances the contribution from the $t\bar{t}$ background. Again, the MC simulation is consistent with the data within the assigned uncertainties.

Around the $Z$ -boson mass peak and at low values of $\phi ^*_{\eta }$ and $p_\mathrm {T}^{\ell \ell }$, the background is dominated by multi-jet and $\gamma \gamma \rightarrow \ell \ell$ processes which together amount to less than 1┬á% of the selected electron-pair or muon-pair event sample. At high $\phi ^*_{\eta }$ and $p_\mathrm {T}^{\ell \ell }$, $t\bar{t}$ and diboson processes dominate and constitute a few percent of the selected data. In the regions of $m_{\ell \ell }$ below the *Z*-boson mass peak, $t\bar{t}$ continues to be a dominant background at larger values of $\phi ^*_{\eta }$ and $p_\mathrm {T}^{\ell \ell }$ (forming up to 20┬á% of the selected data), whilst at lower values of $\phi ^*_{\eta }$ and $p_\mathrm {T}^{\ell \ell }$ the dominant contribution is from $\gamma \gamma \rightarrow \ell \ell$ processes with other contributions from $Z \rightarrow \tau \tau$ and multi-jet processes (totalling between 10 and 20┬á% of the selected data). The fraction of $t\bar{t}$ background in the $m_{\ell \ell }$ regions below $46 ~{\mathrm {GeV}}$ is enhanced by the requirement that $p_\mathrm {T}^{\ell \ell }$ be greater than $45 ~{\mathrm {GeV}}$. In the region of $m_{\ell \ell }$ above the *Z*-boson mass peak, the $t\bar{t}$ background forms more than 30┬á% of the selected data at higher values of $\phi ^*_{\eta }$ and $p_\mathrm {T}^{\ell \ell }$. The total background is smaller at low values (approximately 10┬á% of the selected data) with the dominant contribution again coming from $\gamma \gamma \rightarrow \ell \ell$ processes.

### Corrections for detector effects and FSR

After the estimated total background is subtracted from the data, DrellΓÇôYan signal MC simulation is used to correct to the particle level, accounting for detector resolution and inefficiencies and the effects of FSR.

Since the experimental resolution in $\phi ^*_{\eta }$ is smaller than the chosen bin widths, the fractions of accepted events that fall within the same bin in $\phi ^*_{\eta }$ at the particle level and reconstructed detector level in the MC simulation are high, having typical values of around 90┬á%. Therefore, simple bin-by-bin corrections of the $\phi ^*_{\eta }$ distributions are sufficient. A single iteration is performed by reweighting the signal MC events at particle level to the corrected data and rederiving the correction factors. The correction factors are estimated using an average over all available signal MC samples (as described in Sect.┬á3.2).

The detector resolution has a larger effect in the measurement of $p_\mathrm {T}^{\ell \ell }$. An iterative Bayesian unfolding method [57–59] with seven iterations is used to correct the $p_\mathrm {T}^{\ell \ell }$ distribution to particle level. The response matrix, which connects the $p_\mathrm {T}^{\ell \ell }$ distribution at reconstruction and particle levels is estimated using the Powheg+Pythia signal MC sample.

**Fig. 4:**
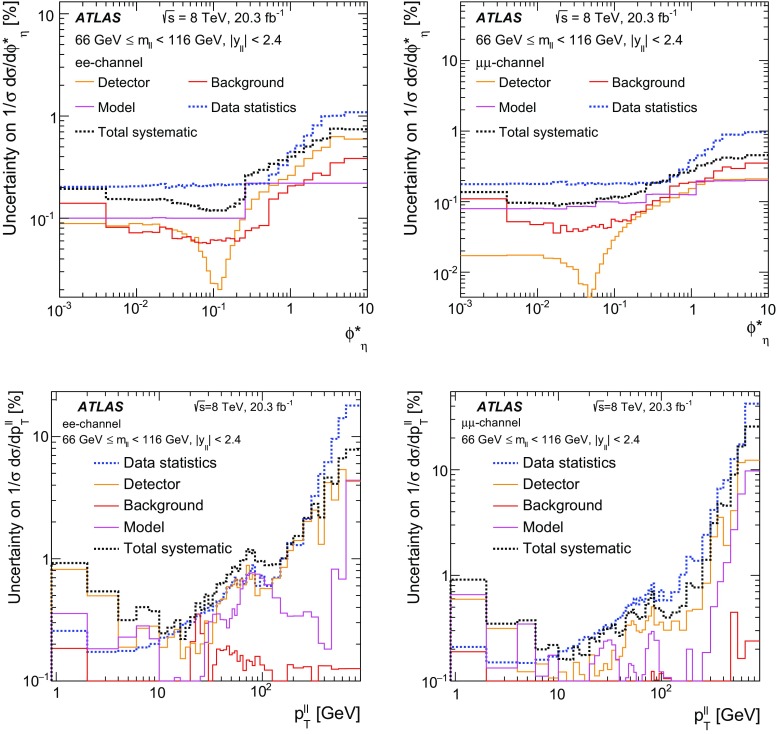
Uncertainty from various sources on $(1/\sigma )\, {\mathrm {d}}\sigma / {\mathrm {d}}\phi ^*_{\eta }$ (*top*) and $(1/\sigma )\, {\mathrm {d}}\sigma / {\mathrm {d}}p_\mathrm {T}^{\ell \ell }$ (*bottom*) for events with $66 ~{\mathrm {GeV}}< m_{\ell \ell }< 116 ~{\mathrm {GeV}}$ and $|y_{\ell \ell }|<2.4$. *Left* Electron-pair channel at dressed level. *Right* Muon-pair channel at bare level

### Systematic uncertainties

In this section the principal sources of uncertainty on the measurements are discussed, as well as the degree to which these uncertainties are correlated (between bins in $\phi ^*_{\eta }$ or $p_\mathrm {T}^{\ell \ell }$, or between the electron-pair and muon-pair channels) when combining the electron-pair and muon-pair results and in quoting the final results. Figure┬á4 provides a summary of the uncertainties arising from data statistics, mis-modelling of the detector, background processes, and of the MC signal samples used to correct the data. These are given for both the electron (dressed level) and muon (bare level) channels as a function of $\phi ^*_{\eta }$ and $p_\mathrm {T}^{\ell \ell }$ for events with $66 ~{\mathrm {GeV}}< m_{\ell \ell }< 116 ~{\mathrm {GeV}}$ and $|y_{\ell \ell }|<2.4$.

The statistical uncertainties on the data, and on the MC samples used to correct the data, are considered as uncorrelated between bins and between channels. In most kinematic regions the statistical uncertainty on the data is larger than the total systematic uncertainty in both $\phi ^*_{\eta }$ and $p_\mathrm {T}^{\ell \ell }$ (for the normalised measurements) and is always a large contribution to the total uncertainty.

Most sources of systematic uncertainty from the modelling of the detector and beam conditions are treated as fully correlated between bins. These comprise possible mis-modelling of the lepton energy (electron) and momentum (muon) scales and their resolution as well as mis-modelling of the lepton reconstruction, identification, trigger and isolation efficiencies [44–46]. Some of the detector uncertainties have a statistical component, which for the $p_\mathrm {T}^{\ell \ell }$ and integrated cross-section measurements is non-negligible and is propagated to the final measurements using a toy MC method. The above uncertainties are treated as uncorrelated between the two channels and are generally a small fraction of the total systematic uncertainty in the individual channels and on the combined result. The exceptions are the energy and momentum scale uncertainties, which become significant for the $p_\mathrm {T}^{\ell \ell }$ measurements at high values of $p_\mathrm {T}^{\ell \ell }$. Also considered are uncertainties due to mis-modelling of the pile-up distribution and of the distribution of the longitudinal position of the primary vertex, which are estimated by varying the associated MC scaling factor and are treated as correlated between channels. The pile-up uncertainty is a small, but non-negligible contribution to the total systematic uncertainty in most kinematic regions and the vertex uncertainty is generally even smaller. An uncertainty is estimated for the possible mis-modelling of the lepton angular resolution. This uncertainty is relevant only for the measurements of $\phi ^*_{\eta }$ and its size is found to be of an order similar to that of the pile-up uncertainty.

Important contributions to the total systematic uncertainty on both $\phi ^*_{\eta }$ and $p_\mathrm {T}^{\ell \ell }$ arise from the modelling of the background processes. The uncertainty arising from varying the normalisation of each MC background within its theoretical cross-section uncertainty is treated as correlated between channels. This source makes a small contribution to the total systematic uncertainty in the $m_{\ell \ell }$ region around the $Z$ -boson mass peak (where the total background is small), but becomes more significant in regions away from the peak. The dominant uncertainty on the multi-jet background arises from the difference in normalisation obtained from template fits performed in the distribution of the isolation variable or in $m_{\ell \ell }$. This is treated as fully correlated between bins and is generally a small contribution to the total uncertainty, becoming more important for the $m_{\ell \ell }$ regions below the $Z$ peak. The statistical uncertainty on the multi-jet background is considered as uncorrelated between bins and channels, and is small.

Several sources of systematic uncertainty are considered, arising from mis-modelling of the underlying physics distributions by the DrellΓÇôYan signal MC generator.

The effect of any mis-modelling of the underlying $\phi ^*_{\eta }$ and $p_\mathrm {T}^{\ell \ell }$ distributions is evaluated as follows. For $\phi ^*_{\eta }$ a second iteration of the bin-by-bin correction procedure (see Sect.┬á3.5) is made and any difference with respect to the first iteration is treated as a systematic uncertainty. This is found to be negligible in all kinematic regions, due to the very small bin-to-bin migration in $\phi ^*_{\eta }$. For $p_\mathrm {T}^{\ell \ell }$ the MC simulation is reweighted at particle level to the unfolded data and the unfolding is repeated. Any change is treated as a systematic uncertainty, which is always found to be a small fraction of the total uncertainty.

The systematic uncertainty due to the choice of signal MC generator used to correct the data is evaluated as follows. For $\phi ^*_{\eta }$ an uncertainty envelope is chosen that encompasses the difference in the bin-by-bin correction factors obtained using any individual signal MC sample compared to the central values. (As described in Sect.┬á3.5, the central values are obtained from an average over all available signal MC samples.) For $p_\mathrm {T}^{\ell \ell }$ the uncertainty is quoted as the difference in the results obtained when unfolding the data with Sherpa, as compared to Powheg+Pythia, which is used for the central values. This source results in a significant contribution to the systematic uncertainty in both $\phi ^*_{\eta }$ and $p_\mathrm {T}^{\ell \ell }$ for the $m_{\ell \ell }$ region around the $Z$ -boson mass peak. The systematic uncertainty on the Born-level measurements below the $Z$ -boson mass peak receives a significant contribution due to the differences in FSR modelling between Photos and Sherpa.

Potential uncertainties on the final $\phi ^*_{\eta }$ and $p_\mathrm {T}^{\ell \ell }$ distributions could arise from the modelling of the PDFs in the MC generators used to correct data to particle level. These are estimated using the CT10 error sets [28] using the LHAPDF interface [60], and are found to be negligible. A correction is applied to the Powheg+Pythia sample, which implements a running coupling for the photon exchange and a running width in the $Z$ -boson propagator. This correction is found to have a negligible effect on the final results.

Powheg+Pythia provides a poor description of the data for the samples with very low mass, $m_{\ell \ell }< 46 ~{\mathrm {GeV}}$ and $p_\mathrm {T}^{\ell \ell }> 45 ~{\mathrm {GeV}}$. The prediction from Powheg+Pythia is reweighted to that from Sherpa in order to evaluate an uncertainty due to this effect, which is found to be a small fraction of the total systematic uncertainty.

The Bayesian unfolding procedure used to correct the $p_\mathrm {T}^{\ell \ell }$ distributions for the effects of detector resolution and FSR has associated uncertainties. A statistical component is estimated using the bootstrap method [61] and the difference in the unfolded result between using six and seven iterations is treated as a systematic uncertainty, which is assumed fully correlated between bins of $p_\mathrm {T}^{\ell \ell }$ and found to be a small fraction of the total systematic uncertainty.

The uncertainty on the integrated luminosity is 2.8┬á%, which is determined following the methodology described in Ref. [62]. This has a negligible impact on the uncertainty in the normalised differential distributions $(1/\sigma )\, {\mathrm {d}}\sigma / {\mathrm {d}}\phi ^*_{\eta }$ and $(1/\sigma )\, {\mathrm {d}}\sigma / {\mathrm {d}}p_\mathrm {T}^{\ell \ell }$.

The total systematic uncertainties are generally smaller than the statistical uncertainties on the data. In $\phi ^*_{\eta }$ the total systematic uncertainties at the *Z*-boson mass peak are at the level of around 1ΓÇ░ at low $\phi ^*_{\eta }$, rising to around 0.5┬á% for high $\phi ^*_{\eta }$. In $p_\mathrm {T}^{\ell \ell }$ the total systematic uncertainties at the *Z*-boson mass peak are at the level of around 0.5┬á% at low $p_\mathrm {T}^{\ell \ell }$, rising to around 10┬á% for high $p_\mathrm {T}^{\ell \ell }$.

The full results for $(1/\sigma )\, {\mathrm {d}}\sigma / {\mathrm {d}}\phi ^*_{\eta }$ and $(1/\sigma )\, {\mathrm {d}}\sigma / {\mathrm {d}}p_\mathrm {T}^{\ell \ell }$ are presented in the Appendix in bins of $|y_{\ell \ell }|$, for which the size of the data statistical uncertainties relative to the systematic uncertainties are larger still.

## Results

### Combination procedure

The differential and integrated cross-section measurements in the electron-pair and muon-pair channels are combined at Born level using the HERA averager tool, which performs a $\chi ^2$ minimisation in which correlations between bins and between the two channels are taken into account [63]. The combinations for the $p_\mathrm {T}^{\ell \ell }$ and $\phi ^*_{\eta }$ measurements are performed separately in each region of $m_{\ell \ell }$ and $|y_{\ell \ell }|$.

### Differential cross-section measurements

Figure┬á5 shows the combined Born-level distributions of $(1/\sigma )\, {\mathrm {d}}\sigma / {\mathrm {d}}\phi ^*_{\eta }$, in three $m_{\ell \ell }$ regions from $46 ~{\mathrm {GeV}}$ to $150 ~{\mathrm {GeV}}$ for $|y_{\ell \ell }|<2.4$. The central panel of each plots in Fig.┬á5 shows the ratios of the values from the individual channels to the combined values and the lower panel of each plot shows the difference between the electron-pair and muon-pair values divided by the uncertainty on that difference (pull). The $\chi ^2$ per degree of freedom is given. The level of agreement between the electron-pair and muon-pair distributions is good. Figure┬á6 shows the equivalent set of plots for the distributions of $(1/\sigma )\, {\mathrm {d}}\sigma / {\mathrm {d}}p_\mathrm {T}^{\ell \ell }$ for the six regions of $m_{\ell \ell }$ from $12 ~{\mathrm {GeV}}$ to $150 ~{\mathrm {GeV}}$. Again the level of agreement between the two channels is good.

**Fig. 5:**
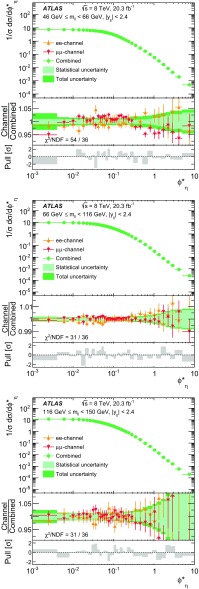
The Born-level distributions of $(1/\sigma )\, {\mathrm {d}}\sigma / {\mathrm {d}}\phi ^*_{\eta }$ for the combination of the electron-pair and muon-pair channels, shown in three $m_{\ell \ell }$ regions from 46 to $150 ~{\mathrm {GeV}}$ for $|y_{\ell \ell }|<2.4$. *The central panel of each plot* shows the ratios of the values from the individual channels to the combined values, where the *error bars* on the individual-channel measurements represent the total uncertainty uncorrelated between bins. *The light-green band* represents the data statistical uncertainty on the combined value and *the dark-green band* represents the total uncertainty (statistical and systematic). The $\chi ^2$ per degree of freedom is given. *The lower panel of each plot* shows the pull, defined as the difference between the electron-pair and muon-pair values divided by the uncertainty on that difference

The values of $(1/\sigma )\, {\mathrm {d}}\sigma / {\mathrm {d}}\phi ^*_{\eta }$ and $(1/\sigma )\, {\mathrm {d}}\sigma / {\mathrm {d}}p_\mathrm {T}^{\ell \ell }$ are given in tables in the Appendix for each region of $m_{\ell \ell }$ and $|y_{\ell \ell }|$ considered. The electron-pair results are given at the dressed and Born levels, and the muon-pair results at the bare, dressed and Born levels. The Born-level combined results are also given. The associated statistical and systematic uncertainties (both uncorrelated and correlated between bins in $\phi ^*_{\eta }$ or $p_\mathrm {T}^{\ell \ell }$) are provided in percentage form.

### Integrated cross-section measurements

In addition to detailed differential studies in $\phi ^*_{\eta }$ and $p_\mathrm {T}^{\ell \ell }$, integrated fiducial cross sections are provided for six regions in $m_{\ell \ell }$ from 12 to $150 ~{\mathrm {GeV}}$. The fiducial phase space is the same as for the $p_\mathrm {T}^{\ell \ell }$ measurements defined in Table┬á1. The Born-level fiducial cross sections are provided in Table┬á4 for the electron-pair and muon-pair channels separately, as well as for their combination. Uncertainties arising from data statistics, mis-modelling of the detector, background processes and of the MC signal samples used to correct the data are provided as a percentage of the cross section. The individual uncertainty sources after the combination are not necessarily orthogonal and also do not include uncertainties uncorrelated between bins of $m_{\ell \ell }$. Therefore their quadratic sum may not give the total systematic uncertainty.

These results are displayed in Fig.┬á7. In the channel combination the $\chi ^2$ per degree of freedom is 8/6, showing that the electron-pair and muon-pair measurements are consistent. A total uncertainty of 0.6┬á%, not including the uncertainty of 2.8┬á% on the integrated luminosity, is reached in the region of the $Z$ -boson mass peak. The fact that in some individual $m_{\ell \ell }$ bins the combined cross section does not lie at the naive weighted average of the individual channel values is due to the effect of systematic uncertainties that are correlated among $m_{\ell \ell }$ bins, but uncorrelated between channels (see, for example, Refs. [64, 65]).

## Comparison to QCD predictions

### Overview

The combined Born-level measurements of $\phi ^*_{\eta }$ and $p_\mathrm {T}^{\ell \ell }$ presented in Sect.┬á4 are compared in this section to a series of theoretical predictions.

A first general comparison is provided by Fig.┬á8. This shows the ratio of the predictions of ResBos for the *Z*-boson mass peak and for $|y_{\ell \ell }|$┬á<┬á2.4 to the combined Born-level data for $(1/\sigma )\,{\mathrm {d}}\sigma / {\mathrm {d}}\phi ^*_{\eta }$ and $(1/\sigma )\,{\mathrm {d}}\sigma / {\mathrm {d}}p_\mathrm {T}^{\ell \ell }$. In order to allow the features of these two distributions to be compared easily, the scales on the abscissae in Fig.┬á8 are aligned according to the approximate relationship [20]^5^ $\sqrt{2} m_Z \phi ^*_{\eta }\approx p_\mathrm {T}^{\ell \ell }$. The general features of the two distributions in Fig.┬á8 are similar. At low values of $\phi ^*_{\eta }$ and $p_\mathrm {T}^{\ell \ell }$, in which non-perturbative effects and soft-gluon resummation are most important, the predictions from ResBos are consistent with the data within the assigned theoretical uncertainties. However, at high values of $\phi ^*_{\eta }$ and $p_\mathrm {T}^{\ell \ell }$, which are more sensitive to the emission of hard partons, the predictions from ResBos are not consistent with the data within theoretical uncertainties. Figure┬á8 illustrates the particular power of $\phi ^*_{\eta }$ to probe the region of low $p_\mathrm {T}^{\ell \ell }$. Finer binning is possible in $\phi ^*_{\eta }$ than in $p_\mathrm {T}^{\ell \ell }$ whilst maintaining smaller systematic uncertainties from experimental resolution.

**Fig. 6:**
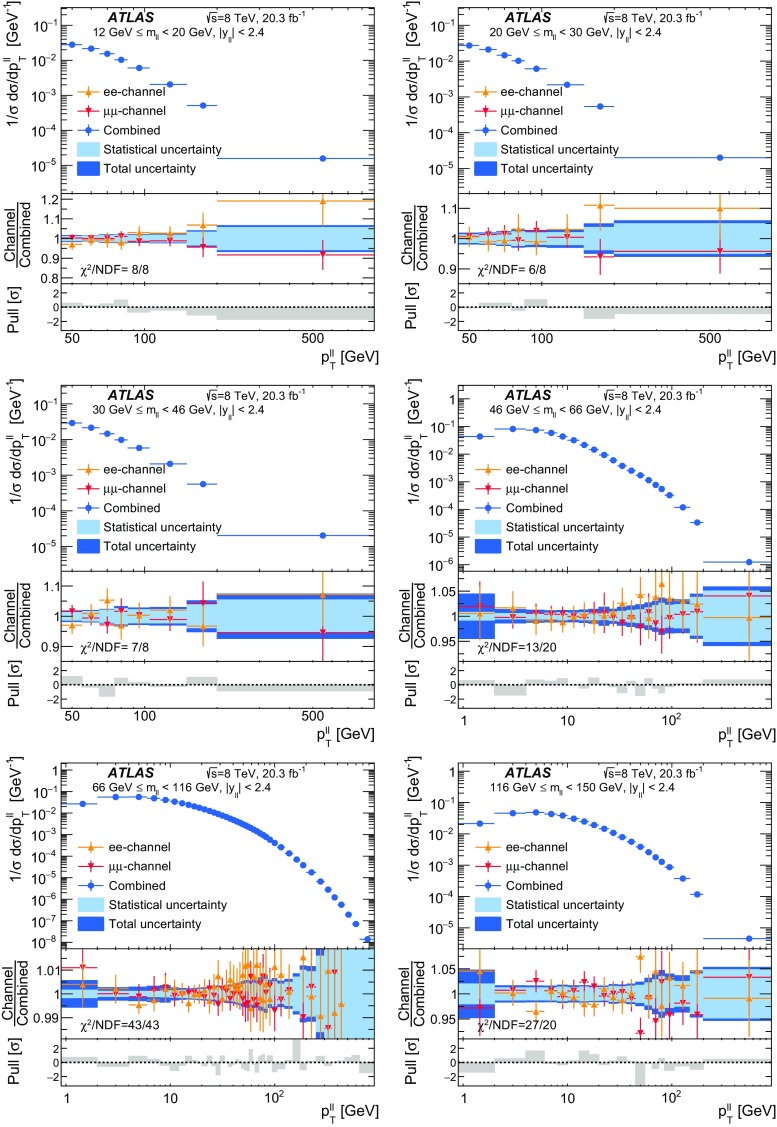
The Born-level distributions of $(1/\sigma )\, {\mathrm {d}}\sigma / {\mathrm {d}}p_\mathrm {T}^{\ell \ell }$ for the combination of the electron-pair and muon-pair channels, shown in six $m_{\ell \ell }$ regions for $|y_{\ell \ell }|<2.4$. *The central panel of each plot* shows the ratios of the values from the individual channels to the combined values, where the *error bars* on the individual-channel measurements represent the total uncertainty uncorrelated between bins. *The light-blue band* represents the data statistical uncertainty on the combined value and *the dark-blue band* represents the total uncertainty (statistical and systematic). The $\chi ^2$ per degree of freedom is given. *The lower panel of each plot* shows the pull, defined as the difference between the electron-pair and muon-pair values divided by the uncertainty on that difference

**Table 4:** Fiducial cross sections at Born level in the electron- and muon-pair channels as well as the combined value. The statistical and systematic uncertainties are given as a percentage of the cross section. An additional uncertainty of 2.8┬á% on the integrated luminosity, which is fully correlated between channels and among all $m_{\ell \ell }$ bins, pertains to these measurements. The individual uncertainty sources after the combination are not necessarily orthogonal and also do not include uncertainties uncorrelated between bins of $m_{\ell \ell }$. Therefore their quadratic sum may not give the total systematic uncertainty

$m_{\ell \ell }$ [GeV]	12ΓÇô20	20ΓÇô30	30ΓÇô46	46ΓÇô66	66ΓÇô116	116ΓÇô150
$\sigma (Z/\gamma ^*\rightarrow e^+e^-)$ [pb]	1.42	1.04	1.01	15.16	537.64	5.72
Statistical uncertainty [%]	0.91	1.05	1.13	0.28	0.04	0.41
Detector uncertainty [%]	2.28	2.12	1.79	3.47	0.83	0.87
Background uncertainty [%]	3.16	1.97	2.36	2.77	0.14	0.83
Model uncertainty [%]	5.11	4.38	3.59	1.59	0.16	0.74
Total systematic uncertainty [%]	6.43	5.25	4.66	4.72	0.86	1.41
$\sigma (Z/\gamma ^*\rightarrow \mu ^+\mu ^-)$ [pb]	1.45	1.04	0.97	14.97	535.25	5.48
Statistical uncertainty [%]	0.69	0.82	0.91	0.21	0.03	0.37
Detector uncertainty [%]	1.07	1.08	1.01	1.10	0.71	0.84
Background uncertainty [%]	0.75	2.19	2.00	1.48	0.04	0.97
Model uncertainty [%]	2.59	1.81	2.36	0.75	0.31	0.31
Total systematic uncertainty [%]	2.90	3.04	3.25	2.00	0.78	1.32
$\sigma (Z/\gamma ^*\rightarrow \ell ^+\ell ^-)$ [pb]	1.45	1.03	0.97	14.96	537.10	5.59
Statistical uncertainty [%]	0.63	0.75	0.83	0.17	0.03	0.31
Detector uncertainty [%]	0.84	0.99	0.87	1.05	0.40	0.56
Background uncertainty [%]	0.18	0.85	1.42	1.28	0.06	0.77
Model uncertainty [%]	1.84	2.24	2.27	0.89	0.19	0.50
Total systematic uncertainty [%]	2.06	2.44	2.38	1.82	0.45	1.03

**Fig. 7:**
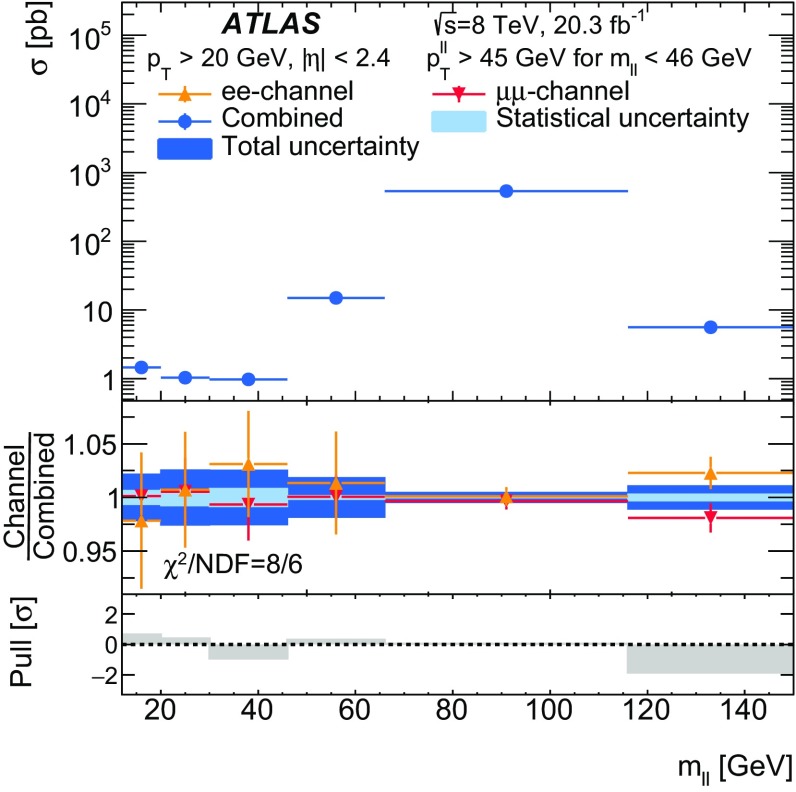
Born-level fiducial cross sections in bins of $m_{\ell \ell }$ for the combination of the electron-pair and muon-pair channels. *The middle plot* shows the ratios of the values from the individual channels to the combined values, where the *error bars* on the individual-channel measurements represent the total uncertainty uncorrelated between bins. *The light-blue band* represents the data statistical uncertainty on the combined value. *The dark-blue band* represents the total uncertainty (statistical and systematic), except for the uncertainty of 2.8┬á% on the integrated luminosity, which is fully correlated between channels and among all $m_{\ell \ell }$ bins. The $\chi ^2$ per degree of freedom is given. *The lower plot* shows the pull, defined as the difference between the electron-pair and muon-pair values divided by the uncertainty on that difference. The fiducial regions to which these cross sections correspond are specified in Table┬á1. Note that $p_\mathrm {T}^{\ell \ell }$ is required to be greater than $45 ~{\mathrm {GeV}}$ for $m_{\ell \ell }{}<46 ~{\mathrm {GeV}}$

**Fig. 8:**
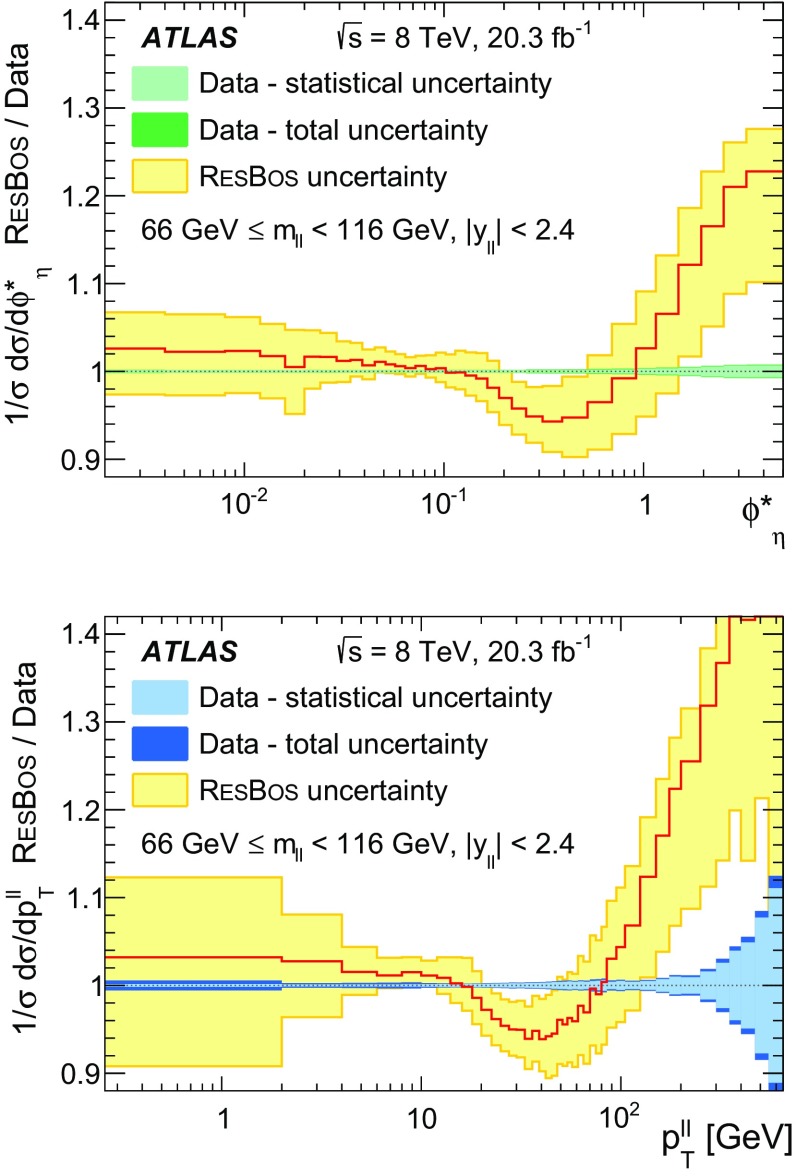
The ratio of the predictions of ResBos for the *Z*-boson mass peak and for $|y_{\ell \ell }|$┬á<┬á2.4 to the combined Born-level data for $(1/\sigma )\,{\mathrm {d}}\sigma / {\mathrm {d}}\phi ^*_{\eta }$ (*top*) and $(1/\sigma )\,{\mathrm {d}}\sigma / {\mathrm {d}}p_\mathrm {T}^{\ell \ell }$ (*bottom*). *The light-green* (*light-blue*) *band* represents the statistical uncertainty on the data for $\phi ^*_{\eta }$ ($p_\mathrm {T}^{\ell \ell }$) and *the dark-green* (*dark-blue*) *band* represents the total uncertainty (statistical and systematic) on the data. *The yellow band* represents the uncertainty in the ResBos calculation arising from varying (See footnote 2) the QCD scales, the non-perturbative parameter $a_Z$, and PDFs

The $\phi ^*_{\eta }$ measurements are compared in detail to predictions from ResBos in Sect.┬á5.2. In Sect.┬á5.3 the normalised $p_\mathrm {T}^{\ell \ell }$ measurements are compared to the predictions from a number of MC generators that use the parton-shower approach. The fixed-order predictions from Dynnlo1.3 [4] are compared to the absolute $p_\mathrm {T}^{\ell \ell }$ differential cross sections in Sect.┬á5.4.

### Comparison to resummed calculations

The predictions of $(1/\sigma )\,{\mathrm {d}}\sigma / {\mathrm {d}}\phi ^*_{\eta }$ from ResBos are compared to the Born-level measurements in Figs.┬á9, 10, 11, 12 and 13. As described above, $\phi ^*_{\eta }$ provides particularly precise measurements in the region sensitive to the effects of soft-gluon resummation and non-perturbative effects and therefore is the observable used to test the predictions from ResBos. Figure┬á9 shows the ratio of $(1/\sigma )\,{\mathrm {d}}\sigma / {\mathrm {d}}\phi ^*_{\eta }$ as predicted by ResBos to the combined Born-level data for the six $|y_{\ell \ell }|$ regions at the $Z$ -boson mass peak. Figure┬á10 shows the same comparison for the three $|y_{\ell \ell }|$ regions in the two $m_{\ell \ell }$ regions adjacent to the *Z*-boson mass peak. Also shown in these figures are the statistical and total uncertainties on the data, as well as the uncertainty in the ResBos calculation arising from varying (See footnote 2) the QCD scales, the non-perturbative parameter $a_Z$, and PDFs.

For values of $\phi ^*_{\eta }< 2$ for the $m_{\ell \ell }$ region around the $Z$ -boson mass peak the predictions from ResBos are generally consistent with the (much more precise) data within the assigned theoretical uncertainties. However, at larger values of $\phi ^*_{\eta }$ this is not the case. For the region of $m_{\ell \ell }$ above the *Z*-boson mass peak the predictions from ResBos are consistent with the data within uncertainties for all values of $\phi ^*_{\eta }$. For the region of $m_{\ell \ell }$ from 46 to $66 ~{\mathrm {GeV}}$ the predictions from ResBos lie below the data for $\phi ^*_{\eta }> 0.4$. In this context it may be noted that a known deficiency of the ResBos prediction is the lack of NNLO QCD corrections for the contributions from $\gamma ^{*}$ and from $Z/\gamma ^{*}$ interference. Similar deviations from the data in the mass region below the *Z* peak were observed in the D0 measurement in Ref. [23].

The theoretical uncertainties are highly correlated between different kinematic regions and therefore, as pointed out in Ref. [23], the ratio of $(1/\sigma )\,{\mathrm {d}}\sigma / {\mathrm {d}}\phi ^*_{\eta }$ in different kinematic regions enables a more precise comparison of the predictions with data. For example, the question of whether or not the non-perturbative contribution to $p_\mathrm {T}^{\ell \ell }$ varies with parton momentum fraction, *x*, or four-momentum transfer, $Q^2$, may be investigated by examining how the shape of $(1/\sigma )\, {\mathrm {d}}\sigma / {\mathrm {d}}\phi ^*_{\eta }$ evolves with $|y_{\ell \ell }|$ and $m_{\ell \ell }$ at low $\phi ^*_{\eta }$.

Figure┬á11 shows the ratio of the distribution of $(1/\sigma )\,{\mathrm {d}}\sigma / {\mathrm {d}}\phi ^*_{\eta }$ in each region of $|y_{\ell \ell }|$ to the distribution in the central region ($|y_{\ell \ell }|<0.4$), for events in the $m_{\ell \ell }$ region around the $Z$ -boson mass peak. The distributions are shown for data (with associated statistical and total uncertainties) as well as for ResBos. It can be seen that the uncertainties on the ResBos predictions, arising from varying (See footnote 2) the QCD scales, the non-perturbative parameter $a_Z$, and PDFs, are of a comparable size to the uncertainties on the corrected data. The predictions from ResBos are consistent with the data within the assigned uncertainties. Figure┬á12 shows equivalent comparisons for the $m_{\ell \ell }$ regions from $46 ~{\mathrm {GeV}}$ to $66 ~{\mathrm {GeV}}$ and from $116 ~{\mathrm {GeV}}$ to $150 ~{\mathrm {GeV}}$. It can be seen that the predictions from ResBos are again consistent with the data within the assigned uncertainties. Therefore it can be concluded that ResBos describes the evolution with $|y_{\ell \ell }|$ of the shape of the $(1/\sigma )\,{\mathrm {d}}\sigma / {\mathrm {d}}\phi ^*_{\eta }$ measurements well, and rather better than it describes the basic shape of the data (Figs.┬á9, 10).

Figure┬á13 shows the ratio of $(1/\sigma )\,{\mathrm {d}}\sigma / {\mathrm {d}}\phi ^*_{\eta }$ in the $m_{\ell \ell }$ region from $116 ~{\mathrm {GeV}}$ to $150 ~{\mathrm {GeV}}$ to that in the $m_{\ell \ell }$ region from $46 ~{\mathrm {GeV}}$ to $66 ~{\mathrm {GeV}}$, for the three divisions of $|y_{\ell \ell }|$. The ratio is shown for data (with associated statistical and total uncertainties) as well as for ResBos. It can again be seen that the uncertainties on the ResBos predictions, arising from varying (See footnote 2) the QCD scales, the non-perturbative parameter $a_Z$, and PDFs, and shown as a yellow band, are of a comparable size to the uncertainties on the corrected data. For values of $\phi ^*_{\eta }< 0.5$ the predictions from ResBos are consistent with the data within the assigned theoretical uncertainties showing that ResBos is able to describe the evolution of the $\phi ^*_{\eta }$ distribution with $m_{\ell \ell }$. However, at larger values of $\phi ^*_{\eta }$ this is not thecase.

**Fig. 9:**
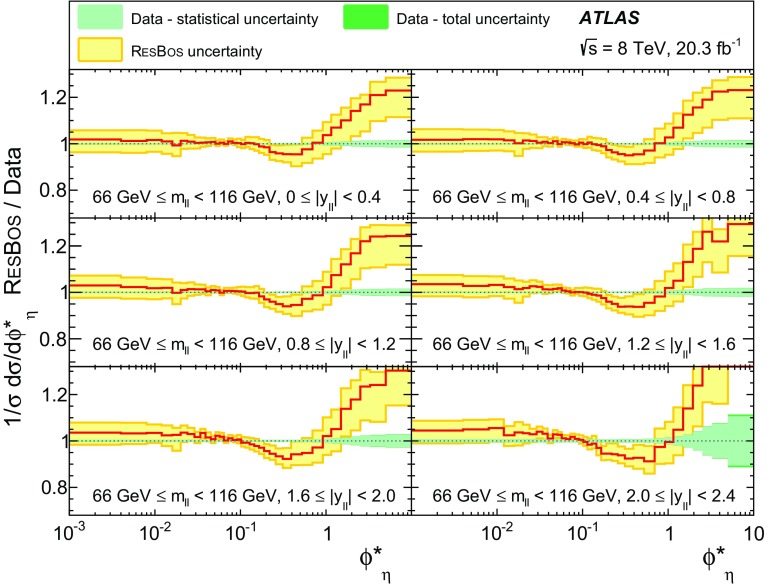
The ratio of $(1/\sigma )\,{\mathrm {d}}\sigma / {\mathrm {d}}\phi ^*_{\eta }$ as predicted by ResBos to the combined Born-level data, for the six $|y_{\ell \ell }|$ regions at the $Z$ -boson mass peak. *The light-green band* represents the statistical uncertainty on the data and *the dark-green band* represents the total uncertainty (statistical and systematic) on the data. *The yellow band* represents the uncertainty in the ResBos calculation arising from varying (See footnote 2) the QCD scales, the non-perturbative parameter $a_Z$, and PDFs

**Fig. 10:**
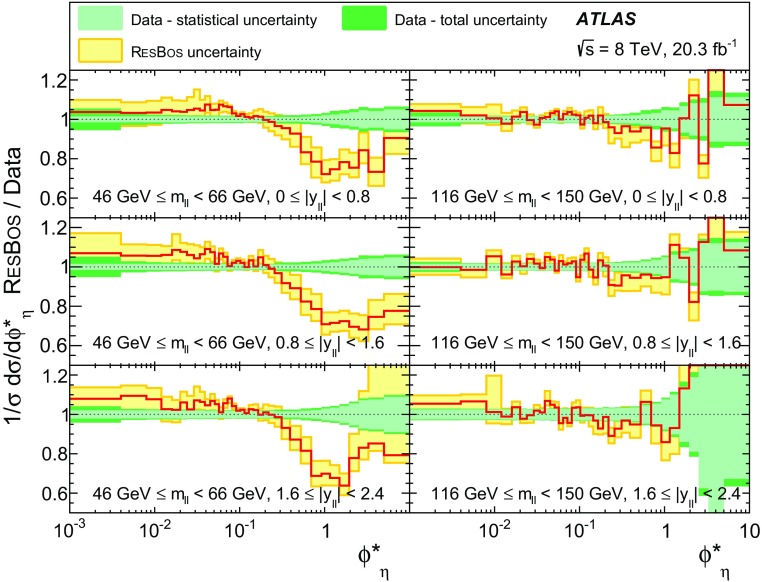
The ratio of $(1/\sigma )\,{\mathrm {d}}\sigma / {\mathrm {d}}\phi ^*_{\eta }$ as predicted by ResBos to the combined Born-level data, for the three $|y_{\ell \ell }|$ regions in the two $m_{\ell \ell }$ regions adjacent to the *Z*-boson mass peak. *The light-green band* represents the statistical uncertainty on the data and *the dark-green band* represents the total uncertainty (statistical and systematic) on the data. *The yellow band* represents the uncertainty in the ResBos calculation arising from varying (See footnote 2) the QCD scales, the non-perturbative parameter $a_Z$, and PDFs

**Fig. 11:**
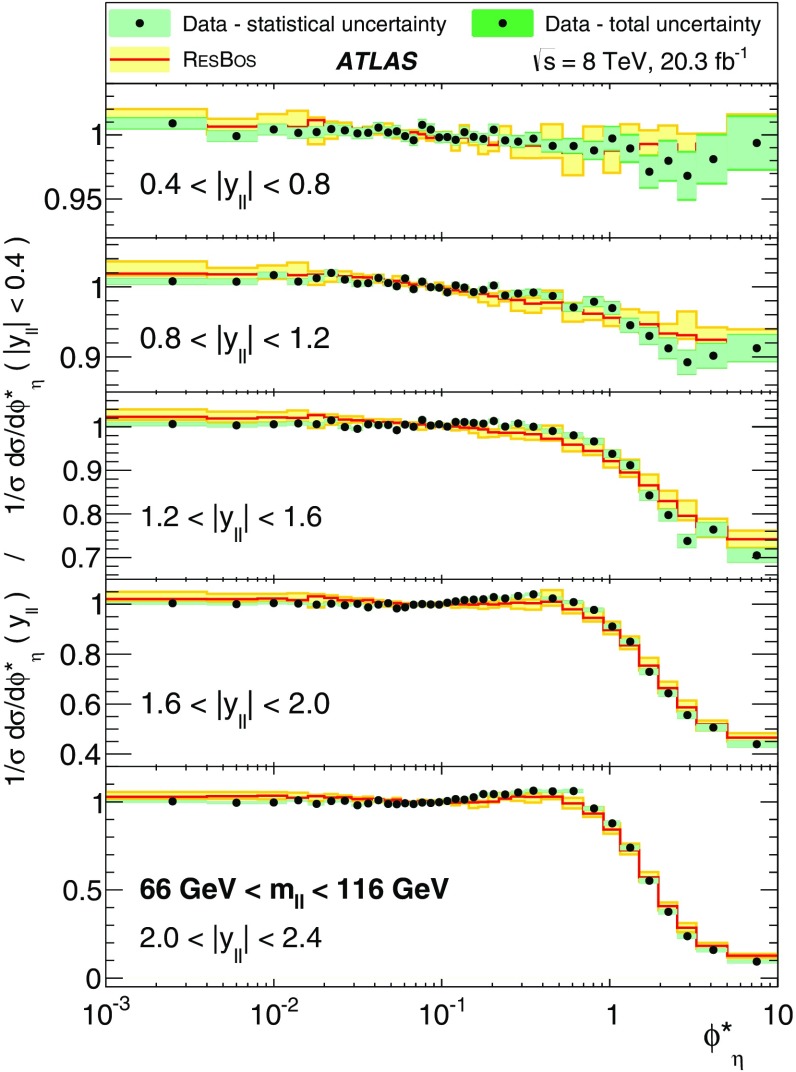
The distribution of $(1/\sigma )\,{\mathrm {d}}\sigma / {\mathrm {d}}\phi ^*_{\eta }$ at Born level in each region of $|y_{\ell \ell }|$, shown as a ratio to the central rapidity region ($|y_{\ell \ell }|<0.4$), for events at the $Z$ -boson mass peak. The data, shown as points, are compared to the predictions of ResBos. *The light-green band* represents the statistical uncertainty on the data and *the dark-green band* represents the total uncertainty on the data (treating systematic uncertainties as uncorrelated between regions of $|y_{\ell \ell }|$). *The yellow band* represents the uncertainty in the ResBos calculation arising from varying (See footnote 2) the QCD scales, the non-perturbative parameter $a_Z$, and PDFs

**Fig. 12:**
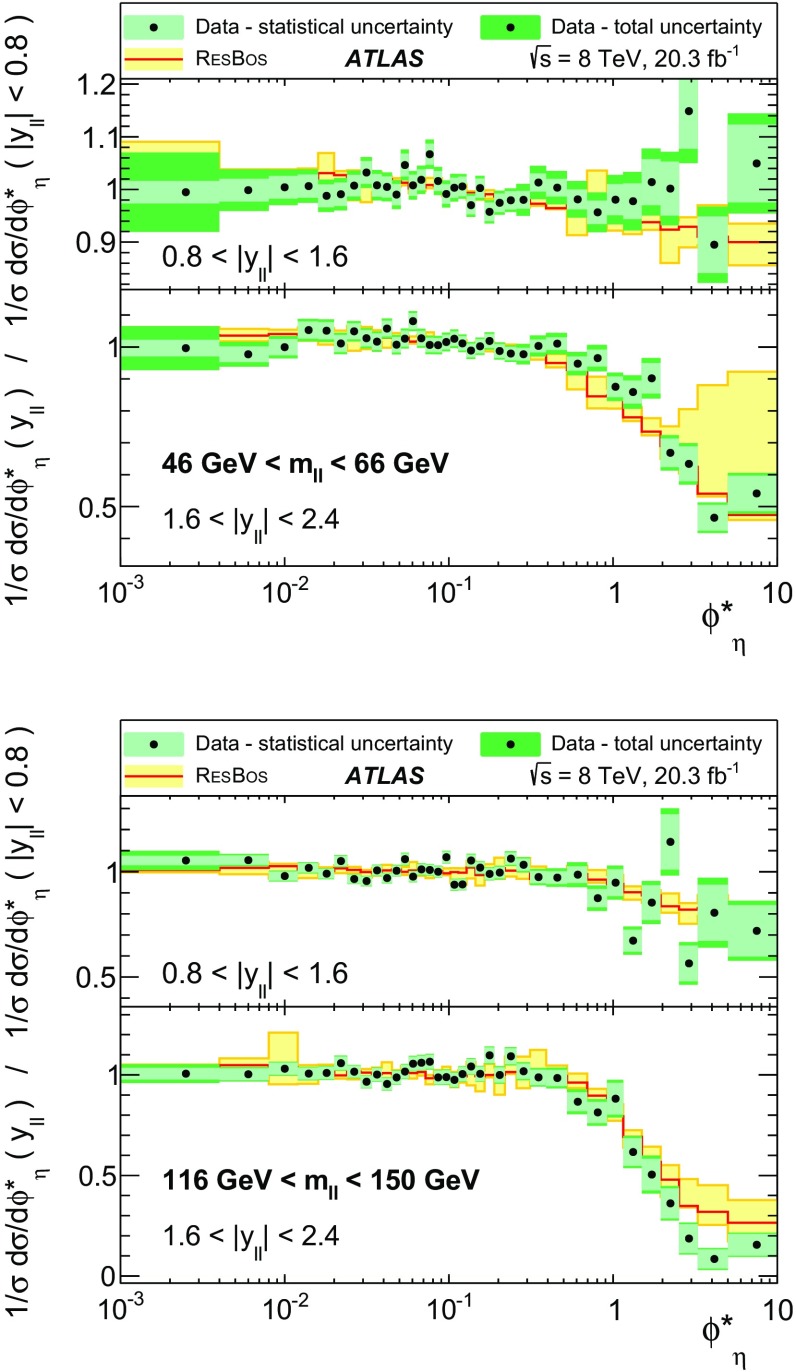
The distribution of $(1/\sigma )\,{\mathrm {d}}\sigma / {\mathrm {d}}\phi ^*_{\eta }$ at Born level in each region of $|y_{\ell \ell }|$, shown as a ratio to the central rapidity region ($|y_{\ell \ell }|<0.8$), for events with $m_{\ell \ell }$ between 46 to $66 ~{\mathrm {GeV}}$ (*upper plots*) and 116 to $150 ~{\mathrm {GeV}}$ (*lower plots*). The data, shown as points, are compared to the predictions of ResBos. *The light-green band* represents the statistical uncertainty on the data and *the dark-green band* represents the total uncertainty on the data (treating systematic uncertainties as uncorrelated between regions of $|y_{\ell \ell }|$). *The yellow band* represents the uncertainty in the ResBos calculation arising from varying (See footnote 2) the QCD scales, the non-perturbative parameter $a_Z$, and PDFs

**Fig. 13:**
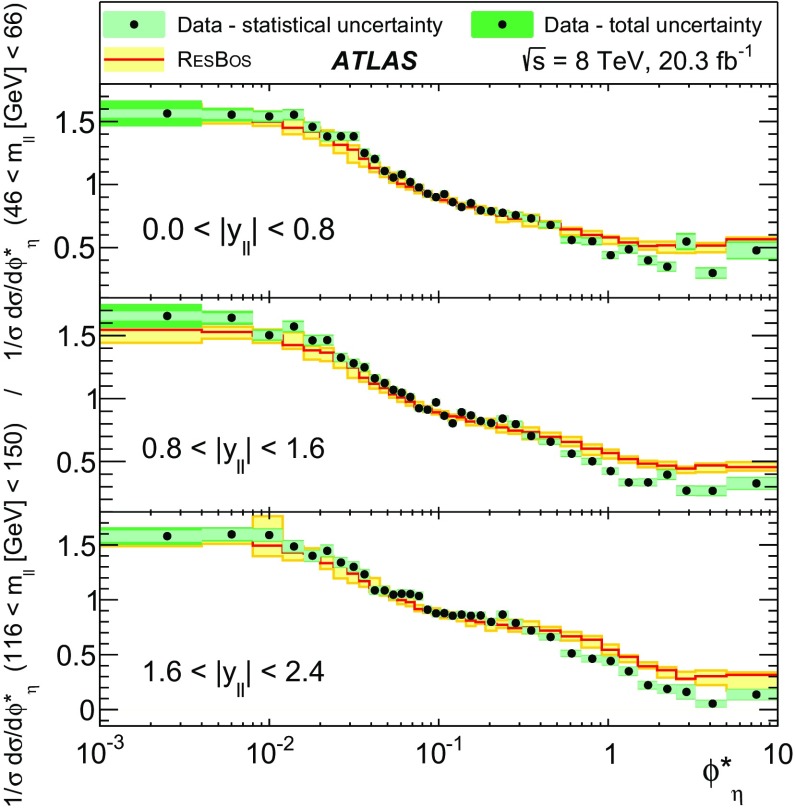
The ratio of $(1/\sigma )\,{\mathrm {d}}\sigma / {\mathrm {d}}\phi ^*_{\eta }$ in the $m_{\ell \ell }$ region from 116 to $150 ~{\mathrm {GeV}}$ to that in the $m_{\ell \ell }$ region from 46 to $66 ~{\mathrm {GeV}}$, for three regions of $|y_{\ell \ell }|$. The data, shown as points, are compared to the predictions of ResBos. *The light-green band* represents the statistical uncertainty on the data and *the dark-green band* represents the total uncertainty on the data (treating systematic uncertainties as uncorrelated between the mass regions). *The yellow band* represents the uncertainty in the ResBos calculation arising from varying (See footnote 2) the QCD scales, the non-perturbative parameter $a_Z$, and PDFs

### Comparison to parton-shower approaches

Figures┬á14, 15 and 16 show the comparison of the $(1/\sigma )\,{\mathrm {d}}\sigma / {\mathrm {d}}p_\mathrm {T}^{\ell \ell }$ distributions to the predictions of MC generators using the parton-shower approach: Powheg+Pythia (with both the AU2 [30] and AZNLO [14] tunes), Powheg+Herwig (only shown for the $m_{\ell \ell }$ region around the $Z$ peak) and Sherpa. Figure┬á14 shows the ratio of $(1/\sigma )\,{\mathrm {d}}\sigma / {\mathrm {d}}p_\mathrm {T}^{\ell \ell }$ as predicted by the MC generators, to the combined Born-level data in each of the six $m_{\ell \ell }$ regions for $|y_{\ell \ell }|< 2.4$. Figure┬á15 shows the ratio for each of the six $|y_{\ell \ell }|$ regions at the $Z$ -boson mass peak. Between $p_\mathrm {T}^{\ell \ell }$ values of approximately $5 ~{\mathrm {GeV}}$ and $100 ~{\mathrm {GeV}}$ for $m_{\ell \ell }{} > 46 ~{\mathrm {GeV}}$ the MC generators describe the shape of the data to within 10┬á%. However, outside this range, and in the regions with very low $m_{\ell \ell }$, the agreement worsens. For values of $p_\mathrm {T}^{\ell \ell }< 50 ~{\mathrm {GeV}}$ for the $m_{\ell \ell }$ region around the *Z*-boson mass peak the best description is provided by Powheg+Pythia (AZNLO), which was tuned to exactly this kinematic region in the $7 ~{\mathrm {TeV}}$ data [14]. However, at high values of $p_\mathrm {T}^{\ell \ell }$ around the *Z*-boson mass peak and in other $m_{\ell \ell }$ regions this MC tune does not describe the data well and also does not outperform the Powheg+Pythia AU2 tune. The differences between Sherpa and the data are generally of a similar magnitude, but of opposite sign, to those seen for Powheg+Pythia.

Figure┬á16 shows the ratio of the distribution of $(1/\sigma )\,{\mathrm {d}}\sigma /{\mathrm {d}}p_\mathrm {T}^{\ell \ell }$ in each region of $|y_{\ell \ell }|$ to the distribution in the central region ($|y_{\ell \ell }|<0.4$), for events in the $m_{\ell \ell }$ region around the $Z$ -boson mass peak. The distributions are shown for data (with associated statistical and total uncertainties) as well as for predictions from three parton-shower MC generators. The MC generators describe the data reasonably well over the entire range of $p_\mathrm {T}^{\ell \ell }$ and generally much better than they describe the $(1/\sigma )\,{\mathrm {d}}\sigma / {\mathrm {d}}p_\mathrm {T}^{\ell \ell }$ distributions (Figs.┬á14, 15) ΓÇô although there are discrepancies of up to 5┬á% with respect to data for $p_\mathrm {T}^{\ell \ell }<4 ~{\mathrm {GeV}}$.

For comparison with Fig.┬á14, Fig.┬á17 shows the ratio of $(1/\sigma )\,{\mathrm {d}}\sigma / {\mathrm {d}}\phi ^*_{\eta }$ as predicted by the MC generators, to the combined Born-level data in each of the three $m_{\ell \ell }$ regions from $46 ~{\mathrm {GeV}}$ to $150 ~{\mathrm {GeV}}$ for $|y_{\ell \ell }|< 2.4$. The differences between MC predictions and data seen in Fig.┬á17 are consistent with those seen in Fig.┬á14.

### Fixed-order QCD and electroweak corrections

Figure┬á18 shows the ratio of ${\mathrm {d}}\sigma / {\mathrm {d}}p_\mathrm {T}^{\ell \ell }$ as predicted by the fixed-order perturbative QCD predictions of Dynnlo to Born-level data for six regions of $m_{\ell \ell }$ from $12 ~{\mathrm {GeV}}$ to $150 ~{\mathrm {GeV}}$. The prediction is shown both with and without NLO EW corrections [53]. The data are shown with their associated statistical and total uncertainties. The predictions are not expected to describe the shape of the data for lower values of $p_\mathrm {T}^{\ell \ell }$, where it is known that the effects of soft-gluon emissions become important. At $p_\mathrm {T}^{\ell \ell }>30 ~{\mathrm {GeV}}$ the shape of the $p_\mathrm {T}^{\ell \ell }$ distribution is described within uncertainties by Dynnlo. However, the prediction is consistently low by about 15┬á% compared to the data across all $m_{\ell \ell }$ ranges, which is not covered by the evaluated scale and PDF uncertainties, although a recent calculation suggests the size of order $\alpha _{\mathrm {s}}^3$ corrections to be +(5ΓÇô10)┬á% for $p_\mathrm {T}^{\ell \ell }\gtrsim 60 ~{\mathrm {GeV}}$ [66]. The observed behaviour of Dynnlo is consistent with the results at $\sqrt{s}=7 ~{\mathrm {TeV}}$ near the *Z* peak [14]. The application of NLO EW corrections predicts an approximately 5┬á% increase of the cross section below the *Z*-peak region due to effects of $\gamma ^*$ exchange, while a suppression of up to 20┬á% at highest $p_\mathrm {T}^{\ell \ell }$ is predicted due to large Sudakov logarithms [53]. The change in the prediction induced by the addition of the EW corrections is significantly smaller than both the uncertainty on the NNLO QCD prediction and the difference between the prediction and data. Therefore, no conclusions can be drawn on whether or not their addition leads to an improvement in agreement between data and theory.

**Fig. 14:**
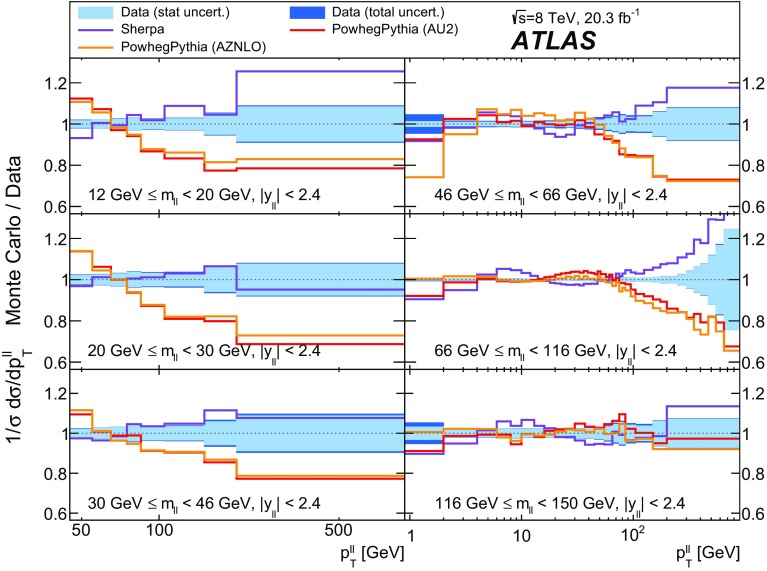
The ratio of $(1/\sigma )\,{\mathrm {d}}\sigma / {\mathrm {d}}p_\mathrm {T}^{\ell \ell }$ as predicted by various MC generators to the combined Born-level data, in six different regions of $m_{\ell \ell }$ for $|y_{\ell \ell }|< 2.4$. *The light-blue band* represents the statistical uncertainty on the data and *the dark-blue band* represents the total uncertainty (statistical and systematic) on the data

## Conclusion

Measurements are presented of the $\phi ^*_{\eta }$ and $p_\mathrm {T}^{\ell \ell }$ distributions of DrellΓÇôYan lepton-pair events using 20.3┬á$\mathrm{fb}^{-1}$ of $\sqrt{s}=8 ~{\mathrm {TeV}}$ *pp* collision data collected with the ATLAS detector. The results presented here expand upon those presented previously by ATLAS at $\sqrt{s}=7 ~{\mathrm {TeV}}$, by providing measurements in regions of $m_{\ell \ell }$ above and below, as well as on, the $Z$ -boson mass peak, and also in finer divisions of $|y_{\ell \ell }|$ than were presented at $\sqrt{s}=7 ~{\mathrm {TeV}}$. Measurements for both the electron- and muon-pair channels are provided corresponding to a variety of particle-level definitions that differ in the size of the correction for final-state photon radiation. The results from the two channels at the Born level are combined and compared to a variety of theoretical predictions. In addition, measurements of the integrated cross section in six bins of $m_{\ell \ell }$ are given.

**Fig. 15:**
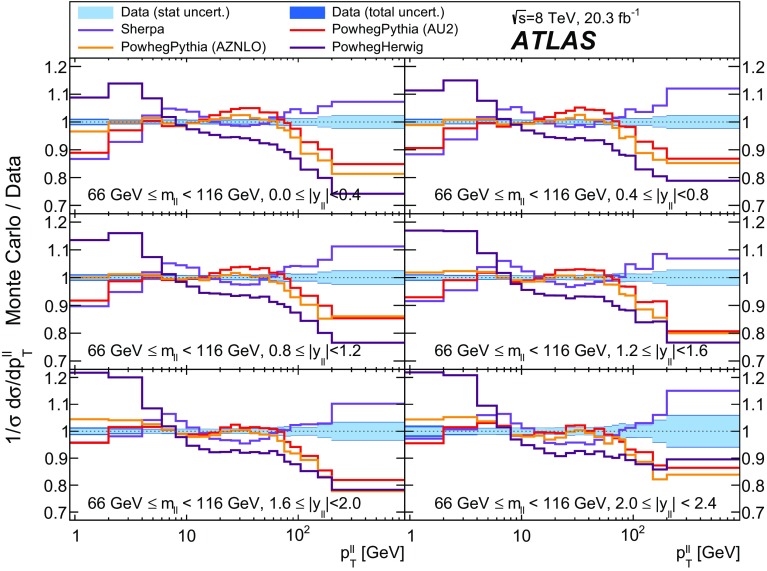
The ratio of $(1/\sigma )\,{\mathrm {d}}\sigma / {\mathrm {d}}p_\mathrm {T}^{\ell \ell }$ as predicted by various MC generators to the combined Born-level data, in different $|y_{\ell \ell }|$ ranges for events at the $Z$ -boson mass peak. *The light-blue band* represents the statistical uncertainty on the data and *the dark-blue band* represents the total uncertainty (statistical and systematic) on the data

**Fig. 16:**
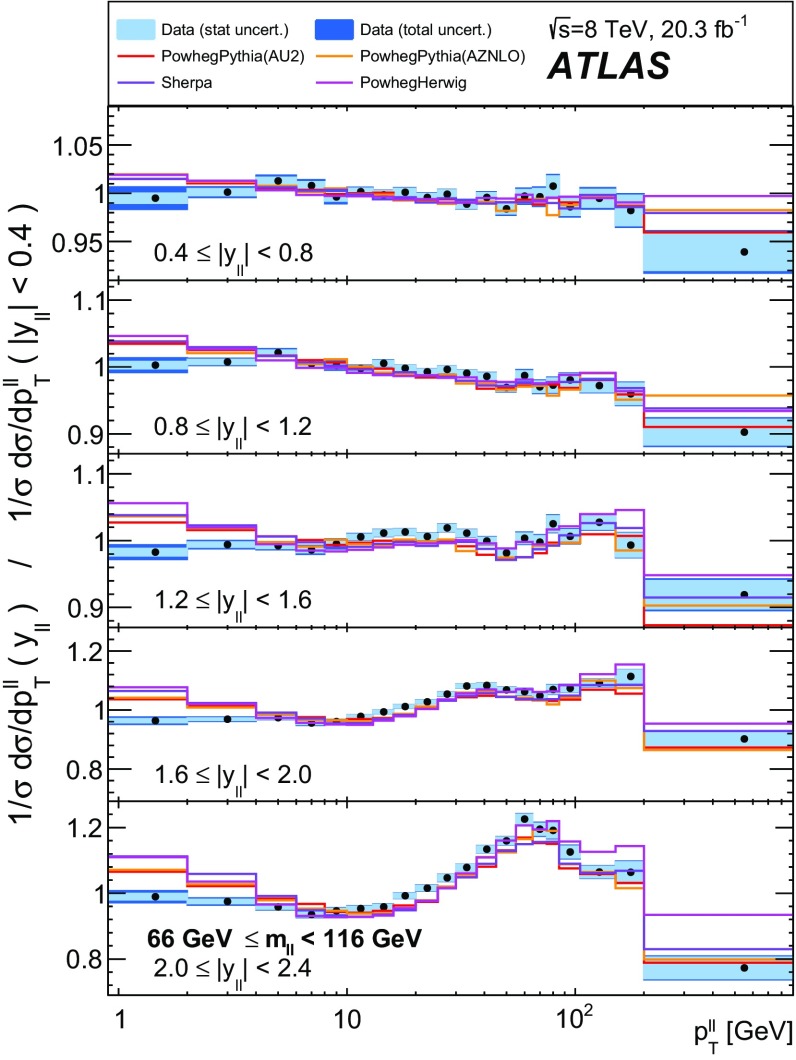
The distribution of $(1/\sigma )\,{\mathrm {d}}\sigma / {\mathrm {d}}p_\mathrm {T}^{\ell \ell }$ at Born level in each region of $|y_{\ell \ell }|$, shown as a ratio to the central rapidity region ($|y_{\ell \ell }|<0.4$), for events at the $Z$ -boson mass peak. The data, shown as points, are compared to the predictions of various MC generators. *The light-blue band* represents the statistical uncertainty on the data and *the dark-blue band* represents the total uncertainty on the data (treating systematic uncertainties as uncorrelated between regions of $|y_{\ell \ell }|$)

**Fig. 17:**
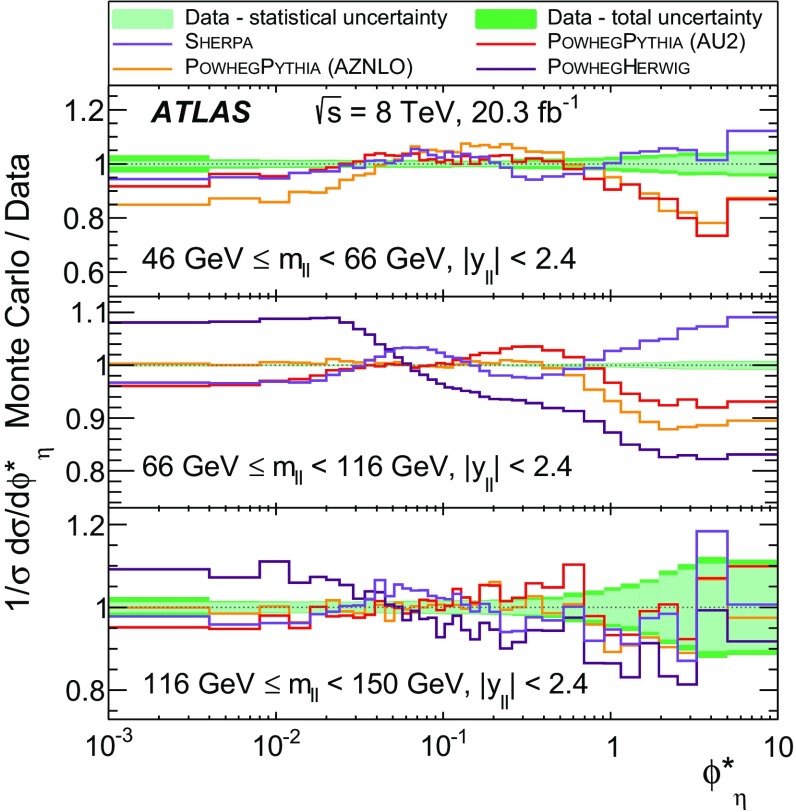
The ratio of $(1/\sigma )\,{\mathrm {d}}\sigma / {\mathrm {d}}\phi ^*_{\eta }$ as predicted by various MC generators to the combined Born-level data, in three different regions of $m_{\ell \ell }$ for $|y_{\ell \ell }|< 2.4$. *The light-green band* represents the statistical uncertainty on the data and *the dark-green band* represents the total uncertainty (statistical and systematic) on the data

The predictions from ResBos, which include the effects of soft-gluon resummation, are compared to the normalised $\phi ^*_{\eta }$ distributions $(1/\sigma )\, {\mathrm {d}}\sigma / {\mathrm {d}}\phi ^*_{\eta }$. These predictions are consistent with the data within the assigned theoretical uncertainties within certain kinematic regions, especially at low values of $\phi ^*_{\eta }$: $\phi ^*_{\eta }< 0.4$ for $46 ~{\mathrm {GeV}}< m_{\ell \ell }< 66 ~{\mathrm {GeV}}$; $\phi ^*_{\eta }< 2$ for $66 ~{\mathrm {GeV}}< m_{\ell \ell }< 116 ~{\mathrm {GeV}}$; and over the full range of $\phi ^*_{\eta }$ for $116 ~{\mathrm {GeV}}< m_{\ell \ell }< 150 ~{\mathrm {GeV}}$. However, outside these kinematic ranges, i.e., for larger values of $\phi ^*_{\eta }$, the predictions show significant deviations from the data. The evolution of $(1/\sigma )\, {\mathrm {d}}\sigma / {\mathrm {d}}\phi ^*_{\eta }$ with $|y_{\ell \ell }|$ and $m_{\ell \ell }$ (for which the theoretical uncertainties on the predictions largely cancel) is generally well described by ResBos.

Predictions from MC generators with parton showers are compared to the normalised $p_\mathrm {T}^{\ell \ell }$ distributions in a similar manner. Between $p_\mathrm {T}^{\ell \ell }$ values of approximately $5 ~{\mathrm {GeV}}$ and $100 ~{\mathrm {GeV}}$ for $m_{\ell \ell }{} > 46 ~{\mathrm {GeV}}$ the MC generators describe the basic shape of the data to within 10┬á%. However outside this range, and in the very-low regions of $m_{\ell \ell }$ the agreement worsens. The MC generators do though provide a reasonable description of the evolution of the $p_\mathrm {T}^{\ell \ell }$ distributions with $|y_{\ell \ell }|$ for the $m_{\ell \ell }$ region around the $Z$ -boson mass peak. Fixed-order predictions from Dynnlo are compared to the absolute $p_\mathrm {T}^{\ell \ell }$ differential cross-section distributions. The predictions describe the shape of the data within uncertainties for $p_\mathrm {T}^{\ell \ell }>40 ~{\mathrm {GeV}}$ but only describe the absolute values to within 15┬á%, which is not covered by the evaluated scale and PDF uncertainties. The data and QCD predictions are not precise enough to be sensitive to the inclusion of EW corrections.

**Fig. 18:**
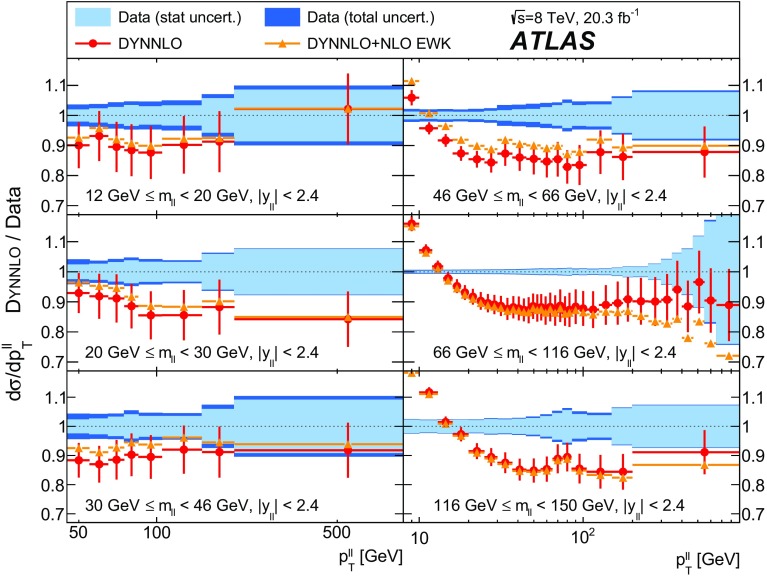
The ratio of ${\mathrm {d}}\sigma / {\mathrm {d}}p_\mathrm {T}^{\ell \ell }$ as predicted by the Dynnlo MC generator to the combined Born-level data, for six regions of $m_{\ell \ell }$from 12 to $150 ~{\mathrm {GeV}}$. Two sets of Dynnlo predictions are shown, one of which includes NLO EW corrections while the other does not. The *error bars* on the Dynnlo predictions represent the uncertainty arising from varying the QCD scales and PDFs. Additional uncertainties introduced by the inclusion of the EW corrections are at the level of 2ΓÇô4┬á% and are always significantly smaller than the QCD scale and PDF uncertainties. Therefore for clarity these points are shown without uncertainty *bars*. *The light-blue band* represents the statistical uncertainty on the data and *the dark-blue band* represents the total uncertainty (statistical and systematic) on the data

## Appendix

In the Tables┬á6, 7, 8, 9, 10, 11, 12, 13, 14, 15, 16, 17, 18, 19, 20, 21, 22, 23, 24, 25, 26, 27, 28, 29, 30 and 31 in this appendix the values of $(1/\sigma )\, d\sigma / d\phi ^*_{\eta }$ and $(1/\sigma )\, d\sigma / dp_\mathrm {T}^{\ell \ell }$ are given for each region of $m_{\ell \ell }$ and $|y_{\ell \ell }|$ considered. The electron-pair results are given at the dressed and Born levels, and the muon-pair results at the bare, dressed and Born levels. The Born-level combined results are also given. The associated statistical and systematic uncertainties (both uncorrelated and correlated between bins in $\phi ^*_{\eta }$ or $p_\mathrm {T}^{\ell \ell }$) are provided in percentage form.

**Table 5:** The values of $(1/\sigma )\,{\mathrm {d}}\sigma /{\mathrm {d}}\phi ^*_{\eta }$ in each bin of $\phi ^*_{\eta }$ for the electron and muon channels separately (for various particle-level definitions) and for the Born-level combination in the kinematic region $46\ ~{\mathrm {GeV}}\le m_{\ell \ell }< 66\ ~{\mathrm {GeV}},\ 0 \le |y_{\ell \ell }| < 0.8$. The associated statistical and systematic (both uncorrelated and correlated between bins of $\phi ^*_{\eta }$) are provided in percentage form

Bin	$(1/\sigma )\,d\sigma /d\phi ^*_{\eta }$ ┬▒ Statistical [%] ┬▒ Uncorrelated systematic [%] ┬▒ Correlated systematic [%]					
	Electron channel		Muon channel			Combination
	Dressed	Born	Bare	Dressed	Born	Born
0ΓÇô0.004	6.778┬á┬▒┬á2.4┬á┬▒┬á0.9┬á┬▒┬á5.9	7.256┬á┬▒┬á2.4┬á┬▒┬á1.0┬á┬▒┬á6.0	6.688┬á┬▒┬á2.0┬á┬▒┬á0.8┬á┬▒┬á5.0	6.687┬á┬▒┬á2.0┬á┬▒┬á0.8┬á┬▒┬á5.0	7.157┬á┬▒┬á2.0┬á┬▒┬á0.8┬á┬▒┬á5.2	7.248┬á┬▒┬á1.5┬á┬▒┬á0.6┬á┬▒┬á5.3
0.004ΓÇô0.008	6.662┬á┬▒┬á2.3┬á┬▒┬á0.9┬á┬▒┬á2.3	7.051┬á┬▒┬á2.3┬á┬▒┬á0.9┬á┬▒┬á2.6	7.079┬á┬▒┬á2.0┬á┬▒┬á0.8┬á┬▒┬á1.9	7.046┬á┬▒┬á2.0┬á┬▒┬á0.8┬á┬▒┬á1.9	7.469┬á┬▒┬á2.0┬á┬▒┬á0.8┬á┬▒┬á2.4	7.258┬á┬▒┬á1.5┬á┬▒┬á0.6┬á┬▒┬á2.3
0.008ΓÇô0.012	6.781┬á┬▒┬á2.3┬á┬▒┬á0.9┬á┬▒┬á1.5	7.179┬á┬▒┬á2.3┬á┬▒┬á0.9┬á┬▒┬á1.9	6.747┬á┬▒┬á2.1┬á┬▒┬á0.8┬á┬▒┬á1.4	6.704┬á┬▒┬á2.1┬á┬▒┬á0.8┬á┬▒┬á1.4	7.169┬á┬▒┬á2.1┬á┬▒┬á0.8┬á┬▒┬á2.0	7.141┬á┬▒┬á1.5┬á┬▒┬á0.6┬á┬▒┬á1.7
0.012ΓÇô0.016	6.561┬á┬▒┬á2.3┬á┬▒┬á0.9┬á┬▒┬á1.1	6.926┬á┬▒┬á2.3┬á┬▒┬á0.9┬á┬▒┬á1.6	6.680┬á┬▒┬á2.0┬á┬▒┬á0.8┬á┬▒┬á1.1	6.660┬á┬▒┬á2.1┬á┬▒┬á0.8┬á┬▒┬á1.1	7.085┬á┬▒┬á2.0┬á┬▒┬á0.8┬á┬▒┬á1.8	6.981┬á┬▒┬á1.5┬á┬▒┬á0.6┬á┬▒┬á1.5
0.016ΓÇô0.020	6.540┬á┬▒┬á2.3┬á┬▒┬á0.9┬á┬▒┬á1.0	6.927┬á┬▒┬á2.3┬á┬▒┬á1.0┬á┬▒┬á1.5	6.542┬á┬▒┬á2.0┬á┬▒┬á0.8┬á┬▒┬á0.9	6.484┬á┬▒┬á2.0┬á┬▒┬á0.8┬á┬▒┬á0.9	6.884┬á┬▒┬á2.0┬á┬▒┬á0.8┬á┬▒┬á1.6	6.861┬á┬▒┬á1.5┬á┬▒┬á0.6┬á┬▒┬á1.4
0.020ΓÇô0.024	6.327┬á┬▒┬á2.3┬á┬▒┬á0.9┬á┬▒┬á1.1	6.714┬á┬▒┬á2.3┬á┬▒┬á0.9┬á┬▒┬á1.6	6.437┬á┬▒┬á2.1┬á┬▒┬á0.9┬á┬▒┬á0.9	6.415┬á┬▒┬á2.1┬á┬▒┬á0.9┬á┬▒┬á0.9	6.755┬á┬▒┬á2.1┬á┬▒┬á0.9┬á┬▒┬á1.7	6.693┬á┬▒┬á1.6┬á┬▒┬á0.6┬á┬▒┬á1.4
0.024ΓÇô0.029	6.102┬á┬▒┬á2.1┬á┬▒┬á0.8┬á┬▒┬á0.9	6.408┬á┬▒┬á2.1┬á┬▒┬á0.9┬á┬▒┬á1.4	6.072┬á┬▒┬á1.9┬á┬▒┬á0.8┬á┬▒┬á0.9	6.075┬á┬▒┬á1.9┬á┬▒┬á0.8┬á┬▒┬á0.9	6.472┬á┬▒┬á1.9┬á┬▒┬á0.8┬á┬▒┬á1.5	6.398┬á┬▒┬á1.4┬á┬▒┬á0.6┬á┬▒┬á1.2
0.029ΓÇô0.034	5.682┬á┬▒┬á2.2┬á┬▒┬á0.8┬á┬▒┬á0.8	5.957┬á┬▒┬á2.2┬á┬▒┬á0.9┬á┬▒┬á1.4	5.877┬á┬▒┬á2.0┬á┬▒┬á0.8┬á┬▒┬á0.8	5.904┬á┬▒┬á2.0┬á┬▒┬á0.8┬á┬▒┬á0.8	6.214┬á┬▒┬á2.0┬á┬▒┬á0.8┬á┬▒┬á1.4	6.062┬á┬▒┬á1.5┬á┬▒┬á0.6┬á┬▒┬á1.2
0.034ΓÇô0.039	5.868┬á┬▒┬á2.2┬á┬▒┬á0.8┬á┬▒┬á0.9	6.185┬á┬▒┬á2.2┬á┬▒┬á0.9┬á┬▒┬á1.4	5.468┬á┬▒┬á2.1┬á┬▒┬á0.8┬á┬▒┬á0.8	5.482┬á┬▒┬á2.1┬á┬▒┬á0.8┬á┬▒┬á0.8	5.798┬á┬▒┬á2.1┬á┬▒┬á0.8┬á┬▒┬á1.4	5.919┬á┬▒┬á1.5┬á┬▒┬á0.6┬á┬▒┬á1.2
0.039ΓÇô0.045	5.263┬á┬▒┬á2.1┬á┬▒┬á0.8┬á┬▒┬á0.5	5.485┬á┬▒┬á2.1┬á┬▒┬á0.9┬á┬▒┬á1.3	5.428┬á┬▒┬á1.9┬á┬▒┬á0.8┬á┬▒┬á0.8	5.449┬á┬▒┬á1.9┬á┬▒┬á0.8┬á┬▒┬á0.8	5.669┬á┬▒┬á1.9┬á┬▒┬á0.8┬á┬▒┬á1.4	5.544┬á┬▒┬á1.4┬á┬▒┬á0.6┬á┬▒┬á1.1
0.045ΓÇô0.051	5.032┬á┬▒┬á2.1┬á┬▒┬á0.8┬á┬▒┬á0.6	5.274┬á┬▒┬á2.1┬á┬▒┬á0.9┬á┬▒┬á1.3	5.182┬á┬▒┬á1.9┬á┬▒┬á0.8┬á┬▒┬á0.9	5.210┬á┬▒┬á1.9┬á┬▒┬á0.8┬á┬▒┬á0.9	5.505┬á┬▒┬á1.9┬á┬▒┬á0.8┬á┬▒┬á1.5	5.351┬á┬▒┬á1.4┬á┬▒┬á0.6┬á┬▒┬á1.1
0.051ΓÇô0.057	4.796┬á┬▒┬á2.2┬á┬▒┬á0.8┬á┬▒┬á0.6	4.964┬á┬▒┬á2.2┬á┬▒┬á0.8┬á┬▒┬á1.3	4.862┬á┬▒┬á2.0┬á┬▒┬á0.8┬á┬▒┬á0.8	4.897┬á┬▒┬á2.0┬á┬▒┬á0.8┬á┬▒┬á0.8	5.111┬á┬▒┬á2.0┬á┬▒┬á0.8┬á┬▒┬á1.4	5.003┬á┬▒┬á1.5┬á┬▒┬á0.6┬á┬▒┬á1.1
0.057ΓÇô0.064	4.443┬á┬▒┬á2.1┬á┬▒┬á0.8┬á┬▒┬á0.7	4.603┬á┬▒┬á2.1┬á┬▒┬á0.8┬á┬▒┬á1.3	4.430┬á┬▒┬á1.9┬á┬▒┬á0.8┬á┬▒┬á0.5	4.443┬á┬▒┬á1.9┬á┬▒┬á0.8┬á┬▒┬á0.5	4.663┬á┬▒┬á1.9┬á┬▒┬á0.8┬á┬▒┬á1.5	4.597┬á┬▒┬á1.4┬á┬▒┬á0.6┬á┬▒┬á1.2
0.064ΓÇô0.072	4.113┬á┬▒┬á2.0┬á┬▒┬á0.8┬á┬▒┬á0.6	4.271┬á┬▒┬á2.0┬á┬▒┬á0.8┬á┬▒┬á1.3	4.052┬á┬▒┬á1.9┬á┬▒┬á0.8┬á┬▒┬á0.4	4.082┬á┬▒┬á1.9┬á┬▒┬á0.8┬á┬▒┬á0.4	4.256┬á┬▒┬á1.9┬á┬▒┬á0.8┬á┬▒┬á1.4	4.245┬á┬▒┬á1.4┬á┬▒┬á0.6┬á┬▒┬á1.2
0.072ΓÇô0.081	3.766┬á┬▒┬á2.0┬á┬▒┬á0.7┬á┬▒┬á0.7	3.876┬á┬▒┬á2.0┬á┬▒┬á0.8┬á┬▒┬á1.3	3.759┬á┬▒┬á1.8┬á┬▒┬á0.7┬á┬▒┬á0.5	3.787┬á┬▒┬á1.8┬á┬▒┬á0.7┬á┬▒┬á0.5	3.912┬á┬▒┬á1.8┬á┬▒┬á0.7┬á┬▒┬á1.5	3.866┬á┬▒┬á1.3┬á┬▒┬á0.5┬á┬▒┬á1.2
0.081ΓÇô0.091	3.400┬á┬▒┬á2.0┬á┬▒┬á1.2┬á┬▒┬á1.0	3.495┬á┬▒┬á2.0┬á┬▒┬á1.2┬á┬▒┬á1.5	3.517┬á┬▒┬á1.8┬á┬▒┬á0.7┬á┬▒┬á0.5	3.521┬á┬▒┬á1.8┬á┬▒┬á0.7┬á┬▒┬á0.5	3.665┬á┬▒┬á1.8┬á┬▒┬á0.7┬á┬▒┬á1.5	3.580┬á┬▒┬á1.3┬á┬▒┬á0.7┬á┬▒┬á1.2
0.091ΓÇô0.102	3.231┬á┬▒┬á2.0┬á┬▒┬á0.7┬á┬▒┬á0.8	3.318┬á┬▒┬á2.0┬á┬▒┬á0.8┬á┬▒┬á1.4	3.107┬á┬▒┬á1.8┬á┬▒┬á0.7┬á┬▒┬á0.5	3.130┬á┬▒┬á1.8┬á┬▒┬á0.7┬á┬▒┬á0.5	3.224┬á┬▒┬á1.8┬á┬▒┬á0.7┬á┬▒┬á1.5	3.240┬á┬▒┬á1.3┬á┬▒┬á0.5┬á┬▒┬á1.2
0.102ΓÇô0.114	2.833┬á┬▒┬á2.0┬á┬▒┬á0.7┬á┬▒┬á0.8	2.848┬á┬▒┬á2.0┬á┬▒┬á0.8┬á┬▒┬á1.4	2.814┬á┬▒┬á1.8┬á┬▒┬á0.7┬á┬▒┬á0.5	2.822┬á┬▒┬á1.8┬á┬▒┬á0.7┬á┬▒┬á0.5	2.882┬á┬▒┬á1.8┬á┬▒┬á0.7┬á┬▒┬á1.5	2.844┬á┬▒┬á1.3┬á┬▒┬á0.5┬á┬▒┬á1.2
0.114ΓÇô0.128	2.555┬á┬▒┬á2.0┬á┬▒┬á0.7┬á┬▒┬á0.7	2.596┬á┬▒┬á2.0┬á┬▒┬á0.8┬á┬▒┬á1.3	2.477┬á┬▒┬á1.8┬á┬▒┬á0.7┬á┬▒┬á0.7	2.487┬á┬▒┬á1.8┬á┬▒┬á0.7┬á┬▒┬á0.7	2.518┬á┬▒┬á1.8┬á┬▒┬á0.7┬á┬▒┬á0.9	2.535┬á┬▒┬á1.3┬á┬▒┬á0.5┬á┬▒┬á0.9
0.128ΓÇô0.145	2.206┬á┬▒┬á1.9┬á┬▒┬á0.7┬á┬▒┬á0.7	2.204┬á┬▒┬á1.9┬á┬▒┬á0.7┬á┬▒┬á1.4	2.175┬á┬▒┬á1.7┬á┬▒┬á0.7┬á┬▒┬á0.5	2.170┬á┬▒┬á1.7┬á┬▒┬á0.7┬á┬▒┬á0.5	2.160┬á┬▒┬á1.7┬á┬▒┬á0.7┬á┬▒┬á0.8	2.173┬á┬▒┬á1.3┬á┬▒┬á0.5┬á┬▒┬á0.9
0.145ΓÇô0.165	1.830┬á┬▒┬á1.9┬á┬▒┬á0.7┬á┬▒┬á0.7	1.799┬á┬▒┬á1.9┬á┬▒┬á0.8┬á┬▒┬á1.4	1.846┬á┬▒┬á1.7┬á┬▒┬á0.7┬á┬▒┬á0.6	1.850┬á┬▒┬á1.7┬á┬▒┬á0.7┬á┬▒┬á0.6	1.836┬á┬▒┬á1.7┬á┬▒┬á0.7┬á┬▒┬á0.8	1.811┬á┬▒┬á1.3┬á┬▒┬á0.5┬á┬▒┬á0.9
0.165ΓÇô0.189	1.545┬á┬▒┬á1.9┬á┬▒┬á0.7┬á┬▒┬á0.8	1.519┬á┬▒┬á1.9┬á┬▒┬á0.8┬á┬▒┬á1.4	1.535┬á┬▒┬á1.7┬á┬▒┬á0.7┬á┬▒┬á0.5	1.538┬á┬▒┬á1.7┬á┬▒┬á0.7┬á┬▒┬á0.5	1.497┬á┬▒┬á1.7┬á┬▒┬á0.7┬á┬▒┬á0.8	1.497┬á┬▒┬á1.3┬á┬▒┬á0.5┬á┬▒┬á1.0
0.189ΓÇô0.219	1.235┬á┬▒┬á1.9┬á┬▒┬á1.1┬á┬▒┬á1.0	1.185┬á┬▒┬á1.9┬á┬▒┬á1.2┬á┬▒┬á1.5	1.292┬á┬▒┬á1.7┬á┬▒┬á0.7┬á┬▒┬á0.6	1.292┬á┬▒┬á1.7┬á┬▒┬á0.7┬á┬▒┬á0.6	1.240┬á┬▒┬á1.7┬á┬▒┬á0.7┬á┬▒┬á0.8	1.214┬á┬▒┬á1.3┬á┬▒┬á0.6┬á┬▒┬á0.9
0.219ΓÇô0.258	1.008┬á┬▒┬á1.8┬á┬▒┬á0.7┬á┬▒┬á0.9	0.949┬á┬▒┬á1.8┬á┬▒┬á0.7┬á┬▒┬á1.5	1.003┬á┬▒┬á1.7┬á┬▒┬á0.7┬á┬▒┬á0.6	1.001┬á┬▒┬á1.7┬á┬▒┬á0.7┬á┬▒┬á0.6	0.944┬á┬▒┬á1.7┬á┬▒┬á0.7┬á┬▒┬á0.9	0.943┬á┬▒┬á1.2┬á┬▒┬á0.5┬á┬▒┬á1.0
0.258ΓÇô0.312	0.767┬á┬▒┬á1.8┬á┬▒┬á0.7┬á┬▒┬á0.9	0.707┬á┬▒┬á1.8┬á┬▒┬á0.8┬á┬▒┬á2.2	0.772┬á┬▒┬á1.6┬á┬▒┬á0.7┬á┬▒┬á0.7	0.771┬á┬▒┬á1.6┬á┬▒┬á0.7┬á┬▒┬á0.7	0.702┬á┬▒┬á1.6┬á┬▒┬á0.7┬á┬▒┬á1.9	0.697┬á┬▒┬á1.2┬á┬▒┬á0.5┬á┬▒┬á1.7
0.312ΓÇô0.391	0.545┬á┬▒┬á1.8┬á┬▒┬á0.8┬á┬▒┬á0.9	0.488┬á┬▒┬á1.8┬á┬▒┬á0.9┬á┬▒┬á2.2	0.530┬á┬▒┬á1.6┬á┬▒┬á0.7┬á┬▒┬á0.6	0.531┬á┬▒┬á1.6┬á┬▒┬á0.7┬á┬▒┬á0.6	0.472┬á┬▒┬á1.6┬á┬▒┬á0.7┬á┬▒┬á1.8	0.477┬á┬▒┬á1.2┬á┬▒┬á0.5┬á┬▒┬á1.7
0.391ΓÇô0.524	0.337┬á┬▒┬á1.8┬á┬▒┬á0.7┬á┬▒┬á1.0	0.299┬á┬▒┬á1.8┬á┬▒┬á0.7┬á┬▒┬á2.2	0.336┬á┬▒┬á1.6┬á┬▒┬á0.6┬á┬▒┬á1.1	0.335┬á┬▒┬á1.6┬á┬▒┬á0.6┬á┬▒┬á1.1	0.293┬á┬▒┬á1.6┬á┬▒┬á0.6┬á┬▒┬á2.0	0.295┬á┬▒┬á1.2┬á┬▒┬á0.5┬á┬▒┬á1.7
0.524ΓÇô0.695	0.201┬á┬▒┬á2.0┬á┬▒┬á0.8┬á┬▒┬á1.8	0.183┬á┬▒┬á2.0┬á┬▒┬á0.8┬á┬▒┬á2.7	0.194┬á┬▒┬á1.8┬á┬▒┬á0.8┬á┬▒┬á1.9	0.193┬á┬▒┬á1.8┬á┬▒┬á0.8┬á┬▒┬á1.9	0.170┬á┬▒┬á1.8┬á┬▒┬á0.8┬á┬▒┬á2.5	0.176┬á┬▒┬á1.4┬á┬▒┬á0.6┬á┬▒┬á1.9
0.695ΓÇô0.918	0.105┬á┬▒┬á2.5┬á┬▒┬á1.0┬á┬▒┬á1.5	0.0978┬á┬▒┬á2.5┬á┬▒┬á1.0┬á┬▒┬á2.5	0.113┬á┬▒┬á2.2┬á┬▒┬á1.0┬á┬▒┬á2.1	0.112┬á┬▒┬á2.2┬á┬▒┬á1.0┬á┬▒┬á2.1	0.102┬á┬▒┬á2.2┬á┬▒┬á1.0┬á┬▒┬á2.7	0.101┬á┬▒┬á1.6┬á┬▒┬á0.7┬á┬▒┬á2.0
0.918ΓÇô1.153	0.0647┬á┬▒┬á3.2┬á┬▒┬á1.3┬á┬▒┬á1.9	0.0623┬á┬▒┬á3.2┬á┬▒┬á1.4┬á┬▒┬á2.7	0.0613┬á┬▒┬á3.0┬á┬▒┬á1.3┬á┬▒┬á2.9	0.0609┬á┬▒┬á3.0┬á┬▒┬á1.3┬á┬▒┬á2.9	0.0569┬á┬▒┬á3.0┬á┬▒┬á1.3┬á┬▒┬á3.4	0.0598┬á┬▒┬á2.2┬á┬▒┬á0.9┬á┬▒┬á2.3
1.153ΓÇô1.496	0.0342┬á┬▒┬á3.9┬á┬▒┬á2.7┬á┬▒┬á2.9	0.0330┬á┬▒┬á3.9┬á┬▒┬á2.8┬á┬▒┬á3.6	0.0333┬á┬▒┬á3.2┬á┬▒┬á1.7┬á┬▒┬á4.2	0.0328┬á┬▒┬á3.2┬á┬▒┬á1.7┬á┬▒┬á4.2	0.0315┬á┬▒┬á3.2┬á┬▒┬á1.7┬á┬▒┬á4.8	0.0330┬á┬▒┬á2.5┬á┬▒┬á1.5┬á┬▒┬á3.2
1.496ΓÇô1.947	0.0184┬á┬▒┬á4.7┬á┬▒┬á2.2┬á┬▒┬á3.0	0.0181┬á┬▒┬á4.7┬á┬▒┬á2.2┬á┬▒┬á3.6	0.0169┬á┬▒┬á4.1┬á┬▒┬á2.1┬á┬▒┬á3.3	0.0167┬á┬▒┬á4.1┬á┬▒┬á2.1┬á┬▒┬á3.3	0.0160┬á┬▒┬á4.1┬á┬▒┬á2.1┬á┬▒┬á4.0	0.0170┬á┬▒┬á3.1┬á┬▒┬á1.5┬á┬▒┬á3.2
1.947ΓÇô2.522	0.00907┬á┬▒┬á6.1┬á┬▒┬á3.0┬á┬▒┬á3.7	0.00885┬á┬▒┬á6.1┬á┬▒┬á3.1┬á┬▒┬á4.3	0.00989┬á┬▒┬á4.7┬á┬▒┬á2.2┬á┬▒┬á2.9	0.00975┬á┬▒┬á4.7┬á┬▒┬á2.2┬á┬▒┬á2.9	0.00950┬á┬▒┬á4.7┬á┬▒┬á2.2┬á┬▒┬á3.6	0.00939┬á┬▒┬á3.7┬á┬▒┬á1.8┬á┬▒┬á3.2
2.522ΓÇô3.277	0.00454┬á┬▒┬á7.5┬á┬▒┬á4.6┬á┬▒┬á3.1	0.00445┬á┬▒┬á7.5┬á┬▒┬á4.7┬á┬▒┬á3.8	0.00447┬á┬▒┬á6.1┬á┬▒┬á2.7┬á┬▒┬á4.2	0.00441┬á┬▒┬á6.1┬á┬▒┬á2.7┬á┬▒┬á4.2	0.00430┬á┬▒┬á6.1┬á┬▒┬á2.7┬á┬▒┬á4.8	0.00446┬á┬▒┬á4.7┬á┬▒┬á2.4┬á┬▒┬á3.4
3.277ΓÇô5.000	0.00252┬á┬▒┬á6.3┬á┬▒┬á2.8┬á┬▒┬á4.0	0.00252┬á┬▒┬á6.3┬á┬▒┬á2.8┬á┬▒┬á4.6	0.00220┬á┬▒┬á5.7┬á┬▒┬á2.6┬á┬▒┬á3.8	0.00219┬á┬▒┬á5.7┬á┬▒┬á2.6┬á┬▒┬á3.8	0.00214┬á┬▒┬á5.7┬á┬▒┬á2.6┬á┬▒┬á4.4	0.00232┬á┬▒┬á4.2┬á┬▒┬á1.9┬á┬▒┬á3.3
5.000ΓÇô10.000	0.000525┬á┬▒┬á8.6┬á┬▒┬á3.9┬á┬▒┬á3.4	0.000510┬á┬▒┬á8.6┬á┬▒┬á3.9┬á┬▒┬á4.0	0.000585┬á┬▒┬á6.4┬á┬▒┬á2.9┬á┬▒┬á4.0	0.000577┬á┬▒┬á6.4┬á┬▒┬á2.9┬á┬▒┬á4.0	0.000545┬á┬▒┬á6.4┬á┬▒┬á2.9┬á┬▒┬á4.6	0.000542┬á┬▒┬á5.1┬á┬▒┬á2.3┬á┬▒┬á3.3

**Table 6:** The values of $(1/\sigma )\,{\mathrm {d}}\sigma /{\mathrm {d}}\phi ^*_{\eta }$ in each bin of $\phi ^*_{\eta }$ for the electron and muon channels separately (for various particle-level definitions) and for the Born-level combination in the kinematic region $46\ ~{\mathrm {GeV}}\le m_{\ell \ell }< 66\ ~{\mathrm {GeV}},\ 0.8 \le |y_{\ell \ell }| < 1.6$. The associated statistical and systematic (both uncorrelated and correlated between bins of $\phi ^*_{\eta }$) are provided in percentage form

Bin	$(1/\sigma )\,d\sigma /d\phi ^*_{\eta }$ ┬▒ Statistical [%] ┬▒ Uncorrelated systematic [%] ┬▒ Correlated systematic [%]					
	Electron channel		Muon channel			Combination
	Dressed	Born	Bare	Dressed	Born	Born
0.000ΓÇô0.004	7.182┬á┬▒┬á2.7┬á┬▒┬á1.1┬á┬▒┬á5.3	7.754┬á┬▒┬á2.7┬á┬▒┬á1.1┬á┬▒┬á5.3	6.713┬á┬▒┬á1.9┬á┬▒┬á0.8┬á┬▒┬á4.7	6.704┬á┬▒┬á1.9┬á┬▒┬á0.8┬á┬▒┬á4.7	7.109┬á┬▒┬á1.9┬á┬▒┬á0.8┬á┬▒┬á5.0	7.311┬á┬▒┬á1.5┬á┬▒┬á0.6┬á┬▒┬á4.9
0.004ΓÇô0.008	7.048┬á┬▒┬á2.6┬á┬▒┬á1.1┬á┬▒┬á2.5	7.465┬á┬▒┬á2.6┬á┬▒┬á1.1┬á┬▒┬á2.7	6.917┬á┬▒┬á1.9┬á┬▒┬á0.8┬á┬▒┬á1.9	6.884┬á┬▒┬á1.9┬á┬▒┬á0.8┬á┬▒┬á1.9	7.255┬á┬▒┬á1.9┬á┬▒┬á0.8┬á┬▒┬á2.5	7.349┬á┬▒┬á1.5┬á┬▒┬á0.6┬á┬▒┬á2.3
0.008ΓÇô0.012	6.842┬á┬▒┬á2.6┬á┬▒┬á1.1┬á┬▒┬á1.7	7.254┬á┬▒┬á2.6┬á┬▒┬á1.1┬á┬▒┬á2.0	6.898┬á┬▒┬á1.9┬á┬▒┬á0.8┬á┬▒┬á1.3	6.851┬á┬▒┬á1.9┬á┬▒┬á0.8┬á┬▒┬á1.3	7.247┬á┬▒┬á1.9┬á┬▒┬á0.8┬á┬▒┬á2.0	7.270┬á┬▒┬á1.5┬á┬▒┬á0.6┬á┬▒┬á1.7
0.012ΓÇô0.016	6.408┬á┬▒┬á2.7┬á┬▒┬á1.1┬á┬▒┬á1.1	6.812┬á┬▒┬á2.7┬á┬▒┬á1.2┬á┬▒┬á1.6	6.758┬á┬▒┬á1.9┬á┬▒┬á0.8┬á┬▒┬á1.0	6.757┬á┬▒┬á1.9┬á┬▒┬á0.8┬á┬▒┬á1.0	7.249┬á┬▒┬á1.9┬á┬▒┬á0.8┬á┬▒┬á1.9	7.123┬á┬▒┬á1.6┬á┬▒┬á0.6┬á┬▒┬á1.5
0.016ΓÇô0.020	6.302┬á┬▒┬á2.7┬á┬▒┬á1.1┬á┬▒┬á1.4	6.619┬á┬▒┬á2.7┬á┬▒┬á1.1┬á┬▒┬á1.8	6.625┬á┬▒┬á2.0┬á┬▒┬á0.8┬á┬▒┬á1.1	6.630┬á┬▒┬á2.0┬á┬▒┬á0.8┬á┬▒┬á1.1	7.000┬á┬▒┬á2.0┬á┬▒┬á0.8┬á┬▒┬á1.9	6.872┬á┬▒┬á1.6┬á┬▒┬á0.7┬á┬▒┬á1.4
0.020ΓÇô0.024	6.328┬á┬▒┬á2.7┬á┬▒┬á1.1┬á┬▒┬á1.1	6.654┬á┬▒┬á2.7┬á┬▒┬á1.1┬á┬▒┬á1.6	6.326┬á┬▒┬á2.0┬á┬▒┬á0.8┬á┬▒┬á0.9	6.334┬á┬▒┬á2.0┬á┬▒┬á0.8┬á┬▒┬á0.9	6.722┬á┬▒┬á2.0┬á┬▒┬á0.8┬á┬▒┬á1.8	6.725┬á┬▒┬á1.6┬á┬▒┬á0.7┬á┬▒┬á1.4
0.024ΓÇô0.029	6.279┬á┬▒┬á2.4┬á┬▒┬á0.9┬á┬▒┬á0.9	6.570┬á┬▒┬á2.4┬á┬▒┬á1.0┬á┬▒┬á1.4	6.161┬á┬▒┬á1.8┬á┬▒┬á0.7┬á┬▒┬á0.8	6.141┬á┬▒┬á1.8┬á┬▒┬á0.7┬á┬▒┬á0.8	6.497┬á┬▒┬á1.8┬á┬▒┬á0.7┬á┬▒┬á1.7	6.535┬á┬▒┬á1.4┬á┬▒┬á0.6┬á┬▒┬á1.3
0.029ΓÇô0.034	6.047┬á┬▒┬á2.4┬á┬▒┬á1.0┬á┬▒┬á0.8	6.369┬á┬▒┬á2.4┬á┬▒┬á1.0┬á┬▒┬á1.4	5.931┬á┬▒┬á1.9┬á┬▒┬á0.8┬á┬▒┬á0.7	5.944┬á┬▒┬á1.9┬á┬▒┬á0.8┬á┬▒┬á0.7	6.311┬á┬▒┬á1.9┬á┬▒┬á0.8┬á┬▒┬á1.6	6.344┬á┬▒┬á1.5┬á┬▒┬á0.6┬á┬▒┬á1.2
0.034ΓÇô0.039	5.803┬á┬▒┬á2.6┬á┬▒┬á1.1┬á┬▒┬á1.1	6.074┬á┬▒┬á2.6┬á┬▒┬á1.1┬á┬▒┬á1.5	5.684┬á┬▒┬á1.9┬á┬▒┬á0.8┬á┬▒┬á0.7	5.664┬á┬▒┬á1.9┬á┬▒┬á0.8┬á┬▒┬á0.7	6.004┬á┬▒┬á1.9┬á┬▒┬á0.8┬á┬▒┬á1.6	6.049┬á┬▒┬á1.5┬á┬▒┬á0.6┬á┬▒┬á1.2
0.039ΓÇô0.045	5.295┬á┬▒┬á2.4┬á┬▒┬á0.9┬á┬▒┬á0.6	5.522┬á┬▒┬á2.4┬á┬▒┬á1.0┬á┬▒┬á1.3	5.417┬á┬▒┬á1.8┬á┬▒┬á0.7┬á┬▒┬á0.7	5.413┬á┬▒┬á1.8┬á┬▒┬á0.7┬á┬▒┬á0.7	5.695┬á┬▒┬á1.8┬á┬▒┬á0.7┬á┬▒┬á1.6	5.648┬á┬▒┬á1.4┬á┬▒┬á0.6┬á┬▒┬á1.2
0.045ΓÇô0.051	5.149┬á┬▒┬á2.4┬á┬▒┬á1.0┬á┬▒┬á0.9	5.351┬á┬▒┬á2.4┬á┬▒┬á1.0┬á┬▒┬á1.5	5.158┬á┬▒┬á1.8┬á┬▒┬á0.7┬á┬▒┬á1.0	5.189┬á┬▒┬á1.8┬á┬▒┬á0.7┬á┬▒┬á1.0	5.398┬á┬▒┬á1.8┬á┬▒┬á0.7┬á┬▒┬á1.8	5.373┬á┬▒┬á1.5┬á┬▒┬á0.6┬á┬▒┬á1.2
0.051ΓÇô0.057	4.906┬á┬▒┬á2.5┬á┬▒┬á1.0┬á┬▒┬á0.7	5.112┬á┬▒┬á2.5┬á┬▒┬á1.0┬á┬▒┬á1.3	5.115┬á┬▒┬á1.8┬á┬▒┬á0.7┬á┬▒┬á0.8	5.139┬á┬▒┬á1.8┬á┬▒┬á0.7┬á┬▒┬á0.8	5.394┬á┬▒┬á1.8┬á┬▒┬á0.7┬á┬▒┬á1.7	5.308┬á┬▒┬á1.5┬á┬▒┬á0.6┬á┬▒┬á1.2
0.057ΓÇô0.064	4.396┬á┬▒┬á2.4┬á┬▒┬á0.9┬á┬▒┬á0.7	4.555┬á┬▒┬á2.4┬á┬▒┬á1.0┬á┬▒┬á1.3	4.535┬á┬▒┬á1.8┬á┬▒┬á0.7┬á┬▒┬á0.8	4.569┬á┬▒┬á1.8┬á┬▒┬á0.7┬á┬▒┬á0.8	4.775┬á┬▒┬á1.8┬á┬▒┬á0.7┬á┬▒┬á1.4	4.698┬á┬▒┬á1.4┬á┬▒┬á0.6┬á┬▒┬á1.1
0.064ΓÇô0.072	4.289┬á┬▒┬á2.3┬á┬▒┬á0.9┬á┬▒┬á1.0	4.427┬á┬▒┬á2.3┬á┬▒┬á1.0┬á┬▒┬á1.5	4.180┬á┬▒┬á1.7┬á┬▒┬á0.7┬á┬▒┬á0.7	4.198┬á┬▒┬á1.7┬á┬▒┬á0.7┬á┬▒┬á0.7	4.360┬á┬▒┬á1.7┬á┬▒┬á0.7┬á┬▒┬á1.4	4.383┬á┬▒┬á1.4┬á┬▒┬á0.6┬á┬▒┬á1.1
0.072ΓÇô0.081	3.884┬á┬▒┬á2.3┬á┬▒┬á0.9┬á┬▒┬á0.8	3.993┬á┬▒┬á2.3┬á┬▒┬á0.9┬á┬▒┬á1.4	4.079┬á┬▒┬á1.6┬á┬▒┬á0.7┬á┬▒┬á0.7	4.110┬á┬▒┬á1.6┬á┬▒┬á0.7┬á┬▒┬á0.7	4.273┬á┬▒┬á1.6┬á┬▒┬á0.7┬á┬▒┬á1.3	4.182┬á┬▒┬á1.3┬á┬▒┬á0.5┬á┬▒┬á1.1
0.081ΓÇô0.091	3.645┬á┬▒┬á2.2┬á┬▒┬á0.9┬á┬▒┬á0.8	3.749┬á┬▒┬á2.2┬á┬▒┬á0.9┬á┬▒┬á1.4	3.465┬á┬▒┬á1.7┬á┬▒┬á0.7┬á┬▒┬á0.4	3.486┬á┬▒┬á1.7┬á┬▒┬á0.7┬á┬▒┬á0.4	3.611┬á┬▒┬á1.7┬á┬▒┬á0.7┬á┬▒┬á1.2	3.688┬á┬▒┬á1.3┬á┬▒┬á0.5┬á┬▒┬á1.0
0.091ΓÇô0.102	3.172┬á┬▒┬á2.3┬á┬▒┬á0.9┬á┬▒┬á0.7	3.241┬á┬▒┬á2.3┬á┬▒┬á1.0┬á┬▒┬á1.3	3.153┬á┬▒┬á1.7┬á┬▒┬á0.7┬á┬▒┬á0.5	3.165┬á┬▒┬á1.7┬á┬▒┬á0.7┬á┬▒┬á0.5	3.232┬á┬▒┬á1.7┬á┬▒┬á0.7┬á┬▒┬á1.2	3.256┬á┬▒┬á1.3┬á┬▒┬á0.6┬á┬▒┬á1.0
0.102ΓÇô0.114	2.869┬á┬▒┬á2.3┬á┬▒┬á0.9┬á┬▒┬á0.7	2.927┬á┬▒┬á2.3┬á┬▒┬á1.0┬á┬▒┬á1.4	2.795┬á┬▒┬á1.7┬á┬▒┬á0.7┬á┬▒┬á0.5	2.802┬á┬▒┬á1.7┬á┬▒┬á0.7┬á┬▒┬á0.5	2.850┬á┬▒┬á1.7┬á┬▒┬á0.7┬á┬▒┬á1.2	2.893┬á┬▒┬á1.4┬á┬▒┬á0.6┬á┬▒┬á1.0
0.114ΓÇô0.128	2.520┬á┬▒┬á2.3┬á┬▒┬á1.0┬á┬▒┬á0.8	2.540┬á┬▒┬á2.3┬á┬▒┬á1.0┬á┬▒┬á1.4	2.538┬á┬▒┬á1.6┬á┬▒┬á0.7┬á┬▒┬á0.5	2.550┬á┬▒┬á1.6┬á┬▒┬á0.7┬á┬▒┬á0.5	2.585┬á┬▒┬á1.6┬á┬▒┬á0.7┬á┬▒┬á0.6	2.585┬á┬▒┬á1.3┬á┬▒┬á0.6┬á┬▒┬á0.7
0.128ΓÇô0.145	2.092┬á┬▒┬á2.2┬á┬▒┬á0.9┬á┬▒┬á0.8	2.091┬á┬▒┬á2.2┬á┬▒┬á1.0┬á┬▒┬á1.4	2.158┬á┬▒┬á1.6┬á┬▒┬á0.6┬á┬▒┬á0.6	2.154┬á┬▒┬á1.6┬á┬▒┬á0.6┬á┬▒┬á0.6	2.151┬á┬▒┬á1.6┬á┬▒┬á0.6┬á┬▒┬á0.7	2.137┬á┬▒┬á1.3┬á┬▒┬á0.5┬á┬▒┬á0.8
0.145ΓÇô0.165	1.806┬á┬▒┬á2.3┬á┬▒┬á0.9┬á┬▒┬á0.8	1.768┬á┬▒┬á2.3┬á┬▒┬á0.9┬á┬▒┬á1.4	1.891┬á┬▒┬á1.6┬á┬▒┬á0.6┬á┬▒┬á0.6	1.889┬á┬▒┬á1.6┬á┬▒┬á0.6┬á┬▒┬á0.6	1.868┬á┬▒┬á1.6┬á┬▒┬á0.6┬á┬▒┬á0.7	1.841┬á┬▒┬á1.3┬á┬▒┬á0.5┬á┬▒┬á0.8
0.165ΓÇô0.189	1.462┬á┬▒┬á2.3┬á┬▒┬á0.9┬á┬▒┬á0.7	1.419┬á┬▒┬á2.3┬á┬▒┬á1.0┬á┬▒┬á1.4	1.497┬á┬▒┬á1.6┬á┬▒┬á0.7┬á┬▒┬á0.5	1.496┬á┬▒┬á1.6┬á┬▒┬á0.7┬á┬▒┬á0.5	1.458┬á┬▒┬á1.6┬á┬▒┬á0.7┬á┬▒┬á0.6	1.453┬á┬▒┬á1.3┬á┬▒┬á0.5┬á┬▒┬á0.7
0.189ΓÇô0.219	1.216┬á┬▒┬á2.2┬á┬▒┬á1.1┬á┬▒┬á0.7	1.162┬á┬▒┬á2.2┬á┬▒┬á1.1┬á┬▒┬á1.4	1.268┬á┬▒┬á1.6┬á┬▒┬á0.6┬á┬▒┬á0.6	1.268┬á┬▒┬á1.6┬á┬▒┬á0.6┬á┬▒┬á0.6	1.209┬á┬▒┬á1.6┬á┬▒┬á0.6┬á┬▒┬á0.7	1.199┬á┬▒┬á1.3┬á┬▒┬á0.6┬á┬▒┬á0.7
0.219ΓÇô0.258	0.989┬á┬▒┬á2.2┬á┬▒┬á0.8┬á┬▒┬á0.8	0.937┬á┬▒┬á2.2┬á┬▒┬á0.9┬á┬▒┬á1.4	0.982┬á┬▒┬á1.6┬á┬▒┬á0.6┬á┬▒┬á0.6	0.991┬á┬▒┬á1.6┬á┬▒┬á0.6┬á┬▒┬á0.6	0.928┬á┬▒┬á1.6┬á┬▒┬á0.6┬á┬▒┬á0.7	0.937┬á┬▒┬á1.3┬á┬▒┬á0.5┬á┬▒┬á0.8
0.258ΓÇô0.312	0.738┬á┬▒┬á2.1┬á┬▒┬á0.9┬á┬▒┬á0.9	0.669┬á┬▒┬á2.1┬á┬▒┬á1.0┬á┬▒┬á2.2	0.755┬á┬▒┬á1.5┬á┬▒┬á0.6┬á┬▒┬á0.5	0.756┬á┬▒┬á1.5┬á┬▒┬á0.6┬á┬▒┬á0.5	0.686┬á┬▒┬á1.5┬á┬▒┬á0.6┬á┬▒┬á2.2	0.693┬á┬▒┬á1.2┬á┬▒┬á0.5┬á┬▒┬á1.7
0.312ΓÇô0.391	0.554┬á┬▒┬á2.0┬á┬▒┬á0.9┬á┬▒┬á1.2	0.499┬á┬▒┬á2.0┬á┬▒┬á1.0┬á┬▒┬á2.4	0.526┬á┬▒┬á1.5┬á┬▒┬á0.6┬á┬▒┬á1.2	0.524┬á┬▒┬á1.5┬á┬▒┬á0.6┬á┬▒┬á1.2	0.465┬á┬▒┬á1.5┬á┬▒┬á0.6┬á┬▒┬á2.4	0.490┬á┬▒┬á1.2┬á┬▒┬á0.5┬á┬▒┬á1.8
0.391ΓÇô0.524	0.325┬á┬▒┬á2.1┬á┬▒┬á0.8┬á┬▒┬á1.3	0.288┬á┬▒┬á2.1┬á┬▒┬á0.9┬á┬▒┬á2.4	0.331┬á┬▒┬á1.5┬á┬▒┬á0.7┬á┬▒┬á1.6	0.329┬á┬▒┬á1.5┬á┬▒┬á0.7┬á┬▒┬á1.6	0.291┬á┬▒┬á1.5┬á┬▒┬á0.7┬á┬▒┬á2.6	0.300┬á┬▒┬á1.2┬á┬▒┬á0.6┬á┬▒┬á1.9
0.524ΓÇô0.695	0.197┬á┬▒┬á2.4┬á┬▒┬á1.3┬á┬▒┬á1.9	0.179┬á┬▒┬á2.4┬á┬▒┬á1.3┬á┬▒┬á2.8	0.187┬á┬▒┬á1.7┬á┬▒┬á0.7┬á┬▒┬á2.2	0.186┬á┬▒┬á1.7┬á┬▒┬á0.7┬á┬▒┬á2.2	0.163┬á┬▒┬á1.7┬á┬▒┬á0.7┬á┬▒┬á3.1	0.176┬á┬▒┬á1.4┬á┬▒┬á0.6┬á┬▒┬á2.1
0.695ΓÇô0.918	0.102┬á┬▒┬á2.9┬á┬▒┬á1.8┬á┬▒┬á1.9	0.0961┬á┬▒┬á2.9┬á┬▒┬á1.8┬á┬▒┬á2.8	0.101┬á┬▒┬á2.1┬á┬▒┬á0.9┬á┬▒┬á2.5	0.0998┬á┬▒┬á2.1┬á┬▒┬á0.9┬á┬▒┬á2.5	0.0921┬á┬▒┬á2.1┬á┬▒┬á0.9┬á┬▒┬á3.3	0.0978┬á┬▒┬á1.7┬á┬▒┬á0.8┬á┬▒┬á2.2
0.918ΓÇô1.153	0.0605┬á┬▒┬á3.8┬á┬▒┬á1.8┬á┬▒┬á2.3	0.0584┬á┬▒┬á3.8┬á┬▒┬á1.9┬á┬▒┬á3.1	0.0594┬á┬▒┬á2.6┬á┬▒┬á1.1┬á┬▒┬á3.4	0.0588┬á┬▒┬á2.6┬á┬▒┬á1.1┬á┬▒┬á3.4	0.0547┬á┬▒┬á2.6┬á┬▒┬á1.1┬á┬▒┬á4.0	0.0594┬á┬▒┬á2.1┬á┬▒┬á1.0┬á┬▒┬á2.5
1.153ΓÇô1.496	0.0352┬á┬▒┬á4.2┬á┬▒┬á3.4┬á┬▒┬á2.6	0.0345┬á┬▒┬á4.2┬á┬▒┬á3.4┬á┬▒┬á3.4	0.0315┬á┬▒┬á3.1┬á┬▒┬á1.6┬á┬▒┬á2.7	0.0310┬á┬▒┬á3.1┬á┬▒┬á1.6┬á┬▒┬á2.7	0.0300┬á┬▒┬á3.1┬á┬▒┬á1.6┬á┬▒┬á3.2	0.0327┬á┬▒┬á2.5┬á┬▒┬á1.5┬á┬▒┬á2.4
1.496ΓÇô1.947	0.0174┬á┬▒┬á5.2┬á┬▒┬á3.3┬á┬▒┬á3.7	0.0171┬á┬▒┬á5.2┬á┬▒┬á3.4┬á┬▒┬á4.3	0.0177┬á┬▒┬á3.8┬á┬▒┬á1.8┬á┬▒┬á2.2	0.0175┬á┬▒┬á3.8┬á┬▒┬á1.8┬á┬▒┬á2.2	0.0167┬á┬▒┬á3.8┬á┬▒┬á1.8┬á┬▒┬á2.8	0.0175┬á┬▒┬á3.0┬á┬▒┬á1.6┬á┬▒┬á2.4
1.947ΓÇô2.522	0.00950┬á┬▒┬á6.2┬á┬▒┬á3.6┬á┬▒┬á3.9	0.00936┬á┬▒┬á6.2┬á┬▒┬á3.7┬á┬▒┬á4.5	0.00938┬á┬▒┬á4.4┬á┬▒┬á2.0┬á┬▒┬á2.4	0.00923┬á┬▒┬á4.4┬á┬▒┬á2.0┬á┬▒┬á2.4	0.00905┬á┬▒┬á4.4┬á┬▒┬á2.0┬á┬▒┬á2.9	0.00953┬á┬▒┬á3.5┬á┬▒┬á1.8┬á┬▒┬á2.4
2.522ΓÇô3.277	0.00567┬á┬▒┬á6.3┬á┬▒┬á3.9┬á┬▒┬á11	0.00574┬á┬▒┬á6.3┬á┬▒┬á3.9┬á┬▒┬á11	0.00493┬á┬▒┬á5.4┬á┬▒┬á3.3┬á┬▒┬á2.0	0.00494┬á┬▒┬á5.4┬á┬▒┬á3.3┬á┬▒┬á2.0	0.00480┬á┬▒┬á5.4┬á┬▒┬á3.3┬á┬▒┬á2.6	0.00519┬á┬▒┬á4.1┬á┬▒┬á2.5┬á┬▒┬á3.3
3.277ΓÇô5.000	0.00208┬á┬▒┬á7.3┬á┬▒┬á4.5┬á┬▒┬á4.1	0.00204┬á┬▒┬á7.3┬á┬▒┬á4.5┬á┬▒┬á4.6	0.00206┬á┬▒┬á5.5┬á┬▒┬á3.4┬á┬▒┬á3.6	0.00204┬á┬▒┬á5.5┬á┬▒┬á3.4┬á┬▒┬á3.6	0.00200┬á┬▒┬á5.5┬á┬▒┬á3.4┬á┬▒┬á3.9	0.00211┬á┬▒┬á4.3┬á┬▒┬á2.7┬á┬▒┬á2.7
5.000ΓÇô10.000	0.000603┬á┬▒┬á7.9┬á┬▒┬á4.4┬á┬▒┬á3.3	0.000599┬á┬▒┬á7.9┬á┬▒┬á4.4┬á┬▒┬á3.9	0.000536┬á┬▒┬á6.4┬á┬▒┬á3.8┬á┬▒┬á3.4	0.000529┬á┬▒┬á6.4┬á┬▒┬á3.8┬á┬▒┬á3.4	0.000521┬á┬▒┬á6.4┬á┬▒┬á3.8┬á┬▒┬á3.8	0.000577┬á┬▒┬á4.9┬á┬▒┬á2.9┬á┬▒┬á2.7

**Table 7:** The values of $(1/\sigma )\,{\mathrm {d}}\sigma /{\mathrm {d}}\phi ^*_{\eta }$ in each bin of $\phi ^*_{\eta }$ for the electron and muon channels separately (for various particle-level definitions) and for the Born-level combination in the kinematic region $46\ ~{\mathrm {GeV}}\le m_{\ell \ell }< 66\ ~{\mathrm {GeV}},\ 1.6 \le |y_{\ell \ell }| < 2.4$. The associated statistical and systematic (both uncorrelated and correlated between bins of $\phi ^*_{\eta }$) are provided in percentage form

Bin	$(1/\sigma )\,d\sigma /d\phi ^*_{\eta }$ ┬▒ Statistical [%] ┬▒ Uncorrelated systematic [%] ┬▒ Correlated systematic [%]					
	Electron channel		Muon channel			Combination
	Dressed	Born	Bare	Dressed	Born	Born
0.000ΓÇô0.004	6.724┬á┬▒┬á3.7┬á┬▒┬á1.7┬á┬▒┬á4.0	7.096┬á┬▒┬á3.7┬á┬▒┬á1.7┬á┬▒┬á4.2	6.844┬á┬▒┬á2.6┬á┬▒┬á1.1┬á┬▒┬á3.2	6.783┬á┬▒┬á2.6┬á┬▒┬á1.1┬á┬▒┬á3.2	7.249┬á┬▒┬á2.6┬á┬▒┬á1.1┬á┬▒┬á3.3	7.260┬á┬▒┬á2.1┬á┬▒┬á0.9┬á┬▒┬á3.5
0.004ΓÇô0.008	6.620┬á┬▒┬á3.7┬á┬▒┬á1.8┬á┬▒┬á1.9	6.958┬á┬▒┬á3.7┬á┬▒┬á1.8┬á┬▒┬á2.2	6.899┬á┬▒┬á2.4┬á┬▒┬á1.0┬á┬▒┬á1.5	6.752┬á┬▒┬á2.4┬á┬▒┬á1.0┬á┬▒┬á1.5	7.155┬á┬▒┬á2.4┬á┬▒┬á1.0┬á┬▒┬á1.7	7.129┬á┬▒┬á2.0┬á┬▒┬á0.9┬á┬▒┬á1.7
0.008ΓÇô0.012	6.546┬á┬▒┬á3.6┬á┬▒┬á1.8┬á┬▒┬á1.2	6.826┬á┬▒┬á3.6┬á┬▒┬á1.8┬á┬▒┬á1.7	7.009┬á┬▒┬á2.5┬á┬▒┬á1.0┬á┬▒┬á1.2	6.942┬á┬▒┬á2.5┬á┬▒┬á1.0┬á┬▒┬á1.2	7.338┬á┬▒┬á2.5┬á┬▒┬á1.0┬á┬▒┬á1.4	7.174┬á┬▒┬á2.1┬á┬▒┬á0.9┬á┬▒┬á1.3
0.012ΓÇô0.016	6.493┬á┬▒┬á3.6┬á┬▒┬á1.4┬á┬▒┬á1.6	6.877┬á┬▒┬á3.6┬á┬▒┬á1.5┬á┬▒┬á1.9	7.341┬á┬▒┬á2.5┬á┬▒┬á1.1┬á┬▒┬á0.9	7.206┬á┬▒┬á2.5┬á┬▒┬á1.1┬á┬▒┬á0.9	7.625┬á┬▒┬á2.5┬á┬▒┬á1.1┬á┬▒┬á1.2	7.389┬á┬▒┬á2.0┬á┬▒┬á0.9┬á┬▒┬á1.2
0.016ΓÇô0.020	6.632┬á┬▒┬á3.6┬á┬▒┬á1.5┬á┬▒┬á2.0	6.939┬á┬▒┬á3.6┬á┬▒┬á1.5┬á┬▒┬á2.3	6.958┬á┬▒┬á2.5┬á┬▒┬á1.0┬á┬▒┬á0.9	6.958┬á┬▒┬á2.5┬á┬▒┬á1.0┬á┬▒┬á0.9	7.457┬á┬▒┬á2.5┬á┬▒┬á1.0┬á┬▒┬á1.3	7.249┬á┬▒┬á2.1┬á┬▒┬á0.9┬á┬▒┬á1.2
0.020ΓÇô0.024	6.727┬á┬▒┬á3.6┬á┬▒┬á1.5┬á┬▒┬á0.9	7.004┬á┬▒┬á3.6┬á┬▒┬á1.5┬á┬▒┬á1.5	6.505┬á┬▒┬á2.7┬á┬▒┬á1.1┬á┬▒┬á0.7	6.504┬á┬▒┬á2.7┬á┬▒┬á1.1┬á┬▒┬á0.7	6.724┬á┬▒┬á2.7┬á┬▒┬á1.1┬á┬▒┬á1.1	6.803┬á┬▒┬á2.1┬á┬▒┬á0.9┬á┬▒┬á1.0
0.024ΓÇô0.029	6.531┬á┬▒┬á3.3┬á┬▒┬á1.3┬á┬▒┬á1.1	6.896┬á┬▒┬á3.3┬á┬▒┬á1.4┬á┬▒┬á1.6	6.452┬á┬▒┬á2.4┬á┬▒┬á1.0┬á┬▒┬á0.7	6.378┬á┬▒┬á2.4┬á┬▒┬á1.0┬á┬▒┬á0.7	6.721┬á┬▒┬á2.4┬á┬▒┬á1.0┬á┬▒┬á1.1	6.747┬á┬▒┬á1.9┬á┬▒┬á0.8┬á┬▒┬á1.0
0.029ΓÇô0.034	6.023┬á┬▒┬á3.4┬á┬▒┬á1.4┬á┬▒┬á1.0	6.297┬á┬▒┬á3.4┬á┬▒┬á1.4┬á┬▒┬á1.5	5.969┬á┬▒┬á2.5┬á┬▒┬á1.0┬á┬▒┬á0.7	5.980┬á┬▒┬á2.5┬á┬▒┬á1.0┬á┬▒┬á0.7	6.272┬á┬▒┬á2.5┬á┬▒┬á1.0┬á┬▒┬á1.1	6.261┬á┬▒┬á2.0┬á┬▒┬á0.8┬á┬▒┬á1.0
0.034ΓÇô0.039	6.204┬á┬▒┬á3.3┬á┬▒┬á1.4┬á┬▒┬á0.9	6.499┬á┬▒┬á3.3┬á┬▒┬á1.4┬á┬▒┬á1.5	5.529┬á┬▒┬á2.6┬á┬▒┬á1.1┬á┬▒┬á0.6	5.509┬á┬▒┬á2.6┬á┬▒┬á1.1┬á┬▒┬á0.6	5.840┬á┬▒┬á2.6┬á┬▒┬á1.1┬á┬▒┬á1.0	6.050┬á┬▒┬á2.0┬á┬▒┬á0.8┬á┬▒┬á1.0
0.039ΓÇô0.045	5.461┬á┬▒┬á3.2┬á┬▒┬á1.8┬á┬▒┬á0.9	5.675┬á┬▒┬á3.2┬á┬▒┬á1.8┬á┬▒┬á1.4	5.619┬á┬▒┬á2.3┬á┬▒┬á0.9┬á┬▒┬á0.7	5.625┬á┬▒┬á2.3┬á┬▒┬á0.9┬á┬▒┬á0.7	5.999┬á┬▒┬á2.3┬á┬▒┬á0.9┬á┬▒┬á1.1	5.895┬á┬▒┬á1.9┬á┬▒┬á0.9┬á┬▒┬á1.0
0.045ΓÇô0.051	5.384┬á┬▒┬á3.3┬á┬▒┬á1.8┬á┬▒┬á1.1	5.592┬á┬▒┬á3.3┬á┬▒┬á1.8┬á┬▒┬á1.5	5.153┬á┬▒┬á2.4┬á┬▒┬á1.0┬á┬▒┬á0.8	5.170┬á┬▒┬á2.4┬á┬▒┬á1.0┬á┬▒┬á0.8	5.355┬á┬▒┬á2.4┬á┬▒┬á1.0┬á┬▒┬á1.2	5.418┬á┬▒┬á1.9┬á┬▒┬á0.9┬á┬▒┬á1.0
0.051ΓÇô0.057	5.016┬á┬▒┬á3.4┬á┬▒┬á1.4┬á┬▒┬á1.3	5.256┬á┬▒┬á3.4┬á┬▒┬á1.5┬á┬▒┬á1.7	4.916┬á┬▒┬á2.4┬á┬▒┬á1.0┬á┬▒┬á0.6	4.891┬á┬▒┬á2.4┬á┬▒┬á1.0┬á┬▒┬á0.6	5.109┬á┬▒┬á2.4┬á┬▒┬á1.0┬á┬▒┬á1.0	5.161┬á┬▒┬á2.0┬á┬▒┬á0.8┬á┬▒┬á1.0
0.057ΓÇô0.064	4.760┬á┬▒┬á3.2┬á┬▒┬á1.4┬á┬▒┬á0.8	4.923┬á┬▒┬á3.2┬á┬▒┬á1.4┬á┬▒┬á1.4	4.848┬á┬▒┬á2.3┬á┬▒┬á0.9┬á┬▒┬á0.4	4.878┬á┬▒┬á2.3┬á┬▒┬á0.9┬á┬▒┬á0.4	5.037┬á┬▒┬á2.3┬á┬▒┬á0.9┬á┬▒┬á0.8	4.995┬á┬▒┬á1.9┬á┬▒┬á0.8┬á┬▒┬á0.8
0.064ΓÇô0.072	4.364┬á┬▒┬á3.2┬á┬▒┬á2.0┬á┬▒┬á1.0	4.489┬á┬▒┬á3.2┬á┬▒┬á2.0┬á┬▒┬á1.5	4.181┬á┬▒┬á2.2┬á┬▒┬á0.9┬á┬▒┬á0.4	4.203┬á┬▒┬á2.2┬á┬▒┬á0.9┬á┬▒┬á0.4	4.359┬á┬▒┬á2.2┬á┬▒┬á0.9┬á┬▒┬á0.8	4.383┬á┬▒┬á1.8┬á┬▒┬á0.9┬á┬▒┬á0.8
0.072ΓÇô0.081	3.733┬á┬▒┬á3.2┬á┬▒┬á2.0┬á┬▒┬á0.9	3.810┬á┬▒┬á3.2┬á┬▒┬á2.1┬á┬▒┬á1.5	3.849┬á┬▒┬á2.2┬á┬▒┬á0.9┬á┬▒┬á0.5	3.856┬á┬▒┬á2.2┬á┬▒┬á0.9┬á┬▒┬á0.5	3.958┬á┬▒┬á2.2┬á┬▒┬á0.9┬á┬▒┬á0.8	3.911┬á┬▒┬á1.8┬á┬▒┬á0.9┬á┬▒┬á0.8
0.081ΓÇô0.091	3.600┬á┬▒┬á3.1┬á┬▒┬á1.2┬á┬▒┬á0.6	3.679┬á┬▒┬á3.1┬á┬▒┬á1.2┬á┬▒┬á1.3	3.482┬á┬▒┬á2.2┬á┬▒┬á0.9┬á┬▒┬á0.4	3.505┬á┬▒┬á2.2┬á┬▒┬á0.9┬á┬▒┬á0.4	3.594┬á┬▒┬á2.2┬á┬▒┬á0.9┬á┬▒┬á0.8	3.620┬á┬▒┬á1.8┬á┬▒┬á0.7┬á┬▒┬á0.8
0.091ΓÇô0.102	3.201┬á┬▒┬á3.1┬á┬▒┬á1.4┬á┬▒┬á0.9	3.247┬á┬▒┬á3.1┬á┬▒┬á1.5┬á┬▒┬á1.5	3.279┬á┬▒┬á2.3┬á┬▒┬á0.9┬á┬▒┬á0.5	3.283┬á┬▒┬á2.3┬á┬▒┬á0.9┬á┬▒┬á0.5	3.344┬á┬▒┬á2.3┬á┬▒┬á0.9┬á┬▒┬á0.8	3.307┬á┬▒┬á1.8┬á┬▒┬á0.8┬á┬▒┬á0.9
0.102ΓÇô0.114	2.927┬á┬▒┬á3.1┬á┬▒┬á1.4┬á┬▒┬á1.3	2.942┬á┬▒┬á3.1┬á┬▒┬á1.5┬á┬▒┬á1.7	2.898┬á┬▒┬á2.2┬á┬▒┬á0.9┬á┬▒┬á0.3	2.901┬á┬▒┬á2.2┬á┬▒┬á0.9┬á┬▒┬á0.3	2.921┬á┬▒┬á2.2┬á┬▒┬á0.9┬á┬▒┬á0.7	2.932┬á┬▒┬á1.8┬á┬▒┬á0.8┬á┬▒┬á0.8
0.114ΓÇô0.128	2.599┬á┬▒┬á3.0┬á┬▒┬á1.9┬á┬▒┬á0.9	2.618┬á┬▒┬á3.0┬á┬▒┬á2.0┬á┬▒┬á1.5	2.526┬á┬▒┬á2.2┬á┬▒┬á0.9┬á┬▒┬á0.5	2.528┬á┬▒┬á2.2┬á┬▒┬á0.9┬á┬▒┬á0.5	2.560┬á┬▒┬á2.2┬á┬▒┬á0.9┬á┬▒┬á0.8	2.577┬á┬▒┬á1.8┬á┬▒┬á0.9┬á┬▒┬á0.8
0.128ΓÇô0.145	1.987┬á┬▒┬á3.1┬á┬▒┬á2.0┬á┬▒┬á1.1	1.954┬á┬▒┬á3.1┬á┬▒┬á2.0┬á┬▒┬á1.6	2.236┬á┬▒┬á2.1┬á┬▒┬á0.8┬á┬▒┬á0.4	2.241┬á┬▒┬á2.1┬á┬▒┬á0.8┬á┬▒┬á0.4	2.234┬á┬▒┬á2.1┬á┬▒┬á0.8┬á┬▒┬á0.8	2.160┬á┬▒┬á1.7┬á┬▒┬á0.8┬á┬▒┬á0.8
0.145ΓÇô0.165	1.840┬á┬▒┬á3.1┬á┬▒┬á1.3┬á┬▒┬á1.3	1.829┬á┬▒┬á3.1┬á┬▒┬á1.3┬á┬▒┬á1.7	1.862┬á┬▒┬á2.2┬á┬▒┬á0.9┬á┬▒┬á0.4	1.860┬á┬▒┬á2.2┬á┬▒┬á0.9┬á┬▒┬á0.4	1.824┬á┬▒┬á2.2┬á┬▒┬á0.9┬á┬▒┬á0.8	1.825┬á┬▒┬á1.8┬á┬▒┬á0.7┬á┬▒┬á0.9
0.165ΓÇô0.189	1.544┬á┬▒┬á3.0┬á┬▒┬á1.7┬á┬▒┬á1.1	1.500┬á┬▒┬á3.0┬á┬▒┬á1.8┬á┬▒┬á1.6	1.594┬á┬▒┬á2.1┬á┬▒┬á0.9┬á┬▒┬á0.5	1.610┬á┬▒┬á2.1┬á┬▒┬á0.9┬á┬▒┬á0.5	1.546┬á┬▒┬á2.1┬á┬▒┬á0.9┬á┬▒┬á0.8	1.533┬á┬▒┬á1.7┬á┬▒┬á0.8┬á┬▒┬á0.9
0.189ΓÇô0.219	1.302┬á┬▒┬á3.0┬á┬▒┬á2.0┬á┬▒┬á1.0	1.253┬á┬▒┬á3.0┬á┬▒┬á2.1┬á┬▒┬á1.6	1.232┬á┬▒┬á2.2┬á┬▒┬á0.9┬á┬▒┬á0.5	1.233┬á┬▒┬á2.2┬á┬▒┬á0.9┬á┬▒┬á0.5	1.179┬á┬▒┬á2.2┬á┬▒┬á0.9┬á┬▒┬á0.8	1.205┬á┬▒┬á1.8┬á┬▒┬á0.9┬á┬▒┬á0.8
0.219ΓÇô0.258	1.000┬á┬▒┬á2.9┬á┬▒┬á1.6┬á┬▒┬á0.9	0.939┬á┬▒┬á2.9┬á┬▒┬á1.6┬á┬▒┬á1.6	0.977┬á┬▒┬á2.2┬á┬▒┬á0.9┬á┬▒┬á0.6	0.985┬á┬▒┬á2.2┬á┬▒┬á0.9┬á┬▒┬á0.6	0.925┬á┬▒┬á2.2┬á┬▒┬á0.9┬á┬▒┬á0.9	0.929┬á┬▒┬á1.7┬á┬▒┬á0.8┬á┬▒┬á0.9
0.258ΓÇô0.312	0.728┬á┬▒┬á2.9┬á┬▒┬á1.6┬á┬▒┬á1.5	0.669┬á┬▒┬á2.9┬á┬▒┬á1.6┬á┬▒┬á2.4	0.749┬á┬▒┬á2.0┬á┬▒┬á0.8┬á┬▒┬á0.6	0.747┬á┬▒┬á2.0┬á┬▒┬á0.8┬á┬▒┬á0.6	0.690┬á┬▒┬á2.0┬á┬▒┬á0.8┬á┬▒┬á1.9	0.685┬á┬▒┬á1.6┬á┬▒┬á0.7┬á┬▒┬á1.7
0.312ΓÇô0.391	0.538┬á┬▒┬á2.8┬á┬▒┬á1.3┬á┬▒┬á1.1	0.488┬á┬▒┬á2.8┬á┬▒┬á1.4┬á┬▒┬á2.3	0.530┬á┬▒┬á2.0┬á┬▒┬á0.8┬á┬▒┬á1.1	0.534┬á┬▒┬á2.0┬á┬▒┬á0.8┬á┬▒┬á1.1	0.473┬á┬▒┬á2.0┬á┬▒┬á0.8┬á┬▒┬á2.1	0.481┬á┬▒┬á1.6┬á┬▒┬á0.7┬á┬▒┬á1.8
0.391ΓÇô0.524	0.347┬á┬▒┬á2.7┬á┬▒┬á1.0┬á┬▒┬á1.4	0.315┬á┬▒┬á2.7┬á┬▒┬á1.1┬á┬▒┬á2.4	0.326┬á┬▒┬á2.0┬á┬▒┬á0.8┬á┬▒┬á1.2	0.327┬á┬▒┬á2.0┬á┬▒┬á0.8┬á┬▒┬á1.2	0.290┬á┬▒┬á2.0┬á┬▒┬á0.8┬á┬▒┬á2.1	0.299┬á┬▒┬á1.6┬á┬▒┬á0.7┬á┬▒┬á1.8
0.524ΓÇô0.695	0.191┬á┬▒┬á3.3┬á┬▒┬á3.0┬á┬▒┬á1.7	0.176┬á┬▒┬á3.3┬á┬▒┬á3.0┬á┬▒┬á2.6	0.181┬á┬▒┬á2.3┬á┬▒┬á0.9┬á┬▒┬á2.0	0.182┬á┬▒┬á2.3┬á┬▒┬á0.9┬á┬▒┬á2.0	0.163┬á┬▒┬á2.3┬á┬▒┬á0.9┬á┬▒┬á2.7	0.168┬á┬▒┬á1.9┬á┬▒┬á1.0┬á┬▒┬á2.1
0.695ΓÇô0.918	0.103┬á┬▒┬á3.9┬á┬▒┬á2.7┬á┬▒┬á1.7	0.0993┬á┬▒┬á3.9┬á┬▒┬á2.7┬á┬▒┬á2.6	0.102┬á┬▒┬á2.7┬á┬▒┬á1.2┬á┬▒┬á2.2	0.102┬á┬▒┬á2.7┬á┬▒┬á1.2┬á┬▒┬á2.2	0.0953┬á┬▒┬á2.7┬á┬▒┬á1.2┬á┬▒┬á2.8	0.0978┬á┬▒┬á2.2┬á┬▒┬á1.1┬á┬▒┬á2.2
0.918ΓÇô1.153	0.0543┬á┬▒┬á5.2┬á┬▒┬á4.2┬á┬▒┬á3.4	0.0529┬á┬▒┬á5.2┬á┬▒┬á4.2┬á┬▒┬á3.9	0.0543┬á┬▒┬á3.6┬á┬▒┬á1.5┬á┬▒┬á1.0	0.0539┬á┬▒┬á3.6┬á┬▒┬á1.5┬á┬▒┬á1.0	0.0519┬á┬▒┬á3.6┬á┬▒┬á1.5┬á┬▒┬á2.0	0.0526┬á┬▒┬á3.0┬á┬▒┬á1.5┬á┬▒┬á1.9
1.153ΓÇô1.496	0.0285┬á┬▒┬á6.0┬á┬▒┬á3.9┬á┬▒┬á3.8	0.0282┬á┬▒┬á6.0┬á┬▒┬á4.0┬á┬▒┬á4.4	0.0300┬á┬▒┬á4.2┬á┬▒┬á2.2┬á┬▒┬á1.4	0.0295┬á┬▒┬á4.2┬á┬▒┬á2.2┬á┬▒┬á1.4	0.0285┬á┬▒┬á4.2┬á┬▒┬á2.2┬á┬▒┬á1.6	0.0285┬á┬▒┬á3.4┬á┬▒┬á2.0┬á┬▒┬á1.7
1.496ΓÇô1.947	0.0152┬á┬▒┬á6.9┬á┬▒┬á3.6┬á┬▒┬á3.5	0.0152┬á┬▒┬á6.9┬á┬▒┬á3.6┬á┬▒┬á4.1	0.0150┬á┬▒┬á5.7┬á┬▒┬á2.8┬á┬▒┬á1.8	0.0152┬á┬▒┬á5.7┬á┬▒┬á2.8┬á┬▒┬á1.8	0.0153┬á┬▒┬á5.7┬á┬▒┬á2.8┬á┬▒┬á2.0	0.0154┬á┬▒┬á4.4┬á┬▒┬á2.2┬á┬▒┬á2.0
1.947ΓÇô2.522	0.00628┬á┬▒┬á9.3┬á┬▒┬á4.2┬á┬▒┬á3.9	0.00634┬á┬▒┬á9.3┬á┬▒┬á4.3┬á┬▒┬á4.4	0.00625┬á┬▒┬á7.0┬á┬▒┬á3.3┬á┬▒┬á1.9	0.00634┬á┬▒┬á7.0┬á┬▒┬á3.3┬á┬▒┬á1.9	0.00619┬á┬▒┬á7.0┬á┬▒┬á3.3┬á┬▒┬á2.0	0.00631┬á┬▒┬á5.6┬á┬▒┬á2.6┬á┬▒┬á2.1
2.522ΓÇô3.277	0.00274┬á┬▒┬á13┬á┬▒┬á4.4┬á┬▒┬á4.7	0.00272┬á┬▒┬á13┬á┬▒┬á4.5┬á┬▒┬á5.2	0.00292┬á┬▒┬á8.8┬á┬▒┬á3.8┬á┬▒┬á1.9	0.00294┬á┬▒┬á8.8┬á┬▒┬á3.8┬á┬▒┬á1.9	0.00293┬á┬▒┬á8.8┬á┬▒┬á3.8┬á┬▒┬á2.0	0.00284┬á┬▒┬á7.2┬á┬▒┬á2.9┬á┬▒┬á2.2
3.277ΓÇô5.000	0.00115┬á┬▒┬á12┬á┬▒┬á4.3┬á┬▒┬á5.4	0.00118┬á┬▒┬á12┬á┬▒┬á4.4┬á┬▒┬á5.9	0.00102┬á┬▒┬á9.8┬á┬▒┬á4.3┬á┬▒┬á1.8	0.00106┬á┬▒┬á9.8┬á┬▒┬á4.3┬á┬▒┬á1.8	0.00104┬á┬▒┬á9.8┬á┬▒┬á4.3┬á┬▒┬á2.0	0.00109┬á┬▒┬á7.6┬á┬▒┬á3.1┬á┬▒┬á2.4
5.000ΓÇô10.000	0.000213┬á┬▒┬á17┬á┬▒┬á5.6┬á┬▒┬á5.1	0.000209┬á┬▒┬á17┬á┬▒┬á5.7┬á┬▒┬á5.4	0.000309┬á┬▒┬á10┬á┬▒┬á4.5┬á┬▒┬á2.2	0.000321┬á┬▒┬á10┬á┬▒┬á4.5┬á┬▒┬á2.2	0.000354┬á┬▒┬á10┬á┬▒┬á4.5┬á┬▒┬á2.3	0.000295┬á┬▒┬á9.0┬á┬▒┬á3.5┬á┬▒┬á2.3

**Table 8:** The values of $(1/\sigma )\,{\mathrm {d}}\sigma /{\mathrm {d}}\phi ^*_{\eta }$ in each bin of $\phi ^*_{\eta }$ for the electron and muon channels separately (for various particle-level definitions) and for the Born-level combination in the kinematic region $66\ ~{\mathrm {GeV}}\le m_{\ell \ell }< 116\ ~{\mathrm {GeV}},\ 0 \le |y_{\ell \ell }| < 0.4$. The associated statistical and systematic (both uncorrelated and correlated between bins of $\phi ^*_{\eta }$) are provided in percentage form

Bin	$(1/\sigma )\,d\sigma /d\phi ^*_{\eta }$ ┬▒ Statistical [%] ┬▒ Uncorrelated systematic [%] ┬▒ Correlated systematic [%]					
	Electron channel		Muon channel			Combination
	Dressed	Born	Bare	Dressed	Born	Born
0.000ΓÇô0.004	9.252┬á┬▒┬á0.4┬á┬▒┬á0.1┬á┬▒┬á0.2	9.331┬á┬▒┬á0.4┬á┬▒┬á0.1┬á┬▒┬á0.2	9.359┬á┬▒┬á0.4┬á┬▒┬á0.1┬á┬▒┬á0.1	9.359┬á┬▒┬á0.4┬á┬▒┬á0.1┬á┬▒┬á0.1	9.437┬á┬▒┬á0.4┬á┬▒┬á0.1┬á┬▒┬á0.2	9.386┬á┬▒┬á0.3┬á┬▒┬á0.1┬á┬▒┬á0.2
0.004ΓÇô0.008	9.264┬á┬▒┬á0.4┬á┬▒┬á0.1┬á┬▒┬á0.1	9.353┬á┬▒┬á0.4┬á┬▒┬á0.1┬á┬▒┬á0.2	9.279┬á┬▒┬á0.4┬á┬▒┬á0.1┬á┬▒┬á0.1	9.270┬á┬▒┬á0.4┬á┬▒┬á0.1┬á┬▒┬á0.1	9.347┬á┬▒┬á0.4┬á┬▒┬á0.1┬á┬▒┬á0.1	9.346┬á┬▒┬á0.3┬á┬▒┬á0.1┬á┬▒┬á0.1
0.008ΓÇô0.012	9.001┬á┬▒┬á0.4┬á┬▒┬á0.1┬á┬▒┬á0.1	9.067┬á┬▒┬á0.4┬á┬▒┬á0.1┬á┬▒┬á0.1	9.079┬á┬▒┬á0.4┬á┬▒┬á0.1┬á┬▒┬á0.1	9.074┬á┬▒┬á0.4┬á┬▒┬á0.1┬á┬▒┬á0.1	9.161┬á┬▒┬á0.4┬á┬▒┬á0.1┬á┬▒┬á0.1	9.115┬á┬▒┬á0.3┬á┬▒┬á0.1┬á┬▒┬á0.1
0.012ΓÇô0.016	8.810┬á┬▒┬á0.4┬á┬▒┬á0.1┬á┬▒┬á0.1	8.881┬á┬▒┬á0.4┬á┬▒┬á0.1┬á┬▒┬á0.1	8.878┬á┬▒┬á0.4┬á┬▒┬á0.1┬á┬▒┬á0.1	8.875┬á┬▒┬á0.4┬á┬▒┬á0.1┬á┬▒┬á0.1	8.933┬á┬▒┬á0.4┬á┬▒┬á0.1┬á┬▒┬á0.1	8.908┬á┬▒┬á0.3┬á┬▒┬á0.1┬á┬▒┬á0.1
0.016ΓÇô0.020	8.627┬á┬▒┬á0.4┬á┬▒┬á0.1┬á┬▒┬á0.1	8.697┬á┬▒┬á0.4┬á┬▒┬á0.1┬á┬▒┬á0.1	8.479┬á┬▒┬á0.4┬á┬▒┬á0.1┬á┬▒┬á0.1	8.474┬á┬▒┬á0.4┬á┬▒┬á0.1┬á┬▒┬á0.1	8.534┬á┬▒┬á0.4┬á┬▒┬á0.1┬á┬▒┬á0.1	8.607┬á┬▒┬á0.3┬á┬▒┬á0.1┬á┬▒┬á0.1
0.020ΓÇô0.024	8.112┬á┬▒┬á0.4┬á┬▒┬á0.1┬á┬▒┬á0.1	8.162┬á┬▒┬á0.4┬á┬▒┬á0.1┬á┬▒┬á0.1	8.163┬á┬▒┬á0.4┬á┬▒┬á0.1┬á┬▒┬á0.1	8.164┬á┬▒┬á0.4┬á┬▒┬á0.1┬á┬▒┬á0.1	8.223┬á┬▒┬á0.4┬á┬▒┬á0.1┬á┬▒┬á0.1	8.196┬á┬▒┬á0.3┬á┬▒┬á0.1┬á┬▒┬á0.1
0.024ΓÇô0.029	7.778┬á┬▒┬á0.4┬á┬▒┬á0.1┬á┬▒┬á0.1	7.817┬á┬▒┬á0.4┬á┬▒┬á0.1┬á┬▒┬á0.1	7.823┬á┬▒┬á0.4┬á┬▒┬á0.1┬á┬▒┬á0.0	7.822┬á┬▒┬á0.4┬á┬▒┬á0.1┬á┬▒┬á0.0	7.864┬á┬▒┬á0.4┬á┬▒┬á0.1┬á┬▒┬á0.1	7.840┬á┬▒┬á0.3┬á┬▒┬á0.1┬á┬▒┬á0.1
0.029ΓÇô0.034	7.344┬á┬▒┬á0.4┬á┬▒┬á0.1┬á┬▒┬á0.1	7.384┬á┬▒┬á0.4┬á┬▒┬á0.1┬á┬▒┬á0.1	7.401┬á┬▒┬á0.4┬á┬▒┬á0.1┬á┬▒┬á0.0	7.395┬á┬▒┬á0.4┬á┬▒┬á0.1┬á┬▒┬á0.0	7.431┬á┬▒┬á0.4┬á┬▒┬á0.1┬á┬▒┬á0.1	7.407┬á┬▒┬á0.3┬á┬▒┬á0.1┬á┬▒┬á0.1
0.034ΓÇô0.039	6.884┬á┬▒┬á0.4┬á┬▒┬á0.1┬á┬▒┬á0.1	6.909┬á┬▒┬á0.4┬á┬▒┬á0.1┬á┬▒┬á0.1	6.861┬á┬▒┬á0.4┬á┬▒┬á0.1┬á┬▒┬á0.0	6.863┬á┬▒┬á0.4┬á┬▒┬á0.1┬á┬▒┬á0.0	6.898┬á┬▒┬á0.4┬á┬▒┬á0.1┬á┬▒┬á0.1	6.903┬á┬▒┬á0.3┬á┬▒┬á0.1┬á┬▒┬á0.1
0.039ΓÇô0.045	6.367┬á┬▒┬á0.4┬á┬▒┬á0.1┬á┬▒┬á0.1	6.375┬á┬▒┬á0.4┬á┬▒┬á0.1┬á┬▒┬á0.1	6.392┬á┬▒┬á0.4┬á┬▒┬á0.1┬á┬▒┬á0.0	6.392┬á┬▒┬á0.4┬á┬▒┬á0.1┬á┬▒┬á0.0	6.416┬á┬▒┬á0.4┬á┬▒┬á0.1┬á┬▒┬á0.1	6.396┬á┬▒┬á0.3┬á┬▒┬á0.1┬á┬▒┬á0.1
0.045ΓÇô0.051	5.865┬á┬▒┬á0.4┬á┬▒┬á0.1┬á┬▒┬á0.1	5.873┬á┬▒┬á0.4┬á┬▒┬á0.1┬á┬▒┬á0.1	5.877┬á┬▒┬á0.4┬á┬▒┬á0.1┬á┬▒┬á0.0	5.872┬á┬▒┬á0.4┬á┬▒┬á0.1┬á┬▒┬á0.0	5.878┬á┬▒┬á0.4┬á┬▒┬á0.1┬á┬▒┬á0.1	5.875┬á┬▒┬á0.3┬á┬▒┬á0.1┬á┬▒┬á0.1
0.051ΓÇô0.057	5.438┬á┬▒┬á0.4┬á┬▒┬á0.1┬á┬▒┬á0.1	5.441┬á┬▒┬á0.4┬á┬▒┬á0.1┬á┬▒┬á0.1	5.430┬á┬▒┬á0.4┬á┬▒┬á0.1┬á┬▒┬á0.0	5.434┬á┬▒┬á0.4┬á┬▒┬á0.1┬á┬▒┬á0.0	5.436┬á┬▒┬á0.4┬á┬▒┬á0.1┬á┬▒┬á0.1	5.438┬á┬▒┬á0.3┬á┬▒┬á0.1┬á┬▒┬á0.1
0.057ΓÇô0.064	4.954┬á┬▒┬á0.4┬á┬▒┬á0.1┬á┬▒┬á0.1	4.952┬á┬▒┬á0.4┬á┬▒┬á0.1┬á┬▒┬á0.1	4.970┬á┬▒┬á0.4┬á┬▒┬á0.1┬á┬▒┬á0.1	4.966┬á┬▒┬á0.4┬á┬▒┬á0.1┬á┬▒┬á0.1	4.962┬á┬▒┬á0.4┬á┬▒┬á0.1┬á┬▒┬á0.2	4.957┬á┬▒┬á0.3┬á┬▒┬á0.1┬á┬▒┬á0.1
0.064ΓÇô0.072	4.522┬á┬▒┬á0.4┬á┬▒┬á0.1┬á┬▒┬á0.1	4.514┬á┬▒┬á0.4┬á┬▒┬á0.1┬á┬▒┬á0.1	4.514┬á┬▒┬á0.4┬á┬▒┬á0.1┬á┬▒┬á0.1	4.514┬á┬▒┬á0.4┬á┬▒┬á0.1┬á┬▒┬á0.1	4.503┬á┬▒┬á0.4┬á┬▒┬á0.1┬á┬▒┬á0.2	4.507┬á┬▒┬á0.3┬á┬▒┬á0.1┬á┬▒┬á0.1
0.072ΓÇô0.081	4.021┬á┬▒┬á0.4┬á┬▒┬á0.1┬á┬▒┬á0.1	4.011┬á┬▒┬á0.4┬á┬▒┬á0.1┬á┬▒┬á0.1	3.984┬á┬▒┬á0.4┬á┬▒┬á0.1┬á┬▒┬á0.1	3.983┬á┬▒┬á0.4┬á┬▒┬á0.1┬á┬▒┬á0.1	3.970┬á┬▒┬á0.4┬á┬▒┬á0.1┬á┬▒┬á0.2	3.988┬á┬▒┬á0.3┬á┬▒┬á0.1┬á┬▒┬á0.1
0.081ΓÇô0.091	3.572┬á┬▒┬á0.4┬á┬▒┬á0.1┬á┬▒┬á0.1	3.558┬á┬▒┬á0.4┬á┬▒┬á0.1┬á┬▒┬á0.1	3.576┬á┬▒┬á0.4┬á┬▒┬á0.1┬á┬▒┬á0.1	3.576┬á┬▒┬á0.4┬á┬▒┬á0.1 ┬á┬▒┬á0.1	3.567┬á┬▒┬á0.4┬á┬▒┬á0.1┬á┬▒┬á0.2	3.561┬á┬▒┬á0.3┬á┬▒┬á0.1┬á┬▒┬á0.1
0.091ΓÇô0.102	3.145┬á┬▒┬á0.4┬á┬▒┬á0.1┬á┬▒┬á0.1	3.132┬á┬▒┬á0.4┬á┬▒┬á0.1┬á┬▒┬á0.1	3.165┬á┬▒┬á0.4┬á┬▒┬á0.1┬á┬▒┬á0.1	3.165┬á┬▒┬á0.4┬á┬▒┬á0.1┬á┬▒┬á0.1	3.145┬á┬▒┬á0.4┬á┬▒┬á0.1┬á┬▒┬á0.2	3.138┬á┬▒┬á0.3┬á┬▒┬á0.1┬á┬▒┬á0.1
0.102ΓÇô0.114	2.764┬á┬▒┬á0.4┬á┬▒┬á0.1┬á┬▒┬á0.1	2.752┬á┬▒┬á0.4┬á┬▒┬á0.1┬á┬▒┬á0.1	2.774┬á┬▒┬á0.4┬á┬▒┬á0.1┬á┬▒┬á0.1	2.773┬á┬▒┬á0.4┬á┬▒┬á0.1┬á┬▒┬á0.1	2.763┬á┬▒┬á0.4┬á┬▒┬á0.1┬á┬▒┬á0.2	2.757┬á┬▒┬á0.3┬á┬▒┬á0.1┬á┬▒┬á0.1
0.114ΓÇô0.128	2.394┬á┬▒┬á0.4┬á┬▒┬á0.1┬á┬▒┬á0.1	2.382┬á┬▒┬á0.4┬á┬▒┬á0.1┬á┬▒┬á0.1	2.379┬á┬▒┬á0.4┬á┬▒┬á0.1┬á┬▒┬á0.1	2.378┬á┬▒┬á0.4┬á┬▒┬á0.1┬á┬▒┬á0.1	2.363┬á┬▒┬á0.4┬á┬▒┬á0.1┬á┬▒┬á0.1	2.371┬á┬▒┬á0.3┬á┬▒┬á0.1┬á┬▒┬á0.1
0.128ΓÇô0.145	2.023┬á┬▒┬á0.4┬á┬▒┬á0.1┬á┬▒┬á0.1	2.009┬á┬▒┬á0.4┬á┬▒┬á0.1┬á┬▒┬á0.1	2.037┬á┬▒┬á0.4┬á┬▒┬á0.1┬á┬▒┬á0.1	2.038┬á┬▒┬á0.4┬á┬▒┬á0.1┬á┬▒┬á0.1	2.026┬á┬▒┬á0.4┬á┬▒┬á0.1┬á┬▒┬á0.1	2.017┬á┬▒┬á0.3┬á┬▒┬á0.1┬á┬▒┬á0.1
0.145ΓÇô0.165	1.697┬á┬▒┬á0.4┬á┬▒┬á0.1┬á┬▒┬á0.1	1.686┬á┬▒┬á0.4┬á┬▒┬á0.1┬á┬▒┬á0.1	1.698┬á┬▒┬á0.4┬á┬▒┬á0.1┬á┬▒┬á0.1	1.698┬á┬▒┬á0.4┬á┬▒┬á0.1┬á┬▒┬á0.1	1.688┬á┬▒┬á0.4┬á┬▒┬á0.1┬á┬▒┬á0.1	1.687┬á┬▒┬á0.3┬á┬▒┬á0.1┬á┬▒┬á0.1
0.165ΓÇô0.189	1.396┬á┬▒┬á0.4┬á┬▒┬á0.1┬á┬▒┬á0.1	1.388┬á┬▒┬á0.4┬á┬▒┬á0.1┬á┬▒┬á0.1	1.391┬á┬▒┬á0.4┬á┬▒┬á0.1┬á┬▒┬á0.1	1.391┬á┬▒┬á0.4┬á┬▒┬á0.1┬á┬▒┬á0.1	1.382┬á┬▒┬á0.4┬á┬▒┬á0.1┬á┬▒┬á0.2	1.384┬á┬▒┬á0.3┬á┬▒┬á0.1┬á┬▒┬á0.1
0.189ΓÇô0.219	1.111┬á┬▒┬á0.4┬á┬▒┬á0.1┬á┬▒┬á0.1	1.104┬á┬▒┬á0.4┬á┬▒┬á0.1┬á┬▒┬á0.1	1.105┬á┬▒┬á0.4┬á┬▒┬á0.1┬á┬▒┬á0.1	1.106┬á┬▒┬á0.4┬á┬▒┬á0.1┬á┬▒┬á0.1	1.098┬á┬▒┬á0.4┬á┬▒┬á0.1┬á┬▒┬á0.2	1.101┬á┬▒┬á0.3┬á┬▒┬á0.1┬á┬▒┬á0.1
0.219ΓÇô0.258	0.851┬á┬▒┬á0.4┬á┬▒┬á0.1┬á┬▒┬á0.1	0.846┬á┬▒┬á0.4┬á┬▒┬á0.1┬á┬▒┬á0.1	0.856┬á┬▒┬á0.4┬á┬▒┬á0.1┬á┬▒┬á0.1	0.857┬á┬▒┬á0.4┬á┬▒┬á0.1┬á┬▒┬á0.1	0.852┬á┬▒┬á0.4┬á┬▒┬á0.1┬á┬▒┬á0.2	0.849┬á┬▒┬á0.3┬á┬▒┬á0.1┬á┬▒┬á0.1
0.258ΓÇô0.312	0.618┬á┬▒┬á0.4┬á┬▒┬á0.1┬á┬▒┬á0.2	0.615┬á┬▒┬á0.4┬á┬▒┬á0.1┬á┬▒┬á0.3	0.616┬á┬▒┬á0.4┬á┬▒┬á0.1┬á┬▒┬á0.1	0.616┬á┬▒┬á0.4┬á┬▒┬á0.1┬á┬▒┬á0.1	0.613┬á┬▒┬á0.4┬á┬▒┬á0.1┬á┬▒┬á0.2	0.614┬á┬▒┬á0.3┬á┬▒┬á0.1┬á┬▒┬á0.2
0.312ΓÇô0.391	0.411┬á┬▒┬á0.4┬á┬▒┬á0.1┬á┬▒┬á0.3	0.410┬á┬▒┬á0.4┬á┬▒┬á0.1┬á┬▒┬á0.3	0.412┬á┬▒┬á0.4┬á┬▒┬á0.1┬á┬▒┬á0.2	0.412┬á┬▒┬á0.4┬á┬▒┬á0.1┬á┬▒┬á0.2	0.410┬á┬▒┬á0.4┬á┬▒┬á0.1┬á┬▒┬á0.2	0.410┬á┬▒┬á0.3┬á┬▒┬á0.1┬á┬▒┬á0.2
0.391ΓÇô0.524	0.241┬á┬▒┬á0.4┬á┬▒┬á0.1┬á┬▒┬á0.3	0.240┬á┬▒┬á0.4┬á┬▒┬á0.1┬á┬▒┬á0.3	0.239┬á┬▒┬á0.4┬á┬▒┬á0.1┬á┬▒┬á0.2	0.239┬á┬▒┬á0.4┬á┬▒┬á0.1┬á┬▒┬á0.2	0.239┬á┬▒┬á0.4┬á┬▒┬á0.1┬á┬▒┬á0.2	0.239┬á┬▒┬á0.3┬á┬▒┬á0.1┬á┬▒┬á0.2
0.524ΓÇô0.695	0.126┬á┬▒┬á0.5┬á┬▒┬á0.2┬á┬▒┬á0.3	0.126┬á┬▒┬á0.5┬á┬▒┬á0.2┬á┬▒┬á0.3	0.124┬á┬▒┬á0.5┬á┬▒┬á0.1┬á┬▒┬á0.2	0.124┬á┬▒┬á0.5┬á┬▒┬á0.1┬á┬▒┬á0.2	0.124┬á┬▒┬á0.5┬á┬▒┬á0.1┬á┬▒┬á0.3	0.125┬á┬▒┬á0.4┬á┬▒┬á0.1┬á┬▒┬á0.2
0.695ΓÇô0.918	0.0635┬á┬▒┬á0.7┬á┬▒┬á0.2┬á┬▒┬á0.3	0.0633┬á┬▒┬á0.7┬á┬▒┬á0.2┬á┬▒┬á0.3	0.0631┬á┬▒┬á0.6┬á┬▒┬á0.2┬á┬▒┬á0.3	0.0631┬á┬▒┬á0.6┬á┬▒┬á0.2┬á┬▒┬á0.3	0.0629┬á┬▒┬á0.6┬á┬▒┬á0.2┬á┬▒┬á0.3	0.0631┬á┬▒┬á0.5┬á┬▒┬á0.1┬á┬▒┬á0.3
0.918ΓÇô1.153	0.0335┬á┬▒┬á0.9┬á┬▒┬á0.3┬á┬▒┬á0.3	0.0334┬á┬▒┬á0.9┬á┬▒┬á0.3┬á┬▒┬á0.3	0.0328┬á┬▒┬á0.8┬á┬▒┬á0.2┬á┬▒┬á0.4	0.0329┬á┬▒┬á0.8┬á┬▒┬á0.2┬á┬▒┬á0.4	0.0328┬á┬▒┬á0.8┬á┬▒┬á0.2┬á┬▒┬á0.4	0.0331┬á┬▒┬á0.6┬á┬▒┬á0.2┬á┬▒┬á0.3
1.153ΓÇô1.496	0.0178┬á┬▒┬á1.0┬á┬▒┬á0.2┬á┬▒┬á0.4	0.0177┬á┬▒┬á1.0┬á┬▒┬á0.2┬á┬▒┬á0.4	0.0173┬á┬▒┬á1.0┬á┬▒┬á0.2┬á┬▒┬á0.4	0.0173┬á┬▒┬á1.0┬á┬▒┬á0.2┬á┬▒┬á0.4	0.0173┬á┬▒┬á1.0┬á┬▒┬á0.2┬á┬▒┬á0.5	0.0175┬á┬▒┬á0.7┬á┬▒┬á0.2┬á┬▒┬á0.4
1.496ΓÇô1.947	0.00883┬á┬▒┬á1.2┬á┬▒┬á0.3┬á┬▒┬á0.4	0.00880┬á┬▒┬á1.2┬á┬▒┬á0.3┬á┬▒┬á0.4	0.00875┬á┬▒┬á1.2┬á┬▒┬á0.3┬á┬▒┬á0.4	0.00878┬á┬▒┬á1.2┬á┬▒┬á0.3┬á┬▒┬á0.4	0.00877┬á┬▒┬á1.2┬á┬▒┬á0.3┬á┬▒┬á0.5	0.00879┬á┬▒┬á0.9┬á┬▒┬á0.2┬á┬▒┬á0.4
1.947ΓÇô2.522	0.00451┬á┬▒┬á1.6┬á┬▒┬á0.4┬á┬▒┬á0.6	0.00449┬á┬▒┬á1.6┬á┬▒┬á0.4┬á┬▒┬á0.6	0.00444┬á┬▒┬á1.4┬á┬▒┬á0.4┬á┬▒┬á0.5	0.00445┬á┬▒┬á1.4┬á┬▒┬á0.4┬á┬▒┬á0.5	0.00443┬á┬▒┬á1.4┬á┬▒┬á0.4┬á┬▒┬á0.6	0.00446┬á┬▒┬á1.1┬á┬▒┬á0.3┬á┬▒┬á0.4
2.522ΓÇô3.277	0.00239┬á┬▒┬á1.9┬á┬▒┬á0.4┬á┬▒┬á0.6	0.00238┬á┬▒┬á1.9┬á┬▒┬á0.4┬á┬▒┬á0.6	0.00227┬á┬▒┬á1.7┬á┬▒┬á0.5┬á┬▒┬á0.6	0.00228┬á┬▒┬á1.7┬á┬▒┬á0.5┬á┬▒┬á0.6	0.00227┬á┬▒┬á1.7┬á┬▒┬á0.5┬á┬▒┬á0.6	0.00232┬á┬▒┬á1.3┬á┬▒┬á0.3┬á┬▒┬á0.4
3.277ΓÇô5.000	0.00103┬á┬▒┬á1.9┬á┬▒┬á0.4┬á┬▒┬á0.7	0.00103┬á┬▒┬á1.9┬á┬▒┬á0.4┬á┬▒┬á0.7	0.00102┬á┬▒┬á1.7┬á┬▒┬á0.4┬á┬▒┬á0.6	0.00102┬á┬▒┬á1.7┬á┬▒┬á0.4┬á┬▒┬á0.6	0.00102┬á┬▒┬á1.7┬á┬▒┬á0.4┬á┬▒┬á0.6	0.00102┬á┬▒┬á1.3┬á┬▒┬á0.3┬á┬▒┬á0.5
5.000ΓÇô10.000	0.000306┬á┬▒┬á2.1┬á┬▒┬á0.5┬á┬▒┬á0.7	0.000306┬á┬▒┬á2.1┬á┬▒┬á0.5┬á┬▒┬á0.7	0.000300┬á┬▒┬á1.9┬á┬▒┬á0.5┬á┬▒┬á0.6	0.000301┬á┬▒┬á1.9┬á┬▒┬á0.5┬á┬▒┬á0.6	0.000301┬á┬▒┬á1.9┬á┬▒┬á0.5┬á┬▒┬á0.7	0.000303┬á┬▒┬á1.4┬á┬▒┬á0.3┬á┬▒┬á0.5

**Table 9:** The values of $(1/\sigma )\,{\mathrm {d}}\sigma /{\mathrm {d}}\phi ^*_{\eta }$ in each bin of $\phi ^*_{\eta }$ for the electron and muon channels separately (for various particle-level definitions) and for the Born-level combination in the kinematic region $66\ ~{\mathrm {GeV}}\le m_{\ell \ell }< 116\ ~{\mathrm {GeV}},\ 0.4 \le |y_{\ell \ell }| < 0.8$. The associated statistical and systematic (both uncorrelated and correlated between bins of $\phi ^*_{\eta }$) are provided in percentage form

Bin	$(1/\sigma )\,d\sigma /d\phi ^*_{\eta }$ ┬▒ Statistical [%] ┬▒ Uncorrelated systematic [%] ┬▒ Correlated systematic [%]					
	Electron channel		Muon channel			Combination
	Dressed	Born	Bare	Dressed	Born	Born
0.000ΓÇô0.004	9.405┬á┬▒┬á0.4┬á┬▒┬á0.1┬á┬▒┬á0.2	9.503┬á┬▒┬á0.4┬á┬▒┬á0.1┬á┬▒┬á0.2	9.386┬á┬▒┬á0.4┬á┬▒┬á0.1┬á┬▒┬á0.1	9.383┬á┬▒┬á0.4┬á┬▒┬á0.1┬á┬▒┬á0.1	9.452┬á┬▒┬á0.4┬á┬▒┬á0.1┬á┬▒┬á0.2	9.465┬á┬▒┬á0.3┬á┬▒┬á0.1┬á┬▒┬á0.2
0.004ΓÇô0.008	9.230┬á┬▒┬á0.4┬á┬▒┬á0.1┬á┬▒┬á0.1	9.300┬á┬▒┬á0.4┬á┬▒┬á0.1┬á┬▒┬á0.1	9.290┬á┬▒┬á0.4┬á┬▒┬á0.1┬á┬▒┬á0.1	9.289┬á┬▒┬á0.4┬á┬▒┬á0.1┬á┬▒┬á0.1	9.372┬á┬▒┬á0.4┬á┬▒┬á0.1┬á┬▒┬á0.1	9.332┬á┬▒┬á0.3┬á┬▒┬á0.1┬á┬▒┬á0.1
0.008ΓÇô0.012	9.055┬á┬▒┬á0.4┬á┬▒┬á0.1┬á┬▒┬á0.1	9.140┬á┬▒┬á0.4┬á┬▒┬á0.1┬á┬▒┬á0.1	9.100┬á┬▒┬á0.4┬á┬▒┬á0.1┬á┬▒┬á0.0	9.102┬á┬▒┬á0.4┬á┬▒┬á0.1┬á┬▒┬á0.0	9.173┬á┬▒┬á0.4┬á┬▒┬á0.1┬á┬▒┬á0.1	9.148┬á┬▒┬á0.3┬á┬▒┬á0.1┬á┬▒┬á0.1
0.012ΓÇô0.016	8.813┬á┬▒┬á0.4┬á┬▒┬á0.1┬á┬▒┬á0.1	8.877┬á┬▒┬á0.4┬á┬▒┬á0.1┬á┬▒┬á0.1	8.888┬á┬▒┬á0.4┬á┬▒┬á0.1┬á┬▒┬á0.0	8.890┬á┬▒┬á0.4┬á┬▒┬á0.1┬á┬▒┬á0.0	8.960┬á┬▒┬á0.4┬á┬▒┬á0.1┬á┬▒┬á0.1	8.917┬á┬▒┬á0.3┬á┬▒┬á0.1┬á┬▒┬á0.1
0.016ΓÇô0.020	8.530┬á┬▒┬á0.4┬á┬▒┬á0.1┬á┬▒┬á0.1	8.592┬á┬▒┬á0.4┬á┬▒┬á0.1┬á┬▒┬á0.1	8.595┬á┬▒┬á0.4┬á┬▒┬á0.1┬á┬▒┬á0.0	8.590┬á┬▒┬á0.4┬á┬▒┬á0.1┬á┬▒┬á0.0	8.659┬á┬▒┬á0.4┬á┬▒┬á0.1┬á┬▒┬á0.1	8.621┬á┬▒┬á0.3┬á┬▒┬á0.1┬á┬▒┬á0.1
0.020ΓÇô0.024	8.163┬á┬▒┬á0.5┬á┬▒┬á0.1┬á┬▒┬á0.1	8.209┬á┬▒┬á0.5┬á┬▒┬á0.1┬á┬▒┬á0.1	8.210┬á┬▒┬á0.4┬á┬▒┬á0.1┬á┬▒┬á0.0	8.207┬á┬▒┬á0.4┬á┬▒┬á0.1┬á┬▒┬á0.0	8.257┬á┬▒┬á0.4┬á┬▒┬á0.1┬á┬▒┬á0.1	8.228┬á┬▒┬á0.3┬á┬▒┬á0.1┬á┬▒┬á0.1
0.024ΓÇô0.029	7.849┬á┬▒┬á0.4┬á┬▒┬á0.1┬á┬▒┬á0.1	7.898┬á┬▒┬á0.4┬á┬▒┬á0.1┬á┬▒┬á0.1	7.813┬á┬▒┬á0.4┬á┬▒┬á0.1┬á┬▒┬á0.1	7.809┬á┬▒┬á0.4┬á┬▒┬á0.1┬á┬▒┬á0.1	7.853┬á┬▒┬á0.4┬á┬▒┬á0.1┬á┬▒┬á0.2	7.863┬á┬▒┬á0.3┬á┬▒┬á0.1┬á┬▒┬á0.1
0.029ΓÇô0.034	7.382┬á┬▒┬á0.4┬á┬▒┬á0.1┬á┬▒┬á0.1	7.411┬á┬▒┬á0.4┬á┬▒┬á0.1┬á┬▒┬á0.1	7.393┬á┬▒┬á0.4┬á┬▒┬á0.1┬á┬▒┬á0.1	7.388┬á┬▒┬á0.4┬á┬▒┬á0.1┬á┬▒┬á0.1	7.427┬á┬▒┬á0.4┬á┬▒┬á0.1┬á┬▒┬á0.2	7.412┬á┬▒┬á0.3┬á┬▒┬á0.1┬á┬▒┬á0.1
0.034ΓÇô0.039	6.874┬á┬▒┬á0.4┬á┬▒┬á0.1┬á┬▒┬á0.1	6.895┬á┬▒┬á0.4┬á┬▒┬á0.1┬á┬▒┬á0.1	6.909┬á┬▒┬á0.4┬á┬▒┬á0.1┬á┬▒┬á0.1	6.907┬á┬▒┬á0.4┬á┬▒┬á0.1┬á┬▒┬á0.1	6.933┬á┬▒┬á0.4┬á┬▒┬á0.1┬á┬▒┬á0.2	6.910┬á┬▒┬á0.3┬á┬▒┬á0.1┬á┬▒┬á0.1
0.039ΓÇô0.045	6.410┬á┬▒┬á0.4┬á┬▒┬á0.1┬á┬▒┬á0.1	6.425┬á┬▒┬á0.4┬á┬▒┬á0.1┬á┬▒┬á0.1	6.425┬á┬▒┬á0.4┬á┬▒┬á0.1┬á┬▒┬á0.1	6.417┬á┬▒┬á0.4┬á┬▒┬á0.1┬á┬▒┬á0.1	6.443┬á┬▒┬á0.4┬á┬▒┬á0.1┬á┬▒┬á0.2	6.428┬á┬▒┬á0.3┬á┬▒┬á0.1┬á┬▒┬á0.1
0.045ΓÇô0.051	5.850┬á┬▒┬á0.4┬á┬▒┬á0.1┬á┬▒┬á0.1	5.856┬á┬▒┬á0.4┬á┬▒┬á0.1┬á┬▒┬á0.1	5.903┬á┬▒┬á0.4┬á┬▒┬á0.1┬á┬▒┬á0.1	5.906┬á┬▒┬á0.4┬á┬▒┬á0.1┬á┬▒┬á0.1	5.913┬á┬▒┬á0.4┬á┬▒┬á0.1┬á┬▒┬á0.2	5.882┬á┬▒┬á0.3┬á┬▒┬á0.1┬á┬▒┬á0.1
0.051ΓÇô0.057	5.427┬á┬▒┬á0.5┬á┬▒┬á0.1┬á┬▒┬á0.1	5.429┬á┬▒┬á0.5┬á┬▒┬á0.1┬á┬▒┬á0.1	5.477┬á┬▒┬á0.4┬á┬▒┬á0.1┬á┬▒┬á0.1	5.475┬á┬▒┬á0.4┬á┬▒┬á0.1┬á┬▒┬á0.1	5.477┬á┬▒┬á0.4┬á┬▒┬á0.1┬á┬▒┬á0.2	5.450┬á┬▒┬á0.3┬á┬▒┬á0.1┬á┬▒┬á0.1
0.057ΓÇô0.064	4.933┬á┬▒┬á0.4┬á┬▒┬á0.1┬á┬▒┬á0.1	4.930┬á┬▒┬á0.4┬á┬▒┬á0.1┬á┬▒┬á0.1	4.979┬á┬▒┬á0.4┬á┬▒┬á0.1┬á┬▒┬á0.1	4.977┬á┬▒┬á0.4┬á┬▒┬á0.1┬á┬▒┬á0.1	4.972┬á┬▒┬á0.4┬á┬▒┬á0.1┬á┬▒┬á0.2	4.949┬á┬▒┬á0.3┬á┬▒┬á0.1┬á┬▒┬á0.1
0.064ΓÇô0.072	4.503┬á┬▒┬á0.4┬á┬▒┬á0.1┬á┬▒┬á0.1	4.499┬á┬▒┬á0.4┬á┬▒┬á0.1┬á┬▒┬á0.1	4.490┬á┬▒┬á0.4┬á┬▒┬á0.1┬á┬▒┬á0.1	4.492┬á┬▒┬á0.4┬á┬▒┬á0.1┬á┬▒┬á0.1	4.484┬á┬▒┬á0.4┬á┬▒┬á0.1┬á┬▒┬á0.2	4.485┬á┬▒┬á0.3┬á┬▒┬á0.1┬á┬▒┬á0.1
0.072ΓÇô0.081	4.020┬á┬▒┬á0.4┬á┬▒┬á0.1┬á┬▒┬á0.1	4.011┬á┬▒┬á0.4┬á┬▒┬á0.1┬á┬▒┬á0.1	4.037┬á┬▒┬á0.4┬á┬▒┬á0.1┬á┬▒┬á0.1	4.035┬á┬▒┬á0.4┬á┬▒┬á0.1┬á┬▒┬á0.1	4.027┬á┬▒┬á0.4┬á┬▒┬á0.1┬á┬▒┬á0.2	4.016┬á┬▒┬á0.3┬á┬▒┬á0.1┬á┬▒┬á0.1
0.081ΓÇô0.091	3.585┬á┬▒┬á0.4┬á┬▒┬á0.1┬á┬▒┬á0.1	3.573┬á┬▒┬á0.4┬á┬▒┬á0.1┬á┬▒┬á0.1	3.586┬á┬▒┬á0.4┬á┬▒┬á0.1┬á┬▒┬á0.1	3.587┬á┬▒┬á0.4┬á┬▒┬á0.1┬á┬▒┬á0.1	3.579┬á┬▒┬á0.4┬á┬▒┬á0.1┬á┬▒┬á0.2	3.574┬á┬▒┬á0.3┬á┬▒┬á0.1┬á┬▒┬á0.1
0.091ΓÇô0.102	3.146┬á┬▒┬á0.4┬á┬▒┬á0.1┬á┬▒┬á0.1	3.132┬á┬▒┬á0.4┬á┬▒┬á0.1┬á┬▒┬á0.1	3.146┬á┬▒┬á0.4┬á┬▒┬á0.1┬á┬▒┬á0.1	3.144┬á┬▒┬á0.4┬á┬▒┬á0.1┬á┬▒┬á0.1	3.132┬á┬▒┬á0.4┬á┬▒┬á0.1┬á┬▒┬á0.2	3.130┬á┬▒┬á0.3┬á┬▒┬á0.1┬á┬▒┬á0.1
0.102ΓÇô0.114	2.786┬á┬▒┬á0.4┬á┬▒┬á0.1┬á┬▒┬á0.1	2.772┬á┬▒┬á0.4┬á┬▒┬á0.1┬á┬▒┬á0.1	2.753┬á┬▒┬á0.4┬á┬▒┬á0.1┬á┬▒┬á0.1	2.752┬á┬▒┬á0.4┬á┬▒┬á0.1┬á┬▒┬á0.1	2.737┬á┬▒┬á0.4┬á┬▒┬á0.1┬á┬▒┬á0.2	2.750┬á┬▒┬á0.3┬á┬▒┬á0.1┬á┬▒┬á0.1
0.114ΓÇô0.128	2.380┬á┬▒┬á0.4┬á┬▒┬á0.1┬á┬▒┬á0.1	2.365┬á┬▒┬á0.4┬á┬▒┬á0.1┬á┬▒┬á0.1	2.370┬á┬▒┬á0.4┬á┬▒┬á0.1┬á┬▒┬á0.1	2.369┬á┬▒┬á0.4┬á┬▒┬á0.1┬á┬▒┬á0.1	2.358┬á┬▒┬á0.4┬á┬▒┬á0.1┬á┬▒┬á0.1	2.361┬á┬▒┬á0.3┬á┬▒┬á0.1┬á┬▒┬á0.1
0.128ΓÇô0.145	2.034┬á┬▒┬á0.4┬á┬▒┬á0.1┬á┬▒┬á0.1	2.022┬á┬▒┬á0.4┬á┬▒┬á0.1┬á┬▒┬á0.1	2.034┬á┬▒┬á0.4┬á┬▒┬á0.1┬á┬▒┬á0.1	2.034┬á┬▒┬á0.4┬á┬▒┬á0.1┬á┬▒┬á0.1	2.021┬á┬▒┬á0.4┬á┬▒┬á0.1┬á┬▒┬á0.1	2.021┬á┬▒┬á0.3┬á┬▒┬á0.1┬á┬▒┬á0.1
0.145ΓÇô0.165	1.694┬á┬▒┬á0.4┬á┬▒┬á0.1┬á┬▒┬á0.1	1.683┬á┬▒┬á0.4┬á┬▒┬á0.1┬á┬▒┬á0.1	1.695┬á┬▒┬á0.4┬á┬▒┬á0.1┬á┬▒┬á0.1	1.695┬á┬▒┬á0.4┬á┬▒┬á0.1┬á┬▒┬á0.1	1.684┬á┬▒┬á0.4┬á┬▒┬á0.1┬á┬▒┬á0.1	1.683┬á┬▒┬á0.3┬á┬▒┬á0.1┬á┬▒┬á0.1
0.165ΓÇô0.189	1.396┬á┬▒┬á0.4┬á┬▒┬á0.1┬á┬▒┬á0.1	1.388┬á┬▒┬á0.4┬á┬▒┬á0.1┬á┬▒┬á0.1	1.381┬á┬▒┬á0.4┬á┬▒┬á0.1┬á┬▒┬á0.1	1.381┬á┬▒┬á0.4┬á┬▒┬á0.1┬á┬▒┬á0.1	1.373┬á┬▒┬á0.4┬á┬▒┬á0.1┬á┬▒┬á0.1	1.379┬á┬▒┬á0.3┬á┬▒┬á0.1┬á┬▒┬á0.1
0.189ΓÇô0.219	1.115┬á┬▒┬á0.4┬á┬▒┬á0.1┬á┬▒┬á0.1	1.108┬á┬▒┬á0.4┬á┬▒┬á0.1┬á┬▒┬á0.1	1.109┬á┬▒┬á0.4┬á┬▒┬á0.1┬á┬▒┬á0.1	1.110┬á┬▒┬á0.4┬á┬▒┬á0.1┬á┬▒┬á0.1	1.102┬á┬▒┬á0.4┬á┬▒┬á0.1┬á┬▒┬á0.1	1.105┬á┬▒┬á0.3┬á┬▒┬á0.1┬á┬▒┬á0.1
0.219ΓÇô0.258	0.853┬á┬▒┬á0.4┬á┬▒┬á0.1┬á┬▒┬á0.2	0.849┬á┬▒┬á0.4┬á┬▒┬á0.1┬á┬▒┬á0.2	0.848┬á┬▒┬á0.4┬á┬▒┬á0.1┬á┬▒┬á0.1	0.848┬á┬▒┬á0.4┬á┬▒┬á0.1┬á┬▒┬á0.1	0.843┬á┬▒┬á0.4┬á┬▒┬á0.1┬á┬▒┬á0.1	0.845┬á┬▒┬á0.3┬á┬▒┬á0.1┬á┬▒┬á0.1
0.258ΓÇô0.312	0.616┬á┬▒┬á0.4┬á┬▒┬á0.1┬á┬▒┬á0.2	0.613┬á┬▒┬á0.4┬á┬▒┬á0.1┬á┬▒┬á0.2	0.612┬á┬▒┬á0.4┬á┬▒┬á0.1┬á┬▒┬á0.1	0.612┬á┬▒┬á0.4┬á┬▒┬á0.1┬á┬▒┬á0.1	0.609┬á┬▒┬á0.4┬á┬▒┬á0.1┬á┬▒┬á0.2	0.610┬á┬▒┬á0.3┬á┬▒┬á0.1┬á┬▒┬á0.2
0.312ΓÇô0.391	0.410┬á┬▒┬á0.4┬á┬▒┬á0.1┬á┬▒┬á0.3	0.408┬á┬▒┬á0.4┬á┬▒┬á0.1┬á┬▒┬á0.3	0.410┬á┬▒┬á0.4┬á┬▒┬á0.1┬á┬▒┬á0.2	0.410┬á┬▒┬á0.4┬á┬▒┬á0.1┬á┬▒┬á0.2	0.409┬á┬▒┬á0.4┬á┬▒┬á0.1┬á┬▒┬á0.2	0.408┬á┬▒┬á0.3┬á┬▒┬á0.1┬á┬▒┬á0.2
0.391ΓÇô0.524	0.240┬á┬▒┬á0.5┬á┬▒┬á0.1┬á┬▒┬á0.3	0.240┬á┬▒┬á0.5┬á┬▒┬á0.1┬á┬▒┬á0.3	0.236┬á┬▒┬á0.4┬á┬▒┬á0.1┬á┬▒┬á0.2	0.236┬á┬▒┬á0.4┬á┬▒┬á0.1┬á┬▒┬á0.2	0.235┬á┬▒┬á0.4┬á┬▒┬á0.1┬á┬▒┬á0.2	0.237┬á┬▒┬á0.3┬á┬▒┬á0.1┬á┬▒┬á0.2
0.524ΓÇô0.695	0.124┬á┬▒┬á0.6┬á┬▒┬á0.2┬á┬▒┬á0.3	0.124┬á┬▒┬á0.6┬á┬▒┬á0.2┬á┬▒┬á0.3	0.124┬á┬▒┬á0.5┬á┬▒┬á0.1┬á┬▒┬á0.2	0.124┬á┬▒┬á0.5┬á┬▒┬á0.1┬á┬▒┬á0.2	0.124┬á┬▒┬á0.5┬á┬▒┬á0.1┬á┬▒┬á0.2	0.124┬á┬▒┬á0.4┬á┬▒┬á0.1┬á┬▒┬á0.2
0.695ΓÇô0.918	0.0629┬á┬▒┬á0.7┬á┬▒┬á0.2┬á┬▒┬á0.3	0.0627┬á┬▒┬á0.7┬á┬▒┬á0.2┬á┬▒┬á0.3	0.0623┬á┬▒┬á0.6┬á┬▒┬á0.1┬á┬▒┬á0.3	0.0623┬á┬▒┬á0.6┬á┬▒┬á0.1┬á┬▒┬á0.3	0.0621┬á┬▒┬á0.6┬á┬▒┬á0.1┬á┬▒┬á0.3	0.0623┬á┬▒┬á0.5┬á┬▒┬á0.1┬á┬▒┬á0.2
0.918ΓÇô1.153	0.0330┬á┬▒┬á0.9┬á┬▒┬á0.3┬á┬▒┬á0.3	0.0329┬á┬▒┬á0.9┬á┬▒┬á0.3┬á┬▒┬á0.3	0.0331┬á┬▒┬á0.8┬á┬▒┬á0.2┬á┬▒┬á0.3	0.0332┬á┬▒┬á0.8┬á┬▒┬á0.2┬á┬▒┬á0.3	0.0331┬á┬▒┬á0.8┬á┬▒┬á0.2┬á┬▒┬á0.3	0.0330┬á┬▒┬á0.6┬á┬▒┬á0.2┬á┬▒┬á0.2
1.153ΓÇô1.496	0.0175┬á┬▒┬á1.0┬á┬▒┬á0.3┬á┬▒┬á0.5	0.0174┬á┬▒┬á1.0┬á┬▒┬á0.3┬á┬▒┬á0.5	0.0174┬á┬▒┬á1.0┬á┬▒┬á0.2┬á┬▒┬á0.3	0.0174┬á┬▒┬á1.0┬á┬▒┬á0.2┬á┬▒┬á0.3	0.0173┬á┬▒┬á1.0┬á┬▒┬á0.2┬á┬▒┬á0.5	0.0173┬á┬▒┬á0.7┬á┬▒┬á0.2┬á┬▒┬á0.4
1.496ΓÇô1.947	0.00850┬á┬▒┬á1.3┬á┬▒┬á0.3┬á┬▒┬á0.5	0.00847┬á┬▒┬á1.3┬á┬▒┬á0.3┬á┬▒┬á0.5	0.00861┬á┬▒┬á1.2┬á┬▒┬á0.2┬á┬▒┬á0.4	0.00862┬á┬▒┬á1.2┬á┬▒┬á0.2┬á┬▒┬á0.4	0.00862┬á┬▒┬á1.2┬á┬▒┬á0.2┬á┬▒┬á0.5	0.00853┬á┬▒┬á0.9┬á┬▒┬á0.2┬á┬▒┬á0.4
1.947ΓÇô2.522	0.00440┬á┬▒┬á1.6┬á┬▒┬á0.4┬á┬▒┬á0.6	0.00439┬á┬▒┬á1.6┬á┬▒┬á0.4┬á┬▒┬á0.6	0.00438┬á┬▒┬á1.5┬á┬▒┬á0.4┬á┬▒┬á0.3	0.00439┬á┬▒┬á1.5┬á┬▒┬á0.4┬á┬▒┬á0.3	0.00437┬á┬▒┬á1.5┬á┬▒┬á0.4┬á┬▒┬á0.5	0.00437┬á┬▒┬á1.1┬á┬▒┬á0.3┬á┬▒┬á0.4
2.522ΓÇô3.277	0.00229┬á┬▒┬á2.0┬á┬▒┬á0.4┬á┬▒┬á0.6	0.00229┬á┬▒┬á2.0┬á┬▒┬á0.5┬á┬▒┬á0.6	0.00224┬á┬▒┬á1.8┬á┬▒┬á0.5┬á┬▒┬á0.4	0.00224┬á┬▒┬á1.8┬á┬▒┬á0.5┬á┬▒┬á0.4	0.00223┬á┬▒┬á1.8┬á┬▒┬á0.5┬á┬▒┬á0.6	0.00225┬á┬▒┬á1.3┬á┬▒┬á0.3┬á┬▒┬á0.4
3.277ΓÇô5.000	0.000994┬á┬▒┬á2.0┬á┬▒┬á0.6┬á┬▒┬á0.8	0.000993┬á┬▒┬á2.0┬á┬▒┬á0.6┬á┬▒┬á0.8	0.00101┬á┬▒┬á1.8┬á┬▒┬á0.4┬á┬▒┬á0.3	0.00101┬á┬▒┬á1.8┬á┬▒┬á0.4┬á┬▒┬á0.3	0.00102┬á┬▒┬á1.8┬á┬▒┬á0.4┬á┬▒┬á0.5	0.00100┬á┬▒┬á1.3┬á┬▒┬á0.4┬á┬▒┬á0.4
5.000ΓÇô10.000	0.000309┬á┬▒┬á2.1┬á┬▒┬á0.5┬á┬▒┬á0.8	0.000308┬á┬▒┬á2.1┬á┬▒┬á0.5┬á┬▒┬á0.8	0.000297┬á┬▒┬á1.9┬á┬▒┬á0.5┬á┬▒┬á0.4	0.000297┬á┬▒┬á1.9┬á┬▒┬á0.5┬á┬▒┬á0.4	0.000297┬á┬▒┬á1.9┬á┬▒┬á0.5┬á┬▒┬á0.6	0.000301┬á┬▒┬á1.4┬á┬▒┬á0.4┬á┬▒┬á0.5

**Table 10:** The values of $(1/\sigma )\,{\mathrm {d}}\sigma /{\mathrm {d}}\phi ^*_{\eta }$ in each bin of $\phi ^*_{\eta }$ for the electron and muon channels separately (for various particle-level definitions) and for the Born-level combination in the kinematic region $66\ ~{\mathrm {GeV}}\le m_{\ell \ell }< 116\ ~{\mathrm {GeV}},\ 0.8 \le |y_{\ell \ell }| < 1.2$. The associated statistical and systematic (both uncorrelated and correlated between bins of $\phi ^*_{\eta }$) are provided in percentage form

Bin	$(1/\sigma )\,d\sigma /d\phi ^*_{\eta }$ ┬▒ Statistical [%] ┬▒ Uncorrelated systematic [%] ┬▒ Correlated systematic [%]					
	Electron channel		Muon channel			Combination
	Dressed	Born	Bare	Dressed	Born	Born
0.000ΓÇô0.004	9.387┬á┬▒┬á0.4┬á┬▒┬á0.1┬á┬▒┬á0.2	9.476┬á┬▒┬á0.4┬á┬▒┬á0.1┬á┬▒┬á0.2	9.394┬á┬▒┬á0.4┬á┬▒┬á0.1┬á┬▒┬á0.1	9.388┬á┬▒┬á0.4┬á┬▒┬á0.1┬á┬▒┬á0.1	9.455┬á┬▒┬á0.4┬á┬▒┬á0.1┬á┬▒┬á0.2	9.458┬á┬▒┬á0.3┬á┬▒┬á0.1┬á┬▒┬á0.2
0.004ΓÇô0.008	9.268┬á┬▒┬á0.4┬á┬▒┬á0.1┬á┬▒┬á0.1	9.352┬á┬▒┬á0.4┬á┬▒┬á0.1┬á┬▒┬á0.1	9.374┬á┬▒┬á0.4┬á┬▒┬á0.1┬á┬▒┬á0.0	9.371┬á┬▒┬á0.4┬á┬▒┬á0.1┬á┬▒┬á0.0	9.462┬á┬▒┬á0.4┬á┬▒┬á0.1┬á┬▒┬á0.2	9.412┬á┬▒┬á0.3┬á┬▒┬á0.1┬á┬▒┬á0.1
0.008ΓÇô0.012	9.252┬á┬▒┬á0.4┬á┬▒┬á0.1┬á┬▒┬á0.1	9.330┬á┬▒┬á0.4┬á┬▒┬á0.1┬á┬▒┬á0.1	9.160┬á┬▒┬á0.4┬á┬▒┬á0.1┬á┬▒┬á0.0	9.155┬á┬▒┬á0.4┬á┬▒┬á0.1┬á┬▒┬á0.0	9.231┬á┬▒┬á0.4┬á┬▒┬á0.1┬á┬▒┬á0.2	9.265┬á┬▒┬á0.3┬á┬▒┬á0.1┬á┬▒┬á0.1
0.012ΓÇô0.016	8.913┬á┬▒┬á0.5┬á┬▒┬á0.2┬á┬▒┬á0.1	8.989┬á┬▒┬á0.5┬á┬▒┬á0.2┬á┬▒┬á0.1	8.907┬á┬▒┬á0.4┬á┬▒┬á0.1┬á┬▒┬á0.0	8.900┬á┬▒┬á0.4┬á┬▒┬á0.1┬á┬▒┬á0.0	8.968┬á┬▒┬á0.4┬á┬▒┬á0.1┬á┬▒┬á0.2	8.973┬á┬▒┬á0.3┬á┬▒┬á0.1┬á┬▒┬á0.1
0.016ΓÇô0.020	8.699┬á┬▒┬á0.5┬á┬▒┬á0.2┬á┬▒┬á0.1	8.772┬á┬▒┬á0.5┬á┬▒┬á0.2┬á┬▒┬á0.1	8.619┬á┬▒┬á0.4┬á┬▒┬á0.1┬á┬▒┬á0.0	8.613┬á┬▒┬á0.4┬á┬▒┬á0.1┬á┬▒┬á0.0	8.681┬á┬▒┬á0.4┬á┬▒┬á0.1┬á┬▒┬á0.2	8.712┬á┬▒┬á0.3┬á┬▒┬á0.1┬á┬▒┬á0.1
0.020ΓÇô0.024	8.264┬á┬▒┬á0.5┬á┬▒┬á0.1┬á┬▒┬á0.1	8.312┬á┬▒┬á0.5┬á┬▒┬á0.1┬á┬▒┬á0.1	8.352┬á┬▒┬á0.4┬á┬▒┬á0.1┬á┬▒┬á0.0	8.348┬á┬▒┬á0.4┬á┬▒┬á0.1┬á┬▒┬á0.0	8.394┬á┬▒┬á0.4┬á┬▒┬á0.1┬á┬▒┬á0.2	8.356┬á┬▒┬á0.3┬á┬▒┬á0.1┬á┬▒┬á0.1
0.024ΓÇô0.029	7.876┬á┬▒┬á0.4┬á┬▒┬á0.1┬á┬▒┬á0.1	7.920┬á┬▒┬á0.4┬á┬▒┬á0.1┬á┬▒┬á0.1	7.869┬á┬▒┬á0.4┬á┬▒┬á0.1┬á┬▒┬á0.1	7.868┬á┬▒┬á0.4┬á┬▒┬á0.1┬á┬▒┬á0.1	7.926┬á┬▒┬á0.4┬á┬▒┬á0.1┬á┬▒┬á0.1	7.918┬á┬▒┬á0.3┬á┬▒┬á0.1┬á┬▒┬á0.1
0.029ΓÇô0.034	7.364┬á┬▒┬á0.4┬á┬▒┬á0.2┬á┬▒┬á0.1	7.396┬á┬▒┬á0.4┬á┬▒┬á0.2┬á┬▒┬á0.1	7.443┬á┬▒┬á0.4┬á┬▒┬á0.1┬á┬▒┬á0.1	7.437┬á┬▒┬á0.4┬á┬▒┬á0.1┬á┬▒┬á0.1	7.473┬á┬▒┬á0.4┬á┬▒┬á0.1┬á┬▒┬á0.1	7.439┬á┬▒┬á0.3┬á┬▒┬á0.1┬á┬▒┬á0.1
0.034ΓÇô0.039	6.923┬á┬▒┬á0.5┬á┬▒┬á0.2┬á┬▒┬á0.1	6.950┬á┬▒┬á0.5┬á┬▒┬á0.2┬á┬▒┬á0.1	6.915┬á┬▒┬á0.4┬á┬▒┬á0.1┬á┬▒┬á0.0	6.906┬á┬▒┬á0.4┬á┬▒┬á0.1┬á┬▒┬á0.0	6.934┬á┬▒┬á0.4┬á┬▒┬á0.1┬á┬▒┬á0.1	6.937┬á┬▒┬á0.3┬á┬▒┬á0.1┬á┬▒┬á0.1
0.039ΓÇô0.045	6.430┬á┬▒┬á0.4┬á┬▒┬á0.1┬á┬▒┬á0.1	6.450┬á┬▒┬á0.4┬á┬▒┬á0.1┬á┬▒┬á0.1	6.484┬á┬▒┬á0.4┬á┬▒┬á0.1┬á┬▒┬á0.1	6.483┬á┬▒┬á0.4┬á┬▒┬á0.1┬á┬▒┬á0.1	6.499┬á┬▒┬á0.4┬á┬▒┬á0.1┬á┬▒┬á0.1	6.475┬á┬▒┬á0.3┬á┬▒┬á0.1┬á┬▒┬á0.1
0.045ΓÇô0.051	5.921┬á┬▒┬á0.5┬á┬▒┬á0.1┬á┬▒┬á0.1	5.923┬á┬▒┬á0.5┬á┬▒┬á0.2┬á┬▒┬á0.1	5.884┬á┬▒┬á0.4┬á┬▒┬á0.1┬á┬▒┬á0.1	5.884┬á┬▒┬á0.4┬á┬▒┬á0.1┬á┬▒┬á0.1	5.898┬á┬▒┬á0.4┬á┬▒┬á0.1┬á┬▒┬á0.1	5.905┬á┬▒┬á0.3┬á┬▒┬á0.1┬á┬▒┬á0.1
0.051ΓÇô0.057	5.410┬á┬▒┬á0.5┬á┬▒┬á0.1┬á┬▒┬á0.1	5.410┬á┬▒┬á0.5┬á┬▒┬á0.1┬á┬▒┬á0.1	5.469┬á┬▒┬á0.4┬á┬▒┬á0.1┬á┬▒┬á0.1	5.470┬á┬▒┬á0.4┬á┬▒┬á0.1┬á┬▒┬á0.1	5.466┬á┬▒┬á0.4┬á┬▒┬á0.1┬á┬▒┬á0.1	5.441┬á┬▒┬á0.3┬á┬▒┬á0.1┬á┬▒┬á0.1
0.057ΓÇô0.064	5.012┬á┬▒┬á0.5┬á┬▒┬á0.1┬á┬▒┬á0.1	5.008┬á┬▒┬á0.5┬á┬▒┬á0.1┬á┬▒┬á0.1	5.019┬á┬▒┬á0.4┬á┬▒┬á0.1┬á┬▒┬á0.1	5.016┬á┬▒┬á0.4┬á┬▒┬á0.1┬á┬▒┬á0.1	5.023┬á┬▒┬á0.4┬á┬▒┬á0.1┬á┬▒┬á0.1	5.015┬á┬▒┬á0.3┬á┬▒┬á0.1┬á┬▒┬á0.1
0.064ΓÇô0.072	4.492┬á┬▒┬á0.5┬á┬▒┬á0.1┬á┬▒┬á0.1	4.483┬á┬▒┬á0.5┬á┬▒┬á0.1┬á┬▒┬á0.1	4.506┬á┬▒┬á0.4┬á┬▒┬á0.1┬á┬▒┬á0.1	4.506┬á┬▒┬á0.4┬á┬▒┬á0.1┬á┬▒┬á0.1	4.498┬á┬▒┬á0.4┬á┬▒┬á0.1┬á┬▒┬á0.1	4.491┬á┬▒┬á0.3┬á┬▒┬á0.1┬á┬▒┬á0.1
0.072ΓÇô0.081	4.019┬á┬▒┬á0.5┬á┬▒┬á0.1┬á┬▒┬á0.1	4.009┬á┬▒┬á0.5┬á┬▒┬á0.1┬á┬▒┬á0.1	4.038┬á┬▒┬á0.4┬á┬▒┬á0.1┬á┬▒┬á0.1	4.037┬á┬▒┬á0.4┬á┬▒┬á0.1┬á┬▒┬á0.1	4.024┬á┬▒┬á0.4┬á┬▒┬á0.1┬á┬▒┬á0.1	4.016┬á┬▒┬á0.3┬á┬▒┬á0.1┬á┬▒┬á0.1
0.081ΓÇô0.091	3.589┬á┬▒┬á0.5┬á┬▒┬á0.1┬á┬▒┬á0.1	3.576┬á┬▒┬á0.5┬á┬▒┬á0.1┬á┬▒┬á0.1	3.566┬á┬▒┬á0.4┬á┬▒┬á0.1┬á┬▒┬á0.1	3.564┬á┬▒┬á0.4┬á┬▒┬á0.1┬á┬▒┬á0.1	3.550┬á┬▒┬á0.4┬á┬▒┬á0.1┬á┬▒┬á0.1	3.559┬á┬▒┬á0.3┬á┬▒┬á0.1┬á┬▒┬á0.1
0.091ΓÇô0.102	3.141┬á┬▒┬á0.5┬á┬▒┬á0.1┬á┬▒┬á0.1	3.128┬á┬▒┬á0.5┬á┬▒┬á0.1┬á┬▒┬á0.1	3.147┬á┬▒┬á0.4┬á┬▒┬á0.1┬á┬▒┬á0.1	3.149┬á┬▒┬á0.4┬á┬▒┬á0.1┬á┬▒┬á0.1	3.138┬á┬▒┬á0.4┬á┬▒┬á0.1┬á┬▒┬á0.1	3.133┬á┬▒┬á0.3┬á┬▒┬á0.1┬á┬▒┬á0.1
0.102ΓÇô0.114	2.755┬á┬▒┬á0.5┬á┬▒┬á0.1┬á┬▒┬á0.1	2.740┬á┬▒┬á0.5┬á┬▒┬á0.1┬á┬▒┬á0.1	2.746┬á┬▒┬á0.4┬á┬▒┬á0.1┬á┬▒┬á0.1	2.745┬á┬▒┬á0.4┬á┬▒┬á0.1┬á┬▒┬á0.1	2.732┬á┬▒┬á0.4┬á┬▒┬á0.1┬á┬▒┬á0.1	2.734┬á┬▒┬á0.3┬á┬▒┬á0.1┬á┬▒┬á0.1
0.114ΓÇô0.128	2.394┬á┬▒┬á0.5┬á┬▒┬á0.1┬á┬▒┬á0.1	2.380┬á┬▒┬á0.5┬á┬▒┬á0.1┬á┬▒┬á0.1	2.388┬á┬▒┬á0.4┬á┬▒┬á0.1┬á┬▒┬á0.1	2.388┬á┬▒┬á0.4┬á┬▒┬á0.1┬á┬▒┬á0.1	2.374┬á┬▒┬á0.4┬á┬▒┬á0.1┬á┬▒┬á0.1	2.375┬á┬▒┬á0.3┬á┬▒┬á0.1┬á┬▒┬á0.1
0.128ΓÇô0.145	2.029┬á┬▒┬á0.5┬á┬▒┬á0.1┬á┬▒┬á0.1	2.017┬á┬▒┬á0.5┬á┬▒┬á0.1┬á┬▒┬á0.1	2.025┬á┬▒┬á0.4┬á┬▒┬á0.1┬á┬▒┬á0.1	2.025┬á┬▒┬á0.4┬á┬▒┬á0.1┬á┬▒┬á0.1	2.015┬á┬▒┬á0.4┬á┬▒┬á0.1┬á┬▒┬á0.1	2.015┬á┬▒┬á0.3┬á┬▒┬á0.1┬á┬▒┬á0.1
0.145ΓÇô0.165	1.695┬á┬▒┬á0.5┬á┬▒┬á0.1┬á┬▒┬á0.1	1.684┬á┬▒┬á0.5┬á┬▒┬á0.1┬á┬▒┬á0.1	1.678┬á┬▒┬á0.4┬á┬▒┬á0.1┬á┬▒┬á0.1	1.679┬á┬▒┬á0.4┬á┬▒┬á0.1┬á┬▒┬á0.1	1.667┬á┬▒┬á0.4┬á┬▒┬á0.1┬á┬▒┬á0.1	1.673┬á┬▒┬á0.3┬á┬▒┬á0.1┬á┬▒┬á0.1
0.165ΓÇô0.189	1.378┬á┬▒┬á0.5┬á┬▒┬á0.1┬á┬▒┬á0.1	1.368┬á┬▒┬á0.5┬á┬▒┬á0.1┬á┬▒┬á0.1	1.394┬á┬▒┬á0.4┬á┬▒┬á0.1┬á┬▒┬á0.1	1.394┬á┬▒┬á0.4┬á┬▒┬á0.1┬á┬▒┬á0.1	1.385┬á┬▒┬á0.4┬á┬▒┬á0.1┬á┬▒┬á0.1	1.378┬á┬▒┬á0.3┬á┬▒┬á0.1┬á┬▒┬á0.1
0.189ΓÇô0.219	1.106┬á┬▒┬á0.5┬á┬▒┬á0.1┬á┬▒┬á0.1	1.100┬á┬▒┬á0.5┬á┬▒┬á0.1┬á┬▒┬á0.1	1.109┬á┬▒┬á0.4┬á┬▒┬á0.1┬á┬▒┬á0.1	1.110┬á┬▒┬á0.4┬á┬▒┬á0.1┬á┬▒┬á0.1	1.105┬á┬▒┬á0.4┬á┬▒┬á0.1┬á┬▒┬á0.2	1.102┬á┬▒┬á0.3┬á┬▒┬á0.1┬á┬▒┬á0.1
0.219ΓÇô0.258	0.845┬á┬▒┬á0.5┬á┬▒┬á0.1┬á┬▒┬á0.1	0.840┬á┬▒┬á0.5┬á┬▒┬á0.1┬á┬▒┬á0.1	0.843┬á┬▒┬á0.4┬á┬▒┬á0.1┬á┬▒┬á0.1	0.843┬á┬▒┬á0.4┬á┬▒┬á0.1┬á┬▒┬á0.1	0.838┬á┬▒┬á0.4┬á┬▒┬á0.1┬á┬▒┬á0.1	0.839┬á┬▒┬á0.3┬á┬▒┬á0.1┬á┬▒┬á0.1
0.258ΓÇô0.312	0.614┬á┬▒┬á0.5┬á┬▒┬á0.1┬á┬▒┬á0.2	0.611┬á┬▒┬á0.5┬á┬▒┬á0.1┬á┬▒┬á0.2	0.609┬á┬▒┬á0.4┬á┬▒┬á0.1┬á┬▒┬á0.1	0.609┬á┬▒┬á0.4┬á┬▒┬á0.1┬á┬▒┬á0.1	0.606┬á┬▒┬á0.4┬á┬▒┬á0.1┬á┬▒┬á0.2	0.608┬á┬▒┬á0.3┬á┬▒┬á0.1┬á┬▒┬á0.2
0.312ΓÇô0.391	0.409┬á┬▒┬á0.5┬á┬▒┬á0.1┬á┬▒┬á0.3	0.407┬á┬▒┬á0.5┬á┬▒┬á0.1┬á┬▒┬á0.3	0.408┬á┬▒┬á0.4┬á┬▒┬á0.1┬á┬▒┬á0.2	0.408┬á┬▒┬á0.4┬á┬▒┬á0.1┬á┬▒┬á0.2	0.406┬á┬▒┬á0.4┬á┬▒┬á0.1┬á┬▒┬á0.2	0.406┬á┬▒┬á0.3┬á┬▒┬á0.1┬á┬▒┬á0.2
0.391ΓÇô0.524	0.238┬á┬▒┬á0.5┬á┬▒┬á0.1┬á┬▒┬á0.3	0.237┬á┬▒┬á0.5┬á┬▒┬á0.1┬á┬▒┬á0.3	0.236┬á┬▒┬á0.4┬á┬▒┬á0.1┬á┬▒┬á0.2	0.236┬á┬▒┬á0.4┬á┬▒┬á0.1┬á┬▒┬á0.2	0.235┬á┬▒┬á0.4┬á┬▒┬á0.1┬á┬▒┬á0.2	0.236┬á┬▒┬á0.3┬á┬▒┬á0.1┬á┬▒┬á0.2
0.524ΓÇô0.695	0.122┬á┬▒┬á0.6┬á┬▒┬á0.3┬á┬▒┬á0.3	0.122┬á┬▒┬á0.6┬á┬▒┬á0.3┬á┬▒┬á0.3	0.121┬á┬▒┬á0.5┬á┬▒┬á0.1┬á┬▒┬á0.2	0.121┬á┬▒┬á0.5┬á┬▒┬á0.1┬á┬▒┬á0.2	0.121┬á┬▒┬á0.5┬á┬▒┬á0.1┬á┬▒┬á0.3	0.121┬á┬▒┬á0.4┬á┬▒┬á0.1┬á┬▒┬á0.2
0.695ΓÇô0.918	0.0624┬á┬▒┬á0.7┬á┬▒┬á0.3┬á┬▒┬á0.3	0.0622┬á┬▒┬á0.7┬á┬▒┬á0.3┬á┬▒┬á0.3	0.0616┬á┬▒┬á0.6┬á┬▒┬á0.1┬á┬▒┬á0.2	0.0617┬á┬▒┬á0.6┬á┬▒┬á0.1┬á┬▒┬á0.2	0.0615┬á┬▒┬á0.6┬á┬▒┬á0.1┬á┬▒┬á0.3	0.0618┬á┬▒┬á0.5┬á┬▒┬á0.2┬á┬▒┬á0.2
0.918ΓÇô1.153	0.0321┬á┬▒┬á1.0┬á┬▒┬á0.3┬á┬▒┬á0.3	0.0320┬á┬▒┬á1.0┬á┬▒┬á0.3┬á┬▒┬á0.3	0.0323┬á┬▒┬á0.8┬á┬▒┬á0.2┬á┬▒┬á0.2	0.0323┬á┬▒┬á0.8┬á┬▒┬á0.2┬á┬▒┬á0.2	0.0322┬á┬▒┬á0.8┬á┬▒┬á0.2┬á┬▒┬á0.3	0.0321┬á┬▒┬á0.6┬á┬▒┬á0.2┬á┬▒┬á0.2
1.153ΓÇô1.496	0.0165┬á┬▒┬á1.1┬á┬▒┬á0.3┬á┬▒┬á0.4	0.0165┬á┬▒┬á1.1┬á┬▒┬á0.3┬á┬▒┬á0.4	0.0166┬á┬▒┬á1.0┬á┬▒┬á0.2┬á┬▒┬á0.2	0.0167┬á┬▒┬á1.0┬á┬▒┬á0.2┬á┬▒┬á0.2	0.0166┬á┬▒┬á1.0┬á┬▒┬á0.2┬á┬▒┬á0.3	0.0165┬á┬▒┬á0.7┬á┬▒┬á0.2┬á┬▒┬á0.3
1.496ΓÇô1.947	0.00801┬á┬▒┬á1.4┬á┬▒┬á0.4┬á┬▒┬á0.4	0.00797┬á┬▒┬á1.4┬á┬▒┬á0.4┬á┬▒┬á0.4	0.00835┬á┬▒┬á1.2┬á┬▒┬á0.3┬á┬▒┬á0.3	0.00838┬á┬▒┬á1.2┬á┬▒┬á0.3┬á┬▒┬á0.3	0.00833┬á┬▒┬á1.2┬á┬▒┬á0.3┬á┬▒┬á0.3	0.00817┬á┬▒┬á0.9┬á┬▒┬á0.2┬á┬▒┬á0.3
1.947ΓÇô2.522	0.00412┬á┬▒┬á1.7┬á┬▒┬á0.4┬á┬▒┬á0.4	0.00410┬á┬▒┬á1.7┬á┬▒┬á0.4┬á┬▒┬á0.4	0.00406┬á┬▒┬á1.6┬á┬▒┬á0.3┬á┬▒┬á0.4	0.00407┬á┬▒┬á1.6┬á┬▒┬á0.3┬á┬▒┬á0.4	0.00405┬á┬▒┬á1.6┬á┬▒┬á0.3┬á┬▒┬á0.4	0.00407┬á┬▒┬á1.2┬á┬▒┬á0.3┬á┬▒┬á0.3
2.522ΓÇô3.277	0.00210┬á┬▒┬á2.1┬á┬▒┬á0.5┬á┬▒┬á0.5	0.00209┬á┬▒┬á2.1┬á┬▒┬á0.5┬á┬▒┬á0.5	0.00207┬á┬▒┬á1.9┬á┬▒┬á0.4┬á┬▒┬á0.3	0.00208┬á┬▒┬á1.9┬á┬▒┬á0.4┬á┬▒┬á0.3	0.00207┬á┬▒┬á1.9┬á┬▒┬á0.4┬á┬▒┬á0.4	0.00207┬á┬▒┬á1.4┬á┬▒┬á0.3┬á┬▒┬á0.3
3.277ΓÇô5.000	0.000942┬á┬▒┬á2.1┬á┬▒┬á0.5┬á┬▒┬á0.8	0.000940┬á┬▒┬á2.1┬á┬▒┬á0.6┬á┬▒┬á0.8	0.000909┬á┬▒┬á1.8┬á┬▒┬á0.4┬á┬▒┬á0.4	0.000909┬á┬▒┬á1.9┬á┬▒┬á0.4┬á┬▒┬á0.4	0.000909┬á┬▒┬á1.9┬á┬▒┬á0.4┬á┬▒┬á0.4	0.000922┬á┬▒┬á1.4┬á┬▒┬á0.3┬á┬▒┬á0.4
5.000ΓÇô10.000	0.000282┬á┬▒┬á2.3┬á┬▒┬á0.5┬á┬▒┬á0.6	0.000282┬á┬▒┬á2.3┬á┬▒┬á0.5┬á┬▒┬á0.6	0.000274┬á┬▒┬á2.0┬á┬▒┬á0.5┬á┬▒┬á0.4	0.000275┬á┬▒┬á2.0┬á┬▒┬á0.5┬á┬▒┬á0.4	0.000273┬á┬▒┬á2.0┬á┬▒┬á0.5┬á┬▒┬á0.5	0.000276┬á┬▒┬á1.5┬á┬▒┬á0.3┬á┬▒┬á0.4

**Table 11:** The values of $(1/\sigma )\,{\mathrm {d}}\sigma /{\mathrm {d}}\phi ^*_{\eta }$ in each bin of $\phi ^*_{\eta }$ for the electron and muon channels separately (for various particle-level definitions) and for the Born-level combination in the kinematic region $66\ ~{\mathrm {GeV}}\le m_{\ell \ell }< 116\ ~{\mathrm {GeV}},\ 1.2 \le |y_{\ell \ell }| < 1.6$. The associated statistical and systematic (both uncorrelated and correlated between bins of $\phi ^*_{\eta }$) are provided in percentage form

Bin	$(1/\sigma )\,d\sigma /d\phi ^*_{\eta }$ ┬▒ Statistical [%] ┬▒ Uncorrelated systematic [%] ┬▒ Correlated systematic [%]					
	Electron channel		Muon channel			Combination
	Dressed	Born	Bare	Dressed	Born	Born
0.000ΓÇô0.004	9.382┬á┬▒┬á0.5┬á┬▒┬á0.1┬á┬▒┬á0.2	9.470┬á┬▒┬á0.5┬á┬▒┬á0.2┬á┬▒┬á0.2	9.358┬á┬▒┬á0.4┬á┬▒┬á0.1┬á┬▒┬á0.1	9.352┬á┬▒┬á0.4┬á┬▒┬á0.1┬á┬▒┬á0.1	9.433┬á┬▒┬á0.4┬á┬▒┬á0.1┬á┬▒┬á0.2	9.443┬á┬▒┬á0.3┬á┬▒┬á0.1┬á┬▒┬á0.2
0.004ΓÇô0.008	9.317┬á┬▒┬á0.5┬á┬▒┬á0.2┬á┬▒┬á0.2	9.402┬á┬▒┬á0.5┬á┬▒┬á0.2┬á┬▒┬á0.2	9.289┬á┬▒┬á0.4┬á┬▒┬á0.1┬á┬▒┬á0.1	9.281┬á┬▒┬á0.4┬á┬▒┬á0.1┬á┬▒┬á0.1	9.363┬á┬▒┬á0.4┬á┬▒┬á0.1┬á┬▒┬á0.2	9.374┬á┬▒┬á0.3┬á┬▒┬á0.1┬á┬▒┬á0.1
0.008ΓÇô0.012	9.095┬á┬▒┬á0.5┬á┬▒┬á0.2┬á┬▒┬á0.2	9.169┬á┬▒┬á0.5┬á┬▒┬á0.2┬á┬▒┬á0.2	9.101┬á┬▒┬á0.4┬á┬▒┬á0.1┬á┬▒┬á0.0	9.093┬á┬▒┬á0.4┬á┬▒┬á0.1┬á┬▒┬á0.0	9.167┬á┬▒┬á0.4┬á┬▒┬á0.1┬á┬▒┬á0.2	9.165┬á┬▒┬á0.3┬á┬▒┬á0.1┬á┬▒┬á0.1
0.012ΓÇô0.016	8.874┬á┬▒┬á0.5┬á┬▒┬á0.1┬á┬▒┬á0.2	8.945┬á┬▒┬á0.5┬á┬▒┬á0.2┬á┬▒┬á0.2	8.918┬á┬▒┬á0.4┬á┬▒┬á0.1┬á┬▒┬á0.0	8.905┬á┬▒┬á0.4┬á┬▒┬á0.1┬á┬▒┬á0.0	8.999┬á┬▒┬á0.4┬á┬▒┬á0.1┬á┬▒┬á0.2	8.975┬á┬▒┬á0.3┬á┬▒┬á0.1┬á┬▒┬á0.1
0.016ΓÇô0.020	8.552┬á┬▒┬á0.5┬á┬▒┬á0.2┬á┬▒┬á0.2	8.617┬á┬▒┬á0.5┬á┬▒┬á0.2┬á┬▒┬á0.2	8.632┬á┬▒┬á0.4┬á┬▒┬á0.1┬á┬▒┬á0.0	8.629┬á┬▒┬á0.4┬á┬▒┬á0.1┬á┬▒┬á0.0	8.682┬á┬▒┬á0.4┬á┬▒┬á0.1┬á┬▒┬á0.2	8.655┬á┬▒┬á0.3┬á┬▒┬á0.1┬á┬▒┬á0.1
0.020ΓÇô0.024	8.242┬á┬▒┬á0.5┬á┬▒┬á0.2┬á┬▒┬á0.2	8.299┬á┬▒┬á0.5┬á┬▒┬á0.2┬á┬▒┬á0.2	8.265┬á┬▒┬á0.4┬á┬▒┬á0.1┬á┬▒┬á0.0	8.264┬á┬▒┬á0.4┬á┬▒┬á0.1┬á┬▒┬á0.0	8.332┬á┬▒┬á0.4┬á┬▒┬á0.1┬á┬▒┬á0.2	8.317┬á┬▒┬á0.3┬á┬▒┬á0.1┬á┬▒┬á0.1
0.024ΓÇô0.029	7.803┬á┬▒┬á0.5┬á┬▒┬á0.2┬á┬▒┬á0.2	7.841┬á┬▒┬á0.5┬á┬▒┬á0.2┬á┬▒┬á0.2	7.805┬á┬▒┬á0.4┬á┬▒┬á0.1┬á┬▒┬á0.0	7.792┬á┬▒┬á0.4┬á┬▒┬á0.1┬á┬▒┬á0.0	7.835┬á┬▒┬á0.4┬á┬▒┬á0.1┬á┬▒┬á0.2	7.833┬á┬▒┬á0.3┬á┬▒┬á0.1┬á┬▒┬á0.2
0.029ΓÇô0.034	7.312┬á┬▒┬á0.5┬á┬▒┬á0.1┬á┬▒┬á0.2	7.340┬á┬▒┬á0.5┬á┬▒┬á0.2┬á┬▒┬á0.2	7.371┬á┬▒┬á0.4┬á┬▒┬á0.1┬á┬▒┬á0.0	7.363┬á┬▒┬á0.4┬á┬▒┬á0.1┬á┬▒┬á0.0	7.395┬á┬▒┬á0.4┬á┬▒┬á0.1┬á┬▒┬á0.2	7.371┬á┬▒┬á0.3┬á┬▒┬á0.1┬á┬▒┬á0.2
0.034ΓÇô0.039	6.920┬á┬▒┬á0.5┬á┬▒┬á0.2┬á┬▒┬á0.2	6.938┬á┬▒┬á0.5┬á┬▒┬á0.2┬á┬▒┬á0.2	6.907┬á┬▒┬á0.4┬á┬▒┬á0.1┬á┬▒┬á0.0	6.914┬á┬▒┬á0.4┬á┬▒┬á0.1┬á┬▒┬á0.0	6.944┬á┬▒┬á0.4┬á┬▒┬á0.1┬á┬▒┬á0.2	6.939┬á┬▒┬á0.3┬á┬▒┬á0.1┬á┬▒┬á0.2
0.039ΓÇô0.045	6.397┬á┬▒┬á0.5┬á┬▒┬á0.1┬á┬▒┬á0.2	6.413┬á┬▒┬á0.5┬á┬▒┬á0.1┬á┬▒┬á0.2	6.401┬á┬▒┬á0.4┬á┬▒┬á0.1┬á┬▒┬á0.0	6.404┬á┬▒┬á0.4┬á┬▒┬á0.1┬á┬▒┬á0.0	6.427┬á┬▒┬á0.4┬á┬▒┬á0.1┬á┬▒┬á0.2	6.420┬á┬▒┬á0.3┬á┬▒┬á0.1┬á┬▒┬á0.2
0.045ΓÇô0.051	5.877┬á┬▒┬á0.5┬á┬▒┬á0.2┬á┬▒┬á0.1	5.886┬á┬▒┬á0.5┬á┬▒┬á0.2┬á┬▒┬á0.1	5.914┬á┬▒┬á0.4┬á┬▒┬á0.1┬á┬▒┬á0.0	5.908┬á┬▒┬á0.4┬á┬▒┬á0.1┬á┬▒┬á0.0	5.910┬á┬▒┬á0.4┬á┬▒┬á0.1┬á┬▒┬á0.2	5.898┬á┬▒┬á0.3┬á┬▒┬á0.1┬á┬▒┬á0.2
0.051ΓÇô0.057	5.366┬á┬▒┬á0.5┬á┬▒┬á0.2┬á┬▒┬á0.1	5.364┬á┬▒┬á0.5┬á┬▒┬á0.2┬á┬▒┬á0.1	5.417┬á┬▒┬á0.4┬á┬▒┬á0.1┬á┬▒┬á0.0	5.415┬á┬▒┬á0.4┬á┬▒┬á0.1┬á┬▒┬á0.0	5.416┬á┬▒┬á0.4┬á┬▒┬á0.1┬á┬▒┬á0.2	5.394┬á┬▒┬á0.3┬á┬▒┬á0.1┬á┬▒┬á0.2
0.057ΓÇô0.064	4.985┬á┬▒┬á0.5┬á┬▒┬á0.1┬á┬▒┬á0.1	4.985┬á┬▒┬á0.5┬á┬▒┬á0.2┬á┬▒┬á0.1	4.981┬á┬▒┬á0.4┬á┬▒┬á0.1┬á┬▒┬á0.1	4.979┬á┬▒┬á0.4┬á┬▒┬á0.1┬á┬▒┬á0.1	4.982┬á┬▒┬á0.4┬á┬▒┬á0.1┬á┬▒┬á0.2	4.980┬á┬▒┬á0.3┬á┬▒┬á0.1┬á┬▒┬á0.2
0.064ΓÇô0.072	4.526┬á┬▒┬á0.5┬á┬▒┬á0.1┬á┬▒┬á0.1	4.519┬á┬▒┬á0.5┬á┬▒┬á0.1┬á┬▒┬á0.1	4.524┬á┬▒┬á0.4┬á┬▒┬á0.1┬á┬▒┬á0.1	4.521┬á┬▒┬á0.4┬á┬▒┬á0.1┬á┬▒┬á0.1	4.503┬á┬▒┬á0.4┬á┬▒┬á0.1┬á┬▒┬á0.2	4.506┬á┬▒┬á0.3┬á┬▒┬á0.1┬á┬▒┬á0.2
0.072ΓÇô0.081	4.049┬á┬▒┬á0.5┬á┬▒┬á0.2┬á┬▒┬á0.1	4.037┬á┬▒┬á0.5┬á┬▒┬á0.2┬á┬▒┬á0.1	4.071┬á┬▒┬á0.4┬á┬▒┬á0.1┬á┬▒┬á0.1	4.071┬á┬▒┬á0.4┬á┬▒┬á0.1┬á┬▒┬á0.1	4.059┬á┬▒┬á0.4┬á┬▒┬á0.1┬á┬▒┬á0.2	4.049┬á┬▒┬á0.3┬á┬▒┬á0.1┬á┬▒┬á0.1
0.081ΓÇô0.091	3.593┬á┬▒┬á0.5┬á┬▒┬á0.2┬á┬▒┬á0.1	3.576┬á┬▒┬á0.5┬á┬▒┬á0.2┬á┬▒┬á0.1	3.590┬á┬▒┬á0.4┬á┬▒┬á0.1┬á┬▒┬á0.1	3.587┬á┬▒┬á0.4┬á┬▒┬á0.1┬á┬▒┬á0.1	3.571┬á┬▒┬á0.4┬á┬▒┬á0.1┬á┬▒┬á0.2	3.570┬á┬▒┬á0.3┬á┬▒┬á0.1┬á┬▒┬á0.1
0.091ΓÇô0.102	3.196┬á┬▒┬á0.5┬á┬▒┬á0.2┬á┬▒┬á0.1	3.182┬á┬▒┬á0.5┬á┬▒┬á0.2┬á┬▒┬á0.1	3.159┬á┬▒┬á0.4┬á┬▒┬á0.1┬á┬▒┬á0.1	3.158┬á┬▒┬á0.4┬á┬▒┬á0.1┬á┬▒┬á0.1	3.142┬á┬▒┬á0.4┬á┬▒┬á0.1┬á┬▒┬á0.2	3.154┬á┬▒┬á0.3┬á┬▒┬á0.1┬á┬▒┬á0.1
0.102ΓÇô0.114	2.756┬á┬▒┬á0.5┬á┬▒┬á0.2┬á┬▒┬á0.1	2.742┬á┬▒┬á0.5┬á┬▒┬á0.2┬á┬▒┬á0.1	2.781┬á┬▒┬á0.4┬á┬▒┬á0.1┬á┬▒┬á0.1	2.784┬á┬▒┬á0.4┬á┬▒┬á0.1┬á┬▒┬á0.1	2.769┬á┬▒┬á0.4┬á┬▒┬á0.1┬á┬▒┬á0.2	2.757┬á┬▒┬á0.3┬á┬▒┬á0.1┬á┬▒┬á0.1
0.114ΓÇô0.128	2.403┬á┬▒┬á0.5┬á┬▒┬á0.2┬á┬▒┬á0.1	2.388┬á┬▒┬á0.5┬á┬▒┬á0.2┬á┬▒┬á0.1	2.418┬á┬▒┬á0.4┬á┬▒┬á0.1┬á┬▒┬á0.1	2.418┬á┬▒┬á0.4┬á┬▒┬á0.1┬á┬▒┬á0.1	2.403┬á┬▒┬á0.4┬á┬▒┬á0.1┬á┬▒┬á0.1	2.396┬á┬▒┬á0.3┬á┬▒┬á0.1┬á┬▒┬á0.1
0.128ΓÇô0.145	2.065┬á┬▒┬á0.5┬á┬▒┬á0.2┬á┬▒┬á0.1	2.053┬á┬▒┬á0.5┬á┬▒┬á0.2┬á┬▒┬á0.1	2.045┬á┬▒┬á0.4┬á┬▒┬á0.1┬á┬▒┬á0.1	2.045┬á┬▒┬á0.4┬á┬▒┬á0.1┬á┬▒┬á0.1	2.032┬á┬▒┬á0.4┬á┬▒┬á0.1┬á┬▒┬á0.1	2.039┬á┬▒┬á0.3┬á┬▒┬á0.1┬á┬▒┬á0.1
0.145ΓÇô0.165	1.715┬á┬▒┬á0.5┬á┬▒┬á0.2┬á┬▒┬á0.1	1.705┬á┬▒┬á0.5┬á┬▒┬á0.2┬á┬▒┬á0.1	1.709┬á┬▒┬á0.4┬á┬▒┬á0.1┬á┬▒┬á0.1	1.711┬á┬▒┬á0.4┬á┬▒┬á0.1┬á┬▒┬á0.1	1.700┬á┬▒┬á0.4┬á┬▒┬á0.1┬á┬▒┬á0.1	1.701┬á┬▒┬á0.3┬á┬▒┬á0.1┬á┬▒┬á0.1
0.165ΓÇô0.189	1.404┬á┬▒┬á0.5┬á┬▒┬á0.2┬á┬▒┬á0.1	1.395┬á┬▒┬á0.5┬á┬▒┬á0.2┬á┬▒┬á0.1	1.403┬á┬▒┬á0.4┬á┬▒┬á0.1┬á┬▒┬á0.1	1.404┬á┬▒┬á0.4┬á┬▒┬á0.1┬á┬▒┬á0.1	1.394┬á┬▒┬á0.4┬á┬▒┬á0.1┬á┬▒┬á0.1	1.394┬á┬▒┬á0.3┬á┬▒┬á0.1┬á┬▒┬á0.1
0.189ΓÇô0.219	1.117┬á┬▒┬á0.5┬á┬▒┬á0.3┬á┬▒┬á0.2	1.110┬á┬▒┬á0.5┬á┬▒┬á0.3┬á┬▒┬á0.2	1.123┬á┬▒┬á0.4┬á┬▒┬á0.1┬á┬▒┬á0.1	1.124┬á┬▒┬á0.4┬á┬▒┬á0.1┬á┬▒┬á0.1	1.118┬á┬▒┬á0.4┬á┬▒┬á0.1┬á┬▒┬á0.1	1.115┬á┬▒┬á0.3┬á┬▒┬á0.1┬á┬▒┬á0.1
0.219ΓÇô0.258	0.851┬á┬▒┬á0.5┬á┬▒┬á0.3┬á┬▒┬á0.2	0.846┬á┬▒┬á0.5┬á┬▒┬á0.3┬á┬▒┬á0.2	0.856┬á┬▒┬á0.4┬á┬▒┬á0.1┬á┬▒┬á0.1	0.856┬á┬▒┬á0.4┬á┬▒┬á0.1┬á┬▒┬á0.1	0.852┬á┬▒┬á0.4┬á┬▒┬á0.1┬á┬▒┬á0.1	0.850┬á┬▒┬á0.3┬á┬▒┬á0.1┬á┬▒┬á0.1
0.258ΓÇô0.312	0.621┬á┬▒┬á0.5┬á┬▒┬á0.2┬á┬▒┬á0.3	0.618┬á┬▒┬á0.5┬á┬▒┬á0.2┬á┬▒┬á0.3	0.622┬á┬▒┬á0.4┬á┬▒┬á0.1┬á┬▒┬á0.1	0.623┬á┬▒┬á0.4┬á┬▒┬á0.1┬á┬▒┬á0.1	0.620┬á┬▒┬á0.4┬á┬▒┬á0.1┬á┬▒┬á0.2	0.618┬á┬▒┬á0.3┬á┬▒┬á0.1┬á┬▒┬á0.2
0.312ΓÇô0.391	0.415┬á┬▒┬á0.5┬á┬▒┬á0.2┬á┬▒┬á0.3	0.413┬á┬▒┬á0.5┬á┬▒┬á0.2┬á┬▒┬á0.4	0.409┬á┬▒┬á0.4┬á┬▒┬á0.1┬á┬▒┬á0.1	0.409┬á┬▒┬á0.4┬á┬▒┬á0.1┬á┬▒┬á0.1	0.408┬á┬▒┬á0.4┬á┬▒┬á0.1┬á┬▒┬á0.2	0.409┬á┬▒┬á0.3┬á┬▒┬á0.1┬á┬▒┬á0.2
0.391ΓÇô0.524	0.238┬á┬▒┬á0.5┬á┬▒┬á0.1┬á┬▒┬á0.3	0.238┬á┬▒┬á0.5┬á┬▒┬á0.1┬á┬▒┬á0.3	0.237┬á┬▒┬á0.4┬á┬▒┬á0.1┬á┬▒┬á0.1	0.237┬á┬▒┬á0.4┬á┬▒┬á0.1┬á┬▒┬á0.1	0.237┬á┬▒┬á0.4┬á┬▒┬á0.1┬á┬▒┬á0.2	0.237┬á┬▒┬á0.3┬á┬▒┬á0.1┬á┬▒┬á0.2
0.524ΓÇô0.695	0.124┬á┬▒┬á0.7┬á┬▒┬á0.4┬á┬▒┬á0.3	0.124┬á┬▒┬á0.7┬á┬▒┬á0.4┬á┬▒┬á0.3	0.122┬á┬▒┬á0.5┬á┬▒┬á0.2┬á┬▒┬á0.2	0.122┬á┬▒┬á0.5┬á┬▒┬á0.2┬á┬▒┬á0.2	0.122┬á┬▒┬á0.5┬á┬▒┬á0.2┬á┬▒┬á0.2	0.122┬á┬▒┬á0.4┬á┬▒┬á0.2┬á┬▒┬á0.2
0.695ΓÇô0.918	0.0615┬á┬▒┬á0.8┬á┬▒┬á0.4┬á┬▒┬á0.4	0.0614┬á┬▒┬á0.8┬á┬▒┬á0.4┬á┬▒┬á0.4	0.0609┬á┬▒┬á0.7┬á┬▒┬á0.2┬á┬▒┬á0.1	0.0610┬á┬▒┬á0.7┬á┬▒┬á0.2┬á┬▒┬á0.1	0.0609┬á┬▒┬á0.7┬á┬▒┬á0.2┬á┬▒┬á0.2	0.0610┬á┬▒┬á0.5┬á┬▒┬á0.2┬á┬▒┬á0.2
0.918ΓÇô1.153	0.0313┬á┬▒┬á1.1┬á┬▒┬á0.5┬á┬▒┬á0.4	0.0312┬á┬▒┬á1.1┬á┬▒┬á0.5┬á┬▒┬á0.4	0.0310┬á┬▒┬á0.9┬á┬▒┬á0.2┬á┬▒┬á0.2	0.0311┬á┬▒┬á0.9┬á┬▒┬á0.2┬á┬▒┬á0.2	0.0310┬á┬▒┬á0.9┬á┬▒┬á0.2┬á┬▒┬á0.2	0.0310┬á┬▒┬á0.7┬á┬▒┬á0.2┬á┬▒┬á0.2
1.153ΓÇô1.496	0.0163┬á┬▒┬á1.3┬á┬▒┬á0.3┬á┬▒┬á0.5	0.0163┬á┬▒┬á1.3┬á┬▒┬á0.3┬á┬▒┬á0.5	0.0158┬á┬▒┬á1.1┬á┬▒┬á0.3┬á┬▒┬á0.2	0.0158┬á┬▒┬á1.1┬á┬▒┬á0.3┬á┬▒┬á0.2	0.0158┬á┬▒┬á1.1┬á┬▒┬á0.3┬á┬▒┬á0.4	0.0160┬á┬▒┬á0.8┬á┬▒┬á0.2┬á┬▒┬á0.3
1.496ΓÇô1.947	0.00752┬á┬▒┬á1.6┬á┬▒┬á0.4┬á┬▒┬á0.5	0.00749┬á┬▒┬á1.6┬á┬▒┬á0.4┬á┬▒┬á0.5	0.00740┬á┬▒┬á1.3┬á┬▒┬á0.3┬á┬▒┬á0.3	0.00741┬á┬▒┬á1.3┬á┬▒┬á0.3┬á┬▒┬á0.3	0.00737┬á┬▒┬á1.3┬á┬▒┬á0.3┬á┬▒┬á0.5	0.00740┬á┬▒┬á1.0┬á┬▒┬á0.2┬á┬▒┬á0.3
1.947ΓÇô2.522	0.00356┬á┬▒┬á2.0┬á┬▒┬á0.5┬á┬▒┬á0.6	0.00354┬á┬▒┬á2.0┬á┬▒┬á0.5┬á┬▒┬á0.6	0.00356┬á┬▒┬á1.7┬á┬▒┬á0.4┬á┬▒┬á0.4	0.00357┬á┬▒┬á1.7┬á┬▒┬á0.4┬á┬▒┬á0.4	0.00357┬á┬▒┬á1.7┬á┬▒┬á0.4┬á┬▒┬á0.5	0.00355┬á┬▒┬á1.3┬á┬▒┬á0.3┬á┬▒┬á0.4
2.522ΓÇô3.277	0.00168┬á┬▒┬á2.5┬á┬▒┬á0.6┬á┬▒┬á0.6	0.00168┬á┬▒┬á2.5┬á┬▒┬á0.6┬á┬▒┬á0.6	0.00175┬á┬▒┬á2.1┬á┬▒┬á0.4┬á┬▒┬á0.6	0.00176┬á┬▒┬á2.1┬á┬▒┬á0.4┬á┬▒┬á0.6	0.00175┬á┬▒┬á2.1┬á┬▒┬á0.4┬á┬▒┬á0.7	0.00171┬á┬▒┬á1.6┬á┬▒┬á0.4┬á┬▒┬á0.4
3.277ΓÇô5.000	0.000769┬á┬▒┬á2.4┬á┬▒┬á0.6┬á┬▒┬á0.6	0.000768┬á┬▒┬á2.4┬á┬▒┬á0.6┬á┬▒┬á0.6	0.000792┬á┬▒┬á2.1┬á┬▒┬á0.7┬á┬▒┬á0.5	0.000796┬á┬▒┬á2.1┬á┬▒┬á0.7┬á┬▒┬á0.5	0.000795┬á┬▒┬á2.1┬á┬▒┬á0.7┬á┬▒┬á0.6	0.000781┬á┬▒┬á1.6┬á┬▒┬á0.5┬á┬▒┬á0.4
5.000ΓÇô10.000	0.000215┬á┬▒┬á2.7┬á┬▒┬á0.8┬á┬▒┬á0.7	0.000215┬á┬▒┬á2.7┬á┬▒┬á0.8┬á┬▒┬á0.7	0.000213┬á┬▒┬á2.4┬á┬▒┬á0.5┬á┬▒┬á0.4	0.000213┬á┬▒┬á2.4┬á┬▒┬á0.5┬á┬▒┬á0.4	0.000213┬á┬▒┬á2.4┬á┬▒┬á0.5┬á┬▒┬á0.6	0.000213┬á┬▒┬á1.8┬á┬▒┬á0.4┬á┬▒┬á0.4

**Table 12:** The values of $(1/\sigma )\,{\mathrm {d}}\sigma /{\mathrm {d}}\phi ^*_{\eta }$ in each bin of $\phi ^*_{\eta }$ for the electron and muon channels separately (for various particle-level definitions) and for the Born-level combination in the kinematic region $66\ ~{\mathrm {GeV}}\le m_{\ell \ell }< 116\ ~{\mathrm {GeV}},\ 1.6 \le |y_{\ell \ell }| < 2.0$. The associated statistical and systematic (both uncorrelated and correlated between bins of $\phi ^*_{\eta }$) are provided in percentage form

Bin	$(1/\sigma )\,d\sigma /d\phi ^*_{\eta }$ ┬▒ Statistical [%] ┬▒ Uncorrelated systematic [%] ┬▒ Correlated systematic [%]					
	Electron channel		Muon channel			Combination
	Dressed	Born	Bare	Dressed	Born	Born
0.000ΓÇô0.004	9.378┬á┬▒┬á0.6┬á┬▒┬á0.3┬á┬▒┬á0.3	9.472┬á┬▒┬á0.6┬á┬▒┬á0.3┬á┬▒┬á0.3	9.338┬á┬▒┬á0.5┬á┬▒┬á0.1┬á┬▒┬á0.1	9.325┬á┬▒┬á0.5┬á┬▒┬á0.1┬á┬▒┬á0.1	9.405┬á┬▒┬á0.5┬á┬▒┬á0.1┬á┬▒┬á0.2	9.418┬á┬▒┬á0.4┬á┬▒┬á0.1┬á┬▒┬á0.1
0.004ΓÇô0.008	9.264┬á┬▒┬á0.6┬á┬▒┬á0.2┬á┬▒┬á0.2	9.368┬á┬▒┬á0.6┬á┬▒┬á0.2┬á┬▒┬á0.2	9.273┬á┬▒┬á0.5┬á┬▒┬á0.1┬á┬▒┬á0.0	9.274┬á┬▒┬á0.5┬á┬▒┬á0.1┬á┬▒┬á0.0	9.347┬á┬▒┬á0.5┬á┬▒┬á0.1┬á┬▒┬á0.1	9.345┬á┬▒┬á0.4┬á┬▒┬á0.1┬á┬▒┬á0.1
0.008ΓÇô0.012	9.014┬á┬▒┬á0.6┬á┬▒┬á0.2┬á┬▒┬á0.2	9.077┬á┬▒┬á0.6┬á┬▒┬á0.2┬á┬▒┬á0.3	9.131┬á┬▒┬á0.5┬á┬▒┬á0.1┬á┬▒┬á0.0	9.130┬á┬▒┬á0.5┬á┬▒┬á0.1┬á┬▒┬á0.0	9.199┬á┬▒┬á0.5┬á┬▒┬á0.1┬á┬▒┬á0.1	9.153┬á┬▒┬á0.4┬á┬▒┬á0.1┬á┬▒┬á0.1
0.012ΓÇô0.016	8.875┬á┬▒┬á0.7┬á┬▒┬á0.2┬á┬▒┬á0.2	8.951┬á┬▒┬á0.7┬á┬▒┬á0.2┬á┬▒┬á0.2	8.864┬á┬▒┬á0.5┬á┬▒┬á0.1┬á┬▒┬á0.0	8.855┬á┬▒┬á0.5┬á┬▒┬á0.1┬á┬▒┬á0.0	8.930┬á┬▒┬á0.5┬á┬▒┬á0.1┬á┬▒┬á0.1	8.928┬á┬▒┬á0.4┬á┬▒┬á0.1┬á┬▒┬á0.1
0.016ΓÇô0.020	8.592┬á┬▒┬á0.7┬á┬▒┬á0.2┬á┬▒┬á0.2	8.657┬á┬▒┬á0.7┬á┬▒┬á0.2┬á┬▒┬á0.2	8.503┬á┬▒┬á0.5┬á┬▒┬á0.1┬á┬▒┬á0.0	8.493┬á┬▒┬á0.5┬á┬▒┬á0.1┬á┬▒┬á0.0	8.561┬á┬▒┬á0.5┬á┬▒┬á0.1┬á┬▒┬á0.1	8.588┬á┬▒┬á0.4┬á┬▒┬á0.1┬á┬▒┬á0.1
0.020ΓÇô0.024	8.207┬á┬▒┬á0.7┬á┬▒┬á0.2┬á┬▒┬á0.2	8.259┬á┬▒┬á0.7┬á┬▒┬á0.2┬á┬▒┬á0.2	8.149┬á┬▒┬á0.5┬á┬▒┬á0.1┬á┬▒┬á0.1	8.143┬á┬▒┬á0.5┬á┬▒┬á0.1┬á┬▒┬á0.1	8.195┬á┬▒┬á0.5┬á┬▒┬á0.1┬á┬▒┬á0.2	8.211┬á┬▒┬á0.4┬á┬▒┬á0.1┬á┬▒┬á0.1
0.024ΓÇô0.029	7.721┬á┬▒┬á0.6┬á┬▒┬á0.2┬á┬▒┬á0.2	7.755┬á┬▒┬á0.6┬á┬▒┬á0.2┬á┬▒┬á0.2	7.776┬á┬▒┬á0.5┬á┬▒┬á0.1┬á┬▒┬á0.0	7.769┬á┬▒┬á0.5┬á┬▒┬á0.1┬á┬▒┬á0.0	7.842┬á┬▒┬á0.5┬á┬▒┬á0.1┬á┬▒┬á0.3	7.804┬á┬▒┬á0.4┬á┬▒┬á0.1┬á┬▒┬á0.2
0.029ΓÇô0.034	7.439┬á┬▒┬á0.6┬á┬▒┬á0.2┬á┬▒┬á0.2	7.484┬á┬▒┬á0.6┬á┬▒┬á0.2┬á┬▒┬á0.3	7.343┬á┬▒┬á0.5┬á┬▒┬á0.1┬á┬▒┬á0.0	7.345┬á┬▒┬á0.5┬á┬▒┬á0.1┬á┬▒┬á0.0	7.398┬á┬▒┬á0.5┬á┬▒┬á0.1┬á┬▒┬á0.3	7.416┬á┬▒┬á0.4┬á┬▒┬á0.1┬á┬▒┬á0.2
0.034ΓÇô0.039	6.755┬á┬▒┬á0.7┬á┬▒┬á0.2┬á┬▒┬á0.2	6.765┬á┬▒┬á0.7┬á┬▒┬á0.3┬á┬▒┬á0.3	6.832┬á┬▒┬á0.5┬á┬▒┬á0.1┬á┬▒┬á0.1	6.824┬á┬▒┬á0.5┬á┬▒┬á0.1┬á┬▒┬á0.1	6.848┬á┬▒┬á0.5┬á┬▒┬á0.1┬á┬▒┬á0.3	6.813┬á┬▒┬á0.4┬á┬▒┬á0.1┬á┬▒┬á0.2
0.039ΓÇô0.045	6.394┬á┬▒┬á0.6┬á┬▒┬á0.2┬á┬▒┬á0.2	6.414┬á┬▒┬á0.6┬á┬▒┬á0.2┬á┬▒┬á0.2	6.377┬á┬▒┬á0.5┬á┬▒┬á0.1┬á┬▒┬á0.0	6.375┬á┬▒┬á0.5┬á┬▒┬á0.1┬á┬▒┬á0.0	6.383┬á┬▒┬á0.5┬á┬▒┬á0.1┬á┬▒┬á0.3	6.383┬á┬▒┬á0.4┬á┬▒┬á0.1┬á┬▒┬á0.2
0.045ΓÇô0.051	5.878┬á┬▒┬á0.7┬á┬▒┬á0.2┬á┬▒┬á0.2	5.881┬á┬▒┬á0.7┬á┬▒┬á0.2┬á┬▒┬á0.2	5.906┬á┬▒┬á0.5┬á┬▒┬á0.1┬á┬▒┬á0.1	5.903┬á┬▒┬á0.5┬á┬▒┬á0.1┬á┬▒┬á0.1	5.916┬á┬▒┬á0.5┬á┬▒┬á0.1┬á┬▒┬á0.3	5.895┬á┬▒┬á0.4┬á┬▒┬á0.1┬á┬▒┬á0.2
0.051ΓÇô0.057	5.309┬á┬▒┬á0.7┬á┬▒┬á0.2┬á┬▒┬á0.2	5.302┬á┬▒┬á0.7┬á┬▒┬á0.2┬á┬▒┬á0.2	5.371┬á┬▒┬á0.5┬á┬▒┬á0.1┬á┬▒┬á0.0	5.370┬á┬▒┬á0.5┬á┬▒┬á0.1┬á┬▒┬á0.0	5.374┬á┬▒┬á0.5┬á┬▒┬á0.1┬á┬▒┬á0.3	5.344┬á┬▒┬á0.4┬á┬▒┬á0.1┬á┬▒┬á0.2
0.057ΓÇô0.064	4.899┬á┬▒┬á0.7┬á┬▒┬á0.2┬á┬▒┬á0.2	4.896┬á┬▒┬á0.7┬á┬▒┬á0.2┬á┬▒┬á0.2	4.909┬á┬▒┬á0.5┬á┬▒┬á0.1┬á┬▒┬á0.1	4.907┬á┬▒┬á0.5┬á┬▒┬á0.1┬á┬▒┬á0.1	4.900┬á┬▒┬á0.5┬á┬▒┬á0.1┬á┬▒┬á0.2	4.893┬á┬▒┬á0.4┬á┬▒┬á0.1┬á┬▒┬á0.1
0.064ΓÇô0.072	4.508┬á┬▒┬á0.6┬á┬▒┬á0.2┬á┬▒┬á0.2	4.503┬á┬▒┬á0.6┬á┬▒┬á0.2┬á┬▒┬á0.2	4.511┬á┬▒┬á0.5┬á┬▒┬á0.1┬á┬▒┬á0.1	4.507┬á┬▒┬á0.5┬á┬▒┬á0.1┬á┬▒┬á0.1	4.498┬á┬▒┬á0.5┬á┬▒┬á0.1┬á┬▒┬á0.2	4.495┬á┬▒┬á0.4┬á┬▒┬á0.1┬á┬▒┬á0.1
0.072ΓÇô0.081	4.011┬á┬▒┬á0.6┬á┬▒┬á0.4┬á┬▒┬á0.2	3.996┬á┬▒┬á0.6┬á┬▒┬á0.4┬á┬▒┬á0.2	4.008┬á┬▒┬á0.5┬á┬▒┬á0.1┬á┬▒┬á0.1	4.005┬á┬▒┬á0.5┬á┬▒┬á0.1┬á┬▒┬á0.1	3.990┬á┬▒┬á0.5┬á┬▒┬á0.1┬á┬▒┬á0.2	3.988┬á┬▒┬á0.4┬á┬▒┬á0.1┬á┬▒┬á0.1
0.081ΓÇô0.091	3.574┬á┬▒┬á0.7┬á┬▒┬á0.4┬á┬▒┬á0.2	3.561┬á┬▒┬á0.7┬á┬▒┬á0.4┬á┬▒┬á0.2	3.574┬á┬▒┬á0.5┬á┬▒┬á0.1┬á┬▒┬á0.1	3.575┬á┬▒┬á0.5┬á┬▒┬á0.1┬á┬▒┬á0.1	3.558┬á┬▒┬á0.5┬á┬▒┬á0.1┬á┬▒┬á0.2	3.556┬á┬▒┬á0.4┬á┬▒┬á0.1┬á┬▒┬á0.1
0.091ΓÇô0.102	3.159┬á┬▒┬á0.7┬á┬▒┬á0.2┬á┬▒┬á0.2	3.142┬á┬▒┬á0.7┬á┬▒┬á0.2┬á┬▒┬á0.2	3.157┬á┬▒┬á0.5┬á┬▒┬á0.1┬á┬▒┬á0.1	3.155┬á┬▒┬á0.5┬á┬▒┬á0.1┬á┬▒┬á0.1	3.130┬á┬▒┬á0.5┬á┬▒┬á0.1┬á┬▒┬á0.2	3.131┬á┬▒┬á0.4┬á┬▒┬á0.1┬á┬▒┬á0.1
0.102ΓÇô0.114	2.806┬á┬▒┬á0.7┬á┬▒┬á0.2┬á┬▒┬á0.1	2.793┬á┬▒┬á0.7┬á┬▒┬á0.2┬á┬▒┬á0.1	2.774┬á┬▒┬á0.5┬á┬▒┬á0.1┬á┬▒┬á0.1	2.776┬á┬▒┬á0.5┬á┬▒┬á0.1┬á┬▒┬á0.1	2.764┬á┬▒┬á0.5┬á┬▒┬á0.1┬á┬▒┬á0.2	2.772┬á┬▒┬á0.4┬á┬▒┬á0.1┬á┬▒┬á0.1
0.114ΓÇô0.128	2.407┬á┬▒┬á0.7┬á┬▒┬á0.2┬á┬▒┬á0.1	2.392┬á┬▒┬á0.7┬á┬▒┬á0.2┬á┬▒┬á0.1	2.416┬á┬▒┬á0.5┬á┬▒┬á0.1┬á┬▒┬á0.1	2.416┬á┬▒┬á0.5┬á┬▒┬á0.1┬á┬▒┬á0.1	2.401┬á┬▒┬á0.5┬á┬▒┬á0.1┬á┬▒┬á0.2	2.396┬á┬▒┬á0.4┬á┬▒┬á0.1┬á┬▒┬á0.1
0.128ΓÇô0.145	2.072┬á┬▒┬á0.7┬á┬▒┬á0.2┬á┬▒┬á0.1	2.058┬á┬▒┬á0.7┬á┬▒┬á0.2┬á┬▒┬á0.1	2.061┬á┬▒┬á0.5┬á┬▒┬á0.1┬á┬▒┬á0.1	2.061┬á┬▒┬á0.5┬á┬▒┬á0.1┬á┬▒┬á0.1	2.048┬á┬▒┬á0.5┬á┬▒┬á0.1┬á┬▒┬á0.2	2.050┬á┬▒┬á0.4┬á┬▒┬á0.1┬á┬▒┬á0.1
0.145ΓÇô0.165	1.730┬á┬▒┬á0.7┬á┬▒┬á0.2┬á┬▒┬á0.2	1.716┬á┬▒┬á0.7┬á┬▒┬á0.2┬á┬▒┬á0.2	1.729┬á┬▒┬á0.5┬á┬▒┬á0.1┬á┬▒┬á0.1	1.730┬á┬▒┬á0.5┬á┬▒┬á0.1┬á┬▒┬á0.1	1.718┬á┬▒┬á0.5┬á┬▒┬á0.1┬á┬▒┬á0.2	1.717┬á┬▒┬á0.4┬á┬▒┬á0.1┬á┬▒┬á0.1
0.165ΓÇô0.189	1.417┬á┬▒┬á0.7┬á┬▒┬á0.2┬á┬▒┬á0.2	1.408┬á┬▒┬á0.7┬á┬▒┬á0.2┬á┬▒┬á0.2	1.423┬á┬▒┬á0.5┬á┬▒┬á0.1┬á┬▒┬á0.1	1.422┬á┬▒┬á0.5┬á┬▒┬á0.1┬á┬▒┬á0.1	1.414┬á┬▒┬á0.5┬á┬▒┬á0.1┬á┬▒┬á0.2	1.411┬á┬▒┬á0.4┬á┬▒┬á0.1┬á┬▒┬á0.1
0.189ΓÇô0.219	1.137┬á┬▒┬á0.7┬á┬▒┬á0.4┬á┬▒┬á0.3	1.130┬á┬▒┬á0.7┬á┬▒┬á0.4┬á┬▒┬á0.3	1.138┬á┬▒┬á0.5┬á┬▒┬á0.1┬á┬▒┬á0.1	1.139┬á┬▒┬á0.5┬á┬▒┬á0.1┬á┬▒┬á0.1	1.133┬á┬▒┬á0.5┬á┬▒┬á0.1┬á┬▒┬á0.2	1.131┬á┬▒┬á0.4┬á┬▒┬á0.1┬á┬▒┬á0.2
0.219ΓÇô0.258	0.871┬á┬▒┬á0.7┬á┬▒┬á0.4┬á┬▒┬á0.3	0.866┬á┬▒┬á0.7┬á┬▒┬á0.4┬á┬▒┬á0.3	0.875┬á┬▒┬á0.5┬á┬▒┬á0.1┬á┬▒┬á0.1	0.876┬á┬▒┬á0.5┬á┬▒┬á0.1┬á┬▒┬á0.1	0.870┬á┬▒┬á0.5┬á┬▒┬á0.1┬á┬▒┬á0.2	0.869┬á┬▒┬á0.4┬á┬▒┬á0.1┬á┬▒┬á0.2
0.258ΓÇô0.312	0.643┬á┬▒┬á0.7┬á┬▒┬á0.2┬á┬▒┬á0.4	0.641┬á┬▒┬á0.7┬á┬▒┬á0.3┬á┬▒┬á0.4	0.634┬á┬▒┬á0.5┬á┬▒┬á0.1┬á┬▒┬á0.1	0.635┬á┬▒┬á0.5┬á┬▒┬á0.1┬á┬▒┬á0.1	0.631┬á┬▒┬á0.5┬á┬▒┬á0.1┬á┬▒┬á0.2	0.634┬á┬▒┬á0.4┬á┬▒┬á0.1┬á┬▒┬á0.2
0.312ΓÇô0.391	0.428┬á┬▒┬á0.7┬á┬▒┬á0.3┬á┬▒┬á0.4	0.426┬á┬▒┬á0.7┬á┬▒┬á0.3┬á┬▒┬á0.4	0.427┬á┬▒┬á0.5┬á┬▒┬á0.1┬á┬▒┬á0.1	0.427┬á┬▒┬á0.5┬á┬▒┬á0.1┬á┬▒┬á0.1	0.425┬á┬▒┬á0.5┬á┬▒┬á0.1┬á┬▒┬á0.2	0.425┬á┬▒┬á0.4┬á┬▒┬á0.1┬á┬▒┬á0.2
0.391ΓÇô0.524	0.244┬á┬▒┬á0.7┬á┬▒┬á0.3┬á┬▒┬á0.5	0.244┬á┬▒┬á0.7┬á┬▒┬á0.3┬á┬▒┬á0.5	0.245┬á┬▒┬á0.5┬á┬▒┬á0.1┬á┬▒┬á0.1	0.246┬á┬▒┬á0.5┬á┬▒┬á0.1┬á┬▒┬á0.1	0.245┬á┬▒┬á0.5┬á┬▒┬á0.1┬á┬▒┬á0.2	0.245┬á┬▒┬á0.4┬á┬▒┬á0.1┬á┬▒┬á0.2
0.524ΓÇô0.695	0.125┬á┬▒┬á0.8┬á┬▒┬á0.4┬á┬▒┬á0.6	0.124┬á┬▒┬á0.8┬á┬▒┬á0.4┬á┬▒┬á0.6	0.126┬á┬▒┬á0.6┬á┬▒┬á0.1┬á┬▒┬á0.2	0.127┬á┬▒┬á0.6┬á┬▒┬á0.1┬á┬▒┬á0.2	0.126┬á┬▒┬á0.6┬á┬▒┬á0.1┬á┬▒┬á0.2	0.126┬á┬▒┬á0.5┬á┬▒┬á0.2┬á┬▒┬á0.2
0.695ΓÇô0.918	0.0621┬á┬▒┬á1.0┬á┬▒┬á0.4┬á┬▒┬á0.7	0.0620┬á┬▒┬á1.0┬á┬▒┬á0.4┬á┬▒┬á0.7	0.0613┬á┬▒┬á0.8┬á┬▒┬á0.2┬á┬▒┬á0.2	0.0615┬á┬▒┬á0.8┬á┬▒┬á0.2┬á┬▒┬á0.2	0.0615┬á┬▒┬á0.8┬á┬▒┬á0.2┬á┬▒┬á0.3	0.0616┬á┬▒┬á0.6┬á┬▒┬á0.2┬á┬▒┬á0.3
0.918ΓÇô1.153	0.0305┬á┬▒┬á1.4┬á┬▒┬á0.5┬á┬▒┬á0.7	0.0304┬á┬▒┬á1.4┬á┬▒┬á0.5┬á┬▒┬á0.7	0.0301┬á┬▒┬á1.1┬á┬▒┬á0.2┬á┬▒┬á0.2	0.0301┬á┬▒┬á1.1┬á┬▒┬á0.2┬á┬▒┬á0.2	0.0300┬á┬▒┬á1.1┬á┬▒┬á0.2┬á┬▒┬á0.3	0.0301┬á┬▒┬á0.9┬á┬▒┬á0.2┬á┬▒┬á0.3
1.153ΓÇô1.496	0.0148┬á┬▒┬á1.6┬á┬▒┬á0.6┬á┬▒┬á0.8	0.0148┬á┬▒┬á1.6┬á┬▒┬á0.6┬á┬▒┬á0.8	0.0149┬á┬▒┬á1.3┬á┬▒┬á0.2┬á┬▒┬á0.3	0.0150┬á┬▒┬á1.3┬á┬▒┬á0.2┬á┬▒┬á0.3	0.0149┬á┬▒┬á1.3┬á┬▒┬á0.2┬á┬▒┬á0.6	0.0149┬á┬▒┬á1.0┬á┬▒┬á0.2┬á┬▒┬á0.4
1.496ΓÇô1.947	0.00643┬á┬▒┬á2.1┬á┬▒┬á0.6┬á┬▒┬á1.0	0.00641┬á┬▒┬á2.1┬á┬▒┬á0.6┬á┬▒┬á1.0	0.00638┬á┬▒┬á1.8┬á┬▒┬á0.3┬á┬▒┬á0.4	0.00641┬á┬▒┬á1.8┬á┬▒┬á0.3┬á┬▒┬á0.4	0.00642┬á┬▒┬á1.8┬á┬▒┬á0.3┬á┬▒┬á0.7	0.00641┬á┬▒┬á1.4┬á┬▒┬á0.3┬á┬▒┬á0.5
1.947ΓÇô2.522	0.00274┬á┬▒┬á2.9┬á┬▒┬á0.7┬á┬▒┬á1.0	0.00272┬á┬▒┬á2.9┬á┬▒┬á0.7┬á┬▒┬á1.1	0.00299┬á┬▒┬á2.3┬á┬▒┬á0.4┬á┬▒┬á0.4	0.00299┬á┬▒┬á2.3┬á┬▒┬á0.4┬á┬▒┬á0.4	0.00297┬á┬▒┬á2.3┬á┬▒┬á0.4┬á┬▒┬á0.7	0.00287┬á┬▒┬á1.8┬á┬▒┬á0.4┬á┬▒┬á0.5
2.522ΓÇô3.277	0.00131┬á┬▒┬á3.6┬á┬▒┬á0.7┬á┬▒┬á1.2	0.00130┬á┬▒┬á3.6┬á┬▒┬á0.8┬á┬▒┬á1.2	0.00128┬á┬▒┬á3.0┬á┬▒┬á0.6┬á┬▒┬á0.4	0.00128┬á┬▒┬á3.0┬á┬▒┬á0.6┬á┬▒┬á0.4	0.00129┬á┬▒┬á3.0┬á┬▒┬á0.6┬á┬▒┬á0.7	0.00129┬á┬▒┬á2.3┬á┬▒┬á0.5┬á┬▒┬á0.5
3.277ΓÇô5.000	0.000510┬á┬▒┬á3.8┬á┬▒┬á1.1┬á┬▒┬á1.4	0.000509┬á┬▒┬á3.8┬á┬▒┬á1.1┬á┬▒┬á1.4	0.000519┬á┬▒┬á3.1┬á┬▒┬á0.6┬á┬▒┬á0.7	0.000524┬á┬▒┬á3.1┬á┬▒┬á0.6┬á┬▒┬á0.7	0.000525┬á┬▒┬á3.1┬á┬▒┬á0.6┬á┬▒┬á0.9	0.000517┬á┬▒┬á2.4┬á┬▒┬á0.5┬á┬▒┬á0.7
5.000ΓÇô10.000	0.000141┬á┬▒┬á4.2┬á┬▒┬á0.9┬á┬▒┬á1.3	0.000141┬á┬▒┬á4.2┬á┬▒┬á0.9┬á┬▒┬á1.3	0.000127┬á┬▒┬á3.8┬á┬▒┬á0.6┬á┬▒┬á0.5	0.000128┬á┬▒┬á3.8┬á┬▒┬á0.6┬á┬▒┬á0.5	0.000128┬á┬▒┬á3.8┬á┬▒┬á0.6┬á┬▒┬á0.7	0.000133┬á┬▒┬á2.8┬á┬▒┬á0.5┬á┬▒┬á0.6

**Table 13:** The values of $(1/\sigma )\,{\mathrm {d}}\sigma /{\mathrm {d}}\phi ^*_{\eta }$ in each bin of $\phi ^*_{\eta }$ for the electron and muon channels separately (for various particle-level definitions) and for the Born-level combination in the kinematic region $66\ ~{\mathrm {GeV}}\le m_{\ell \ell }< 116\ ~{\mathrm {GeV}},\ 2.0 \le |y_{\ell \ell }| < 2.4$. The associated statistical and systematic (both uncorrelated and correlated between bins of $\phi ^*_{\eta }$) are provided in percentage form

Bin	$(1/\sigma )\,d\sigma /d\phi ^*_{\eta }$ ┬▒ Statistical [%] ┬▒ Uncorrelated systematic [%] ┬▒ Correlated systematic [%]					
	Electron channel		Muon channel			Combination
	Dressed	Born	Bare	Dressed	Born	Born
0.000ΓÇô0.004	9.324┬á┬▒┬á1.0┬á┬▒┬á0.4┬á┬▒┬á0.3	9.417┬á┬▒┬á1.0┬á┬▒┬á0.4┬á┬▒┬á0.3	9.348┬á┬▒┬á0.8┬á┬▒┬á0.1┬á┬▒┬á0.1	9.347┬á┬▒┬á0.8┬á┬▒┬á0.1┬á┬▒┬á0.1	9.417┬á┬▒┬á0.8┬á┬▒┬á0.1┬á┬▒┬á0.4	9.417┬á┬▒┬á0.6┬á┬▒┬á0.2┬á┬▒┬á0.2
0.004ΓÇô0.008	9.101┬á┬▒┬á1.0┬á┬▒┬á0.4┬á┬▒┬á0.3	9.182┬á┬▒┬á1.0┬á┬▒┬á0.4┬á┬▒┬á0.3	9.294┬á┬▒┬á0.9┬á┬▒┬á0.2┬á┬▒┬á0.1	9.288┬á┬▒┬á0.9┬á┬▒┬á0.2┬á┬▒┬á0.1	9.372┬á┬▒┬á0.9┬á┬▒┬á0.2┬á┬▒┬á0.4	9.303┬á┬▒┬á0.7┬á┬▒┬á0.2┬á┬▒┬á0.2
0.008ΓÇô0.012	9.083┬á┬▒┬á1.0┬á┬▒┬á0.4┬á┬▒┬á0.3	9.179┬á┬▒┬á1.0┬á┬▒┬á0.4┬á┬▒┬á0.3	8.969┬á┬▒┬á0.9┬á┬▒┬á0.2┬á┬▒┬á0.1	8.959┬á┬▒┬á0.9┬á┬▒┬á0.2┬á┬▒┬á0.1	9.023┬á┬▒┬á0.9┬á┬▒┬á0.2┬á┬▒┬á0.4	9.084┬á┬▒┬á0.7┬á┬▒┬á0.2┬á┬▒┬á0.2
0.012ΓÇô0.016	8.825┬á┬▒┬á1.0┬á┬▒┬á0.4┬á┬▒┬á0.3	8.895┬á┬▒┬á1.0┬á┬▒┬á0.4┬á┬▒┬á0.3	9.015┬á┬▒┬á0.9┬á┬▒┬á0.2┬á┬▒┬á0.1	8.993┬á┬▒┬á0.9┬á┬▒┬á0.2┬á┬▒┬á0.1	9.050┬á┬▒┬á0.9┬á┬▒┬á0.2┬á┬▒┬á0.4	8.991┬á┬▒┬á0.7┬á┬▒┬á0.2┬á┬▒┬á0.2
0.016ΓÇô0.020	8.401┬á┬▒┬á1.0┬á┬▒┬á0.4┬á┬▒┬á0.3	8.462┬á┬▒┬á1.0┬á┬▒┬á0.4┬á┬▒┬á0.3	8.469┬á┬▒┬á0.9┬á┬▒┬á0.2┬á┬▒┬á0.1	8.456┬á┬▒┬á0.9┬á┬▒┬á0.2┬á┬▒┬á0.1	8.540┬á┬▒┬á0.9┬á┬▒┬á0.2┬á┬▒┬á0.4	8.511┬á┬▒┬á0.7┬á┬▒┬á0.2┬á┬▒┬á0.2
0.020ΓÇô0.024	8.047┬á┬▒┬á1.1┬á┬▒┬á0.5┬á┬▒┬á0.3	8.094┬á┬▒┬á1.1┬á┬▒┬á0.5┬á┬▒┬á0.3	8.242┬á┬▒┬á0.9┬á┬▒┬á0.2┬á┬▒┬á0.1	8.241┬á┬▒┬á0.9┬á┬▒┬á0.2┬á┬▒┬á0.1	8.305┬á┬▒┬á0.9┬á┬▒┬á0.2┬á┬▒┬á0.4	8.236┬á┬▒┬á0.7┬á┬▒┬á0.2┬á┬▒┬á0.2
0.024ΓÇô0.029	7.986┬á┬▒┬á1.0┬á┬▒┬á0.5┬á┬▒┬á0.3	8.065┬á┬▒┬á1.0┬á┬▒┬á0.5┬á┬▒┬á0.4	7.748┬á┬▒┬á0.8┬á┬▒┬á0.2┬á┬▒┬á0.0	7.743┬á┬▒┬á0.8┬á┬▒┬á0.2┬á┬▒┬á0.0	7.799┬á┬▒┬á0.8┬á┬▒┬á0.2┬á┬▒┬á0.4	7.891┬á┬▒┬á0.6┬á┬▒┬á0.2┬á┬▒┬á0.2
0.029ΓÇô0.034	7.168┬á┬▒┬á1.0┬á┬▒┬á0.5┬á┬▒┬á0.3	7.173┬á┬▒┬á1.0┬á┬▒┬á0.5┬á┬▒┬á0.3	7.276┬á┬▒┬á0.9┬á┬▒┬á0.1┬á┬▒┬á0.0	7.270┬á┬▒┬á0.9┬á┬▒┬á0.1┬á┬▒┬á0.0	7.310┬á┬▒┬á0.9┬á┬▒┬á0.1┬á┬▒┬á0.4	7.267┬á┬▒┬á0.7┬á┬▒┬á0.2┬á┬▒┬á0.2
0.034ΓÇô0.039	6.833┬á┬▒┬á1.0┬á┬▒┬á0.5┬á┬▒┬á0.3	6.857┬á┬▒┬á1.0┬á┬▒┬á0.5┬á┬▒┬á0.3	6.781┬á┬▒┬á0.9┬á┬▒┬á0.2┬á┬▒┬á0.1	6.783┬á┬▒┬á0.9┬á┬▒┬á0.2┬á┬▒┬á0.1	6.828┬á┬▒┬á0.9┬á┬▒┬á0.2┬á┬▒┬á0.4	6.843┬á┬▒┬á0.7┬á┬▒┬á0.2┬á┬▒┬á0.2
0.039ΓÇô0.045	6.468┬á┬▒┬á1.0┬á┬▒┬á0.4┬á┬▒┬á0.3	6.490┬á┬▒┬á1.0┬á┬▒┬á0.4┬á┬▒┬á0.3	6.415┬á┬▒┬á0.8┬á┬▒┬á0.1┬á┬▒┬á0.0	6.408┬á┬▒┬á0.8┬á┬▒┬á0.1┬á┬▒┬á0.0	6.441┬á┬▒┬á0.8┬á┬▒┬á0.1┬á┬▒┬á0.4	6.457┬á┬▒┬á0.6┬á┬▒┬á0.2┬á┬▒┬á0.2
0.045ΓÇô0.051	5.717┬á┬▒┬á1.0┬á┬▒┬á0.4┬á┬▒┬á0.2	5.715┬á┬▒┬á1.0┬á┬▒┬á0.5┬á┬▒┬á0.3	5.865┬á┬▒┬á0.9┬á┬▒┬á0.2┬á┬▒┬á0.0	5.870┬á┬▒┬á0.9┬á┬▒┬á0.2┬á┬▒┬á0.0	5.854┬á┬▒┬á0.9┬á┬▒┬á0.2┬á┬▒┬á0.4	5.807┬á┬▒┬á0.7┬á┬▒┬á0.2┬á┬▒┬á0.2
0.051ΓÇô0.057	5.413┬á┬▒┬á1.1┬á┬▒┬á0.4┬á┬▒┬á0.2	5.410┬á┬▒┬á1.1┬á┬▒┬á0.4┬á┬▒┬á0.2	5.333┬á┬▒┬á0.9┬á┬▒┬á0.2┬á┬▒┬á0.1	5.330┬á┬▒┬á0.9┬á┬▒┬á0.2┬á┬▒┬á0.1	5.336┬á┬▒┬á0.9┬á┬▒┬á0.2┬á┬▒┬á0.4	5.366┬á┬▒┬á0.7┬á┬▒┬á0.2┬á┬▒┬á0.2
0.057ΓÇô0.064	4.935┬á┬▒┬á1.0┬á┬▒┬á0.4┬á┬▒┬á0.2	4.934┬á┬▒┬á1.0┬á┬▒┬á0.4┬á┬▒┬á0.2	4.923┬á┬▒┬á0.9┬á┬▒┬á0.2┬á┬▒┬á0.0	4.915┬á┬▒┬á0.9┬á┬▒┬á0.2┬á┬▒┬á0.0	4.906┬á┬▒┬á0.9┬á┬▒┬á0.2┬á┬▒┬á0.2	4.918┬á┬▒┬á0.7┬á┬▒┬á0.2┬á┬▒┬á0.1
0.064ΓÇô0.072	4.502┬á┬▒┬á1.0┬á┬▒┬á0.4┬á┬▒┬á0.2	4.491┬á┬▒┬á1.0┬á┬▒┬á0.4┬á┬▒┬á0.2	4.434┬á┬▒┬á0.9┬á┬▒┬á0.2┬á┬▒┬á0.1	4.435┬á┬▒┬á0.9┬á┬▒┬á0.2┬á┬▒┬á0.1	4.427┬á┬▒┬á0.9┬á┬▒┬á0.2┬á┬▒┬á0.2	4.452┬á┬▒┬á0.7┬á┬▒┬á0.2┬á┬▒┬á0.1
0.072ΓÇô0.081	3.994┬á┬▒┬á1.0┬á┬▒┬á0.5┬á┬▒┬á0.2	3.977┬á┬▒┬á1.0┬á┬▒┬á0.5┬á┬▒┬á0.2	3.978┬á┬▒┬á0.9┬á┬▒┬á0.1┬á┬▒┬á0.1	3.982┬á┬▒┬á0.9┬á┬▒┬á0.1┬á┬▒┬á0.1	3.968┬á┬▒┬á0.9┬á┬▒┬á0.1┬á┬▒┬á0.2	3.972┬á┬▒┬á0.7┬á┬▒┬á0.2┬á┬▒┬á0.2
0.081ΓÇô0.091	3.517┬á┬▒┬á1.0┬á┬▒┬á0.5┬á┬▒┬á0.1	3.496┬á┬▒┬á1.0┬á┬▒┬á0.5┬á┬▒┬á0.2	3.572┬á┬▒┬á0.9┬á┬▒┬á0.1┬á┬▒┬á0.1	3.569┬á┬▒┬á0.9┬á┬▒┬á0.1┬á┬▒┬á0.1	3.563┬á┬▒┬á0.9┬á┬▒┬á0.1┬á┬▒┬á0.2	3.539┬á┬▒┬á0.7┬á┬▒┬á0.2┬á┬▒┬á0.1
0.091ΓÇô0.102	3.161┬á┬▒┬á1.0┬á┬▒┬á0.6┬á┬▒┬á0.1	3.148┬á┬▒┬á1.0┬á┬▒┬á0.6┬á┬▒┬á0.2	3.146┬á┬▒┬á0.8┬á┬▒┬á0.2┬á┬▒┬á0.0	3.144┬á┬▒┬á0.8┬á┬▒┬á0.2┬á┬▒┬á0.0	3.122┬á┬▒┬á0.8┬á┬▒┬á0.2┬á┬▒┬á0.2	3.132┬á┬▒┬á0.7┬á┬▒┬á0.2┬á┬▒┬á0.1
0.102ΓÇô0.114	2.793┬á┬▒┬á1.1┬á┬▒┬á0.6┬á┬▒┬á0.2	2.784┬á┬▒┬á1.1┬á┬▒┬á0.6┬á┬▒┬á0.2	2.775┬á┬▒┬á0.9┬á┬▒┬á0.1┬á┬▒┬á0.1	2.779┬á┬▒┬á0.9┬á┬▒┬á0.1┬á┬▒┬á0.1	2.762┬á┬▒┬á0.9┬á┬▒┬á0.1┬á┬▒┬á0.2	2.770┬á┬▒┬á0.7┬á┬▒┬á0.2┬á┬▒┬á0.1
0.114ΓÇô0.128	2.404┬á┬▒┬á1.1┬á┬▒┬á0.6┬á┬▒┬á0.1	2.385┬á┬▒┬á1.1┬á┬▒┬á0.6┬á┬▒┬á0.1	2.441┬á┬▒┬á0.9┬á┬▒┬á0.1┬á┬▒┬á0.1	2.438┬á┬▒┬á0.9┬á┬▒┬á0.1┬á┬▒┬á0.1	2.420┬á┬▒┬á0.9┬á┬▒┬á0.1┬á┬▒┬á0.3	2.408┬á┬▒┬á0.7┬á┬▒┬á0.2┬á┬▒┬á0.2
0.128ΓÇô0.145	2.036┬á┬▒┬á1.0┬á┬▒┬á0.6┬á┬▒┬á0.1	2.021┬á┬▒┬á1.0┬á┬▒┬á0.6┬á┬▒┬á0.1	2.074┬á┬▒┬á0.8┬á┬▒┬á0.2┬á┬▒┬á0.1	2.074┬á┬▒┬á0.8┬á┬▒┬á0.2┬á┬▒┬á0.1	2.053┬á┬▒┬á0.8┬á┬▒┬á0.2┬á┬▒┬á0.3	2.043┬á┬▒┬á0.7┬á┬▒┬á0.2┬á┬▒┬á0.2
0.145ΓÇô0.165	1.743┬á┬▒┬á1.0┬á┬▒┬á0.6┬á┬▒┬á0.2	1.732┬á┬▒┬á1.0┬á┬▒┬á0.6┬á┬▒┬á0.2	1.736┬á┬▒┬á0.9┬á┬▒┬á0.1┬á┬▒┬á0.1	1.737┬á┬▒┬á0.9┬á┬▒┬á0.1┬á┬▒┬á0.1	1.730┬á┬▒┬á0.9┬á┬▒┬á0.1┬á┬▒┬á0.3	1.730┬á┬▒┬á0.7┬á┬▒┬á0.2┬á┬▒┬á0.2
0.165ΓÇô0.189	1.447┬á┬▒┬á1.0┬á┬▒┬á0.6┬á┬▒┬á0.2	1.439┬á┬▒┬á1.0┬á┬▒┬á0.6┬á┬▒┬á0.2	1.453┬á┬▒┬á0.9┬á┬▒┬á0.1┬á┬▒┬á0.1	1.455┬á┬▒┬á0.9┬á┬▒┬á0.1┬á┬▒┬á0.1	1.449┬á┬▒┬á0.9┬á┬▒┬á0.1┬á┬▒┬á0.3	1.445┬á┬▒┬á0.7┬á┬▒┬á0.2┬á┬▒┬á0.2
0.189ΓÇô0.219	1.175┬á┬▒┬á1.0┬á┬▒┬á0.4┬á┬▒┬á0.2	1.167┬á┬▒┬á1.0┬á┬▒┬á0.4┬á┬▒┬á0.2	1.147┬á┬▒┬á0.9┬á┬▒┬á0.1┬á┬▒┬á0.1	1.147┬á┬▒┬á0.9┬á┬▒┬á0.1┬á┬▒┬á0.1	1.139┬á┬▒┬á0.9┬á┬▒┬á0.1┬á┬▒┬á0.3	1.149┬á┬▒┬á0.7┬á┬▒┬á0.2┬á┬▒┬á0.2
0.219ΓÇô0.258	0.894┬á┬▒┬á1.0┬á┬▒┬á0.5┬á┬▒┬á0.3	0.889┬á┬▒┬á1.0┬á┬▒┬á0.5┬á┬▒┬á0.3	0.888┬á┬▒┬á0.8┬á┬▒┬á0.2┬á┬▒┬á0.1	0.889┬á┬▒┬á0.8┬á┬▒┬á0.2┬á┬▒┬á0.1	0.883┬á┬▒┬á0.8┬á┬▒┬á0.2┬á┬▒┬á0.3	0.885┬á┬▒┬á0.7┬á┬▒┬á0.2┬á┬▒┬á0.2
0.258ΓÇô0.312	0.656┬á┬▒┬á1.0┬á┬▒┬á0.6┬á┬▒┬á0.4	0.653┬á┬▒┬á1.0┬á┬▒┬á0.6┬á┬▒┬á0.4	0.646┬á┬▒┬á0.9┬á┬▒┬á0.1┬á┬▒┬á0.2	0.646┬á┬▒┬á0.9┬á┬▒┬á0.1┬á┬▒┬á0.2	0.643┬á┬▒┬á0.9┬á┬▒┬á0.1┬á┬▒┬á0.4	0.646┬á┬▒┬á0.7┬á┬▒┬á0.2┬á┬▒┬á0.3
0.312ΓÇô0.391	0.438┬á┬▒┬á1.1┬á┬▒┬á0.6┬á┬▒┬á0.5	0.436┬á┬▒┬á1.1┬á┬▒┬á0.6┬á┬▒┬á0.5	0.436┬á┬▒┬á0.9┬á┬▒┬á0.1┬á┬▒┬á0.1	0.437┬á┬▒┬á0.9┬á┬▒┬á0.1┬á┬▒┬á0.1	0.435┬á┬▒┬á0.9┬á┬▒┬á0.1┬á┬▒┬á0.4	0.436┬á┬▒┬á0.7┬á┬▒┬á0.2┬á┬▒┬á0.3
0.391ΓÇô0.524	0.255┬á┬▒┬á1.1┬á┬▒┬á0.6┬á┬▒┬á0.6	0.255┬á┬▒┬á1.1┬á┬▒┬á0.6┬á┬▒┬á0.6	0.253┬á┬▒┬á0.9┬á┬▒┬á0.1┬á┬▒┬á0.2	0.253┬á┬▒┬á0.9┬á┬▒┬á0.1┬á┬▒┬á0.2	0.253┬á┬▒┬á0.9┬á┬▒┬á0.1┬á┬▒┬á0.4	0.254┬á┬▒┬á0.7┬á┬▒┬á0.2┬á┬▒┬á0.3
0.524ΓÇô0.695	0.131┬á┬▒┬á1.3┬á┬▒┬á0.4┬á┬▒┬á0.7	0.130┬á┬▒┬á1.3┬á┬▒┬á0.4┬á┬▒┬á0.7	0.134┬á┬▒┬á1.1┬á┬▒┬á0.2┬á┬▒┬á0.2	0.134┬á┬▒┬á1.1┬á┬▒┬á0.2┬á┬▒┬á0.2	0.134┬á┬▒┬á1.1┬á┬▒┬á0.2┬á┬▒┬á0.4	0.132┬á┬▒┬á0.8┬á┬▒┬á0.2┬á┬▒┬á0.3
0.695ΓÇô0.918	0.0612┬á┬▒┬á1.7┬á┬▒┬á0.5┬á┬▒┬á0.9	0.0611┬á┬▒┬á1.7┬á┬▒┬á0.5┬á┬▒┬á0.9	0.0608┬á┬▒┬á1.4┬á┬▒┬á0.2┬á┬▒┬á0.3	0.0609┬á┬▒┬á1.4┬á┬▒┬á0.2┬á┬▒┬á0.3	0.0607┬á┬▒┬á1.4┬á┬▒┬á0.2┬á┬▒┬á0.4	0.0608┬á┬▒┬á1.1┬á┬▒┬á0.3┬á┬▒┬á0.4
0.918ΓÇô1.153	0.0292┬á┬▒┬á2.3┬á┬▒┬á0.5┬á┬▒┬á1.1	0.0291┬á┬▒┬á2.3┬á┬▒┬á0.5┬á┬▒┬á1.1	0.0288┬á┬▒┬á2.0┬á┬▒┬á0.3┬á┬▒┬á0.3	0.0290┬á┬▒┬á2.0┬á┬▒┬á0.3┬á┬▒┬á0.3	0.0291┬á┬▒┬á2.0┬á┬▒┬á0.3┬á┬▒┬á0.4	0.0291┬á┬▒┬á1.5┬á┬▒┬á0.3┬á┬▒┬á0.4
1.153ΓÇô1.496	0.0132┬á┬▒┬á2.8┬á┬▒┬á0.6┬á┬▒┬á1.3	0.0132┬á┬▒┬á2.8┬á┬▒┬á0.6┬á┬▒┬á1.3	0.0129┬á┬▒┬á2.4┬á┬▒┬á0.5┬á┬▒┬á0.3	0.0129┬á┬▒┬á2.4┬á┬▒┬á0.5┬á┬▒┬á0.3	0.0128┬á┬▒┬á2.4┬á┬▒┬á0.5┬á┬▒┬á1.0	0.0130┬á┬▒┬á1.8┬á┬▒┬á0.4┬á┬▒┬á0.6
1.496ΓÇô1.947	0.00492┬á┬▒┬á4.0┬á┬▒┬á0.8┬á┬▒┬á1.6	0.00493┬á┬▒┬á4.0┬á┬▒┬á0.8┬á┬▒┬á1.6	0.00483┬á┬▒┬á3.5┬á┬▒┬á0.5┬á┬▒┬á0.4	0.00486┬á┬▒┬á3.5┬á┬▒┬á0.5┬á┬▒┬á0.4	0.00483┬á┬▒┬á3.5┬á┬▒┬á0.5┬á┬▒┬á1.0	0.00486┬á┬▒┬á2.6┬á┬▒┬á0.4┬á┬▒┬á0.7
1.947ΓÇô2.522	0.00191┬á┬▒┬á5.7┬á┬▒┬á1.1┬á┬▒┬á2.0	0.00189┬á┬▒┬á5.7┬á┬▒┬á1.1┬á┬▒┬á1.9	0.00153┬á┬▒┬á5.4┬á┬▒┬á0.9┬á┬▒┬á0.7	0.00154┬á┬▒┬á5.4┬á┬▒┬á0.9┬á┬▒┬á0.7	0.00153┬á┬▒┬á5.4┬á┬▒┬á0.9┬á┬▒┬á1.2	0.00168┬á┬▒┬á3.9┬á┬▒┬á0.7┬á┬▒┬á0.9
2.522ΓÇô3.277	0.000583┬á┬▒┬á8.9┬á┬▒┬á1.7┬á┬▒┬á2.3	0.000582┬á┬▒┬á8.9┬á┬▒┬á1.8┬á┬▒┬á2.3	0.000535┬á┬▒┬á8.3┬á┬▒┬á1.1┬á┬▒┬á0.7	0.000536┬á┬▒┬á8.3┬á┬▒┬á1.1┬á┬▒┬á0.7	0.000535┬á┬▒┬á8.3┬á┬▒┬á1.1┬á┬▒┬á1.2	0.000553┬á┬▒┬á6.1┬á┬▒┬á1.0┬á┬▒┬á1.0
3.277ΓÇô5.000	0.000139┬á┬▒┬á12┬á┬▒┬á2.4┬á┬▒┬á3.1	0.000138┬á┬▒┬á12┬á┬▒┬á2.5┬á┬▒┬á3.1	0.000182┬á┬▒┬á9.3┬á┬▒┬á1.3┬á┬▒┬á1.4	0.000183┬á┬▒┬á9.3┬á┬▒┬á1.3┬á┬▒┬á1.4	0.000183┬á┬▒┬á9.3┬á┬▒┬á1.3┬á┬▒┬á1.7	0.000163┬á┬▒┬á7.4┬á┬▒┬á1.3┬á┬▒┬á1.4
5.000ΓÇô10.000	0.0000247┬á┬▒┬á18┬á┬▒┬á5.4┬á┬▒┬á8.4	0.0000248┬á┬▒┬á18┬á┬▒┬á5.4┬á┬▒┬á8.5	0.0000308┬á┬▒┬á13┬á┬▒┬á2.4┬á┬▒┬á1.8	0.0000311┬á┬▒┬á13┬á┬▒┬á2.4┬á┬▒┬á1.8	0.0000314┬á┬▒┬á13┬á┬▒┬á2.4┬á┬▒┬á2.0	0.0000285┬á┬▒┬á11┬á┬▒┬á2.6┬á┬▒┬á3.2

**Table 14:** The values of $(1/\sigma )\,{\mathrm {d}}\sigma /{\mathrm {d}}\phi ^*_{\eta }$ in each bin of $\phi ^*_{\eta }$ for the electron and muon channels separately (for various particle-level definitions) and for the Born-level combination in the kinematic region $116\ ~{\mathrm {GeV}}\le m_{\ell \ell }< 150\ ~{\mathrm {GeV}},\ 0 \le |y_{\ell \ell }| < 0.8$. The associated statistical and systematic (both uncorrelated and correlated between bins of $\phi ^*_{\eta }$) are provided in percentage form

Bin	$(1/\sigma )\,d\sigma /d\phi ^*_{\eta }$ ┬▒ Statistical [%] ┬▒ Uncorrelated systematic [%] ┬▒ Correlated systematic [%]					
	Electron channel		Muon channel			Combination
	Dressed	Born	Bare	Dressed	Born	Born
0.000ΓÇô0.004	11.489┬á┬▒┬á2.7┬á┬▒┬á0.9┬á┬▒┬á2.9	11.576┬á┬▒┬á2.7┬á┬▒┬á0.9┬á┬▒┬á2.9	11.151┬á┬▒┬á1.8┬á┬▒┬á0.7┬á┬▒┬á3.4	11.135┬á┬▒┬á1.8┬á┬▒┬á0.7┬á┬▒┬á3.4	11.235┬á┬▒┬á1.8┬á┬▒┬á0.7┬á┬▒┬á3.5	11.412┬á┬▒┬á1.5┬á┬▒┬á0.6┬á┬▒┬á3.2
0.004ΓÇô0.008	11.375┬á┬▒┬á2.7┬á┬▒┬á0.7┬á┬▒┬á1.3	11.449┬á┬▒┬á2.7┬á┬▒┬á0.8┬á┬▒┬á1.3	11.398┬á┬▒┬á1.7┬á┬▒┬á0.8┬á┬▒┬á1.5	11.361┬á┬▒┬á1.7┬á┬▒┬á0.8┬á┬▒┬á1.5	11.340┬á┬▒┬á1.7┬á┬▒┬á0.8┬á┬▒┬á1.6	11.355┬á┬▒┬á1.5┬á┬▒┬á0.6┬á┬▒┬á1.4
0.008ΓÇô0.012	11.051┬á┬▒┬á2.7┬á┬▒┬á0.8┬á┬▒┬á0.9	11.091┬á┬▒┬á2.7┬á┬▒┬á0.8┬á┬▒┬á0.9	11.056┬á┬▒┬á1.8┬á┬▒┬á0.8┬á┬▒┬á1.2	10.980┬á┬▒┬á1.8┬á┬▒┬á0.8┬á┬▒┬á1.2	11.131┬á┬▒┬á1.8┬á┬▒┬á0.8┬á┬▒┬á1.4	11.077┬á┬▒┬á1.5┬á┬▒┬á0.6┬á┬▒┬á1.0
0.012ΓÇô0.016	10.804┬á┬▒┬á2.7┬á┬▒┬á0.7┬á┬▒┬á1.2	10.904┬á┬▒┬á2.7┬á┬▒┬á0.8┬á┬▒┬á1.2	11.077┬á┬▒┬á1.8┬á┬▒┬á0.8┬á┬▒┬á0.9	11.006┬á┬▒┬á1.8┬á┬▒┬á0.8┬á┬▒┬á0.9	10.970┬á┬▒┬á1.8┬á┬▒┬á0.8┬á┬▒┬á1.2	10.920┬á┬▒┬á1.5┬á┬▒┬á0.6┬á┬▒┬á0.9
0.016ΓÇô0.020	10.038┬á┬▒┬á2.8┬á┬▒┬á0.7┬á┬▒┬á0.8	10.105┬á┬▒┬á2.8┬á┬▒┬á0.8┬á┬▒┬á0.7	10.116┬á┬▒┬á1.9┬á┬▒┬á0.9┬á┬▒┬á0.9	10.039┬á┬▒┬á1.9┬á┬▒┬á0.9┬á┬▒┬á0.9	10.107┬á┬▒┬á1.9┬á┬▒┬á0.9┬á┬▒┬á1.2	10.068┬á┬▒┬á1.6┬á┬▒┬á0.6┬á┬▒┬á0.8
0.020ΓÇô0.024	9.378┬á┬▒┬á2.9┬á┬▒┬á0.8┬á┬▒┬á0.7	9.372┬á┬▒┬á2.9┬á┬▒┬á0.8┬á┬▒┬á0.8	9.319┬á┬▒┬á1.9┬á┬▒┬á0.8┬á┬▒┬á1.0	9.293┬á┬▒┬á1.9┬á┬▒┬á0.8┬á┬▒┬á1.0	9.330┬á┬▒┬á1.9┬á┬▒┬á0.8┬á┬▒┬á1.2	9.307┬á┬▒┬á1.6┬á┬▒┬á0.6┬á┬▒┬á0.8
0.024ΓÇô0.029	9.075┬á┬▒┬á2.7┬á┬▒┬á0.7┬á┬▒┬á0.6	9.117┬á┬▒┬á2.7┬á┬▒┬á0.8┬á┬▒┬á0.6	8.794┬á┬▒┬á1.8┬á┬▒┬á0.8┬á┬▒┬á0.5	8.773┬á┬▒┬á1.8┬á┬▒┬á0.8┬á┬▒┬á0.5	8.847┬á┬▒┬á1.8┬á┬▒┬á0.8┬á┬▒┬á0.7	8.907┬á┬▒┬á1.5┬á┬▒┬á0.6┬á┬▒┬á0.6
0.029ΓÇô0.034	8.348┬á┬▒┬á2.7┬á┬▒┬á0.7┬á┬▒┬á0.6	8.376┬á┬▒┬á2.7┬á┬▒┬á0.8┬á┬▒┬á0.6	8.466┬á┬▒┬á1.8┬á┬▒┬á0.8┬á┬▒┬á0.9	8.532┬á┬▒┬á1.8┬á┬▒┬á0.8┬á┬▒┬á0.9	8.557┬á┬▒┬á1.8┬á┬▒┬á0.8┬á┬▒┬á1.0	8.437┬á┬▒┬á1.5┬á┬▒┬á0.6┬á┬▒┬á0.7
0.034ΓÇô0.039	6.798┬á┬▒┬á3.1┬á┬▒┬á0.9┬á┬▒┬á0.7	6.776┬á┬▒┬á3.1┬á┬▒┬á0.9┬á┬▒┬á0.7	7.793┬á┬▒┬á1.9┬á┬▒┬á0.8┬á┬▒┬á0.9	7.781┬á┬▒┬á1.9┬á┬▒┬á0.8┬á┬▒┬á0.9	7.815┬á┬▒┬á1.9┬á┬▒┬á0.8┬á┬▒┬á1.0	7.449┬á┬▒┬á1.6┬á┬▒┬á0.6┬á┬▒┬á0.7
0.039ΓÇô0.045	6.684┬á┬▒┬á2.8┬á┬▒┬á0.9┬á┬▒┬á0.7	6.689┬á┬▒┬á2.8┬á┬▒┬á0.9┬á┬▒┬á0.7	6.810┬á┬▒┬á1.9┬á┬▒┬á0.8┬á┬▒┬á1.1	6.826┬á┬▒┬á1.9┬á┬▒┬á0.8┬á┬▒┬á1.1	6.806┬á┬▒┬á1.9┬á┬▒┬á0.8┬á┬▒┬á1.1	6.711┬á┬▒┬á1.6┬á┬▒┬á0.6┬á┬▒┬á0.8
0.045ΓÇô0.051	6.001┬á┬▒┬á2.9┬á┬▒┬á0.8┬á┬▒┬á0.7	5.999┬á┬▒┬á2.9┬á┬▒┬á0.9┬á┬▒┬á0.7	5.993┬á┬▒┬á2.0┬á┬▒┬á0.8┬á┬▒┬á1.0	5.965┬á┬▒┬á2.0┬á┬▒┬á0.8┬á┬▒┬á1.0	6.017┬á┬▒┬á2.0┬á┬▒┬á0.8┬á┬▒┬á1.1	5.961┬á┬▒┬á1.6┬á┬▒┬á0.6┬á┬▒┬á0.7
0.051ΓÇô0.057	5.433┬á┬▒┬á3.1┬á┬▒┬á0.9┬á┬▒┬á0.9	5.384┬á┬▒┬á3.1┬á┬▒┬á0.9┬á┬▒┬á1.0	5.455┬á┬▒┬á2.1┬á┬▒┬á0.9┬á┬▒┬á1.1	5.439┬á┬▒┬á2.1┬á┬▒┬á0.9┬á┬▒┬á1.1	5.355┬á┬▒┬á2.1┬á┬▒┬á0.9┬á┬▒┬á1.2	5.316┬á┬▒┬á1.7┬á┬▒┬á0.7┬á┬▒┬á0.8
0.057ΓÇô0.064	5.175┬á┬▒┬á2.9┬á┬▒┬á0.9┬á┬▒┬á0.7	5.210┬á┬▒┬á2.9┬á┬▒┬á0.9┬á┬▒┬á0.7	4.958┬á┬▒┬á2.0┬á┬▒┬á0.8┬á┬▒┬á0.8	4.949┬á┬▒┬á2.0┬á┬▒┬á0.8┬á┬▒┬á0.8	4.933┬á┬▒┬á2.0┬á┬▒┬á0.8┬á┬▒┬á0.8	4.999┬á┬▒┬á1.7┬á┬▒┬á0.6┬á┬▒┬á0.7
0.064ΓÇô0.072	4.099┬á┬▒┬á3.1┬á┬▒┬á0.9┬á┬▒┬á0.7	4.049┬á┬▒┬á3.1┬á┬▒┬á0.9┬á┬▒┬á0.7	4.509┬á┬▒┬á2.0┬á┬▒┬á0.9┬á┬▒┬á0.8	4.528┬á┬▒┬á2.0┬á┬▒┬á0.9┬á┬▒┬á0.8	4.531┬á┬▒┬á2.0┬á┬▒┬á0.9┬á┬▒┬á0.9	4.356┬á┬▒┬á1.7┬á┬▒┬á0.7┬á┬▒┬á0.7
0.072ΓÇô0.081	4.003┬á┬▒┬á3.0┬á┬▒┬á0.8┬á┬▒┬á0.7	4.009┬á┬▒┬á3.0┬á┬▒┬á0.8┬á┬▒┬á0.7	3.750┬á┬▒┬á2.0┬á┬▒┬á0.8┬á┬▒┬á0.9	3.744┬á┬▒┬á2.0┬á┬▒┬á0.8┬á┬▒┬á0.9	3.731┬á┬▒┬á2.0┬á┬▒┬á0.8┬á┬▒┬á0.9	3.802┬á┬▒┬á1.7┬á┬▒┬á0.6┬á┬▒┬á0.7
0.081ΓÇô0.091	3.423┬á┬▒┬á3.1┬á┬▒┬á0.9┬á┬▒┬á0.9	3.437┬á┬▒┬á3.1┬á┬▒┬á1.0┬á┬▒┬á0.9	3.303┬á┬▒┬á2.1┬á┬▒┬á0.9┬á┬▒┬á0.6	3.302┬á┬▒┬á2.1┬á┬▒┬á0.9┬á┬▒┬á0.6	3.303┬á┬▒┬á2.1┬á┬▒┬á0.9┬á┬▒┬á0.7	3.339┬á┬▒┬á1.7┬á┬▒┬á0.7┬á┬▒┬á0.7
0.091ΓÇô0.102	2.992┬á┬▒┬á3.1┬á┬▒┬á0.9┬á┬▒┬á0.9	2.983┬á┬▒┬á3.1┬á┬▒┬á1.0┬á┬▒┬á0.9	2.946┬á┬▒┬á2.1┬á┬▒┬á1.0┬á┬▒┬á0.7	2.958┬á┬▒┬á2.1┬á┬▒┬á1.0┬á┬▒┬á0.7	2.916┬á┬▒┬á2.1┬á┬▒┬á1.0┬á┬▒┬á0.8	2.934┬á┬▒┬á1.7┬á┬▒┬á0.7┬á┬▒┬á0.7
0.102ΓÇô0.114	2.495┬á┬▒┬á3.3┬á┬▒┬á1.9┬á┬▒┬á2.8	2.489┬á┬▒┬á3.3┬á┬▒┬á1.9┬á┬▒┬á2.7	2.694┬á┬▒┬á2.1┬á┬▒┬á0.9┬á┬▒┬á0.7	2.697┬á┬▒┬á2.1┬á┬▒┬á0.9┬á┬▒┬á0.7	2.710┬á┬▒┬á2.1┬á┬▒┬á0.9┬á┬▒┬á0.8	2.644┬á┬▒┬á1.8┬á┬▒┬á0.8┬á┬▒┬á0.9
0.114ΓÇô0.128	2.134┬á┬▒┬á3.3┬á┬▒┬á0.9┬á┬▒┬á0.7	2.125┬á┬▒┬á3.3┬á┬▒┬á0.9┬á┬▒┬á0.7	2.240┬á┬▒┬á2.1┬á┬▒┬á0.9┬á┬▒┬á0.9	2.246┬á┬▒┬á2.1┬á┬▒┬á0.9┬á┬▒┬á0.9	2.227┬á┬▒┬á2.1┬á┬▒┬á0.9┬á┬▒┬á0.9	2.195┬á┬▒┬á1.8┬á┬▒┬á0.7┬á┬▒┬á0.7
0.128ΓÇô0.145	1.857┬á┬▒┬á3.3┬á┬▒┬á1.1┬á┬▒┬á1.1	1.843┬á┬▒┬á3.3┬á┬▒┬á1.1┬á┬▒┬á1.2	1.771┬á┬▒┬á2.2┬á┬▒┬á0.9┬á┬▒┬á0.8	1.785┬á┬▒┬á2.2┬á┬▒┬á0.9┬á┬▒┬á0.8	1.786┬á┬▒┬á2.2┬á┬▒┬á0.9┬á┬▒┬á0.9	1.796┬á┬▒┬á1.8┬á┬▒┬á0.7┬á┬▒┬á0.8
0.145ΓÇô0.165	1.507┬á┬▒┬á3.3┬á┬▒┬á0.9┬á┬▒┬á0.8	1.505┬á┬▒┬á3.3┬á┬▒┬á1.0┬á┬▒┬á0.9	1.579┬á┬▒┬á2.2┬á┬▒┬á1.0┬á┬▒┬á0.8	1.582┬á┬▒┬á2.2┬á┬▒┬á1.0┬á┬▒┬á0.8	1.581┬á┬▒┬á2.2┬á┬▒┬á1.0┬á┬▒┬á0.8	1.553┬á┬▒┬á1.8┬á┬▒┬á0.7┬á┬▒┬á0.7
0.165ΓÇô0.189	1.212┬á┬▒┬á3.5┬á┬▒┬á1.5┬á┬▒┬á1.5	1.200┬á┬▒┬á3.5┬á┬▒┬á1.5┬á┬▒┬á1.5	1.203┬á┬▒┬á2.3┬á┬▒┬á0.9┬á┬▒┬á0.8	1.211┬á┬▒┬á2.3┬á┬▒┬á0.9┬á┬▒┬á0.8	1.193┬á┬▒┬á2.3┬á┬▒┬á0.9┬á┬▒┬á0.8	1.199┬á┬▒┬á1.9┬á┬▒┬á0.8┬á┬▒┬á0.8
0.189ΓÇô0.219	0.979┬á┬▒┬á3.4┬á┬▒┬á1.2┬á┬▒┬á1.2	0.978┬á┬▒┬á3.4┬á┬▒┬á1.2┬á┬▒┬á1.1	0.933┬á┬▒┬á2.3┬á┬▒┬á0.9┬á┬▒┬á1.9	0.933┬á┬▒┬á2.3┬á┬▒┬á0.9┬á┬▒┬á1.9	0.939┬á┬▒┬á2.3┬á┬▒┬á0.9┬á┬▒┬á1.9	0.964┬á┬▒┬á1.9┬á┬▒┬á0.7┬á┬▒┬á1.1
0.219ΓÇô0.258	0.705┬á┬▒┬á3.6┬á┬▒┬á1.4┬á┬▒┬á1.2	0.701┬á┬▒┬á3.6┬á┬▒┬á1.4┬á┬▒┬á1.2	0.741┬á┬▒┬á2.2┬á┬▒┬á1.0┬á┬▒┬á2.1	0.740┬á┬▒┬á2.2┬á┬▒┬á1.0┬á┬▒┬á2.1	0.736┬á┬▒┬á2.2┬á┬▒┬á1.0┬á┬▒┬á2.1	0.736┬á┬▒┬á1.9┬á┬▒┬á0.8┬á┬▒┬á1.2
0.258ΓÇô0.312	0.526┬á┬▒┬á3.7┬á┬▒┬á2.2┬á┬▒┬á2.0	0.524┬á┬▒┬á3.7┬á┬▒┬á2.2┬á┬▒┬á2.0	0.524┬á┬▒┬á2.3┬á┬▒┬á1.1┬á┬▒┬á1.6	0.524┬á┬▒┬á2.3┬á┬▒┬á1.1┬á┬▒┬á1.6	0.522┬á┬▒┬á2.3┬á┬▒┬á1.1┬á┬▒┬á1.8	0.531┬á┬▒┬á2.0┬á┬▒┬á1.0┬á┬▒┬á1.3
0.312ΓÇô0.391	0.354┬á┬▒┬á3.8┬á┬▒┬á1.7┬á┬▒┬á2.3	0.354┬á┬▒┬á3.8┬á┬▒┬á1.7┬á┬▒┬á2.3	0.340┬á┬▒┬á2.4┬á┬▒┬á1.2┬á┬▒┬á2.2	0.341┬á┬▒┬á2.4┬á┬▒┬á1.2┬á┬▒┬á2.2	0.340┬á┬▒┬á2.4┬á┬▒┬á1.2┬á┬▒┬á2.4	0.351┬á┬▒┬á2.0┬á┬▒┬á1.0┬á┬▒┬á1.7
0.391ΓÇô0.524	0.199┬á┬▒┬á4.1┬á┬▒┬á2.0┬á┬▒┬á3.3	0.199┬á┬▒┬á4.1┬á┬▒┬á2.0┬á┬▒┬á3.3	0.196┬á┬▒┬á2.6┬á┬▒┬á1.4┬á┬▒┬á3.0	0.197┬á┬▒┬á2.6┬á┬▒┬á1.4┬á┬▒┬á3.0	0.196┬á┬▒┬á2.6┬á┬▒┬á1.4┬á┬▒┬á3.1	0.201┬á┬▒┬á2.1┬á┬▒┬á1.2┬á┬▒┬á2.3
0.524ΓÇô0.695	0.103┬á┬▒┬á5.3┬á┬▒┬á2.8┬á┬▒┬á3.8	0.103┬á┬▒┬á5.3┬á┬▒┬á2.8┬á┬▒┬á3.8	0.0918┬á┬▒┬á3.3┬á┬▒┬á2.7┬á┬▒┬á4.1	0.0920┬á┬▒┬á3.3┬á┬▒┬á2.7┬á┬▒┬á4.1	0.0910┬á┬▒┬á3.3┬á┬▒┬á2.7┬á┬▒┬á4.1	0.0994┬á┬▒┬á2.8┬á┬▒┬á2.0┬á┬▒┬á3.2
0.695ΓÇô0.918	0.0540┬á┬▒┬á6.3┬á┬▒┬á2.9┬á┬▒┬á3.5	0.0538┬á┬▒┬á6.3┬á┬▒┬á2.9┬á┬▒┬á3.5	0.0527┬á┬▒┬á4.0┬á┬▒┬á2.9┬á┬▒┬á4.3	0.0529┬á┬▒┬á4.0┬á┬▒┬á2.9┬á┬▒┬á4.3	0.0528┬á┬▒┬á4.0┬á┬▒┬á2.9┬á┬▒┬á4.3	0.0558┬á┬▒┬á3.3┬á┬▒┬á2.1┬á┬▒┬á3.2
0.918ΓÇô1.153	0.0258┬á┬▒┬á8.8┬á┬▒┬á6.1┬á┬▒┬á6.2	0.0258┬á┬▒┬á8.8┬á┬▒┬á6.1┬á┬▒┬á6.2	0.0259┬á┬▒┬á5.6┬á┬▒┬á3.3┬á┬▒┬á2.8	0.0262┬á┬▒┬á5.6┬á┬▒┬á3.3┬á┬▒┬á2.8	0.0263┬á┬▒┬á5.6┬á┬▒┬á3.3┬á┬▒┬á2.9	0.0264┬á┬▒┬á4.7┬á┬▒┬á2.9┬á┬▒┬á3.0
1.153ΓÇô1.496	0.0180┬á┬▒┬á8.2┬á┬▒┬á3.2┬á┬▒┬á2.6	0.0181┬á┬▒┬á8.2┬á┬▒┬á3.2┬á┬▒┬á2.6	0.0135┬á┬▒┬á5.8┬á┬▒┬á3.0┬á┬▒┬á4.9	0.0137┬á┬▒┬á5.8┬á┬▒┬á3.0┬á┬▒┬á4.9	0.0141┬á┬▒┬á5.8┬á┬▒┬á3.0┬á┬▒┬á5.2	0.0161┬á┬▒┬á4.6┬á┬▒┬á2.3┬á┬▒┬á3.2
1.496ΓÇô1.947	0.00804┬á┬▒┬á11┬á┬▒┬á4.5┬á┬▒┬á5.0	0.00804┬á┬▒┬á11┬á┬▒┬á4.5┬á┬▒┬á5.1	0.00542┬á┬▒┬á9.2┬á┬▒┬á4.8┬á┬▒┬á7.9	0.00546┬á┬▒┬á9.2┬á┬▒┬á4.8┬á┬▒┬á7.9	0.00537┬á┬▒┬á9.2┬á┬▒┬á4.8┬á┬▒┬á8.1	0.00681┬á┬▒┬á6.7┬á┬▒┬á3.5┬á┬▒┬á4.2
1.947ΓÇô2.522	0.00285┬á┬▒┬á19┬á┬▒┬á14┬á┬▒┬á8.4	0.00281┬á┬▒┬á19┬á┬▒┬á14┬á┬▒┬á8.4	0.00312┬á┬▒┬á9.5┬á┬▒┬á4.7┬á┬▒┬á4.0	0.00320┬á┬▒┬á9.5┬á┬▒┬á4.7┬á┬▒┬á4.0	0.00319┬á┬▒┬á9.5┬á┬▒┬á4.7┬á┬▒┬á4.3	0.00328┬á┬▒┬á8.3┬á┬▒┬á4.6┬á┬▒┬á3.7
2.522ΓÇô3.277	0.00136┬á┬▒┬á24┬á┬▒┬á12┬á┬▒┬á10	0.00134┬á┬▒┬á24┬á┬▒┬á12┬á┬▒┬á10.0	0.00260┬á┬▒┬á9.4┬á┬▒┬á5.5┬á┬▒┬á5.6	0.00262┬á┬▒┬á9.4┬á┬▒┬á5.5┬á┬▒┬á5.6	0.00259┬á┬▒┬á9.4┬á┬▒┬á5.5┬á┬▒┬á5.9	0.00246┬á┬▒┬á8.5┬á┬▒┬á5.0┬á┬▒┬á4.5
3.277ΓÇô5.000	0.000850┬á┬▒┬á19┬á┬▒┬á9.6┬á┬▒┬á6.8	0.000843┬á┬▒┬á19┬á┬▒┬á9.6┬á┬▒┬á6.8	0.000517┬á┬▒┬á15┬á┬▒┬á8.7┬á┬▒┬á10	0.000532┬á┬▒┬á15┬á┬▒┬á8.7┬á┬▒┬á10	0.000530┬á┬▒┬á15┬á┬▒┬á8.7┬á┬▒┬á10	0.000696┬á┬▒┬á11┬á┬▒┬á6.6┬á┬▒┬á5.9
5.000ΓÇô10.000	0.000285┬á┬▒┬á21┬á┬▒┬á9.8┬á┬▒┬á5.3	0.000280┬á┬▒┬á21┬á┬▒┬á9.8┬á┬▒┬á5.3	0.000215┬á┬▒┬á14┬á┬▒┬á7.2┬á┬▒┬á7.5	0.000218┬á┬▒┬á14┬á┬▒┬á7.2┬á┬▒┬á7.5	0.000226┬á┬▒┬á14┬á┬▒┬á7.2┬á┬▒┬á7.7	0.000260┬á┬▒┬á11┬á┬▒┬á5.8┬á┬▒┬á4.7

**Table 15:** The values of $(1/\sigma )\,{\mathrm {d}}\sigma /{\mathrm {d}}\phi ^*_{\eta }$ in each bin of $\phi ^*_{\eta }$ for the electron and muon channels separately (for various particle-level definitions) and for the Born-level combination in the kinematic region $116\ ~{\mathrm {GeV}}\le m_{\ell \ell }< 150\ ~{\mathrm {GeV}},\ 0.8 \le |y_{\ell \ell }| < 1.6$. The associated statistical and systematic (both uncorrelated and correlated between bins of $\phi ^*_{\eta }$) are provided in percentage form

Bin	$(1/\sigma )\,d\sigma /d\phi ^*_{\eta }$ ┬▒ Statistical [%] ┬▒ Uncorrelated systematic [%] ┬▒ Correlated systematic [%]					
	Electron channel		Muon channel			Combination
	Dressed	Born	Bare	Dressed	Born	Born
0.000ΓÇô0.004	10.972┬á┬▒┬á3.1┬á┬▒┬á1.0┬á┬▒┬á2.3	10.997┬á┬▒┬á3.1┬á┬▒┬á1.0┬á┬▒┬á2.3	12.244┬á┬▒┬á1.8┬á┬▒┬á0.8┬á┬▒┬á1.8	12.206┬á┬▒┬á1.8┬á┬▒┬á0.8┬á┬▒┬á1.8	12.288┬á┬▒┬á1.8┬á┬▒┬á0.8┬á┬▒┬á1.8	11.982┬á┬▒┬á1.6┬á┬▒┬á0.6┬á┬▒┬á1.9
0.004ΓÇô0.008	11.989┬á┬▒┬á3.0┬á┬▒┬á0.9┬á┬▒┬á1.0	12.122┬á┬▒┬á3.0┬á┬▒┬á0.9┬á┬▒┬á1.0	11.775┬á┬▒┬á1.8┬á┬▒┬á0.8┬á┬▒┬á0.9	11.843┬á┬▒┬á1.8┬á┬▒┬á0.8┬á┬▒┬á0.9	11.904┬á┬▒┬á1.8┬á┬▒┬á0.8┬á┬▒┬á0.9	11.940┬á┬▒┬á1.6┬á┬▒┬á0.6┬á┬▒┬á0.8
0.008ΓÇô0.012	10.628┬á┬▒┬á3.1┬á┬▒┬á0.9┬á┬▒┬á1.3	10.637┬á┬▒┬á3.1┬á┬▒┬á0.9┬á┬▒┬á1.3	10.892┬á┬▒┬á1.9┬á┬▒┬á0.8┬á┬▒┬á0.7	10.805┬á┬▒┬á1.9┬á┬▒┬á0.8┬á┬▒┬á0.7	10.871┬á┬▒┬á1.9┬á┬▒┬á0.8┬á┬▒┬á0.7	10.818┬á┬▒┬á1.6┬á┬▒┬á0.7┬á┬▒┬á0.7
0.012ΓÇô0.016	10.718┬á┬▒┬á3.1┬á┬▒┬á1.0┬á┬▒┬á0.8	10.832┬á┬▒┬á3.1┬á┬▒┬á1.0┬á┬▒┬á0.8	11.265┬á┬▒┬á1.9┬á┬▒┬á0.8┬á┬▒┬á0.8	11.237┬á┬▒┬á1.9┬á┬▒┬á0.8┬á┬▒┬á0.8	11.211┬á┬▒┬á1.9┬á┬▒┬á0.8┬á┬▒┬á0.8	11.085┬á┬▒┬á1.6┬á┬▒┬á0.7┬á┬▒┬á0.7
0.016ΓÇô0.020	9.871┬á┬▒┬á3.2┬á┬▒┬á0.9┬á┬▒┬á1.1	9.849┬á┬▒┬á3.2┬á┬▒┬á0.9┬á┬▒┬á1.1	9.966┬á┬▒┬á2.0┬á┬▒┬á0.9┬á┬▒┬á0.6	9.860┬á┬▒┬á2.0┬á┬▒┬á0.9┬á┬▒┬á0.6	10.009┬á┬▒┬á2.0┬á┬▒┬á0.9┬á┬▒┬á0.7	9.945┬á┬▒┬á1.7┬á┬▒┬á0.7┬á┬▒┬á0.6
0.020ΓÇô0.024	9.754┬á┬▒┬á3.3┬á┬▒┬á1.1┬á┬▒┬á0.9	9.811┬á┬▒┬á3.3┬á┬▒┬á1.1┬á┬▒┬á0.9	9.756┬á┬▒┬á2.0┬á┬▒┬á0.9┬á┬▒┬á0.7	9.737┬á┬▒┬á2.0┬á┬▒┬á0.9┬á┬▒┬á0.7	9.765┬á┬▒┬á2.0┬á┬▒┬á0.9┬á┬▒┬á0.7	9.747┬á┬▒┬á1.7┬á┬▒┬á0.7┬á┬▒┬á0.6
0.024ΓÇô0.029	8.577┬á┬▒┬á3.1┬á┬▒┬á1.0┬á┬▒┬á0.7	8.597┬á┬▒┬á3.1┬á┬▒┬á1.0┬á┬▒┬á0.7	8.647┬á┬▒┬á1.9┬á┬▒┬á0.8┬á┬▒┬á0.5	8.611┬á┬▒┬á1.9┬á┬▒┬á0.8┬á┬▒┬á0.5	8.609┬á┬▒┬á1.9┬á┬▒┬á0.8┬á┬▒┬á0.6	8.572┬á┬▒┬á1.6┬á┬▒┬á0.7┬á┬▒┬á0.5
0.029ΓÇô0.034	7.516┬á┬▒┬á3.4┬á┬▒┬á1.4┬á┬▒┬á1.7	7.495┬á┬▒┬á3.4┬á┬▒┬á1.4┬á┬▒┬á1.7	8.261┬á┬▒┬á2.0┬á┬▒┬á0.9┬á┬▒┬á0.5	8.268┬á┬▒┬á2.0┬á┬▒┬á0.9┬á┬▒┬á0.5	8.305┬á┬▒┬á2.0┬á┬▒┬á0.9┬á┬▒┬á0.6	8.038┬á┬▒┬á1.7┬á┬▒┬á0.7┬á┬▒┬á0.6
0.034ΓÇô0.039	7.643┬á┬▒┬á3.3┬á┬▒┬á1.0┬á┬▒┬á0.8	7.677┬á┬▒┬á3.3┬á┬▒┬á1.1┬á┬▒┬á0.8	7.411┬á┬▒┬á2.1┬á┬▒┬á0.9┬á┬▒┬á0.5	7.404┬á┬▒┬á2.1┬á┬▒┬á0.9┬á┬▒┬á0.5	7.432┬á┬▒┬á2.1┬á┬▒┬á0.9┬á┬▒┬á0.6	7.473┬á┬▒┬á1.8┬á┬▒┬á0.7┬á┬▒┬á0.5
0.039ΓÇô0.045	6.365┬á┬▒┬á3.3┬á┬▒┬á1.0┬á┬▒┬á0.9	6.356┬á┬▒┬á3.3┬á┬▒┬á1.0┬á┬▒┬á0.9	6.576┬á┬▒┬á2.0┬á┬▒┬á0.9┬á┬▒┬á0.4	6.583┬á┬▒┬á2.0┬á┬▒┬á0.9┬á┬▒┬á0.4	6.552┬á┬▒┬á2.0┬á┬▒┬á0.9┬á┬▒┬á0.5	6.489┬á┬▒┬á1.7┬á┬▒┬á0.7┬á┬▒┬á0.5
0.045ΓÇô0.051	6.080┬á┬▒┬á3.4┬á┬▒┬á1.0┬á┬▒┬á0.6	6.080┬á┬▒┬á3.4┬á┬▒┬á1.0┬á┬▒┬á0.6	5.937┬á┬▒┬á2.1┬á┬▒┬á0.9┬á┬▒┬á0.4	5.931┬á┬▒┬á2.1┬á┬▒┬á0.9┬á┬▒┬á0.4	5.947┬á┬▒┬á2.1┬á┬▒┬á0.9┬á┬▒┬á0.5	5.971┬á┬▒┬á1.8┬á┬▒┬á0.7┬á┬▒┬á0.5
0.051ΓÇô0.057	5.577┬á┬▒┬á3.5┬á┬▒┬á1.0┬á┬▒┬á0.6	5.578┬á┬▒┬á3.5┬á┬▒┬á1.0┬á┬▒┬á0.6	5.636┬á┬▒┬á2.1┬á┬▒┬á0.9┬á┬▒┬á0.4	5.657┬á┬▒┬á2.1┬á┬▒┬á0.9┬á┬▒┬á0.4	5.652┬á┬▒┬á2.1┬á┬▒┬á0.9┬á┬▒┬á0.6	5.616┬á┬▒┬á1.8┬á┬▒┬á0.7┬á┬▒┬á0.5
0.057ΓÇô0.064	4.794┬á┬▒┬á3.6┬á┬▒┬á1.0┬á┬▒┬á0.7	4.748┬á┬▒┬á3.6┬á┬▒┬á1.0┬á┬▒┬á0.7	4.936┬á┬▒┬á2.1┬á┬▒┬á0.9┬á┬▒┬á0.5	4.918┬á┬▒┬á2.1┬á┬▒┬á0.9┬á┬▒┬á0.5	4.927┬á┬▒┬á2.1┬á┬▒┬á0.9┬á┬▒┬á0.5	4.868┬á┬▒┬á1.8┬á┬▒┬á0.7┬á┬▒┬á0.5
0.064ΓÇô0.072	4.645┬á┬▒┬á3.4┬á┬▒┬á1.7┬á┬▒┬á0.6	4.637┬á┬▒┬á3.4┬á┬▒┬á1.8┬á┬▒┬á0.6	4.375┬á┬▒┬á2.1┬á┬▒┬á0.9┬á┬▒┬á0.6	4.370┬á┬▒┬á2.1┬á┬▒┬á0.9┬á┬▒┬á0.6	4.333┬á┬▒┬á2.1┬á┬▒┬á0.9┬á┬▒┬á0.7	4.391┬á┬▒┬á1.8┬á┬▒┬á0.8┬á┬▒┬á0.6
0.072ΓÇô0.081	4.082┬á┬▒┬á3.4┬á┬▒┬á1.1┬á┬▒┬á0.7	4.078┬á┬▒┬á3.4┬á┬▒┬á1.2┬á┬▒┬á0.7	3.774┬á┬▒┬á2.1┬á┬▒┬á0.9┬á┬▒┬á0.6	3.773┬á┬▒┬á2.1┬á┬▒┬á0.9┬á┬▒┬á0.6	3.727┬á┬▒┬á2.1┬á┬▒┬á0.9┬á┬▒┬á0.6	3.822┬á┬▒┬á1.8┬á┬▒┬á0.7┬á┬▒┬á0.6
0.081ΓÇô0.091	3.474┬á┬▒┬á3.5┬á┬▒┬á1.0┬á┬▒┬á0.8	3.457┬á┬▒┬á3.5┬á┬▒┬á1.0┬á┬▒┬á0.8	3.262┬á┬▒┬á2.2┬á┬▒┬á0.9┬á┬▒┬á0.7	3.238┬á┬▒┬á2.2┬á┬▒┬á0.9┬á┬▒┬á0.7	3.269┬á┬▒┬á2.2┬á┬▒┬á0.9┬á┬▒┬á0.7	3.329┬á┬▒┬á1.9┬á┬▒┬á0.7┬á┬▒┬á0.6
0.091ΓÇô0.102	3.205┬á┬▒┬á3.5┬á┬▒┬á1.1┬á┬▒┬á0.9	3.212┬á┬▒┬á3.5┬á┬▒┬á1.2┬á┬▒┬á0.9	3.119┬á┬▒┬á2.1┬á┬▒┬á0.9┬á┬▒┬á0.5	3.125┬á┬▒┬á2.1┬á┬▒┬á0.9┬á┬▒┬á0.5	3.116┬á┬▒┬á2.1┬á┬▒┬á0.9┬á┬▒┬á0.6	3.126┬á┬▒┬á1.8┬á┬▒┬á0.7┬á┬▒┬á0.5
0.102ΓÇô0.114	2.435┬á┬▒┬á3.9┬á┬▒┬á1.2┬á┬▒┬á0.9	2.420┬á┬▒┬á3.9┬á┬▒┬á1.3┬á┬▒┬á0.9	2.528┬á┬▒┬á2.3┬á┬▒┬á0.9┬á┬▒┬á0.6	2.533┬á┬▒┬á2.3┬á┬▒┬á0.9┬á┬▒┬á0.6	2.509┬á┬▒┬á2.3┬á┬▒┬á0.9┬á┬▒┬á0.6	2.473┬á┬▒┬á2.0┬á┬▒┬á0.8┬á┬▒┬á0.5
0.114ΓÇô0.128	2.051┬á┬▒┬á3.9┬á┬▒┬á1.2┬á┬▒┬á0.8	2.041┬á┬▒┬á3.9┬á┬▒┬á1.3┬á┬▒┬á0.8	2.071┬á┬▒┬á2.3┬á┬▒┬á1.1┬á┬▒┬á0.6	2.075┬á┬▒┬á2.3┬á┬▒┬á1.1┬á┬▒┬á0.6	2.066┬á┬▒┬á2.3┬á┬▒┬á1.1┬á┬▒┬á0.6	2.057┬á┬▒┬á2.0┬á┬▒┬á0.9┬á┬▒┬á0.5
0.128ΓÇô0.145	1.828┬á┬▒┬á3.8┬á┬▒┬á1.5┬á┬▒┬á1.7	1.823┬á┬▒┬á3.8┬á┬▒┬á1.5┬á┬▒┬á1.8	1.907┬á┬▒┬á2.2┬á┬▒┬á0.9┬á┬▒┬á0.6	1.902┬á┬▒┬á2.2┬á┬▒┬á0.9┬á┬▒┬á0.6	1.905┬á┬▒┬á2.2┬á┬▒┬á0.9┬á┬▒┬á0.6	1.886┬á┬▒┬á1.9┬á┬▒┬á0.8┬á┬▒┬á0.6
0.145ΓÇô0.165	1.608┬á┬▒┬á3.8┬á┬▒┬á1.2┬á┬▒┬á1.4	1.597┬á┬▒┬á3.8┬á┬▒┬á1.3┬á┬▒┬á1.4	1.579┬á┬▒┬á2.2┬á┬▒┬á1.1┬á┬▒┬á0.5	1.584┬á┬▒┬á2.2┬á┬▒┬á1.1┬á┬▒┬á0.5	1.580┬á┬▒┬á2.2┬á┬▒┬á1.1┬á┬▒┬á0.5	1.579┬á┬▒┬á1.9┬á┬▒┬á0.9┬á┬▒┬á0.5
0.165ΓÇô0.189	1.211┬á┬▒┬á3.9┬á┬▒┬á1.3┬á┬▒┬á1.4	1.207┬á┬▒┬á3.9┬á┬▒┬á1.3┬á┬▒┬á1.5	1.177┬á┬▒┬á2.4┬á┬▒┬á1.0┬á┬▒┬á0.8	1.177┬á┬▒┬á2.4┬á┬▒┬á1.0┬á┬▒┬á0.8	1.173┬á┬▒┬á2.4┬á┬▒┬á1.0┬á┬▒┬á0.8	1.183┬á┬▒┬á2.0┬á┬▒┬á0.8┬á┬▒┬á0.7
0.189ΓÇô0.219	0.940┬á┬▒┬á4.0┬á┬▒┬á1.8┬á┬▒┬á1.2	0.938┬á┬▒┬á4.0┬á┬▒┬á1.8┬á┬▒┬á1.3	0.970┬á┬▒┬á2.3┬á┬▒┬á1.3┬á┬▒┬á0.7	0.972┬á┬▒┬á2.3┬á┬▒┬á1.3┬á┬▒┬á0.7	0.968┬á┬▒┬á2.3┬á┬▒┬á1.3┬á┬▒┬á0.7	0.957┬á┬▒┬á2.0┬á┬▒┬á1.1┬á┬▒┬á0.6
0.219ΓÇô0.258	0.774┬á┬▒┬á4.0┬á┬▒┬á1.7┬á┬▒┬á1.1	0.773┬á┬▒┬á4.0┬á┬▒┬á1.7┬á┬▒┬á1.0	0.784┬á┬▒┬á2.3┬á┬▒┬á1.3┬á┬▒┬á0.5	0.789┬á┬▒┬á2.3┬á┬▒┬á1.3┬á┬▒┬á0.5	0.786┬á┬▒┬á2.3┬á┬▒┬á1.3┬á┬▒┬á0.5	0.780┬á┬▒┬á2.0┬á┬▒┬á1.1┬á┬▒┬á0.5
0.258ΓÇô0.312	0.547┬á┬▒┬á4.1┬á┬▒┬á1.6┬á┬▒┬á2.4	0.546┬á┬▒┬á4.1┬á┬▒┬á1.6┬á┬▒┬á2.4	0.543┬á┬▒┬á2.3┬á┬▒┬á0.9┬á┬▒┬á1.6	0.545┬á┬▒┬á2.3┬á┬▒┬á0.9┬á┬▒┬á1.6	0.542┬á┬▒┬á2.3┬á┬▒┬á0.9┬á┬▒┬á1.6	0.547┬á┬▒┬á2.0┬á┬▒┬á0.8┬á┬▒┬á1.2
0.312ΓÇô0.391	0.355┬á┬▒┬á4.3┬á┬▒┬á1.8┬á┬▒┬á2.3	0.354┬á┬▒┬á4.3┬á┬▒┬á1.8┬á┬▒┬á2.3	0.329┬á┬▒┬á2.6┬á┬▒┬á1.1┬á┬▒┬á2.0	0.330┬á┬▒┬á2.6┬á┬▒┬á1.1┬á┬▒┬á2.0	0.330┬á┬▒┬á2.6┬á┬▒┬á1.1┬á┬▒┬á2.0	0.341┬á┬▒┬á2.2┬á┬▒┬á0.9┬á┬▒┬á1.5
0.391ΓÇô0.524	0.212┬á┬▒┬á4.4┬á┬▒┬á2.0┬á┬▒┬á2.1	0.212┬á┬▒┬á4.4┬á┬▒┬á2.0┬á┬▒┬á2.1	0.186┬á┬▒┬á2.6┬á┬▒┬á1.1┬á┬▒┬á2.0	0.188┬á┬▒┬á2.6┬á┬▒┬á1.1┬á┬▒┬á2.0	0.188┬á┬▒┬á2.6┬á┬▒┬á1.1┬á┬▒┬á2.0	0.195┬á┬▒┬á2.3┬á┬▒┬á1.0┬á┬▒┬á1.7
0.524ΓÇô0.695	0.101┬á┬▒┬á5.9┬á┬▒┬á2.8┬á┬▒┬á3.0	0.101┬á┬▒┬á5.9┬á┬▒┬á2.8┬á┬▒┬á3.0	0.0959┬á┬▒┬á3.4┬á┬▒┬á1.9┬á┬▒┬á1.8	0.0964┬á┬▒┬á3.4┬á┬▒┬á1.9┬á┬▒┬á1.8	0.0969┬á┬▒┬á3.4┬á┬▒┬á1.9┬á┬▒┬á1.8	0.0977┬á┬▒┬á2.9┬á┬▒┬á1.6┬á┬▒┬á1.8
0.695ΓÇô0.918	0.0504┬á┬▒┬á7.3┬á┬▒┬á3.3┬á┬▒┬á3.1	0.0507┬á┬▒┬á7.3┬á┬▒┬á3.3┬á┬▒┬á3.1	0.0475┬á┬▒┬á4.3┬á┬▒┬á2.2┬á┬▒┬á2.5	0.0477┬á┬▒┬á4.3┬á┬▒┬á2.2┬á┬▒┬á2.5	0.0473┬á┬▒┬á4.3┬á┬▒┬á2.2┬á┬▒┬á2.5	0.0486┬á┬▒┬á3.7┬á┬▒┬á1.9┬á┬▒┬á2.3
0.918ΓÇô1.153	0.0254┬á┬▒┬á9.5┬á┬▒┬á3.9┬á┬▒┬á5.0	0.0252┬á┬▒┬á9.5┬á┬▒┬á4.0┬á┬▒┬á5.0	0.0247┬á┬▒┬á5.6┬á┬▒┬á2.7┬á┬▒┬á2.2	0.0251┬á┬▒┬á5.6┬á┬▒┬á2.7┬á┬▒┬á2.2	0.0251┬á┬▒┬á5.6┬á┬▒┬á2.7┬á┬▒┬á2.2	0.0250┬á┬▒┬á4.9┬á┬▒┬á2.2┬á┬▒┬á2.2
1.153ΓÇô1.496	0.0114┬á┬▒┬á12┬á┬▒┬á12┬á┬▒┬á25	0.0115┬á┬▒┬á12┬á┬▒┬á12┬á┬▒┬á25	0.0109┬á┬▒┬á7.1┬á┬▒┬á3.5┬á┬▒┬á2.5	0.0111┬á┬▒┬á7.1┬á┬▒┬á3.5┬á┬▒┬á2.5	0.0107┬á┬▒┬á7.1┬á┬▒┬á3.5┬á┬▒┬á2.6	0.0108┬á┬▒┬á6.3┬á┬▒┬á3.6┬á┬▒┬á3.4
1.496ΓÇô1.947	0.00523┬á┬▒┬á15┬á┬▒┬á6.7┬á┬▒┬á5.6	0.00520┬á┬▒┬á15┬á┬▒┬á6.7┬á┬▒┬á5.6	0.00588┬á┬▒┬á7.9┬á┬▒┬á4.1┬á┬▒┬á2.4	0.00597┬á┬▒┬á7.9┬á┬▒┬á4.1┬á┬▒┬á2.4	0.00592┬á┬▒┬á7.9┬á┬▒┬á4.1┬á┬▒┬á2.5	0.00579┬á┬▒┬á7.0┬á┬▒┬á3.5┬á┬▒┬á2.5
1.947ΓÇô2.522	0.00236┬á┬▒┬á20┬á┬▒┬á8.6┬á┬▒┬á8.2	0.00237┬á┬▒┬á20┬á┬▒┬á8.6┬á┬▒┬á8.2	0.00407┬á┬▒┬á8.9┬á┬▒┬á4.3┬á┬▒┬á4.6	0.00407┬á┬▒┬á8.9┬á┬▒┬á4.3┬á┬▒┬á4.6	0.00403┬á┬▒┬á8.9┬á┬▒┬á4.3┬á┬▒┬á4.7	0.00374┬á┬▒┬á8.0┬á┬▒┬á3.9┬á┬▒┬á3.6
2.522ΓÇô3.277	0.00147┬á┬▒┬á22┬á┬▒┬á9.3┬á┬▒┬á10.0	0.00153┬á┬▒┬á22┬á┬▒┬á9.4┬á┬▒┬á10	0.00124┬á┬▒┬á15┬á┬▒┬á7.3┬á┬▒┬á4.2	0.00128┬á┬▒┬á15┬á┬▒┬á7.3┬á┬▒┬á4.2	0.00129┬á┬▒┬á15┬á┬▒┬á7.3┬á┬▒┬á4.2	0.00138┬á┬▒┬á12┬á┬▒┬á5.8┬á┬▒┬á4.1
3.277ΓÇô5.000	0.000654┬á┬▒┬á22┬á┬▒┬á13┬á┬▒┬á9.8	0.000644┬á┬▒┬á22┬á┬▒┬á13┬á┬▒┬á9.9	0.000505┬á┬▒┬á15┬á┬▒┬á7.9┬á┬▒┬á6.5	0.000511┬á┬▒┬á15┬á┬▒┬á7.9┬á┬▒┬á6.5	0.000506┬á┬▒┬á15┬á┬▒┬á7.9┬á┬▒┬á6.5	0.000559┬á┬▒┬á12┬á┬▒┬á6.8┬á┬▒┬á5.0
5.000ΓÇô10.000	0.000228┬á┬▒┬á21┬á┬▒┬á8.2┬á┬▒┬á9.0	0.000230┬á┬▒┬á21┬á┬▒┬á8.3┬á┬▒┬á8.7	0.000168┬á┬▒┬á16┬á┬▒┬á8.2┬á┬▒┬á2.4	0.000171┬á┬▒┬á16┬á┬▒┬á8.2┬á┬▒┬á2.4	0.000169┬á┬▒┬á16┬á┬▒┬á8.2┬á┬▒┬á2.5	0.000187┬á┬▒┬á13┬á┬▒┬á6.2┬á┬▒┬á3.0

**Table 16:** The values of $(1/\sigma )\,{\mathrm {d}}\sigma /{\mathrm {d}}\phi ^*_{\eta }$ in each bin of $\phi ^*_{\eta }$ for the electron and muon channels separately (for various particle-level definitions) and for the Born-level combination in the kinematic region $116\ ~{\mathrm {GeV}}\le m_{\ell \ell }< 150\ ~{\mathrm {GeV}},\ 1.6 \le |y_{\ell \ell }| < 2.4$. The associated statistical and systematic (both uncorrelated and correlated between bins of $\phi ^*_{\eta }$) are provided in percentage form

Bin	$(1/\sigma )\,d\sigma /d\phi ^*_{\eta }$ ┬▒ Statistical [%] ┬▒ Uncorrelated systematic [%] ┬▒ Correlated systematic [%]					
	Electron channel		Muon channel			Combination
	Dressed	Born	Bare	Dressed	Born	Born
0.000ΓÇô0.004	11.892┬á┬▒┬á5.1┬á┬▒┬á1.6┬á┬▒┬á1.5	12.002┬á┬▒┬á5.1┬á┬▒┬á1.7┬á┬▒┬á1.5	11.291┬á┬▒┬á2.9┬á┬▒┬á1.2┬á┬▒┬á1.3	11.291┬á┬▒┬á2.9┬á┬▒┬á1.2┬á┬▒┬á1.3	11.263┬á┬▒┬á2.9┬á┬▒┬á1.2┬á┬▒┬á1.4	11.481┬á┬▒┬á2.5┬á┬▒┬á1.0┬á┬▒┬á1.2
0.004ΓÇô0.008	11.648┬á┬▒┬á5.2┬á┬▒┬á2.7┬á┬▒┬á1.8	11.765┬á┬▒┬á5.2┬á┬▒┬á2.8┬á┬▒┬á1.8	11.245┬á┬▒┬á2.9┬á┬▒┬á1.2┬á┬▒┬á0.7	11.207┬á┬▒┬á2.9┬á┬▒┬á1.2┬á┬▒┬á0.7	11.271┬á┬▒┬á2.9┬á┬▒┬á1.2┬á┬▒┬á0.9	11.391┬á┬▒┬á2.6┬á┬▒┬á1.1┬á┬▒┬á0.8
0.008ΓÇô0.012	11.455┬á┬▒┬á5.0┬á┬▒┬á1.5┬á┬▒┬á1.0	11.528┬á┬▒┬á5.0┬á┬▒┬á1.6┬á┬▒┬á1.0	11.166┬á┬▒┬á2.9┬á┬▒┬á1.2┬á┬▒┬á0.6	11.164┬á┬▒┬á2.9┬á┬▒┬á1.2┬á┬▒┬á0.6	11.356┬á┬▒┬á2.9┬á┬▒┬á1.2┬á┬▒┬á0.8	11.419┬á┬▒┬á2.5┬á┬▒┬á1.0┬á┬▒┬á0.7
0.012ΓÇô0.016	11.171┬á┬▒┬á5.1┬á┬▒┬á1.6┬á┬▒┬á1.0	11.159┬á┬▒┬á5.1┬á┬▒┬á1.7┬á┬▒┬á1.0	10.965┬á┬▒┬á2.9┬á┬▒┬á1.2┬á┬▒┬á0.8	10.902┬á┬▒┬á2.9┬á┬▒┬á1.2┬á┬▒┬á0.8	10.904┬á┬▒┬á2.9┬á┬▒┬á1.2┬á┬▒┬á1.0	10.991┬á┬▒┬á2.6┬á┬▒┬á1.0┬á┬▒┬á0.8
0.016ΓÇô0.020	9.714┬á┬▒┬á5.6┬á┬▒┬á1.9┬á┬▒┬á1.2	9.678┬á┬▒┬á5.6┬á┬▒┬á2.0┬á┬▒┬á1.2	10.150┬á┬▒┬á3.1┬á┬▒┬á1.3┬á┬▒┬á0.8	10.200┬á┬▒┬á3.1┬á┬▒┬á1.3┬á┬▒┬á0.8	10.303┬á┬▒┬á3.1┬á┬▒┬á1.3┬á┬▒┬á1.0	10.167┬á┬▒┬á2.7┬á┬▒┬á1.1┬á┬▒┬á0.8
0.020ΓÇô0.024	9.337┬á┬▒┬á5.7┬á┬▒┬á2.5┬á┬▒┬á1.9	9.362┬á┬▒┬á5.7┬á┬▒┬á2.5┬á┬▒┬á1.8	10.034┬á┬▒┬á3.1┬á┬▒┬á1.3┬á┬▒┬á0.5	10.084┬á┬▒┬á3.1┬á┬▒┬á1.3┬á┬▒┬á0.5	9.997┬á┬▒┬á3.1┬á┬▒┬á1.3┬á┬▒┬á0.7	9.850┬á┬▒┬á2.7┬á┬▒┬á1.1┬á┬▒┬á0.7
0.024ΓÇô0.029	9.075┬á┬▒┬á5.1┬á┬▒┬á2.4┬á┬▒┬á1.7	9.100┬á┬▒┬á5.1┬á┬▒┬á2.4┬á┬▒┬á1.7	9.051┬á┬▒┬á2.9┬á┬▒┬á1.2┬á┬▒┬á0.3	9.019┬á┬▒┬á2.9┬á┬▒┬á1.2┬á┬▒┬á0.3	9.027┬á┬▒┬á2.9┬á┬▒┬á1.2┬á┬▒┬á0.4	9.049┬á┬▒┬á2.5┬á┬▒┬á1.1┬á┬▒┬á0.5
0.029ΓÇô0.034	7.757┬á┬▒┬á5.6┬á┬▒┬á1.9┬á┬▒┬á1.1	7.764┬á┬▒┬á5.6┬á┬▒┬á1.9┬á┬▒┬á1.1	8.218┬á┬▒┬á3.1┬á┬▒┬á1.2┬á┬▒┬á0.6	8.239┬á┬▒┬á3.1┬á┬▒┬á1.2┬á┬▒┬á0.6	8.263┬á┬▒┬á3.1┬á┬▒┬á1.2┬á┬▒┬á0.7	8.151┬á┬▒┬á2.7┬á┬▒┬á1.1┬á┬▒┬á0.6
0.034ΓÇô0.039	7.263┬á┬▒┬á5.7┬á┬▒┬á1.9┬á┬▒┬á1.6	7.270┬á┬▒┬á5.7┬á┬▒┬á2.0┬á┬▒┬á1.6	7.469┬á┬▒┬á3.2┬á┬▒┬á1.3┬á┬▒┬á0.3	7.356┬á┬▒┬á3.2┬á┬▒┬á1.3┬á┬▒┬á0.3	7.515┬á┬▒┬á3.2┬á┬▒┬á1.3┬á┬▒┬á0.5	7.462┬á┬▒┬á2.8┬á┬▒┬á1.1┬á┬▒┬á0.5
0.039ΓÇô0.045	6.336┬á┬▒┬á5.6┬á┬▒┬á1.8┬á┬▒┬á1.3	6.324┬á┬▒┬á5.6┬á┬▒┬á1.8┬á┬▒┬á1.3	6.501┬á┬▒┬á3.2┬á┬▒┬á1.3┬á┬▒┬á0.5	6.463┬á┬▒┬á3.2┬á┬▒┬á1.3┬á┬▒┬á0.5	6.425┬á┬▒┬á3.2┬á┬▒┬á1.3┬á┬▒┬á0.6	6.409┬á┬▒┬á2.7┬á┬▒┬á1.1┬á┬▒┬á0.5
0.045ΓÇô0.051	6.574┬á┬▒┬á5.5┬á┬▒┬á1.9┬á┬▒┬á1.4	6.582┬á┬▒┬á5.5┬á┬▒┬á1.9┬á┬▒┬á1.4	5.615┬á┬▒┬á3.3┬á┬▒┬á1.3┬á┬▒┬á0.9	5.675┬á┬▒┬á3.3┬á┬▒┬á1.3┬á┬▒┬á0.9	5.648┬á┬▒┬á3.3┬á┬▒┬á1.3┬á┬▒┬á1.0	5.886┬á┬▒┬á2.9┬á┬▒┬á1.1┬á┬▒┬á0.8
0.051ΓÇô0.057	5.317┬á┬▒┬á6.0┬á┬▒┬á2.0┬á┬▒┬á1.3	5.312┬á┬▒┬á6.0┬á┬▒┬á2.0┬á┬▒┬á1.3	5.470┬á┬▒┬á3.3┬á┬▒┬á1.3┬á┬▒┬á0.6	5.445┬á┬▒┬á3.3┬á┬▒┬á1.3┬á┬▒┬á0.6	5.424┬á┬▒┬á3.3┬á┬▒┬á1.3┬á┬▒┬á0.6	5.405┬á┬▒┬á2.9┬á┬▒┬á1.1┬á┬▒┬á0.6
0.057ΓÇô0.064	4.755┬á┬▒┬á6.0┬á┬▒┬á2.0┬á┬▒┬á1.5	4.707┬á┬▒┬á6.0┬á┬▒┬á2.1┬á┬▒┬á1.6	5.408┬á┬▒┬á3.2┬á┬▒┬á1.3┬á┬▒┬á0.9	5.424┬á┬▒┬á3.2┬á┬▒┬á1.3┬á┬▒┬á0.9	5.460┬á┬▒┬á3.2┬á┬▒┬á1.3┬á┬▒┬á1.0	5.277┬á┬▒┬á2.8┬á┬▒┬á1.1┬á┬▒┬á0.8
0.064ΓÇô0.072	4.983┬á┬▒┬á5.5┬á┬▒┬á1.8┬á┬▒┬á1.6	5.002┬á┬▒┬á5.5┬á┬▒┬á1.8┬á┬▒┬á1.6	4.468┬á┬▒┬á3.4┬á┬▒┬á1.4┬á┬▒┬á0.6	4.460┬á┬▒┬á3.4┬á┬▒┬á1.4┬á┬▒┬á0.6	4.476┬á┬▒┬á3.4┬á┬▒┬á1.4┬á┬▒┬á0.7	4.619┬á┬▒┬á2.9┬á┬▒┬á1.1┬á┬▒┬á0.7
0.072ΓÇô0.081	3.911┬á┬▒┬á5.8┬á┬▒┬á2.4┬á┬▒┬á1.6	3.870┬á┬▒┬á5.8┬á┬▒┬á2.4┬á┬▒┬á1.5	4.120┬á┬▒┬á3.2┬á┬▒┬á1.3┬á┬▒┬á0.8	4.103┬á┬▒┬á3.2┬á┬▒┬á1.3┬á┬▒┬á0.8	4.090┬á┬▒┬á3.2┬á┬▒┬á1.3┬á┬▒┬á0.9	4.051┬á┬▒┬á2.8┬á┬▒┬á1.1┬á┬▒┬á0.8
0.081ΓÇô0.091	3.070┬á┬▒┬á6.3┬á┬▒┬á2.4┬á┬▒┬á1.5	3.030┬á┬▒┬á6.3┬á┬▒┬á2.5┬á┬▒┬á1.5	3.441┬á┬▒┬á3.4┬á┬▒┬á1.4┬á┬▒┬á0.6	3.426┬á┬▒┬á3.4┬á┬▒┬á1.4┬á┬▒┬á0.6	3.388┬á┬▒┬á3.4┬á┬▒┬á1.4┬á┬▒┬á0.7	3.298┬á┬▒┬á3.0┬á┬▒┬á1.2┬á┬▒┬á0.6
0.091ΓÇô0.102	3.005┬á┬▒┬á6.1┬á┬▒┬á1.8┬á┬▒┬á4.8	3.011┬á┬▒┬á6.1┬á┬▒┬á1.9┬á┬▒┬á4.8	2.876┬á┬▒┬á3.5┬á┬▒┬á1.6┬á┬▒┬á0.7	2.874┬á┬▒┬á3.5┬á┬▒┬á1.6┬á┬▒┬á0.7	2.852┬á┬▒┬á3.5┬á┬▒┬á1.6┬á┬▒┬á0.8	2.902┬á┬▒┬á3.0┬á┬▒┬á1.3┬á┬▒┬á1.0
0.102ΓÇô0.114	2.530┬á┬▒┬á6.3┬á┬▒┬á1.9┬á┬▒┬á2.2	2.520┬á┬▒┬á6.3┬á┬▒┬á2.0┬á┬▒┬á2.2	2.595┬á┬▒┬á3.6┬á┬▒┬á1.4┬á┬▒┬á0.3	2.590┬á┬▒┬á3.6┬á┬▒┬á1.4┬á┬▒┬á0.3	2.595┬á┬▒┬á3.6┬á┬▒┬á1.4┬á┬▒┬á0.5	2.578┬á┬▒┬á3.1┬á┬▒┬á1.2┬á┬▒┬á0.5
0.114ΓÇô0.128	2.350┬á┬▒┬á6.1┬á┬▒┬á1.8┬á┬▒┬á2.2	2.358┬á┬▒┬á6.1┬á┬▒┬á1.9┬á┬▒┬á2.2	2.160┬á┬▒┬á3.6┬á┬▒┬á1.4┬á┬▒┬á0.6	2.157┬á┬▒┬á3.6┬á┬▒┬á1.4┬á┬▒┬á0.6	2.152┬á┬▒┬á3.6┬á┬▒┬á1.4┬á┬▒┬á0.7	2.205┬á┬▒┬á3.1┬á┬▒┬á1.2┬á┬▒┬á0.7
0.128ΓÇô0.145	1.975┬á┬▒┬á5.9┬á┬▒┬á1.6┬á┬▒┬á1.2	1.985┬á┬▒┬á5.9┬á┬▒┬á1.7┬á┬▒┬á1.2	1.806┬á┬▒┬á3.6┬á┬▒┬á1.6┬á┬▒┬á0.4	1.814┬á┬▒┬á3.6┬á┬▒┬á1.6┬á┬▒┬á0.4	1.825┬á┬▒┬á3.6┬á┬▒┬á1.6┬á┬▒┬á0.5	1.871┬á┬▒┬á3.1┬á┬▒┬á1.2┬á┬▒┬á0.5
0.145ΓÇô0.165	1.409┬á┬▒┬á6.5┬á┬▒┬á1.9┬á┬▒┬á0.8	1.406┬á┬▒┬á6.5┬á┬▒┬á1.9┬á┬▒┬á0.8	1.626┬á┬▒┬á3.5┬á┬▒┬á1.4┬á┬▒┬á0.8	1.634┬á┬▒┬á3.5┬á┬▒┬á1.4┬á┬▒┬á0.8	1.617┬á┬▒┬á3.5┬á┬▒┬á1.4┬á┬▒┬á0.8	1.562┬á┬▒┬á3.1┬á┬▒┬á1.2┬á┬▒┬á0.6
0.165ΓÇô0.189	1.252┬á┬▒┬á6.4┬á┬▒┬á2.4┬á┬▒┬á3.5	1.252┬á┬▒┬á6.4┬á┬▒┬á2.5┬á┬▒┬á3.5	1.333┬á┬▒┬á3.4┬á┬▒┬á1.4┬á┬▒┬á0.9	1.343┬á┬▒┬á3.4┬á┬▒┬á1.4┬á┬▒┬á0.9	1.327┬á┬▒┬á3.4┬á┬▒┬á1.4┬á┬▒┬á0.9	1.316┬á┬▒┬á3.0┬á┬▒┬á1.2┬á┬▒┬á0.9
0.189ΓÇô0.219	0.970┬á┬▒┬á6.6┬á┬▒┬á4.5┬á┬▒┬á4.3	0.970┬á┬▒┬á6.6┬á┬▒┬á4.6┬á┬▒┬á4.3	0.970┬á┬▒┬á3.7┬á┬▒┬á1.5┬á┬▒┬á1.0	0.963┬á┬▒┬á3.7┬á┬▒┬á1.5┬á┬▒┬á1.0	0.962┬á┬▒┬á3.7┬á┬▒┬á1.5┬á┬▒┬á1.0	0.963┬á┬▒┬á3.3┬á┬▒┬á1.5┬á┬▒┬á1.0
0.219ΓÇô0.258	0.856┬á┬▒┬á6.1┬á┬▒┬á2.3┬á┬▒┬á2.3	0.859┬á┬▒┬á6.1┬á┬▒┬á2.3┬á┬▒┬á2.3	0.789┬á┬▒┬á3.6┬á┬▒┬á1.4┬á┬▒┬á0.7	0.788┬á┬▒┬á3.6┬á┬▒┬á1.4┬á┬▒┬á0.7	0.784┬á┬▒┬á3.6┬á┬▒┬á1.4┬á┬▒┬á0.8	0.805┬á┬▒┬á3.1┬á┬▒┬á1.2┬á┬▒┬á0.8
0.258ΓÇô0.312	0.538┬á┬▒┬á6.6┬á┬▒┬á2.1┬á┬▒┬á1.1	0.535┬á┬▒┬á6.6┬á┬▒┬á2.2┬á┬▒┬á1.1	0.541┬á┬▒┬á3.7┬á┬▒┬á1.7┬á┬▒┬á1.0	0.543┬á┬▒┬á3.7┬á┬▒┬á1.7┬á┬▒┬á1.0	0.540┬á┬▒┬á3.7┬á┬▒┬á1.7┬á┬▒┬á1.2	0.541┬á┬▒┬á3.2┬á┬▒┬á1.4┬á┬▒┬á1.0
0.312ΓÇô0.391	0.356┬á┬▒┬á6.8┬á┬▒┬á2.7┬á┬▒┬á2.5	0.357┬á┬▒┬á6.8┬á┬▒┬á2.7┬á┬▒┬á2.4	0.339┬á┬▒┬á3.9┬á┬▒┬á1.6┬á┬▒┬á1.0	0.342┬á┬▒┬á3.9┬á┬▒┬á1.6┬á┬▒┬á1.0	0.341┬á┬▒┬á3.9┬á┬▒┬á1.6┬á┬▒┬á1.2	0.347┬á┬▒┬á3.3┬á┬▒┬á1.4┬á┬▒┬á1.1
0.391ΓÇô0.524	0.194┬á┬▒┬á7.4┬á┬▒┬á3.2┬á┬▒┬á2.4	0.194┬á┬▒┬á7.4┬á┬▒┬á3.2┬á┬▒┬á2.4	0.201┬á┬▒┬á4.0┬á┬▒┬á1.7┬á┬▒┬á1.5	0.199┬á┬▒┬á4.0┬á┬▒┬á1.7┬á┬▒┬á1.5	0.199┬á┬▒┬á4.0┬á┬▒┬á1.7┬á┬▒┬á1.7	0.198┬á┬▒┬á3.5┬á┬▒┬á1.5┬á┬▒┬á1.5
0.524ΓÇô0.695	0.0787┬á┬▒┬á10┬á┬▒┬á5.9┬á┬▒┬á8.2	0.0779┬á┬▒┬á10┬á┬▒┬á6.0┬á┬▒┬á8.2	0.0865┬á┬▒┬á5.4┬á┬▒┬á2.2┬á┬▒┬á1.3	0.0878┬á┬▒┬á5.4┬á┬▒┬á2.2┬á┬▒┬á1.3	0.0873┬á┬▒┬á5.4┬á┬▒┬á2.2┬á┬▒┬á1.5	0.0861┬á┬▒┬á4.7┬á┬▒┬á2.1┬á┬▒┬á1.7
0.695ΓÇô0.918	0.0465┬á┬▒┬á11┬á┬▒┬á4.1┬á┬▒┬á4.6	0.0464┬á┬▒┬á11┬á┬▒┬á4.1┬á┬▒┬á4.6	0.0440┬á┬▒┬á6.4┬á┬▒┬á2.6┬á┬▒┬á2.6	0.0448┬á┬▒┬á6.4┬á┬▒┬á2.6┬á┬▒┬á2.6	0.0450┬á┬▒┬á6.4┬á┬▒┬á2.6┬á┬▒┬á2.6	0.0454┬á┬▒┬á5.6┬á┬▒┬á2.2┬á┬▒┬á2.2
0.918ΓÇô1.153	0.0228┬á┬▒┬á16┬á┬▒┬á5.2┬á┬▒┬á4.6	0.0227┬á┬▒┬á16┬á┬▒┬á5.3┬á┬▒┬á4.6	0.0230┬á┬▒┬á8.8┬á┬▒┬á3.7┬á┬▒┬á2.8	0.0238┬á┬▒┬á8.8┬á┬▒┬á3.7┬á┬▒┬á2.8	0.0235┬á┬▒┬á8.8┬á┬▒┬á3.7┬á┬▒┬á2.9	0.0233┬á┬▒┬á7.6┬á┬▒┬á3.0┬á┬▒┬á2.3
1.153ΓÇô1.496	0.00970┬á┬▒┬á19┬á┬▒┬á6.6┬á┬▒┬á6.5	0.00955┬á┬▒┬á19┬á┬▒┬á6.7┬á┬▒┬á6.5	0.00973┬á┬▒┬á12┬á┬▒┬á5.0┬á┬▒┬á4.7	0.00960┬á┬▒┬á12┬á┬▒┬á5.0┬á┬▒┬á4.7	0.00981┬á┬▒┬á12┬á┬▒┬á5.0┬á┬▒┬á4.8	0.00994┬á┬▒┬á10┬á┬▒┬á4.0┬á┬▒┬á3.6
1.496ΓÇô1.947	0.00496┬á┬▒┬á22┬á┬▒┬á6.7┬á┬▒┬á7.6	0.00491┬á┬▒┬á22┬á┬▒┬á6.8┬á┬▒┬á8.0	0.00262┬á┬▒┬á19┬á┬▒┬á7.6┬á┬▒┬á11	0.00272┬á┬▒┬á19┬á┬▒┬á7.6┬á┬▒┬á11	0.00277┬á┬▒┬á19┬á┬▒┬á7.6┬á┬▒┬á11	0.00343┬á┬▒┬á14┬á┬▒┬á5.6┬á┬▒┬á7.0
1.947ΓÇô2.522	0.00155┬á┬▒┬á34┬á┬▒┬á9.4┬á┬▒┬á3.9	0.00155┬á┬▒┬á34┬á┬▒┬á9.5┬á┬▒┬á3.9	0.000971┬á┬▒┬á23┬á┬▒┬á9.6┬á┬▒┬á10	0.000988┬á┬▒┬á23┬á┬▒┬á9.6┬á┬▒┬á10	0.00101┬á┬▒┬á23┬á┬▒┬á9.6┬á┬▒┬á10	0.00119┬á┬▒┬á19┬á┬▒┬á7.5┬á┬▒┬á6.9
2.522ΓÇô3.277	0.000678┬á┬▒┬á44┬á┬▒┬á9.9┬á┬▒┬á9.2	0.000631┬á┬▒┬á44┬á┬▒┬á11┬á┬▒┬á9.5	0.0000976┬á┬▒┬á116┬á┬▒┬á49┬á┬▒┬á8.2	0.000101┬á┬▒┬á116┬á┬▒┬á49┬á┬▒┬á8.2	0.000106┬á┬▒┬á116┬á┬▒┬á49┬á┬▒┬á8.2	0.000458┬á┬▒┬á38┬á┬▒┬á17┬á┬▒┬á5.4
3.277ΓÇô5.000	0.0000936┬á┬▒┬á101┬á┬▒┬á83┬á┬▒┬á84	0.0000944┬á┬▒┬á101┬á┬▒┬á83┬á┬▒┬á84	0.0000533┬á┬▒┬á65┬á┬▒┬á30┬á┬▒┬á7.0	0.0000535┬á┬▒┬á65┬á┬▒┬á30┬á┬▒┬á7.0	0.0000513┬á┬▒┬á65┬á┬▒┬á30┬á┬▒┬á7.1	0.0000589┬á┬▒┬á55┬á┬▒┬á29┬á┬▒┬á12
5.000ΓÇô10.000	0.0000540┬á┬▒┬á68┬á┬▒┬á19┬á┬▒┬á38	0.0000506┬á┬▒┬á68┬á┬▒┬á19┬á┬▒┬á38	0.0000431┬á┬▒┬á37┬á┬▒┬á17┬á┬▒┬á8.4	0.0000416┬á┬▒┬á37┬á┬▒┬á17┬á┬▒┬á8.4	0.0000387┬á┬▒┬á37┬á┬▒┬á17┬á┬▒┬á8.5	0.0000404┬á┬▒┬á33┬á┬▒┬á14┬á┬▒┬á8.4

**Table 17:** The values of $(1/\sigma )\,{\mathrm {d}}\sigma /{\mathrm {d}}\phi ^*_{\eta }$ in each bin of $\phi ^*_{\eta }$ for the electron and muon channels separately (for various particle-level definitions) and for the Born-level combination in the kinematic region $46\ ~{\mathrm {GeV}}\le m_{\ell \ell }< 66\ ~{\mathrm {GeV}},\ |y_{\ell \ell }| < 2.4$. The associated statistical and systematic (both uncorrelated and correlated between bins of $\phi ^*_{\eta }$) are provided in percentage form

Bin	$(1/\sigma )\,d\sigma /d\phi ^*_{\eta }$ ┬▒ Statistical [%] ┬▒ Uncorrelated systematic [%] ┬▒ Correlated systematic [%]					
	Electron channel		Muon channel			Combination
	Dressed	Born	Bare	Dressed	Born	Born
0.000ΓÇô0.004	6.941┬á┬▒┬á1.6┬á┬▒┬á0.7┬á┬▒┬á5.3	7.435┬á┬▒┬á1.6┬á┬▒┬á0.8┬á┬▒┬á5.4	6.741┬á┬▒┬á1.3┬á┬▒┬á0.3┬á┬▒┬á4.2	6.724┬á┬▒┬á1.3┬á┬▒┬á0.3┬á┬▒┬á4.2	7.169┬á┬▒┬á1.3┬á┬▒┬á0.3┬á┬▒┬á4.4	7.655┬á┬▒┬á1.0┬á┬▒┬á0.3┬á┬▒┬á3.0
0.004ΓÇô0.008	6.819┬á┬▒┬á1.6┬á┬▒┬á0.7┬á┬▒┬á2.3	7.209┬á┬▒┬á1.6┬á┬▒┬á0.7┬á┬▒┬á2.5	6.967┬á┬▒┬á1.2┬á┬▒┬á0.4┬á┬▒┬á1.2	6.909┬á┬▒┬á1.2┬á┬▒┬á0.4┬á┬▒┬á1.2	7.307┬á┬▒┬á1.2┬á┬▒┬á0.4┬á┬▒┬á1.8	7.439┬á┬▒┬á1.0┬á┬▒┬á0.3┬á┬▒┬á1.3
0.008ΓÇô0.012	6.793┬á┬▒┬á1.6┬á┬▒┬á0.7┬á┬▒┬á1.5	7.176┬á┬▒┬á1.6┬á┬▒┬á0.7┬á┬▒┬á1.8	6.877┬á┬▒┬á1.2┬á┬▒┬á0.3┬á┬▒┬á0.6	6.827┬á┬▒┬á1.2┬á┬▒┬á0.3┬á┬▒┬á0.6	7.251┬á┬▒┬á1.2┬á┬▒┬á0.3┬á┬▒┬á1.5	7.346┬á┬▒┬á1.0┬á┬▒┬á0.3┬á┬▒┬á1.1
0.012ΓÇô0.016	6.507┬á┬▒┬á1.6┬á┬▒┬á0.7┬á┬▒┬á1.0	6.895┬á┬▒┬á1.6┬á┬▒┬á0.7┬á┬▒┬á1.5	6.851┬á┬▒┬á1.2┬á┬▒┬á0.3┬á┬▒┬á0.4	6.814┬á┬▒┬á1.2┬á┬▒┬á0.3┬á┬▒┬á0.4	7.265┬á┬▒┬á1.2┬á┬▒┬á0.3┬á┬▒┬á1.4	7.235┬á┬▒┬á0.9┬á┬▒┬á0.3┬á┬▒┬á1.1
0.016ΓÇô0.020	6.498┬á┬▒┬á1.6┬á┬▒┬á0.7┬á┬▒┬á1.2	6.844┬á┬▒┬á1.6┬á┬▒┬á0.7┬á┬▒┬á1.6	6.648┬á┬▒┬á1.2┬á┬▒┬á0.4┬á┬▒┬á0.3	6.627┬á┬▒┬á1.2┬á┬▒┬á0.4┬á┬▒┬á0.3	7.035┬á┬▒┬á1.2┬á┬▒┬á0.4┬á┬▒┬á1.4	7.039┬á┬▒┬á1.0┬á┬▒┬á0.3┬á┬▒┬á1.1
0.020ΓÇô0.024	6.428┬á┬▒┬á1.6┬á┬▒┬á0.7┬á┬▒┬á0.8	6.768┬á┬▒┬á1.6┬á┬▒┬á0.7┬á┬▒┬á1.4	6.445┬á┬▒┬á1.3┬á┬▒┬á0.4┬á┬▒┬á0.2	6.440┬á┬▒┬á1.3┬á┬▒┬á0.4┬á┬▒┬á0.2	6.774┬á┬▒┬á1.3┬á┬▒┬á0.4┬á┬▒┬á1.4	6.852┬á┬▒┬á1.0┬á┬▒┬á0.4┬á┬▒┬á1.1
0.024ΓÇô0.029	6.261┬á┬▒┬á1.4┬á┬▒┬á0.6┬á┬▒┬á0.8	6.573┬á┬▒┬á1.4┬á┬▒┬á0.6┬á┬▒┬á1.4	6.167┬á┬▒┬á1.2┬á┬▒┬á0.3┬á┬▒┬á0.3	6.145┬á┬▒┬á1.2┬á┬▒┬á0.3┬á┬▒┬á0.3	6.514┬á┬▒┬á1.2┬á┬▒┬á0.3┬á┬▒┬á1.3	6.597┬á┬▒┬á0.9┬á┬▒┬á0.3┬á┬▒┬á1.0
0.029ΓÇô0.034	5.900┬á┬▒┬á1.5┬á┬▒┬á0.6┬á┬▒┬á0.7	6.193┬á┬▒┬á1.5┬á┬▒┬á0.6┬á┬▒┬á1.3	5.940┬á┬▒┬á1.2┬á┬▒┬á0.3┬á┬▒┬á0.3	5.959┬á┬▒┬á1.2┬á┬▒┬á0.3┬á┬▒┬á0.3	6.288┬á┬▒┬á1.2┬á┬▒┬á0.3┬á┬▒┬á1.3	6.316┬á┬▒┬á0.9┬á┬▒┬á0.3┬á┬▒┬á1.0
0.034ΓÇô0.039	5.934┬á┬▒┬á1.5┬á┬▒┬á0.6┬á┬▒┬á0.8	6.232┬á┬▒┬á1.5┬á┬▒┬á0.7┬á┬▒┬á1.4	5.545┬á┬▒┬á1.2┬á┬▒┬á0.3┬á┬▒┬á0.4	5.539┬á┬▒┬á1.2┬á┬▒┬á0.3┬á┬▒┬á0.4	5.867┬á┬▒┬á1.2┬á┬▒┬á0.3┬á┬▒┬á1.3	6.041┬á┬▒┬á1.0┬á┬▒┬á0.3┬á┬▒┬á1.1
0.039ΓÇô0.045	5.324┬á┬▒┬á1.4┬á┬▒┬á0.7┬á┬▒┬á0.5	5.547┬á┬▒┬á1.4┬á┬▒┬á0.7┬á┬▒┬á1.3	5.466┬á┬▒┬á1.1┬á┬▒┬á0.3┬á┬▒┬á0.4	5.475┬á┬▒┬á1.1┬á┬▒┬á0.3┬á┬▒┬á0.4	5.753┬á┬▒┬á1.1┬á┬▒┬á0.3┬á┬▒┬á1.3	5.729┬á┬▒┬á0.9┬á┬▒┬á0.3┬á┬▒┬á1.0
0.045ΓÇô0.051	5.159┬á┬▒┬á1.4┬á┬▒┬á0.7┬á┬▒┬á0.6	5.379┬á┬▒┬á1.4┬á┬▒┬á0.8┬á┬▒┬á1.3	5.181┬á┬▒┬á1.1┬á┬▒┬á0.3┬á┬▒┬á0.6	5.208┬á┬▒┬á1.1┬á┬▒┬á0.3┬á┬▒┬á0.6	5.446┬á┬▒┬á1.1┬á┬▒┬á0.3┬á┬▒┬á1.4	5.451┬á┬▒┬á0.9┬á┬▒┬á0.3┬á┬▒┬á1.1
0.051ΓÇô0.057	4.874┬á┬▒┬á1.5┬á┬▒┬á0.6┬á┬▒┬á0.6	5.071┬á┬▒┬á1.5┬á┬▒┬á0.6┬á┬▒┬á1.3	4.960┬á┬▒┬á1.1┬á┬▒┬á0.3┬á┬▒┬á0.6	4.977┬á┬▒┬á1.1┬á┬▒┬á0.3┬á┬▒┬á0.6	5.208┬á┬▒┬á1.1┬á┬▒┬á0.3┬á┬▒┬á1.4	5.194┬á┬▒┬á0.9┬á┬▒┬á0.3┬á┬▒┬á1.1
0.057ΓÇô0.064	4.499┬á┬▒┬á1.4┬á┬▒┬á0.6┬á┬▒┬á0.6	4.661┬á┬▒┬á1.4┬á┬▒┬á0.6┬á┬▒┬á1.3	4.575┬á┬▒┬á1.1┬á┬▒┬á0.3┬á┬▒┬á0.7	4.600┬á┬▒┬á1.1┬á┬▒┬á0.3┬á┬▒┬á0.7	4.802┬á┬▒┬á1.1┬á┬▒┬á0.3┬á┬▒┬á1.4	4.781┬á┬▒┬á0.9┬á┬▒┬á0.3┬á┬▒┬á1.1
0.064ΓÇô0.072	4.231┬á┬▒┬á1.4┬á┬▒┬á0.6┬á┬▒┬á0.6	4.374┬á┬▒┬á1.4┬á┬▒┬á0.7┬á┬▒┬á1.3	4.124┬á┬▒┬á1.1┬á┬▒┬á0.3┬á┬▒┬á0.7	4.148┬á┬▒┬á1.1┬á┬▒┬á0.3┬á┬▒┬á0.7	4.313┬á┬▒┬á1.1┬á┬▒┬á0.3┬á┬▒┬á1.4	4.369┬á┬▒┬á0.9┬á┬▒┬á0.3┬á┬▒┬á1.1
0.072ΓÇô0.081	3.805┬á┬▒┬á1.4┬á┬▒┬á0.6┬á┬▒┬á0.6	3.907┬á┬▒┬á1.4┬á┬▒┬á0.6┬á┬▒┬á1.3	3.920┬á┬▒┬á1.1┬á┬▒┬á0.3┬á┬▒┬á0.8	3.945┬á┬▒┬á1.1┬á┬▒┬á0.3┬á┬▒┬á0.8	4.080┬á┬▒┬á1.1┬á┬▒┬á0.3┬á┬▒┬á1.4	4.042┬á┬▒┬á0.8┬á┬▒┬á0.3┬á┬▒┬á1.1
0.081ΓÇô0.091	3.526┬á┬▒┬á1.3┬á┬▒┬á0.7┬á┬▒┬á0.7	3.620┬á┬▒┬á1.3┬á┬▒┬á0.7┬á┬▒┬á1.3	3.494┬á┬▒┬á1.0┬á┬▒┬á0.3┬á┬▒┬á0.9	3.509┬á┬▒┬á1.0┬á┬▒┬á0.3┬á┬▒┬á0.9	3.633┬á┬▒┬á1.0┬á┬▒┬á0.3┬á┬▒┬á1.5	3.657┬á┬▒┬á0.8┬á┬▒┬á0.3┬á┬▒┬á1.1
0.091ΓÇô0.102	3.202┬á┬▒┬á1.4┬á┬▒┬á0.6┬á┬▒┬á0.6	3.273┬á┬▒┬á1.4┬á┬▒┬á0.6┬á┬▒┬á1.3	3.169┬á┬▒┬á1.0┬á┬▒┬á0.3┬á┬▒┬á0.9	3.184┬á┬▒┬á1.0┬á┬▒┬á0.3┬á┬▒┬á0.9	3.260┬á┬▒┬á1.0┬á┬▒┬á0.3┬á┬▒┬á1.5	3.289┬á┬▒┬á0.8┬á┬▒┬á0.3┬á┬▒┬á1.1
0.102ΓÇô0.114	2.856┬á┬▒┬á1.4┬á┬▒┬á0.6┬á┬▒┬á0.8	2.886┬á┬▒┬á1.4┬á┬▒┬á0.6┬á┬▒┬á1.4	2.817┬á┬▒┬á1.1┬á┬▒┬á0.3┬á┬▒┬á1.0	2.824┬á┬▒┬á1.1┬á┬▒┬á0.3┬á┬▒┬á1.0	2.871┬á┬▒┬á1.1┬á┬▒┬á0.3┬á┬▒┬á1.5	2.896┬á┬▒┬á0.8┬á┬▒┬á0.3┬á┬▒┬á1.1
0.114ΓÇô0.128	2.549┬á┬▒┬á1.3┬á┬▒┬á0.6┬á┬▒┬á0.6	2.577┬á┬▒┬á1.3┬á┬▒┬á0.6┬á┬▒┬á1.3	2.515┬á┬▒┬á1.0┬á┬▒┬á0.2┬á┬▒┬á1.1	2.524┬á┬▒┬á1.0┬á┬▒┬á0.2┬á┬▒┬á1.1	2.557┬á┬▒┬á1.0┬á┬▒┬á0.2┬á┬▒┬á1.2	2.576┬á┬▒┬á0.8┬á┬▒┬á0.2┬á┬▒┬á0.9
0.128ΓÇô0.145	2.122┬á┬▒┬á1.3┬á┬▒┬á0.5┬á┬▒┬á0.6	2.114┬á┬▒┬á1.3┬á┬▒┬á0.6┬á┬▒┬á1.3	2.183┬á┬▒┬á1.0┬á┬▒┬á0.3┬á┬▒┬á1.0	2.180┬á┬▒┬á1.0┬á┬▒┬á0.3┬á┬▒┬á1.0	2.174┬á┬▒┬á1.0┬á┬▒┬á0.3┬á┬▒┬á1.2	2.163┬á┬▒┬á0.8┬á┬▒┬á0.3┬á┬▒┬á0.9
0.145ΓÇô0.165	1.817┬á┬▒┬á1.3┬á┬▒┬á0.6┬á┬▒┬á0.8	1.787┬á┬▒┬á1.3┬á┬▒┬á0.6┬á┬▒┬á1.4	1.868┬á┬▒┬á1.0┬á┬▒┬á0.3┬á┬▒┬á1.0	1.868┬á┬▒┬á1.0┬á┬▒┬á0.3┬á┬▒┬á1.0	1.847┬á┬▒┬á1.0┬á┬▒┬á0.3┬á┬▒┬á1.2	1.834┬á┬▒┬á0.8┬á┬▒┬á0.3┬á┬▒┬á0.9
0.165ΓÇô0.189	1.512┬á┬▒┬á1.3┬á┬▒┬á0.6┬á┬▒┬á0.8	1.474┬á┬▒┬á1.3┬á┬▒┬á0.6┬á┬▒┬á1.4	1.535┬á┬▒┬á1.0┬á┬▒┬á0.3┬á┬▒┬á1.2	1.539┬á┬▒┬á1.0┬á┬▒┬á0.3┬á┬▒┬á1.2	1.495┬á┬▒┬á1.0┬á┬▒┬á0.3┬á┬▒┬á1.3	1.497┬á┬▒┬á0.8┬á┬▒┬á0.3┬á┬▒┬á0.9
0.189ΓÇô0.219	1.240┬á┬▒┬á1.3┬á┬▒┬á0.8┬á┬▒┬á0.8	1.188┬á┬▒┬á1.3┬á┬▒┬á0.8┬á┬▒┬á1.4	1.269┬á┬▒┬á1.0┬á┬▒┬á0.3┬á┬▒┬á1.1	1.269┬á┬▒┬á1.0┬á┬▒┬á0.3┬á┬▒┬á1.1	1.214┬á┬▒┬á1.0┬á┬▒┬á0.3┬á┬▒┬á1.3	1.213┬á┬▒┬á0.8┬á┬▒┬á0.3┬á┬▒┬á0.9
0.219ΓÇô0.258	0.999┬á┬▒┬á1.3┬á┬▒┬á0.6┬á┬▒┬á0.8	0.942┬á┬▒┬á1.3┬á┬▒┬á0.6┬á┬▒┬á1.4	0.987┬á┬▒┬á1.0┬á┬▒┬á0.3┬á┬▒┬á1.2	0.991┬á┬▒┬á1.0┬á┬▒┬á0.3┬á┬▒┬á1.2	0.931┬á┬▒┬á1.0┬á┬▒┬á0.3┬á┬▒┬á1.3	0.940┬á┬▒┬á0.8┬á┬▒┬á0.3┬á┬▒┬á0.9
0.258ΓÇô0.312	0.748┬á┬▒┬á1.2┬á┬▒┬á0.6┬á┬▒┬á0.8	0.685┬á┬▒┬á1.2┬á┬▒┬á0.7┬á┬▒┬á2.2	0.763┬á┬▒┬á0.9┬á┬▒┬á0.2┬á┬▒┬á1.3	0.762┬á┬▒┬á0.9┬á┬▒┬á0.2┬á┬▒┬á1.3	0.696┬á┬▒┬á0.9┬á┬▒┬á0.2┬á┬▒┬á2.4	0.704┬á┬▒┬á0.7┬á┬▒┬á0.3┬á┬▒┬á1.8
0.312ΓÇô0.391	0.545┬á┬▒┬á1.2┬á┬▒┬á0.7┬á┬▒┬á1.0	0.490┬á┬▒┬á1.2┬á┬▒┬á0.7┬á┬▒┬á2.2	0.528┬á┬▒┬á1.0┬á┬▒┬á0.3┬á┬▒┬á1.7	0.529┬á┬▒┬á1.0┬á┬▒┬á0.3┬á┬▒┬á1.7	0.469┬á┬▒┬á1.0┬á┬▒┬á0.3┬á┬▒┬á2.6	0.487┬á┬▒┬á0.8┬á┬▒┬á0.3┬á┬▒┬á1.8
0.391ΓÇô0.524	0.333┬á┬▒┬á1.2┬á┬▒┬á0.6┬á┬▒┬á1.1	0.297┬á┬▒┬á1.2┬á┬▒┬á0.6┬á┬▒┬á2.3	0.331┬á┬▒┬á1.0┬á┬▒┬á0.3┬á┬▒┬á2.1	0.330┬á┬▒┬á1.0┬á┬▒┬á0.3┬á┬▒┬á2.1	0.290┬á┬▒┬á1.0┬á┬▒┬á0.3┬á┬▒┬á2.9	0.301┬á┬▒┬á0.7┬á┬▒┬á0.3┬á┬▒┬á1.8
0.524ΓÇô0.695	0.197┬á┬▒┬á1.4┬á┬▒┬á0.9┬á┬▒┬á1.6	0.179┬á┬▒┬á1.4┬á┬▒┬á1.0┬á┬▒┬á2.6	0.187┬á┬▒┬á1.1┬á┬▒┬á0.3┬á┬▒┬á2.9	0.187┬á┬▒┬á1.1┬á┬▒┬á0.3┬á┬▒┬á2.9	0.165┬á┬▒┬á1.1┬á┬▒┬á0.3┬á┬▒┬á3.5	0.176┬á┬▒┬á0.9┬á┬▒┬á0.3┬á┬▒┬á2.0
0.695ΓÇô0.918	0.103┬á┬▒┬á1.7┬á┬▒┬á1.1┬á┬▒┬á1.5	0.0972┬á┬▒┬á1.7┬á┬▒┬á1.1┬á┬▒┬á2.5	0.105┬á┬▒┬á1.3┬á┬▒┬á0.4┬á┬▒┬á3.1	0.104┬á┬▒┬á1.3┬á┬▒┬á0.4┬á┬▒┬á3.1	0.0963┬á┬▒┬á1.3┬á┬▒┬á0.4┬á┬▒┬á3.7	0.100┬á┬▒┬á1.0┬á┬▒┬á0.4┬á┬▒┬á2.0
0.918ΓÇô1.153	0.0611┬á┬▒┬á2.2┬á┬▒┬á1.3┬á┬▒┬á1.8	0.0590┬á┬▒┬á2.2┬á┬▒┬á1.3┬á┬▒┬á2.7	0.0591┬á┬▒┬á1.8┬á┬▒┬á0.5┬á┬▒┬á3.5	0.0586┬á┬▒┬á1.8┬á┬▒┬á0.5┬á┬▒┬á3.5	0.0550┬á┬▒┬á1.8┬á┬▒┬á0.5┬á┬▒┬á4.0	0.0586┬á┬▒┬á1.4┬á┬▒┬á0.6┬á┬▒┬á2.2
1.153ΓÇô1.496	0.0333┬á┬▒┬á2.6┬á┬▒┬á2.1┬á┬▒┬á2.3	0.0324┬á┬▒┬á2.6┬á┬▒┬á2.1┬á┬▒┬á3.1	0.0320┬á┬▒┬á2.1┬á┬▒┬á1.1┬á┬▒┬á3.6	0.0315┬á┬▒┬á2.1┬á┬▒┬á1.1┬á┬▒┬á3.6	0.0303┬á┬▒┬á2.1┬á┬▒┬á1.1┬á┬▒┬á4.1	0.0322┬á┬▒┬á1.6┬á┬▒┬á1.0┬á┬▒┬á2.4
1.496ΓÇô1.947	0.0174┬á┬▒┬á3.1┬á┬▒┬á2.0┬á┬▒┬á2.5	0.0171┬á┬▒┬á3.1┬á┬▒┬á2.0┬á┬▒┬á3.3	0.0168┬á┬▒┬á2.5┬á┬▒┬á1.1┬á┬▒┬á3.0	0.0167┬á┬▒┬á2.5┬á┬▒┬á1.1┬á┬▒┬á3.0	0.0161┬á┬▒┬á2.5┬á┬▒┬á1.1┬á┬▒┬á3.5	0.0169┬á┬▒┬á1.9┬á┬▒┬á1.0┬á┬▒┬á2.4
1.947ΓÇô2.522	0.00863┬á┬▒┬á4.0┬á┬▒┬á2.3┬á┬▒┬á3.0	0.00850┬á┬▒┬á4.0┬á┬▒┬á2.3┬á┬▒┬á3.7	0.00885┬á┬▒┬á3.0┬á┬▒┬á1.2┬á┬▒┬á3.0	0.00875┬á┬▒┬á3.0┬á┬▒┬á1.2┬á┬▒┬á3.0	0.00855┬á┬▒┬á3.0┬á┬▒┬á1.2┬á┬▒┬á3.5	0.00880┬á┬▒┬á2.4┬á┬▒┬á1.1┬á┬▒┬á2.5
2.522ΓÇô3.277	0.00457┬á┬▒┬á4.6┬á┬▒┬á3.0┬á┬▒┬á5.8	0.00456┬á┬▒┬á4.6┬á┬▒┬á3.0┬á┬▒┬á6.2	0.00432┬á┬▒┬á3.8┬á┬▒┬á1.6┬á┬▒┬á3.1	0.00430┬á┬▒┬á3.8┬á┬▒┬á1.6┬á┬▒┬á3.1	0.00420┬á┬▒┬á3.8┬á┬▒┬á1.6┬á┬▒┬á3.6	0.00445┬á┬▒┬á2.9┬á┬▒┬á1.5┬á┬▒┬á2.7
3.277ΓÇô5.000	0.00207┬á┬▒┬á4.4┬á┬▒┬á2.6┬á┬▒┬á3.0	0.00206┬á┬▒┬á4.4┬á┬▒┬á2.6┬á┬▒┬á3.7	0.00187┬á┬▒┬á3.8┬á┬▒┬á1.6┬á┬▒┬á4.0	0.00187┬á┬▒┬á3.8┬á┬▒┬á1.6┬á┬▒┬á4.0	0.00183┬á┬▒┬á3.8┬á┬▒┬á1.6┬á┬▒┬á4.4	0.00198┬á┬▒┬á2.9┬á┬▒┬á1.4┬á┬▒┬á2.6
5.000ΓÇô10.000	0.000486┬á┬▒┬á5.6┬á┬▒┬á3.0┬á┬▒┬á3.3	0.000478┬á┬▒┬á5.6┬á┬▒┬á3.0┬á┬▒┬á3.9	0.000501┬á┬▒┬á4.5┬á┬▒┬á1.7┬á┬▒┬á3.9	0.000497┬á┬▒┬á4.5┬á┬▒┬á1.7┬á┬▒┬á3.9	0.000487┬á┬▒┬á4.5┬á┬▒┬á1.7┬á┬▒┬á4.3	0.000502┬á┬▒┬á3.4┬á┬▒┬á1.5┬á┬▒┬á2.7

**Table 18:** The values of $(1/\sigma )\,{\mathrm {d}}\sigma /{\mathrm {d}}\phi ^*_{\eta }$ in each bin of $\phi ^*_{\eta }$ for the electron and muon channels separately (for various particle-level definitions) and for the Born-level combination in the kinematic region $66\ ~{\mathrm {GeV}}\le m_{\ell \ell }< 116\ ~{\mathrm {GeV}},\ |y_{\ell \ell }| < 2.4$. The associated statistical and systematic (both uncorrelated and correlated between bins of $\phi ^*_{\eta }$) are provided in percentage form

Bin	$(1/\sigma )\,d\sigma /d\phi ^*_{\eta }$ ┬▒ Statistical [%] ┬▒ Uncorrelated systematic [%] ┬▒ Correlated systematic [%]					
	Electron channel		Muon channel			Combination
	Dressed	Born	Bare	Dressed	Born	Born
0.000ΓÇô0.004	9.362┬á┬▒┬á0.2┬á┬▒┬á0.1┬á┬▒┬á0.2	9.451┬á┬▒┬á0.2┬á┬▒┬á0.1┬á┬▒┬á0.2	9.364┬á┬▒┬á0.2┬á┬▒┬á0.0┬á┬▒┬á0.1	9.359┬á┬▒┬á0.2┬á┬▒┬á0.0┬á┬▒┬á0.1	9.433┬á┬▒┬á0.2┬á┬▒┬á0.0┬á┬▒┬á0.1	9.441┬á┬▒┬á0.1┬á┬▒┬á0.0┬á┬▒┬á0.1
0.004ΓÇô0.008	9.267┬á┬▒┬á0.2┬á┬▒┬á0.1┬á┬▒┬á0.1	9.352┬á┬▒┬á0.2┬á┬▒┬á0.1┬á┬▒┬á0.1	9.299┬á┬▒┬á0.2┬á┬▒┬á0.0┬á┬▒┬á0.1	9.294┬á┬▒┬á0.2┬á┬▒┬á0.0┬á┬▒┬á0.1	9.376┬á┬▒┬á0.2┬á┬▒┬á0.0┬á┬▒┬á0.1	9.365┬á┬▒┬á0.1┬á┬▒┬á0.0┬á┬▒┬á0.1
0.008ΓÇô0.012	9.094┬á┬▒┬á0.2┬á┬▒┬á0.1┬á┬▒┬á0.1	9.169┬á┬▒┬á0.2┬á┬▒┬á0.1┬á┬▒┬á0.1	9.101┬á┬▒┬á0.2┬á┬▒┬á0.0┬á┬▒┬á0.1	9.098┬á┬▒┬á0.2┬á┬▒┬á0.0┬á┬▒┬á0.1	9.173┬á┬▒┬á0.2┬á┬▒┬á0.0┬á┬▒┬á0.1	9.171┬á┬▒┬á0.1┬á┬▒┬á0.0┬á┬▒┬á0.1
0.012ΓÇô0.016	8.855┬á┬▒┬á0.2┬á┬▒┬á0.1┬á┬▒┬á0.1	8.926┬á┬▒┬á0.2┬á┬▒┬á0.1┬á┬▒┬á0.1	8.894┬á┬▒┬á0.2┬á┬▒┬á0.0┬á┬▒┬á0.0	8.888┬á┬▒┬á0.2┬á┬▒┬á0.0┬á┬▒┬á0.0	8.959┬á┬▒┬á0.2┬á┬▒┬á0.0┬á┬▒┬á0.1	8.945┬á┬▒┬á0.1┬á┬▒┬á0.0┬á┬▒┬á0.1
0.016ΓÇô0.020	8.601┬á┬▒┬á0.2┬á┬▒┬á0.1┬á┬▒┬á0.1	8.668┬á┬▒┬á0.2┬á┬▒┬á0.1┬á┬▒┬á0.1	8.562┬á┬▒┬á0.2┬á┬▒┬á0.0┬á┬▒┬á0.0	8.556┬á┬▒┬á0.2┬á┬▒┬á0.0┬á┬▒┬á0.0	8.620┬á┬▒┬á0.2┬á┬▒┬á0.0┬á┬▒┬á0.1	8.640┬á┬▒┬á0.1┬á┬▒┬á0.0┬á┬▒┬á0.1
0.020ΓÇô0.024	8.188┬á┬▒┬á0.2┬á┬▒┬á0.1┬á┬▒┬á0.1	8.238┬á┬▒┬á0.2┬á┬▒┬á0.1┬á┬▒┬á0.1	8.231┬á┬▒┬á0.2┬á┬▒┬á0.0┬á┬▒┬á0.0	8.229┬á┬▒┬á0.2┬á┬▒┬á0.0┬á┬▒┬á0.0	8.284┬á┬▒┬á0.2┬á┬▒┬á0.0┬á┬▒┬á0.1	8.264┬á┬▒┬á0.1┬á┬▒┬á0.0┬á┬▒┬á0.1
0.024ΓÇô0.029	7.825┬á┬▒┬á0.2┬á┬▒┬á0.1┬á┬▒┬á0.1	7.868┬á┬▒┬á0.2┬á┬▒┬á0.1┬á┬▒┬á0.1	7.816┬á┬▒┬á0.2┬á┬▒┬á0.0┬á┬▒┬á0.0	7.811┬á┬▒┬á0.2┬á┬▒┬á0.0┬á┬▒┬á0.0	7.861┬á┬▒┬á0.2┬á┬▒┬á0.0┬á┬▒┬á0.1	7.863┬á┬▒┬á0.1┬á┬▒┬á0.0┬á┬▒┬á0.1
0.029ΓÇô0.034	7.356┬á┬▒┬á0.2┬á┬▒┬á0.1┬á┬▒┬á0.1	7.389┬á┬▒┬á0.2┬á┬▒┬á0.1┬á┬▒┬á0.1	7.389┬á┬▒┬á0.2┬á┬▒┬á0.0┬á┬▒┬á0.0	7.384┬á┬▒┬á0.2┬á┬▒┬á0.0┬á┬▒┬á0.0	7.422┬á┬▒┬á0.2┬á┬▒┬á0.0┬á┬▒┬á0.1	7.408┬á┬▒┬á0.1┬á┬▒┬á0.0┬á┬▒┬á0.1
0.034ΓÇô0.039	6.883┬á┬▒┬á0.2┬á┬▒┬á0.1┬á┬▒┬á0.1	6.905┬á┬▒┬á0.2┬á┬▒┬á0.1┬á┬▒┬á0.1	6.883┬á┬▒┬á0.2┬á┬▒┬á0.0┬á┬▒┬á0.0	6.881┬á┬▒┬á0.2┬á┬▒┬á0.0┬á┬▒┬á0.0	6.911┬á┬▒┬á0.2┬á┬▒┬á0.0┬á┬▒┬á0.1	6.908┬á┬▒┬á0.1┬á┬▒┬á0.0┬á┬▒┬á0.1
0.039ΓÇô0.045	6.403┬á┬▒┬á0.2┬á┬▒┬á0.1┬á┬▒┬á0.1	6.419┬á┬▒┬á0.2┬á┬▒┬á0.1┬á┬▒┬á0.1	6.419┬á┬▒┬á0.2┬á┬▒┬á0.0┬á┬▒┬á0.1	6.417┬á┬▒┬á0.2┬á┬▒┬á0.0┬á┬▒┬á0.1	6.438┬á┬▒┬á0.2┬á┬▒┬á0.0┬á┬▒┬á0.1	6.429┬á┬▒┬á0.1┬á┬▒┬á0.0┬á┬▒┬á0.1
0.045ΓÇô0.051	5.871┬á┬▒┬á0.2┬á┬▒┬á0.1┬á┬▒┬á0.1	5.876┬á┬▒┬á0.2┬á┬▒┬á0.1┬á┬▒┬á0.1	5.893┬á┬▒┬á0.2┬á┬▒┬á0.0┬á┬▒┬á0.1	5.891┬á┬▒┬á0.2┬á┬▒┬á0.0┬á┬▒┬á0.1	5.899┬á┬▒┬á0.2┬á┬▒┬á0.0┬á┬▒┬á0.1	5.888┬á┬▒┬á0.1┬á┬▒┬á0.0┬á┬▒┬á0.1
0.051ΓÇô0.057	5.404┬á┬▒┬á0.2┬á┬▒┬á0.1┬á┬▒┬á0.1	5.404┬á┬▒┬á0.2┬á┬▒┬á0.1┬á┬▒┬á0.1	5.434┬á┬▒┬á0.2┬á┬▒┬á0.0┬á┬▒┬á0.0	5.434┬á┬▒┬á0.2┬á┬▒┬á0.0┬á┬▒┬á0.0	5.435┬á┬▒┬á0.2┬á┬▒┬á0.0┬á┬▒┬á0.1	5.422┬á┬▒┬á0.1┬á┬▒┬á0.0┬á┬▒┬á0.1
0.057ΓÇô0.064	4.960┬á┬▒┬á0.2┬á┬▒┬á0.1┬á┬▒┬á0.1	4.957┬á┬▒┬á0.2┬á┬▒┬á0.1┬á┬▒┬á0.1	4.975┬á┬▒┬á0.2┬á┬▒┬á0.0┬á┬▒┬á0.1	4.972┬á┬▒┬á0.2┬á┬▒┬á0.0┬á┬▒┬á0.1	4.971┬á┬▒┬á0.2┬á┬▒┬á0.0┬á┬▒┬á0.1	4.964┬á┬▒┬á0.1┬á┬▒┬á0.0┬á┬▒┬á0.1
0.064ΓÇô0.072	4.509┬á┬▒┬á0.2┬á┬▒┬á0.1┬á┬▒┬á0.1	4.502┬á┬▒┬á0.2┬á┬▒┬á0.1┬á┬▒┬á0.1	4.506┬á┬▒┬á0.2┬á┬▒┬á0.0┬á┬▒┬á0.1	4.505┬á┬▒┬á0.2┬á┬▒┬á0.0┬á┬▒┬á0.1	4.494┬á┬▒┬á0.2┬á┬▒┬á0.0┬á┬▒┬á0.1	4.496┬á┬▒┬á0.1┬á┬▒┬á0.0┬á┬▒┬á0.1
0.072ΓÇô0.081	4.022┬á┬▒┬á0.2┬á┬▒┬á0.1┬á┬▒┬á0.1	4.011┬á┬▒┬á0.2┬á┬▒┬á0.1┬á┬▒┬á0.1	4.026┬á┬▒┬á0.2┬á┬▒┬á0.0┬á┬▒┬á0.1	4.025┬á┬▒┬á0.2┬á┬▒┬á0.0┬á┬▒┬á0.1	4.013┬á┬▒┬á0.2┬á┬▒┬á0.0┬á┬▒┬á0.1	4.011┬á┬▒┬á0.1┬á┬▒┬á0.0┬á┬▒┬á0.1
0.081ΓÇô0.091	3.580┬á┬▒┬á0.2┬á┬▒┬á0.1┬á┬▒┬á0.1	3.566┬á┬▒┬á0.2┬á┬▒┬á0.1┬á┬▒┬á0.1	3.577┬á┬▒┬á0.2┬á┬▒┬á0.0┬á┬▒┬á0.1	3.577┬á┬▒┬á0.2┬á┬▒┬á0.0┬á┬▒┬á0.1	3.565┬á┬▒┬á0.2┬á┬▒┬á0.0┬á┬▒┬á0.1	3.564┬á┬▒┬á0.1┬á┬▒┬á0.0┬á┬▒┬á0.1
0.091ΓÇô0.102	3.155┬á┬▒┬á0.2┬á┬▒┬á0.1┬á┬▒┬á0.1	3.141┬á┬▒┬á0.2┬á┬▒┬á0.1┬á┬▒┬á0.1	3.154┬á┬▒┬á0.2┬á┬▒┬á0.0┬á┬▒┬á0.1	3.153┬á┬▒┬á0.2┬á┬▒┬á0.0┬á┬▒┬á0.1	3.137┬á┬▒┬á0.2┬á┬▒┬á0.0┬á┬▒┬á0.1	3.138┬á┬▒┬á0.1┬á┬▒┬á0.0┬á┬▒┬á0.1
0.102ΓÇô0.114	2.771┬á┬▒┬á0.2┬á┬▒┬á0.1┬á┬▒┬á0.1	2.758┬á┬▒┬á0.2┬á┬▒┬á0.1┬á┬▒┬á0.1	2.765┬á┬▒┬á0.2┬á┬▒┬á0.0┬á┬▒┬á0.1	2.765┬á┬▒┬á0.2┬á┬▒┬á0.0┬á┬▒┬á0.1	2.752┬á┬▒┬á0.2┬á┬▒┬á0.0┬á┬▒┬á0.1	2.753┬á┬▒┬á0.1┬á┬▒┬á0.0┬á┬▒┬á0.1
0.114ΓÇô0.128	2.394┬á┬▒┬á0.2┬á┬▒┬á0.1┬á┬▒┬á0.1	2.380┬á┬▒┬á0.2┬á┬▒┬á0.1┬á┬▒┬á0.1	2.394┬á┬▒┬á0.2┬á┬▒┬á0.0┬á┬▒┬á0.1	2.394┬á┬▒┬á0.2┬á┬▒┬á0.0┬á┬▒┬á0.1	2.380┬á┬▒┬á0.2┬á┬▒┬á0.0┬á┬▒┬á0.1	2.379┬á┬▒┬á0.1┬á┬▒┬á0.0┬á┬▒┬á0.1
0.128ΓÇô0.145	2.039┬á┬▒┬á0.2┬á┬▒┬á0.1┬á┬▒┬á0.1	2.026┬á┬▒┬á0.2┬á┬▒┬á0.1┬á┬▒┬á0.1	2.040┬á┬▒┬á0.2┬á┬▒┬á0.0┬á┬▒┬á0.1	2.040┬á┬▒┬á0.2┬á┬▒┬á0.0┬á┬▒┬á0.1	2.028┬á┬▒┬á0.2┬á┬▒┬á0.0┬á┬▒┬á0.1	2.026┬á┬▒┬á0.1┬á┬▒┬á0.0┬á┬▒┬á0.1
0.145ΓÇô0.165	1.704┬á┬▒┬á0.2┬á┬▒┬á0.1┬á┬▒┬á0.1	1.693┬á┬▒┬á0.2┬á┬▒┬á0.1┬á┬▒┬á0.1	1.701┬á┬▒┬á0.2┬á┬▒┬á0.0┬á┬▒┬á0.1	1.702┬á┬▒┬á0.2┬á┬▒┬á0.0┬á┬▒┬á0.1	1.691┬á┬▒┬á0.2┬á┬▒┬á0.0┬á┬▒┬á0.1	1.691┬á┬▒┬á0.1┬á┬▒┬á0.0┬á┬▒┬á0.1
0.165ΓÇô0.189	1.398┬á┬▒┬á0.2┬á┬▒┬á0.1┬á┬▒┬á0.1	1.389┬á┬▒┬á0.2┬á┬▒┬á0.1┬á┬▒┬á0.1	1.398┬á┬▒┬á0.2┬á┬▒┬á0.0┬á┬▒┬á0.1	1.399┬á┬▒┬á0.2┬á┬▒┬á0.0┬á┬▒┬á0.1	1.390┬á┬▒┬á0.2┬á┬▒┬á0.0┬á┬▒┬á0.1	1.389┬á┬▒┬á0.1┬á┬▒┬á0.0┬á┬▒┬á0.1
0.189ΓÇô0.219	1.117┬á┬▒┬á0.2┬á┬▒┬á0.1┬á┬▒┬á0.1	1.110┬á┬▒┬á0.2┬á┬▒┬á0.1┬á┬▒┬á0.1	1.116┬á┬▒┬á0.2┬á┬▒┬á0.0┬á┬▒┬á0.1	1.117┬á┬▒┬á0.2┬á┬▒┬á0.0┬á┬▒┬á0.1	1.110┬á┬▒┬á0.2┬á┬▒┬á0.0┬á┬▒┬á0.1	1.110┬á┬▒┬á0.1┬á┬▒┬á0.0┬á┬▒┬á0.1
0.219ΓÇô0.258	0.854┬á┬▒┬á0.2┬á┬▒┬á0.1┬á┬▒┬á0.1	0.849┬á┬▒┬á0.2┬á┬▒┬á0.1┬á┬▒┬á0.1	0.855┬á┬▒┬á0.2┬á┬▒┬á0.0┬á┬▒┬á0.1	0.856┬á┬▒┬á0.2┬á┬▒┬á0.0┬á┬▒┬á0.1	0.851┬á┬▒┬á0.2┬á┬▒┬á0.0┬á┬▒┬á0.1	0.850┬á┬▒┬á0.1┬á┬▒┬á0.0┬á┬▒┬á0.1
0.258ΓÇô0.312	0.621┬á┬▒┬á0.2┬á┬▒┬á0.1┬á┬▒┬á0.3	0.618┬á┬▒┬á0.2┬á┬▒┬á0.1┬á┬▒┬á0.3	0.618┬á┬▒┬á0.2┬á┬▒┬á0.0┬á┬▒┬á0.1	0.619┬á┬▒┬á0.2┬á┬▒┬á0.0┬á┬▒┬á0.1	0.615┬á┬▒┬á0.2┬á┬▒┬á0.0┬á┬▒┬á0.2	0.616┬á┬▒┬á0.1┬á┬▒┬á0.0┬á┬▒┬á0.2
0.312ΓÇô0.391	0.414┬á┬▒┬á0.2┬á┬▒┬á0.1┬á┬▒┬á0.3	0.412┬á┬▒┬á0.2┬á┬▒┬á0.1┬á┬▒┬á0.3	0.413┬á┬▒┬á0.2┬á┬▒┬á0.0┬á┬▒┬á0.1	0.413┬á┬▒┬á0.2┬á┬▒┬á0.0┬á┬▒┬á0.1	0.411┬á┬▒┬á0.2┬á┬▒┬á0.0┬á┬▒┬á0.2	0.411┬á┬▒┬á0.1┬á┬▒┬á0.0┬á┬▒┬á0.2
0.391ΓÇô0.524	0.241┬á┬▒┬á0.2┬á┬▒┬á0.1┬á┬▒┬á0.3	0.240┬á┬▒┬á0.2┬á┬▒┬á0.1┬á┬▒┬á0.3	0.239┬á┬▒┬á0.2┬á┬▒┬á0.0┬á┬▒┬á0.2	0.239┬á┬▒┬á0.2┬á┬▒┬á0.0┬á┬▒┬á0.2	0.238┬á┬▒┬á0.2┬á┬▒┬á0.0┬á┬▒┬á0.2	0.239┬á┬▒┬á0.1┬á┬▒┬á0.0┬á┬▒┬á0.2
0.524ΓÇô0.695	0.124┬á┬▒┬á0.3┬á┬▒┬á0.1┬á┬▒┬á0.3	0.124┬á┬▒┬á0.3┬á┬▒┬á0.1┬á┬▒┬á0.3	0.124┬á┬▒┬á0.2┬á┬▒┬á0.1┬á┬▒┬á0.2	0.124┬á┬▒┬á0.2┬á┬▒┬á0.1┬á┬▒┬á0.2	0.124┬á┬▒┬á0.2┬á┬▒┬á0.1┬á┬▒┬á0.2	0.124┬á┬▒┬á0.2┬á┬▒┬á0.1┬á┬▒┬á0.2
0.695ΓÇô0.918	0.0625┬á┬▒┬á0.3┬á┬▒┬á0.1┬á┬▒┬á0.3	0.0623┬á┬▒┬á0.3┬á┬▒┬á0.1┬á┬▒┬á0.3	0.0619┬á┬▒┬á0.3┬á┬▒┬á0.1┬á┬▒┬á0.2	0.0620┬á┬▒┬á0.3┬á┬▒┬á0.1┬á┬▒┬á0.2	0.0619┬á┬▒┬á0.3┬á┬▒┬á0.1┬á┬▒┬á0.2	0.0620┬á┬▒┬á0.2┬á┬▒┬á0.1┬á┬▒┬á0.2
0.918ΓÇô1.153	0.0322┬á┬▒┬á0.4┬á┬▒┬á0.2┬á┬▒┬á0.4	0.0321┬á┬▒┬á0.4┬á┬▒┬á0.2┬á┬▒┬á0.4	0.0320┬á┬▒┬á0.4┬á┬▒┬á0.1┬á┬▒┬á0.2	0.0320┬á┬▒┬á0.4┬á┬▒┬á0.1┬á┬▒┬á0.2	0.0319┬á┬▒┬á0.4┬á┬▒┬á0.1┬á┬▒┬á0.3	0.0320┬á┬▒┬á0.3┬á┬▒┬á0.1┬á┬▒┬á0.2
1.153ΓÇô1.496	0.0166┬á┬▒┬á0.5┬á┬▒┬á0.1┬á┬▒┬á0.4	0.0166┬á┬▒┬á0.5┬á┬▒┬á0.1┬á┬▒┬á0.4	0.0165┬á┬▒┬á0.5┬á┬▒┬á0.1┬á┬▒┬á0.2	0.0165┬á┬▒┬á0.5┬á┬▒┬á0.1┬á┬▒┬á0.2	0.0164┬á┬▒┬á0.5┬á┬▒┬á0.1┬á┬▒┬á0.3	0.0165┬á┬▒┬á0.3┬á┬▒┬á0.1┬á┬▒┬á0.3
1.496ΓÇô1.947	0.00791┬á┬▒┬á0.6┬á┬▒┬á0.2┬á┬▒┬á0.5	0.00789┬á┬▒┬á0.6┬á┬▒┬á0.2┬á┬▒┬á0.5	0.00796┬á┬▒┬á0.6┬á┬▒┬á0.1┬á┬▒┬á0.3	0.00798┬á┬▒┬á0.6┬á┬▒┬á0.1┬á┬▒┬á0.3	0.00796┬á┬▒┬á0.6┬á┬▒┬á0.1┬á┬▒┬á0.3	0.00791┬á┬▒┬á0.4┬á┬▒┬á0.1┬á┬▒┬á0.3
1.947ΓÇô2.522	0.00392┬á┬▒┬á0.8┬á┬▒┬á0.2┬á┬▒┬á0.5	0.00390┬á┬▒┬á0.8┬á┬▒┬á0.2┬á┬▒┬á0.5	0.00390┬á┬▒┬á0.7┬á┬▒┬á0.2┬á┬▒┬á0.3	0.00391┬á┬▒┬á0.7┬á┬▒┬á0.2┬á┬▒┬á0.3	0.00390┬á┬▒┬á0.7┬á┬▒┬á0.2┬á┬▒┬á0.4	0.00389┬á┬▒┬á0.5┬á┬▒┬á0.1┬á┬▒┬á0.3
2.522ΓÇô3.277	0.00198┬á┬▒┬á1.0┬á┬▒┬á0.2┬á┬▒┬á0.6	0.00198┬á┬▒┬á1.0┬á┬▒┬á0.2┬á┬▒┬á0.6	0.00194┬á┬▒┬á0.9┬á┬▒┬á0.2┬á┬▒┬á0.3	0.00195┬á┬▒┬á0.9┬á┬▒┬á0.2┬á┬▒┬á0.3	0.00194┬á┬▒┬á0.9┬á┬▒┬á0.2┬á┬▒┬á0.4	0.00195┬á┬▒┬á0.7┬á┬▒┬á0.2┬á┬▒┬á0.3
3.277ΓÇô5.000	0.000860┬á┬▒┬á1.0┬á┬▒┬á0.3┬á┬▒┬á0.7	0.000859┬á┬▒┬á1.0┬á┬▒┬á0.3┬á┬▒┬á0.7	0.000861┬á┬▒┬á0.9┬á┬▒┬á0.2┬á┬▒┬á0.3	0.000863┬á┬▒┬á0.9┬á┬▒┬á0.2┬á┬▒┬á0.3	0.000864┬á┬▒┬á0.9┬á┬▒┬á0.2┬á┬▒┬á0.3	0.000859┬á┬▒┬á0.7┬á┬▒┬á0.2┬á┬▒┬á0.3
5.000ΓÇô10.000	0.000255┬á┬▒┬á1.1┬á┬▒┬á0.3┬á┬▒┬á0.7	0.000255┬á┬▒┬á1.1┬á┬▒┬á0.3┬á┬▒┬á0.7	0.000247┬á┬▒┬á1.0┬á┬▒┬á0.2┬á┬▒┬á0.3	0.000247┬á┬▒┬á1.0┬á┬▒┬á0.2┬á┬▒┬á0.3	0.000247┬á┬▒┬á1.0┬á┬▒┬á0.2┬á┬▒┬á0.4	0.000250┬á┬▒┬á0.7┬á┬▒┬á0.2┬á┬▒┬á0.4

**Table 19:** The values of $(1/\sigma )\,{\mathrm {d}}\sigma /{\mathrm {d}}\phi ^*_{\eta }$ in each bin of $\phi ^*_{\eta }$ for the electron and muon channels separately (for various particle-level definitions) and for the Born-level combination in the kinematic region $116\ ~{\mathrm {GeV}}\le m_{\ell \ell }< 150\ ~{\mathrm {GeV}},\ |y_{\ell \ell }| < 2.4$. The associated statistical and systematic (both uncorrelated and correlated between bins of $\phi ^*_{\eta }$) are provided in percentage form

Bin	$(1/\sigma )\,d\sigma /d\phi ^*_{\eta }$ ┬▒ Statistical [%] ┬▒ Uncorrelated systematic [%] ┬▒ Correlated systematic [%]					
	Electron channel		Muon channel			Combination
	Dressed	Born	Bare	Dressed	Born	Born
0.000ΓÇô0.004	11.378┬á┬▒┬á1.9┬á┬▒┬á0.6┬á┬▒┬á2.4	11.447┬á┬▒┬á1.9┬á┬▒┬á0.6┬á┬▒┬á2.4	11.623┬á┬▒┬á1.6┬á┬▒┬á0.4┬á┬▒┬á2.1	11.601┬á┬▒┬á1.6┬á┬▒┬á0.4┬á┬▒┬á2.1	11.676┬á┬▒┬á1.6┬á┬▒┬á0.4┬á┬▒┬á2.2	11.634┬á┬▒┬á1.2┬á┬▒┬á0.3┬á┬▒┬á2.1
0.004ΓÇô0.008	11.623┬á┬▒┬á1.8┬á┬▒┬á0.6┬á┬▒┬á1.0	11.722┬á┬▒┬á1.8┬á┬▒┬á0.7┬á┬▒┬á1.0	11.447┬á┬▒┬á1.6┬á┬▒┬á0.4┬á┬▒┬á0.6	11.449┬á┬▒┬á1.6┬á┬▒┬á0.4┬á┬▒┬á0.6	11.472┬á┬▒┬á1.6┬á┬▒┬á0.4┬á┬▒┬á0.9	11.593┬á┬▒┬á1.2┬á┬▒┬á0.4┬á┬▒┬á0.8
0.008ΓÇô0.012	10.950┬á┬▒┬á1.9┬á┬▒┬á0.6┬á┬▒┬á0.8	10.983┬á┬▒┬á1.9┬á┬▒┬á0.6┬á┬▒┬á0.8	10.949┬á┬▒┬á1.6┬á┬▒┬á0.4┬á┬▒┬á0.3	10.880┬á┬▒┬á1.6┬á┬▒┬á0.4┬á┬▒┬á0.3	11.003┬á┬▒┬á1.6┬á┬▒┬á0.4┬á┬▒┬á0.7	10.994┬á┬▒┬á1.2┬á┬▒┬á0.3┬á┬▒┬á0.6
0.012ΓÇô0.016	10.819┬á┬▒┬á1.9┬á┬▒┬á0.6┬á┬▒┬á0.7	10.908┬á┬▒┬á1.9┬á┬▒┬á0.6┬á┬▒┬á0.7	11.071┬á┬▒┬á1.7┬á┬▒┬á0.4┬á┬▒┬á0.2	11.018┬á┬▒┬á1.7┬á┬▒┬á0.4┬á┬▒┬á0.2	10.992┬á┬▒┬á1.7┬á┬▒┬á0.4┬á┬▒┬á0.6	10.948┬á┬▒┬á1.2┬á┬▒┬á0.3┬á┬▒┬á0.5
0.016ΓÇô0.020	9.950┬á┬▒┬á2.0┬á┬▒┬á0.6┬á┬▒┬á0.6	9.968┬á┬▒┬á2.0┬á┬▒┬á0.6┬á┬▒┬á0.6	10.060┬á┬▒┬á1.7┬á┬▒┬á0.5┬á┬▒┬á0.3	9.989┬á┬▒┬á1.7┬á┬▒┬á0.5┬á┬▒┬á0.3	10.094┬á┬▒┬á1.7┬á┬▒┬á0.5┬á┬▒┬á0.7	10.022┬á┬▒┬á1.3┬á┬▒┬á0.4┬á┬▒┬á0.5
0.020ΓÇô0.024	9.516┬á┬▒┬á2.0┬á┬▒┬á0.6┬á┬▒┬á0.6	9.538┬á┬▒┬á2.0┬á┬▒┬á0.6┬á┬▒┬á0.6	9.632┬á┬▒┬á1.8┬á┬▒┬á0.4┬á┬▒┬á0.3	9.619┬á┬▒┬á1.8┬á┬▒┬á0.4┬á┬▒┬á0.3	9.636┬á┬▒┬á1.8┬á┬▒┬á0.4┬á┬▒┬á0.7	9.585┬á┬▒┬á1.3┬á┬▒┬á0.3┬á┬▒┬á0.5
0.024ΓÇô0.029	8.890┬á┬▒┬á1.9┬á┬▒┬á0.5┬á┬▒┬á0.6	8.922┬á┬▒┬á1.9┬á┬▒┬á0.6┬á┬▒┬á0.6	8.811┬á┬▒┬á1.6┬á┬▒┬á0.4┬á┬▒┬á0.3	8.783┬á┬▒┬á1.6┬á┬▒┬á0.4┬á┬▒┬á0.3	8.818┬á┬▒┬á1.6┬á┬▒┬á0.4┬á┬▒┬á0.5	8.863┬á┬▒┬á1.2┬á┬▒┬á0.3┬á┬▒┬á0.4
0.029ΓÇô0.034	7.962┬á┬▒┬á2.0┬á┬▒┬á0.7┬á┬▒┬á0.7	7.970┬á┬▒┬á2.0┬á┬▒┬á0.7┬á┬▒┬á0.7	8.308┬á┬▒┬á1.7┬á┬▒┬á0.4┬á┬▒┬á0.4	8.344┬á┬▒┬á1.7┬á┬▒┬á0.4┬á┬▒┬á0.4	8.373┬á┬▒┬á1.7┬á┬▒┬á0.4┬á┬▒┬á0.5	8.201┬á┬▒┬á1.3┬á┬▒┬á0.4┬á┬▒┬á0.5
0.034ΓÇô0.039	7.172┬á┬▒┬á2.1┬á┬▒┬á0.6┬á┬▒┬á0.5	7.175┬á┬▒┬á2.1┬á┬▒┬á0.7┬á┬▒┬á0.5	7.551┬á┬▒┬á1.8┬á┬▒┬á0.4┬á┬▒┬á0.4	7.526┬á┬▒┬á1.8┬á┬▒┬á0.4┬á┬▒┬á0.4	7.576┬á┬▒┬á1.8┬á┬▒┬á0.4┬á┬▒┬á0.5	7.393┬á┬▒┬á1.3┬á┬▒┬á0.4┬á┬▒┬á0.5
0.039ΓÇô0.045	6.523┬á┬▒┬á2.0┬á┬▒┬á0.6┬á┬▒┬á0.6	6.520┬á┬▒┬á2.0┬á┬▒┬á0.7┬á┬▒┬á0.6	6.667┬á┬▒┬á1.6┬á┬▒┬á0.4┬á┬▒┬á0.5	6.672┬á┬▒┬á1.6┬á┬▒┬á0.4┬á┬▒┬á0.5	6.645┬á┬▒┬á1.6┬á┬▒┬á0.4┬á┬▒┬á0.6	6.587┬á┬▒┬á1.3┬á┬▒┬á0.4┬á┬▒┬á0.5
0.045ΓÇô0.051	6.099┬á┬▒┬á2.1┬á┬▒┬á0.6┬á┬▒┬á0.6	6.099┬á┬▒┬á2.1┬á┬▒┬á0.6┬á┬▒┬á0.6	5.978┬á┬▒┬á1.8┬á┬▒┬á0.4┬á┬▒┬á0.5	5.971┬á┬▒┬á1.8┬á┬▒┬á0.4┬á┬▒┬á0.5	5.998┬á┬▒┬á1.8┬á┬▒┬á0.4┬á┬▒┬á0.6	6.034┬á┬▒┬á1.4┬á┬▒┬á0.3┬á┬▒┬á0.5
0.051ΓÇô0.057	5.466┬á┬▒┬á2.2┬á┬▒┬á0.6┬á┬▒┬á0.6	5.442┬á┬▒┬á2.2┬á┬▒┬á0.7┬á┬▒┬á0.6	5.484┬á┬▒┬á1.9┬á┬▒┬á0.4┬á┬▒┬á0.5	5.480┬á┬▒┬á1.9┬á┬▒┬á0.4┬á┬▒┬á0.5	5.435┬á┬▒┬á1.9┬á┬▒┬á0.4┬á┬▒┬á0.6	5.435┬á┬▒┬á1.4┬á┬▒┬á0.4┬á┬▒┬á0.5
0.057ΓÇô0.064	4.992┬á┬▒┬á2.1┬á┬▒┬á0.6┬á┬▒┬á0.6	4.984┬á┬▒┬á2.1┬á┬▒┬á0.6┬á┬▒┬á0.6	5.037┬á┬▒┬á1.8┬á┬▒┬á0.4┬á┬▒┬á0.6	5.028┬á┬▒┬á1.8┬á┬▒┬á0.4┬á┬▒┬á0.6	5.029┬á┬▒┬á1.8┬á┬▒┬á0.4┬á┬▒┬á0.7	5.014┬á┬▒┬á1.4┬á┬▒┬á0.4┬á┬▒┬á0.6
0.064ΓÇô0.072	4.404┬á┬▒┬á2.1┬á┬▒┬á0.8┬á┬▒┬á0.6	4.379┬á┬▒┬á2.1┬á┬▒┬á0.9┬á┬▒┬á0.6	4.420┬á┬▒┬á1.9┬á┬▒┬á0.4┬á┬▒┬á0.6	4.426┬á┬▒┬á1.9┬á┬▒┬á0.4┬á┬▒┬á0.6	4.415┬á┬▒┬á1.9┬á┬▒┬á0.4┬á┬▒┬á0.7	4.400┬á┬▒┬á1.4┬á┬▒┬á0.4┬á┬▒┬á0.6
0.072ΓÇô0.081	4.017┬á┬▒┬á2.1┬á┬▒┬á0.6┬á┬▒┬á0.6	4.013┬á┬▒┬á2.1┬á┬▒┬á0.7┬á┬▒┬á0.6	3.839┬á┬▒┬á1.8┬á┬▒┬á0.4┬á┬▒┬á0.6	3.834┬á┬▒┬á1.8┬á┬▒┬á0.4┬á┬▒┬á0.6	3.808┬á┬▒┬á1.8┬á┬▒┬á0.4┬á┬▒┬á0.7	3.892┬á┬▒┬á1.4┬á┬▒┬á0.4┬á┬▒┬á0.6
0.081ΓÇô0.091	3.397┬á┬▒┬á2.2┬á┬▒┬á0.6┬á┬▒┬á0.6	3.392┬á┬▒┬á2.2┬á┬▒┬á0.7┬á┬▒┬á0.6	3.324┬á┬▒┬á1.8┬á┬▒┬á0.5┬á┬▒┬á0.8	3.311┬á┬▒┬á1.8┬á┬▒┬á0.5┬á┬▒┬á0.8	3.318┬á┬▒┬á1.8┬á┬▒┬á0.5┬á┬▒┬á0.9	3.356┬á┬▒┬á1.4┬á┬▒┬á0.4┬á┬▒┬á0.6
0.091ΓÇô0.102	3.067┬á┬▒┬á2.2┬á┬▒┬á0.7┬á┬▒┬á1.2	3.066┬á┬▒┬á2.2┬á┬▒┬á0.7┬á┬▒┬á1.2	3.023┬á┬▒┬á1.9┬á┬▒┬á0.5┬á┬▒┬á0.6	3.031┬á┬▒┬á1.9┬á┬▒┬á0.5┬á┬▒┬á0.6	3.004┬á┬▒┬á1.9┬á┬▒┬á0.5┬á┬▒┬á0.7	3.019┬á┬▒┬á1.4┬á┬▒┬á0.4┬á┬▒┬á0.7
0.102ΓÇô0.114	2.480┬á┬▒┬á2.3┬á┬▒┬á1.1┬á┬▒┬á1.7	2.470┬á┬▒┬á2.3┬á┬▒┬á1.1┬á┬▒┬á1.7	2.617┬á┬▒┬á1.9┬á┬▒┬á0.4┬á┬▒┬á0.6	2.620┬á┬▒┬á1.9┬á┬▒┬á0.4┬á┬▒┬á0.6	2.617┬á┬▒┬á1.9┬á┬▒┬á0.4┬á┬▒┬á0.7	2.568┬á┬▒┬á1.5┬á┬▒┬á0.5┬á┬▒┬á0.7
0.114ΓÇô0.128	2.131┬á┬▒┬á2.3┬á┬▒┬á0.7┬á┬▒┬á0.6	2.124┬á┬▒┬á2.3┬á┬▒┬á0.7┬á┬▒┬á0.6	2.165┬á┬▒┬á1.9┬á┬▒┬á0.6┬á┬▒┬á0.9	2.169┬á┬▒┬á1.9┬á┬▒┬á0.6┬á┬▒┬á0.9	2.156┬á┬▒┬á1.9┬á┬▒┬á0.6┬á┬▒┬á1.0	2.147┬á┬▒┬á1.5┬á┬▒┬á0.4┬á┬▒┬á0.6
0.128ΓÇô0.145	1.859┬á┬▒┬á2.3┬á┬▒┬á0.8┬á┬▒┬á0.5	1.852┬á┬▒┬á2.3┬á┬▒┬á0.8┬á┬▒┬á0.6	1.821┬á┬▒┬á2.0┬á┬▒┬á0.5┬á┬▒┬á0.7	1.828┬á┬▒┬á2.0┬á┬▒┬á0.5┬á┬▒┬á0.7	1.831┬á┬▒┬á2.0┬á┬▒┬á0.5┬á┬▒┬á0.8	1.840┬á┬▒┬á1.5┬á┬▒┬á0.4┬á┬▒┬á0.6
0.145ΓÇô0.165	1.530┬á┬▒┬á2.3┬á┬▒┬á0.7┬á┬▒┬á0.6	1.524┬á┬▒┬á2.3┬á┬▒┬á0.7┬á┬▒┬á0.6	1.572┬á┬▒┬á2.0┬á┬▒┬á0.5┬á┬▒┬á0.7	1.577┬á┬▒┬á2.0┬á┬▒┬á0.5┬á┬▒┬á0.7	1.572┬á┬▒┬á2.0┬á┬▒┬á0.5┬á┬▒┬á0.8	1.552┬á┬▒┬á1.5┬á┬▒┬á0.4┬á┬▒┬á0.5
0.165ΓÇô0.189	1.219┬á┬▒┬á2.4┬á┬▒┬á0.9┬á┬▒┬á0.8	1.213┬á┬▒┬á2.4┬á┬▒┬á0.9┬á┬▒┬á0.8	1.219┬á┬▒┬á2.0┬á┬▒┬á0.4┬á┬▒┬á1.1	1.224┬á┬▒┬á2.0┬á┬▒┬á0.4┬á┬▒┬á1.1	1.212┬á┬▒┬á2.0┬á┬▒┬á0.4┬á┬▒┬á1.2	1.215┬á┬▒┬á1.6┬á┬▒┬á0.4┬á┬▒┬á0.7
0.189ΓÇô0.219	0.966┬á┬▒┬á2.4┬á┬▒┬á1.1┬á┬▒┬á1.2	0.965┬á┬▒┬á2.4┬á┬▒┬á1.1┬á┬▒┬á1.2	0.948┬á┬▒┬á2.1┬á┬▒┬á0.4┬á┬▒┬á1.7	0.948┬á┬▒┬á2.1┬á┬▒┬á0.4┬á┬▒┬á1.7	0.949┬á┬▒┬á2.1┬á┬▒┬á0.4┬á┬▒┬á1.7	0.961┬á┬▒┬á1.6┬á┬▒┬á0.5┬á┬▒┬á0.8
0.219ΓÇô0.258	0.751┬á┬▒┬á2.5┬á┬▒┬á1.0┬á┬▒┬á0.8	0.749┬á┬▒┬á2.5┬á┬▒┬á1.1┬á┬▒┬á0.8	0.767┬á┬▒┬á2.1┬á┬▒┬á0.5┬á┬▒┬á1.8	0.768┬á┬▒┬á2.1┬á┬▒┬á0.5┬á┬▒┬á1.8	0.764┬á┬▒┬á2.1┬á┬▒┬á0.5┬á┬▒┬á1.8	0.764┬á┬▒┬á1.6┬á┬▒┬á0.5┬á┬▒┬á0.8
0.258ΓÇô0.312	0.536┬á┬▒┬á2.5┬á┬▒┬á1.3┬á┬▒┬á1.9	0.534┬á┬▒┬á2.5┬á┬▒┬á1.3┬á┬▒┬á1.9	0.538┬á┬▒┬á2.2┬á┬▒┬á0.6┬á┬▒┬á1.8	0.539┬á┬▒┬á2.2┬á┬▒┬á0.6┬á┬▒┬á1.8	0.536┬á┬▒┬á2.2┬á┬▒┬á0.6┬á┬▒┬á1.8	0.538┬á┬▒┬á1.7┬á┬▒┬á0.6┬á┬▒┬á1.1
0.312ΓÇô0.391	0.356┬á┬▒┬á2.6┬á┬▒┬á1.1┬á┬▒┬á1.8	0.355┬á┬▒┬á2.6┬á┬▒┬á1.1┬á┬▒┬á1.8	0.335┬á┬▒┬á2.4┬á┬▒┬á0.7┬á┬▒┬á2.3	0.337┬á┬▒┬á2.4┬á┬▒┬á0.7┬á┬▒┬á2.3	0.336┬á┬▒┬á2.4┬á┬▒┬á0.7┬á┬▒┬á2.3	0.346┬á┬▒┬á1.8┬á┬▒┬á0.6┬á┬▒┬á1.3
0.391ΓÇô0.524	0.204┬á┬▒┬á2.8┬á┬▒┬á1.3┬á┬▒┬á2.0	0.203┬á┬▒┬á2.8┬á┬▒┬á1.3┬á┬▒┬á2.0	0.192┬á┬▒┬á2.5┬á┬▒┬á0.8┬á┬▒┬á2.8	0.193┬á┬▒┬á2.5┬á┬▒┬á0.8┬á┬▒┬á2.8	0.192┬á┬▒┬á2.5┬á┬▒┬á0.8┬á┬▒┬á2.8	0.198┬á┬▒┬á1.9┬á┬▒┬á0.7┬á┬▒┬á1.7
0.524ΓÇô0.695	0.0988┬á┬▒┬á3.7┬á┬▒┬á1.9┬á┬▒┬á3.1	0.0985┬á┬▒┬á3.7┬á┬▒┬á1.9┬á┬▒┬á3.1	0.0935┬á┬▒┬á3.3┬á┬▒┬á2.0┬á┬▒┬á2.5	0.0940┬á┬▒┬á3.3┬á┬▒┬á2.0┬á┬▒┬á2.5	0.0936┬á┬▒┬á3.3┬á┬▒┬á2.0┬á┬▒┬á2.5	0.0959┬á┬▒┬á2.5┬á┬▒┬á1.4┬á┬▒┬á2.2
0.695ΓÇô0.918	0.0515┬á┬▒┬á4.4┬á┬▒┬á2.0┬á┬▒┬á3.0	0.0515┬á┬▒┬á4.4┬á┬▒┬á2.0┬á┬▒┬á3.0	0.0493┬á┬▒┬á4.1┬á┬▒┬á2.1┬á┬▒┬á3.6	0.0496┬á┬▒┬á4.1┬á┬▒┬á2.1┬á┬▒┬á3.6	0.0494┬á┬▒┬á4.1┬á┬▒┬á2.1┬á┬▒┬á3.6	0.0511┬á┬▒┬á3.0┬á┬▒┬á1.5┬á┬▒┬á2.4
0.918ΓÇô1.153	0.0252┬á┬▒┬á6.0┬á┬▒┬á3.5┬á┬▒┬á2.8	0.0251┬á┬▒┬á6.0┬á┬▒┬á3.5┬á┬▒┬á2.9	0.0255┬á┬▒┬á5.3┬á┬▒┬á2.2┬á┬▒┬á2.4	0.0259┬á┬▒┬á5.3┬á┬▒┬á2.2┬á┬▒┬á2.4	0.0259┬á┬▒┬á5.3┬á┬▒┬á2.2┬á┬▒┬á2.5	0.0255┬á┬▒┬á4.0┬á┬▒┬á1.9┬á┬▒┬á2.0
1.153ΓÇô1.496	0.0144┬á┬▒┬á6.4┬á┬▒┬á4.1┬á┬▒┬á7.8	0.0145┬á┬▒┬á6.4┬á┬▒┬á4.2┬á┬▒┬á7.7	0.0122┬á┬▒┬á6.4┬á┬▒┬á1.6┬á┬▒┬á3.5	0.0123┬á┬▒┬á6.4┬á┬▒┬á1.6┬á┬▒┬á3.5	0.0124┬á┬▒┬á6.4┬á┬▒┬á1.6┬á┬▒┬á3.6	0.0136┬á┬▒┬á4.5┬á┬▒┬á1.9┬á┬▒┬á3.0
1.496ΓÇô1.947	0.00651┬á┬▒┬á8.3┬á┬▒┬á3.3┬á┬▒┬á2.9	0.00649┬á┬▒┬á8.3┬á┬▒┬á3.4┬á┬▒┬á2.9	0.00525┬á┬▒┬á8.5┬á┬▒┬á2.3┬á┬▒┬á5.5	0.00532┬á┬▒┬á8.5┬á┬▒┬á2.3┬á┬▒┬á5.5	0.00527┬á┬▒┬á8.5┬á┬▒┬á2.3┬á┬▒┬á5.6	0.00594┬á┬▒┬á5.9┬á┬▒┬á2.0┬á┬▒┬á2.7
1.947ΓÇô2.522	0.00250┬á┬▒┬á13┬á┬▒┬á7.9┬á┬▒┬á6.5	0.00248┬á┬▒┬á13┬á┬▒┬á7.9┬á┬▒┬á6.6	0.00313┬á┬▒┬á9.9┬á┬▒┬á2.1┬á┬▒┬á4.3	0.00318┬á┬▒┬á9.9┬á┬▒┬á2.1┬á┬▒┬á4.3	0.00317┬á┬▒┬á9.9┬á┬▒┬á2.1┬á┬▒┬á4.4	0.00299┬á┬▒┬á7.8┬á┬▒┬á3.1┬á┬▒┬á3.0
2.522ΓÇô3.277	0.00132┬á┬▒┬á15┬á┬▒┬á6.7┬á┬▒┬á7.2	0.00133┬á┬▒┬á15┬á┬▒┬á6.7┬á┬▒┬á7.2	0.00170┬á┬▒┬á12┬á┬▒┬á4.6┬á┬▒┬á4.3	0.00174┬á┬▒┬á12┬á┬▒┬á4.6┬á┬▒┬á4.3	0.00174┬á┬▒┬á12┬á┬▒┬á4.6┬á┬▒┬á4.4	0.00162┬á┬▒┬á9.1┬á┬▒┬á3.9┬á┬▒┬á3.4
3.277ΓÇô5.000	0.000668┬á┬▒┬á14┬á┬▒┬á7.3┬á┬▒┬á5.2	0.000662┬á┬▒┬á14┬á┬▒┬á7.3┬á┬▒┬á5.2	0.000466┬á┬▒┬á16┬á┬▒┬á5.3┬á┬▒┬á9.3	0.000476┬á┬▒┬á16┬á┬▒┬á5.3┬á┬▒┬á9.3	0.000472┬á┬▒┬á16┬á┬▒┬á5.3┬á┬▒┬á9.3	0.000587┬á┬▒┬á11┬á┬▒┬á4.4┬á┬▒┬á4.3
5.000ΓÇô10.000	0.000229┬á┬▒┬á15┬á┬▒┬á6.2┬á┬▒┬á4.7	0.000226┬á┬▒┬á15┬á┬▒┬á6.3┬á┬▒┬á4.6	0.000177┬á┬▒┬á15┬á┬▒┬á4.4┬á┬▒┬á6.8	0.000179┬á┬▒┬á15┬á┬▒┬á4.4┬á┬▒┬á6.8	0.000181┬á┬▒┬á15┬á┬▒┬á4.4┬á┬▒┬á6.8	0.000206┬á┬▒┬á10┬á┬▒┬á3.7┬á┬▒┬á3.6

**Table 20:** The values of $(1/\sigma )\,d\sigma /dp_\mathrm {T}^{\ell \ell }$ in each bin of $p_\mathrm {T}^{\ell \ell }$ for the electron and muon channels separately (for various generator-level definitions) and for the Born-level combination in the kinematic region $66 \text { GeV} \le m_{\ell \ell }< 116 \text { GeV},\ 0.0 \le |y_{\ell \ell }| < 0.4$. The associated statistical and systematic (both uncorrelated and correlated between bins) are provided in percentage form

Bin	$(1/\sigma )\,d\sigma /dp_\mathrm {T}^{\ell \ell }$ ┬▒ Statistical [%] ┬▒ Uncorrelated systematic [%] ┬▒ Correlated systematic [%]					
	Electron channel		Muon channel			Combination
	Dressed	Born	Bare	Dressed	Born	Born
0.0ΓÇô2.0	2.622eΓêÆ02┬á┬▒┬á0.5┬á┬▒┬á0.2┬á┬▒┬á0.8	2.688eΓêÆ02┬á┬▒┬á0.5┬á┬▒┬á0.2┬á┬▒┬á0.8	2.586eΓêÆ02┬á┬▒┬á0.4┬á┬▒┬á0.2┬á┬▒┬á0.9	2.638eΓêÆ02┬á┬▒┬á0.4┬á┬▒┬á0.2┬á┬▒┬á0.9	2.704eΓêÆ02┬á┬▒┬á0.4┬á┬▒┬á0.2┬á┬▒┬á1.0	2.704eΓêÆ02┬á┬▒┬á0.3┬á┬▒┬á0.2┬á┬▒┬á0.7
2.0ΓÇô4.0	5.438eΓêÆ02┬á┬▒┬á0.3┬á┬▒┬á0.1┬á┬▒┬á0.4	5.544eΓêÆ02┬á┬▒┬á0.3┬á┬▒┬á0.1┬á┬▒┬á0.4	5.385eΓêÆ02┬á┬▒┬á0.3┬á┬▒┬á0.1┬á┬▒┬á0.4	5.472eΓêÆ02┬á┬▒┬á0.3┬á┬▒┬á0.1┬á┬▒┬á0.4	5.582eΓêÆ02┬á┬▒┬á0.3┬á┬▒┬á0.1┬á┬▒┬á0.6	5.569eΓêÆ02┬á┬▒┬á0.2┬á┬▒┬á0.1┬á┬▒┬á0.3
4.0ΓÇô6.0	5.513eΓêÆ02┬á┬▒┬á0.3┬á┬▒┬á0.2┬á┬▒┬á0.4	5.562eΓêÆ02┬á┬▒┬á0.3┬á┬▒┬á0.2┬á┬▒┬á0.4	5.502eΓêÆ02┬á┬▒┬á0.3┬á┬▒┬á0.2┬á┬▒┬á0.5	5.534eΓêÆ02┬á┬▒┬á0.3┬á┬▒┬á0.2┬á┬▒┬á0.5	5.584eΓêÆ02┬á┬▒┬á0.3┬á┬▒┬á0.2┬á┬▒┬á0.6	5.571eΓêÆ02┬á┬▒┬á0.2┬á┬▒┬á0.1┬á┬▒┬á0.4
6.0ΓÇô8.0	4.896eΓêÆ02┬á┬▒┬á0.4┬á┬▒┬á0.2┬á┬▒┬á0.3	4.897eΓêÆ02┬á┬▒┬á0.4┬á┬▒┬á0.2┬á┬▒┬á0.3	4.887eΓêÆ02┬á┬▒┬á0.3┬á┬▒┬á0.2┬á┬▒┬á0.3	4.879eΓêÆ02┬á┬▒┬á0.3┬á┬▒┬á0.2┬á┬▒┬á0.3	4.875eΓêÆ02┬á┬▒┬á0.3┬á┬▒┬á0.2┬á┬▒┬á0.6	4.883eΓêÆ02┬á┬▒┬á0.2┬á┬▒┬á0.1┬á┬▒┬á0.3
8.0ΓÇô10.0	4.127eΓêÆ02┬á┬▒┬á0.4┬á┬▒┬á0.2┬á┬▒┬á0.4	4.096eΓêÆ02┬á┬▒┬á0.4┬á┬▒┬á0.2┬á┬▒┬á0.6	4.139eΓêÆ02┬á┬▒┬á0.4┬á┬▒┬á0.2┬á┬▒┬á0.4	4.111eΓêÆ02┬á┬▒┬á0.4┬á┬▒┬á0.2┬á┬▒┬á0.4	4.082eΓêÆ02┬á┬▒┬á0.4┬á┬▒┬á0.2┬á┬▒┬á0.7	4.086eΓêÆ02┬á┬▒┬á0.3┬á┬▒┬á0.1┬á┬▒┬á0.4
10.0ΓÇô13.0	3.293eΓêÆ02┬á┬▒┬á0.3┬á┬▒┬á0.2┬á┬▒┬á0.3	3.256eΓêÆ02┬á┬▒┬á0.3┬á┬▒┬á0.2┬á┬▒┬á0.3	3.324eΓêÆ02┬á┬▒┬á0.3┬á┬▒┬á0.1┬á┬▒┬á0.3	3.287eΓêÆ02┬á┬▒┬á0.3┬á┬▒┬á0.1┬á┬▒┬á0.3	3.247eΓêÆ02┬á┬▒┬á0.3┬á┬▒┬á0.1┬á┬▒┬á0.4	3.251eΓêÆ02┬á┬▒┬á0.2┬á┬▒┬á0.1┬á┬▒┬á0.2
13.0ΓÇô16.0	2.532eΓêÆ02┬á┬▒┬á0.4┬á┬▒┬á0.2┬á┬▒┬á0.3	2.495eΓêÆ02┬á┬▒┬á0.4┬á┬▒┬á0.2┬á┬▒┬á0.3	2.587eΓêÆ02┬á┬▒┬á0.3┬á┬▒┬á0.1┬á┬▒┬á0.3	2.551eΓêÆ02┬á┬▒┬á0.3┬á┬▒┬á0.1┬á┬▒┬á0.3	2.513eΓêÆ02┬á┬▒┬á0.3┬á┬▒┬á0.1┬á┬▒┬á0.4	2.505eΓêÆ02┬á┬▒┬á0.3┬á┬▒┬á0.1┬á┬▒┬á0.2
16.0ΓÇô20.0	1.920eΓêÆ02┬á┬▒┬á0.4┬á┬▒┬á0.2┬á┬▒┬á0.3	1.891eΓêÆ02┬á┬▒┬á0.4┬á┬▒┬á0.2┬á┬▒┬á0.3	1.943eΓêÆ02┬á┬▒┬á0.3┬á┬▒┬á0.1┬á┬▒┬á0.3	1.920eΓêÆ02┬á┬▒┬á0.3┬á┬▒┬á0.1┬á┬▒┬á0.3	1.892eΓêÆ02┬á┬▒┬á0.3┬á┬▒┬á0.1┬á┬▒┬á0.3	1.892eΓêÆ02┬á┬▒┬á0.2┬á┬▒┬á0.1┬á┬▒┬á0.2
20.0ΓÇô25.0	1.377eΓêÆ02┬á┬▒┬á0.4┬á┬▒┬á0.1┬á┬▒┬á0.2	1.363eΓêÆ02┬á┬▒┬á0.4┬á┬▒┬á0.1┬á┬▒┬á0.2	1.391eΓêÆ02┬á┬▒┬á0.3┬á┬▒┬á0.1┬á┬▒┬á0.3	1.380eΓêÆ02┬á┬▒┬á0.3┬á┬▒┬á0.1┬á┬▒┬á0.3	1.366eΓêÆ02┬á┬▒┬á0.3┬á┬▒┬á0.1┬á┬▒┬á0.3	1.365eΓêÆ02┬á┬▒┬á0.2┬á┬▒┬á0.1┬á┬▒┬á0.2
25.0ΓÇô30.0	9.812eΓêÆ03┬á┬▒┬á0.4┬á┬▒┬á0.1┬á┬▒┬á0.3	9.758eΓêÆ03┬á┬▒┬á0.4┬á┬▒┬á0.1┬á┬▒┬á0.3	9.802eΓêÆ03┬á┬▒┬á0.4┬á┬▒┬á0.2┬á┬▒┬á0.3	9.772eΓêÆ03┬á┬▒┬á0.4┬á┬▒┬á0.2┬á┬▒┬á0.3	9.707eΓêÆ03┬á┬▒┬á0.4┬á┬▒┬á0.2┬á┬▒┬á0.3	9.728eΓêÆ03┬á┬▒┬á0.3┬á┬▒┬á0.1┬á┬▒┬á0.2
30.0ΓÇô37.0	6.865eΓêÆ03┬á┬▒┬á0.4┬á┬▒┬á0.2┬á┬▒┬á0.3	6.850eΓêÆ03┬á┬▒┬á0.4┬á┬▒┬á0.2┬á┬▒┬á0.3	6.843eΓêÆ03┬á┬▒┬á0.4┬á┬▒┬á0.1┬á┬▒┬á0.3	6.845eΓêÆ03┬á┬▒┬á0.4┬á┬▒┬á0.1┬á┬▒┬á0.3	6.835eΓêÆ03┬á┬▒┬á0.4┬á┬▒┬á0.1┬á┬▒┬á0.3	6.840eΓêÆ03┬á┬▒┬á0.3┬á┬▒┬á0.1┬á┬▒┬á0.2
37.0ΓÇô45.0	4.627eΓêÆ03┬á┬▒┬á0.5┬á┬▒┬á0.2┬á┬▒┬á0.5	4.634eΓêÆ03┬á┬▒┬á0.5┬á┬▒┬á0.2┬á┬▒┬á0.5	4.568eΓêÆ03┬á┬▒┬á0.4┬á┬▒┬á0.1┬á┬▒┬á0.3	4.580eΓêÆ03┬á┬▒┬á0.4┬á┬▒┬á0.1┬á┬▒┬á0.3	4.591eΓêÆ03┬á┬▒┬á0.4┬á┬▒┬á0.1┬á┬▒┬á0.3	4.609eΓêÆ03┬á┬▒┬á0.3┬á┬▒┬á0.1┬á┬▒┬á0.2
45.0ΓÇô55.0	3.029eΓêÆ03┬á┬▒┬á0.5┬á┬▒┬á0.2┬á┬▒┬á0.7	3.042eΓêÆ03┬á┬▒┬á0.5┬á┬▒┬á0.2┬á┬▒┬á0.7	2.998eΓêÆ03┬á┬▒┬á0.5┬á┬▒┬á0.2┬á┬▒┬á0.4	3.018eΓêÆ03┬á┬▒┬á0.5┬á┬▒┬á0.2┬á┬▒┬á0.4	3.033eΓêÆ03┬á┬▒┬á0.5┬á┬▒┬á0.2┬á┬▒┬á0.4	3.033eΓêÆ03┬á┬▒┬á0.3┬á┬▒┬á0.1┬á┬▒┬á0.3
55.0ΓÇô65.0	1.926eΓêÆ03┬á┬▒┬á0.6┬á┬▒┬á0.2┬á┬▒┬á0.8	1.939eΓêÆ03┬á┬▒┬á0.6┬á┬▒┬á0.2┬á┬▒┬á0.8	1.900eΓêÆ03┬á┬▒┬á0.6┬á┬▒┬á0.3┬á┬▒┬á0.4	1.917eΓêÆ03┬á┬▒┬á0.6┬á┬▒┬á0.3┬á┬▒┬á0.4	1.931eΓêÆ03┬á┬▒┬á0.6┬á┬▒┬á0.3┬á┬▒┬á0.5	1.932eΓêÆ03┬á┬▒┬á0.4┬á┬▒┬á0.2┬á┬▒┬á0.4
65.0ΓÇô75.0	1.275eΓêÆ03┬á┬▒┬á0.8┬á┬▒┬á0.3┬á┬▒┬á0.9	1.286eΓêÆ03┬á┬▒┬á0.8┬á┬▒┬á0.3┬á┬▒┬á0.9	1.266eΓêÆ03┬á┬▒┬á0.8┬á┬▒┬á0.3┬á┬▒┬á0.5	1.282eΓêÆ03┬á┬▒┬á0.8┬á┬▒┬á0.3┬á┬▒┬á0.5	1.291eΓêÆ03┬á┬▒┬á0.8┬á┬▒┬á0.3┬á┬▒┬á0.6	1.287eΓêÆ03┬á┬▒┬á0.5┬á┬▒┬á0.2┬á┬▒┬á0.4
75.0ΓÇô85.0	8.504eΓêÆ04┬á┬▒┬á0.9┬á┬▒┬á0.4┬á┬▒┬á0.9	8.597eΓêÆ04┬á┬▒┬á0.9┬á┬▒┬á0.4┬á┬▒┬á0.9	8.383eΓêÆ04┬á┬▒┬á1.0┬á┬▒┬á0.4┬á┬▒┬á0.6	8.486eΓêÆ04┬á┬▒┬á1.0┬á┬▒┬á0.4┬á┬▒┬á0.6	8.565eΓêÆ04┬á┬▒┬á1.0┬á┬▒┬á0.4┬á┬▒┬á0.6	8.569eΓêÆ04┬á┬▒┬á0.7┬á┬▒┬á0.3┬á┬▒┬á0.5
85.0ΓÇô105.0	4.956eΓêÆ04┬á┬▒┬á0.8┬á┬▒┬á0.3┬á┬▒┬á0.8	4.995eΓêÆ04┬á┬▒┬á0.8┬á┬▒┬á0.3┬á┬▒┬á0.8	4.774eΓêÆ04┬á┬▒┬á0.8┬á┬▒┬á0.3┬á┬▒┬á0.5	4.833eΓêÆ04┬á┬▒┬á0.8┬á┬▒┬á0.3┬á┬▒┬á0.5	4.868eΓêÆ04┬á┬▒┬á0.8┬á┬▒┬á0.3┬á┬▒┬á0.5	4.927eΓêÆ04┬á┬▒┬á0.6┬á┬▒┬á0.2┬á┬▒┬á0.4
105.0ΓÇô150.0	1.896eΓêÆ04┬á┬▒┬á0.9┬á┬▒┬á0.3┬á┬▒┬á0.7	1.904eΓêÆ04┬á┬▒┬á0.9┬á┬▒┬á0.3┬á┬▒┬á0.7	1.826eΓêÆ04┬á┬▒┬á0.9┬á┬▒┬á0.3┬á┬▒┬á0.5	1.852eΓêÆ04┬á┬▒┬á0.9┬á┬▒┬á0.3┬á┬▒┬á0.5	1.865eΓêÆ04┬á┬▒┬á0.9┬á┬▒┬á0.3┬á┬▒┬á0.5	1.884eΓêÆ04┬á┬▒┬á0.6┬á┬▒┬á0.2┬á┬▒┬á0.4
150.0ΓÇô200.0	5.379eΓêÆ05┬á┬▒┬á1.4┬á┬▒┬á0.5┬á┬▒┬á0.9	5.409eΓêÆ05┬á┬▒┬á1.4┬á┬▒┬á0.5┬á┬▒┬á0.9	5.278eΓêÆ05┬á┬▒┬á1.6┬á┬▒┬á0.5┬á┬▒┬á0.7	5.377eΓêÆ05┬á┬▒┬á1.6┬á┬▒┬á0.5┬á┬▒┬á0.7	5.402eΓêÆ05┬á┬▒┬á1.6┬á┬▒┬á0.5┬á┬▒┬á0.7	5.399eΓêÆ05┬á┬▒┬á1.1┬á┬▒┬á0.4┬á┬▒┬á0.5
200.0ΓÇô900.0	2.288eΓêÆ06┬á┬▒┬á1.8┬á┬▒┬á0.6┬á┬▒┬á1.4	2.296eΓêÆ06┬á┬▒┬á1.8┬á┬▒┬á0.6┬á┬▒┬á1.5	2.220eΓêÆ06┬á┬▒┬á2.1┬á┬▒┬á0.5┬á┬▒┬á0.9	2.277eΓêÆ06┬á┬▒┬á2.1┬á┬▒┬á0.5┬á┬▒┬á0.9	2.287eΓêÆ06┬á┬▒┬á2.1┬á┬▒┬á0.5┬á┬▒┬á1.0	2.284eΓêÆ06┬á┬▒┬á1.4┬á┬▒┬á0.4┬á┬▒┬á0.8

**Table 21:** The values of $(1/\sigma )\,d\sigma /dp_\mathrm {T}^{\ell \ell }$ in each bin of $p_\mathrm {T}^{\ell \ell }$ for the electron and muon channels separately (for various generator-level definitions) and for the Born-level combination in the kinematic region $66~\text {GeV} \le m_{\ell \ell }< 116~\text {GeV},\ 0.4 \le |y_{\ell \ell }| < 0.8$. The associated statistical and systematic (both uncorrelated and correlated between bins) are provided in percentage form

Bin	$(1/\sigma )\,d\sigma /dp_\mathrm {T}^{\ell \ell }$ ┬▒ Statistical [%] ┬▒ Uncorrelated systematic [%] ┬▒ Correlated systematic [%]					
	Electron channel		Muon channel			Combination
	Dressed	Born	Bare	Dressed	Born	Born
0.0ΓÇô2.0	2.601eΓêÆ02┬á┬▒┬á0.6┬á┬▒┬á0.3┬á┬▒┬á1.2	2.663eΓêÆ02┬á┬▒┬á0.6┬á┬▒┬á0.3┬á┬▒┬á1.2	2.620eΓêÆ02┬á┬▒┬á0.4┬á┬▒┬á0.2┬á┬▒┬á0.9	2.672eΓêÆ02┬á┬▒┬á0.4┬á┬▒┬á0.2┬á┬▒┬á0.9	2.739eΓêÆ02┬á┬▒┬á0.4┬á┬▒┬á0.2┬á┬▒┬á0.9	2.690eΓêÆ02┬á┬▒┬á0.3┬á┬▒┬á0.2┬á┬▒┬á0.8
2.0ΓÇô4.0	5.469eΓêÆ02┬á┬▒┬á0.4┬á┬▒┬á0.2┬á┬▒┬á0.6	5.577eΓêÆ02┬á┬▒┬á0.4┬á┬▒┬á0.2┬á┬▒┬á0.6	5.405eΓêÆ02┬á┬▒┬á0.3┬á┬▒┬á0.1┬á┬▒┬á0.3	5.485eΓêÆ02┬á┬▒┬á0.3┬á┬▒┬á0.1┬á┬▒┬á0.3	5.590eΓêÆ02┬á┬▒┬á0.3┬á┬▒┬á0.1┬á┬▒┬á0.4	5.575eΓêÆ02┬á┬▒┬á0.2┬á┬▒┬á0.1┬á┬▒┬á0.3
4.0ΓÇô6.0	5.582eΓêÆ02┬á┬▒┬á0.4┬á┬▒┬á0.1┬á┬▒┬á0.4	5.634eΓêÆ02┬á┬▒┬á0.4┬á┬▒┬á0.1┬á┬▒┬á0.4	5.530eΓêÆ02┬á┬▒┬á0.3┬á┬▒┬á0.1┬á┬▒┬á0.4	5.569eΓêÆ02┬á┬▒┬á0.3┬á┬▒┬á0.1┬á┬▒┬á0.4	5.617eΓêÆ02┬á┬▒┬á0.3┬á┬▒┬á0.1┬á┬▒┬á0.5	5.642eΓêÆ02┬á┬▒┬á0.2┬á┬▒┬á0.1┬á┬▒┬á0.3
6.0ΓÇô8.0	4.875eΓêÆ02┬á┬▒┬á0.4┬á┬▒┬á0.2┬á┬▒┬á0.5	4.874eΓêÆ02┬á┬▒┬á0.4┬á┬▒┬á0.2┬á┬▒┬á0.6	4.941eΓêÆ02┬á┬▒┬á0.3┬á┬▒┬á0.2┬á┬▒┬á0.2	4.936eΓêÆ02┬á┬▒┬á0.3┬á┬▒┬á0.2┬á┬▒┬á0.2	4.933eΓêÆ02┬á┬▒┬á0.3┬á┬▒┬á0.2┬á┬▒┬á0.5	4.922eΓêÆ02┬á┬▒┬á0.3┬á┬▒┬á0.1┬á┬▒┬á0.4
8.0ΓÇô10.0	4.085eΓêÆ02┬á┬▒┬á0.4┬á┬▒┬á0.2┬á┬▒┬á0.5	4.054eΓêÆ02┬á┬▒┬á0.4┬á┬▒┬á0.2┬á┬▒┬á0.5	4.143eΓêÆ02┬á┬▒┬á0.4┬á┬▒┬á0.2┬á┬▒┬á0.3	4.112eΓêÆ02┬á┬▒┬á0.4┬á┬▒┬á0.2┬á┬▒┬á0.3	4.085eΓêÆ02┬á┬▒┬á0.4┬á┬▒┬á0.2┬á┬▒┬á0.5	4.071eΓêÆ02┬á┬▒┬á0.3┬á┬▒┬á0.1┬á┬▒┬á0.4
10.0ΓÇô13.0	3.303eΓêÆ02┬á┬▒┬á0.4┬á┬▒┬á0.2┬á┬▒┬á0.3	3.265eΓêÆ02┬á┬▒┬á0.4┬á┬▒┬á0.2┬á┬▒┬á0.3	3.325eΓêÆ02┬á┬▒┬á0.3┬á┬▒┬á0.1┬á┬▒┬á0.2	3.290eΓêÆ02┬á┬▒┬á0.3┬á┬▒┬á0.1┬á┬▒┬á0.2	3.251eΓêÆ02┬á┬▒┬á0.3┬á┬▒┬á0.1┬á┬▒┬á0.3	3.256eΓêÆ02┬á┬▒┬á0.2┬á┬▒┬á0.1┬á┬▒┬á0.2
13.0ΓÇô16.0	2.548eΓêÆ02┬á┬▒┬á0.4┬á┬▒┬á0.2┬á┬▒┬á0.3	2.511eΓêÆ02┬á┬▒┬á0.4┬á┬▒┬á0.2┬á┬▒┬á0.3	2.569eΓêÆ02┬á┬▒┬á0.3┬á┬▒┬á0.1┬á┬▒┬á0.2	2.533eΓêÆ02┬á┬▒┬á0.3┬á┬▒┬á0.1┬á┬▒┬á0.2	2.498eΓêÆ02┬á┬▒┬á0.3┬á┬▒┬á0.1┬á┬▒┬á0.2	2.501eΓêÆ02┬á┬▒┬á0.3┬á┬▒┬á0.1┬á┬▒┬á0.2
16.0ΓÇô20.0	1.933eΓêÆ02┬á┬▒┬á0.4┬á┬▒┬á0.2┬á┬▒┬á0.3	1.905eΓêÆ02┬á┬▒┬á0.4┬á┬▒┬á0.2┬á┬▒┬á0.4	1.940eΓêÆ02┬á┬▒┬á0.3┬á┬▒┬á0.1┬á┬▒┬á0.2	1.916eΓêÆ02┬á┬▒┬á0.3┬á┬▒┬á0.1┬á┬▒┬á0.2	1.887eΓêÆ02┬á┬▒┬á0.3┬á┬▒┬á0.1┬á┬▒┬á0.3	1.894eΓêÆ02┬á┬▒┬á0.3┬á┬▒┬á0.1┬á┬▒┬á0.2
20.0ΓÇô25.0	1.380eΓêÆ02┬á┬▒┬á0.4┬á┬▒┬á0.2┬á┬▒┬á0.3	1.365eΓêÆ02┬á┬▒┬á0.4┬á┬▒┬á0.2┬á┬▒┬á0.3	1.379eΓêÆ02┬á┬▒┬á0.3┬á┬▒┬á0.1┬á┬▒┬á0.2	1.368eΓêÆ02┬á┬▒┬á0.3┬á┬▒┬á0.1┬á┬▒┬á0.2	1.355eΓêÆ02┬á┬▒┬á0.3┬á┬▒┬á0.1┬á┬▒┬á0.3	1.359eΓêÆ02┬á┬▒┬á0.3┬á┬▒┬á0.1┬á┬▒┬á0.2
25.0ΓÇô30.0	9.832eΓêÆ03┬á┬▒┬á0.5┬á┬▒┬á0.2┬á┬▒┬á0.3	9.771eΓêÆ03┬á┬▒┬á0.5┬á┬▒┬á0.2┬á┬▒┬á0.3	9.800eΓêÆ03┬á┬▒┬á0.4┬á┬▒┬á0.1┬á┬▒┬á0.2	9.759eΓêÆ03┬á┬▒┬á0.4┬á┬▒┬á0.1┬á┬▒┬á0.2	9.695eΓêÆ03┬á┬▒┬á0.4┬á┬▒┬á0.1┬á┬▒┬á0.2	9.719eΓêÆ03┬á┬▒┬á0.3┬á┬▒┬á0.1┬á┬▒┬á0.2
30.0ΓÇô37.0	6.765eΓêÆ03┬á┬▒┬á0.4┬á┬▒┬á0.2┬á┬▒┬á0.3	6.759eΓêÆ03┬á┬▒┬á0.4┬á┬▒┬á0.2┬á┬▒┬á0.3	6.786eΓêÆ03┬á┬▒┬á0.4┬á┬▒┬á0.1┬á┬▒┬á0.3	6.791eΓêÆ03┬á┬▒┬á0.4┬á┬▒┬á0.1┬á┬▒┬á0.3	6.779eΓêÆ03┬á┬▒┬á0.4┬á┬▒┬á0.1┬á┬▒┬á0.3	6.763eΓêÆ03┬á┬▒┬á0.3┬á┬▒┬á0.1┬á┬▒┬á0.2
37.0ΓÇô45.0	4.625eΓêÆ03┬á┬▒┬á0.5┬á┬▒┬á0.2┬á┬▒┬á0.5	4.630eΓêÆ03┬á┬▒┬á0.5┬á┬▒┬á0.2┬á┬▒┬á0.5	4.538eΓêÆ03┬á┬▒┬á0.4┬á┬▒┬á0.2┬á┬▒┬á0.3	4.555eΓêÆ03┬á┬▒┬á0.4┬á┬▒┬á0.2┬á┬▒┬á0.3	4.564eΓêÆ03┬á┬▒┬á0.4┬á┬▒┬á0.2┬á┬▒┬á0.4	4.589eΓêÆ03┬á┬▒┬á0.3┬á┬▒┬á0.1┬á┬▒┬á0.3
45.0ΓÇô55.0	2.993eΓêÆ03┬á┬▒┬á0.5┬á┬▒┬á0.2┬á┬▒┬á0.7	3.006eΓêÆ03┬á┬▒┬á0.5┬á┬▒┬á0.2┬á┬▒┬á0.7	2.939eΓêÆ03┬á┬▒┬á0.5┬á┬▒┬á0.2┬á┬▒┬á0.4	2.955eΓêÆ03┬á┬▒┬á0.5┬á┬▒┬á0.2┬á┬▒┬á0.4	2.969eΓêÆ03┬á┬▒┬á0.5┬á┬▒┬á0.2┬á┬▒┬á0.4	2.984eΓêÆ03┬á┬▒┬á0.4┬á┬▒┬á0.1┬á┬▒┬á0.3
55.0ΓÇô65.0	1.915eΓêÆ03┬á┬▒┬á0.7┬á┬▒┬á0.3┬á┬▒┬á0.9	1.928eΓêÆ03┬á┬▒┬á0.7┬á┬▒┬á0.3┬á┬▒┬á0.9	1.894eΓêÆ03┬á┬▒┬á0.6┬á┬▒┬á0.2┬á┬▒┬á0.5	1.912eΓêÆ03┬á┬▒┬á0.6┬á┬▒┬á0.2┬á┬▒┬á0.5	1.925eΓêÆ03┬á┬▒┬á0.6┬á┬▒┬á0.2┬á┬▒┬á0.5	1.926eΓêÆ03┬á┬▒┬á0.4┬á┬▒┬á0.2┬á┬▒┬á0.4
65.0ΓÇô75.0	1.282eΓêÆ03┬á┬▒┬á0.8┬á┬▒┬á0.4┬á┬▒┬á1.0	1.294eΓêÆ03┬á┬▒┬á0.8┬á┬▒┬á0.4┬á┬▒┬á1.0	1.246eΓêÆ03┬á┬▒┬á0.8┬á┬▒┬á0.3┬á┬▒┬á0.5	1.260eΓêÆ03┬á┬▒┬á0.8┬á┬▒┬á0.3┬á┬▒┬á0.5	1.271eΓêÆ03┬á┬▒┬á0.8┬á┬▒┬á0.3┬á┬▒┬á0.5	1.283eΓêÆ03┬á┬▒┬á0.6┬á┬▒┬á0.2┬á┬▒┬á0.5
75.0ΓÇô85.0	8.543eΓêÆ04┬á┬▒┬á1.0┬á┬▒┬á0.4┬á┬▒┬á1.1	8.627eΓêÆ04┬á┬▒┬á1.0┬á┬▒┬á0.4┬á┬▒┬á1.1	8.402eΓêÆ04┬á┬▒┬á1.0┬á┬▒┬á0.4┬á┬▒┬á0.6	8.501eΓêÆ04┬á┬▒┬á1.0┬á┬▒┬á0.4┬á┬▒┬á0.6	8.586eΓêÆ04┬á┬▒┬á1.0┬á┬▒┬á0.4┬á┬▒┬á0.6	8.633eΓêÆ04┬á┬▒┬á0.7┬á┬▒┬á0.3┬á┬▒┬á0.5
85.0ΓÇô105.0	4.796eΓêÆ04┬á┬▒┬á0.9┬á┬▒┬á0.3┬á┬▒┬á1.0	4.830eΓêÆ04┬á┬▒┬á0.9┬á┬▒┬á0.3┬á┬▒┬á1.0	4.773eΓêÆ04┬á┬▒┬á0.8┬á┬▒┬á0.3┬á┬▒┬á0.5	4.833eΓêÆ04┬á┬▒┬á0.8┬á┬▒┬á0.3┬á┬▒┬á0.5	4.873eΓêÆ04┬á┬▒┬á0.8┬á┬▒┬á0.3┬á┬▒┬á0.5	4.858eΓêÆ04┬á┬▒┬á0.6┬á┬▒┬á0.2┬á┬▒┬á0.5
105.0ΓÇô150.0	1.855eΓêÆ04┬á┬▒┬á0.9┬á┬▒┬á0.3┬á┬▒┬á1.0	1.867eΓêÆ04┬á┬▒┬á0.9┬á┬▒┬á0.3┬á┬▒┬á1.0	1.821eΓêÆ04┬á┬▒┬á0.9┬á┬▒┬á0.2┬á┬▒┬á0.4	1.850eΓêÆ04┬á┬▒┬á0.9┬á┬▒┬á0.2┬á┬▒┬á0.4	1.866eΓêÆ04┬á┬▒┬á0.9┬á┬▒┬á0.2┬á┬▒┬á0.6	1.874eΓêÆ04┬á┬▒┬á0.6┬á┬▒┬á0.2┬á┬▒┬á0.5
150.0ΓÇô200.0	5.087eΓêÆ05┬á┬▒┬á1.5┬á┬▒┬á0.5┬á┬▒┬á1.3	5.110eΓêÆ05┬á┬▒┬á1.5┬á┬▒┬á0.5┬á┬▒┬á1.3	5.338eΓêÆ05┬á┬▒┬á1.6┬á┬▒┬á0.5┬á┬▒┬á0.7	5.453eΓêÆ05┬á┬▒┬á1.6┬á┬▒┬á0.5┬á┬▒┬á0.7	5.481eΓêÆ05┬á┬▒┬á1.6┬á┬▒┬á0.5┬á┬▒┬á0.8	5.303eΓêÆ05┬á┬▒┬á1.1┬á┬▒┬á0.4┬á┬▒┬á0.7
200.0ΓÇô900.0	2.117eΓêÆ06┬á┬▒┬á2.0┬á┬▒┬á0.6┬á┬▒┬á1.7	2.124eΓêÆ06┬á┬▒┬á1.9┬á┬▒┬á0.6┬á┬▒┬á1.8	2.095eΓêÆ06┬á┬▒┬á2.0┬á┬▒┬á0.6┬á┬▒┬á1.1	2.138eΓêÆ06┬á┬▒┬á2.0┬á┬▒┬á0.6┬á┬▒┬á1.1	2.142eΓêÆ06┬á┬▒┬á2.0┬á┬▒┬á0.6┬á┬▒┬á1.1	2.145eΓêÆ06┬á┬▒┬á1.4┬á┬▒┬á0.4┬á┬▒┬á1.0

**Table 22:** The values of $(1/\sigma )\,d\sigma /dp_\mathrm {T}^{\ell \ell }$ in each bin of $p_\mathrm {T}^{\ell \ell }$ for the electron and muon channels separately (for various generator-level definitions) and for the Born-level combination in the kinematic region $66 \text { GeV} \le m_{\ell \ell }< 116 \text { GeV},\ 0.8 \le |y_{\ell \ell }| < 1.2$. The associated statistical and systematic (both uncorrelated and correlated between bins) are provided in percentage form

Bin	$(1/\sigma )\,d\sigma /dp_\mathrm {T}^{\ell \ell }$ ┬▒ Statistical [%] ┬▒ Uncorrelated systematic [%] ┬▒ Correlated systematic [%]					
	Electron channel		Muon channel			Combination
	Dressed	Born	Bare	Dressed	Born	Born
0.0ΓÇô2.0	2.640eΓêÆ02┬á┬▒┬á0.6┬á┬▒┬á0.3┬á┬▒┬á1.0	2.708eΓêÆ02┬á┬▒┬á0.6┬á┬▒┬á0.3┬á┬▒┬á1.0	2.606eΓêÆ02┬á┬▒┬á0.5┬á┬▒┬á0.2┬á┬▒┬á0.9	2.662eΓêÆ02┬á┬▒┬á0.5┬á┬▒┬á0.2┬á┬▒┬á0.9	2.731eΓêÆ02┬á┬▒┬á0.5┬á┬▒┬á0.2┬á┬▒┬á0.9	2.711eΓêÆ02┬á┬▒┬á0.4┬á┬▒┬á0.2┬á┬▒┬á0.6
2.0ΓÇô4.0	5.532eΓêÆ02┬á┬▒┬á0.4┬á┬▒┬á0.2┬á┬▒┬á0.6	5.639eΓêÆ02┬á┬▒┬á0.4┬á┬▒┬á0.2┬á┬▒┬á0.6	5.406eΓêÆ02┬á┬▒┬á0.3┬á┬▒┬á0.2┬á┬▒┬á0.3	5.491eΓêÆ02┬á┬▒┬á0.3┬á┬▒┬á0.2┬á┬▒┬á0.3	5.597eΓêÆ02┬á┬▒┬á0.3┬á┬▒┬á0.2┬á┬▒┬á0.4	5.611eΓêÆ02┬á┬▒┬á0.2┬á┬▒┬á0.1┬á┬▒┬á0.3
4.0ΓÇô6.0	5.598eΓêÆ02┬á┬▒┬á0.4┬á┬▒┬á0.2┬á┬▒┬á0.3	5.653eΓêÆ02┬á┬▒┬á0.4┬á┬▒┬á0.2┬á┬▒┬á0.3	5.608eΓêÆ02┬á┬▒┬á0.3┬á┬▒┬á0.1┬á┬▒┬á0.4	5.645eΓêÆ02┬á┬▒┬á0.3┬á┬▒┬á0.1┬á┬▒┬á0.4	5.700eΓêÆ02┬á┬▒┬á0.3┬á┬▒┬á0.1┬á┬▒┬á0.5	5.691eΓêÆ02┬á┬▒┬á0.2┬á┬▒┬á0.1┬á┬▒┬á0.3
6.0ΓÇô8.0	4.889eΓêÆ02┬á┬▒┬á0.4┬á┬▒┬á0.2┬á┬▒┬á0.4	4.887eΓêÆ02┬á┬▒┬á0.4┬á┬▒┬á0.2┬á┬▒┬á0.4	4.919eΓêÆ02┬á┬▒┬á0.3┬á┬▒┬á0.1┬á┬▒┬á0.4	4.913eΓêÆ02┬á┬▒┬á0.3┬á┬▒┬á0.1┬á┬▒┬á0.4	4.913eΓêÆ02┬á┬▒┬á0.3┬á┬▒┬á0.1┬á┬▒┬á0.5	4.906eΓêÆ02┬á┬▒┬á0.3┬á┬▒┬á0.1┬á┬▒┬á0.3
8.0ΓÇô10.0	4.126eΓêÆ02┬á┬▒┬á0.5┬á┬▒┬á0.3┬á┬▒┬á0.5	4.095eΓêÆ02┬á┬▒┬á0.5┬á┬▒┬á0.3┬á┬▒┬á0.6	4.159eΓêÆ02┬á┬▒┬á0.4┬á┬▒┬á0.2┬á┬▒┬á0.4	4.132eΓêÆ02┬á┬▒┬á0.4┬á┬▒┬á0.2┬á┬▒┬á0.4	4.098eΓêÆ02┬á┬▒┬á0.4┬á┬▒┬á0.2┬á┬▒┬á0.6	4.096eΓêÆ02┬á┬▒┬á0.3┬á┬▒┬á0.1┬á┬▒┬á0.4
10.0ΓÇô13.0	3.288eΓêÆ02┬á┬▒┬á0.4┬á┬▒┬á0.2┬á┬▒┬á0.4	3.246eΓêÆ02┬á┬▒┬á0.4┬á┬▒┬á0.2┬á┬▒┬á0.4	3.324eΓêÆ02┬á┬▒┬á0.3┬á┬▒┬á0.1┬á┬▒┬á0.3	3.284eΓêÆ02┬á┬▒┬á0.3┬á┬▒┬á0.1┬á┬▒┬á0.3	3.242eΓêÆ02┬á┬▒┬á0.3┬á┬▒┬á0.1┬á┬▒┬á0.5	3.244eΓêÆ02┬á┬▒┬á0.3┬á┬▒┬á0.1┬á┬▒┬á0.3
13.0ΓÇô16.0	2.552eΓêÆ02┬á┬▒┬á0.5┬á┬▒┬á0.2┬á┬▒┬á0.4	2.515eΓêÆ02┬á┬▒┬á0.5┬á┬▒┬á0.2┬á┬▒┬á0.4	2.592eΓêÆ02┬á┬▒┬á0.4┬á┬▒┬á0.1┬á┬▒┬á0.3	2.556eΓêÆ02┬á┬▒┬á0.4┬á┬▒┬á0.1┬á┬▒┬á0.3	2.518eΓêÆ02┬á┬▒┬á0.4┬á┬▒┬á0.1┬á┬▒┬á0.3	2.519eΓêÆ02┬á┬▒┬á0.3┬á┬▒┬á0.1┬á┬▒┬á0.2
16.0ΓÇô20.0	1.917eΓêÆ02┬á┬▒┬á0.4┬á┬▒┬á0.2┬á┬▒┬á0.3	1.888eΓêÆ02┬á┬▒┬á0.4┬á┬▒┬á0.2┬á┬▒┬á0.3	1.942eΓêÆ02┬á┬▒┬á0.3┬á┬▒┬á0.1┬á┬▒┬á0.2	1.916eΓêÆ02┬á┬▒┬á0.3┬á┬▒┬á0.1┬á┬▒┬á0.2	1.887eΓêÆ02┬á┬▒┬á0.3┬á┬▒┬á0.1┬á┬▒┬á0.2	1.888eΓêÆ02┬á┬▒┬á0.3┬á┬▒┬á0.1┬á┬▒┬á0.2
20.0ΓÇô25.0	1.360eΓêÆ02┬á┬▒┬á0.4┬á┬▒┬á0.2┬á┬▒┬á0.3	1.346eΓêÆ02┬á┬▒┬á0.4┬á┬▒┬á0.2┬á┬▒┬á0.3	1.384eΓêÆ02┬á┬▒┬á0.3┬á┬▒┬á0.1┬á┬▒┬á0.2	1.373eΓêÆ02┬á┬▒┬á0.3┬á┬▒┬á0.1┬á┬▒┬á0.2	1.360eΓêÆ02┬á┬▒┬á0.3┬á┬▒┬á0.1┬á┬▒┬á0.2	1.355eΓêÆ02┬á┬▒┬á0.3┬á┬▒┬á0.1┬á┬▒┬á0.2
25.0ΓÇô30.0	9.826eΓêÆ03┬á┬▒┬á0.5┬á┬▒┬á0.2┬á┬▒┬á0.3	9.766eΓêÆ03┬á┬▒┬á0.5┬á┬▒┬á0.2┬á┬▒┬á0.3	9.730eΓêÆ03┬á┬▒┬á0.4┬á┬▒┬á0.1┬á┬▒┬á0.2	9.698eΓêÆ03┬á┬▒┬á0.4┬á┬▒┬á0.1┬á┬▒┬á0.2	9.646eΓêÆ03┬á┬▒┬á0.4┬á┬▒┬á0.1┬á┬▒┬á0.3	9.693eΓêÆ03┬á┬▒┬á0.3┬á┬▒┬á0.1┬á┬▒┬á0.2
30.0ΓÇô37.0	6.841eΓêÆ03┬á┬▒┬á0.5┬á┬▒┬á0.2┬á┬▒┬á0.4	6.830eΓêÆ03┬á┬▒┬á0.5┬á┬▒┬á0.2┬á┬▒┬á0.4	6.760eΓêÆ03┬á┬▒┬á0.4┬á┬▒┬á0.1┬á┬▒┬á0.2	6.757eΓêÆ03┬á┬▒┬á0.4┬á┬▒┬á0.1┬á┬▒┬á0.2	6.747eΓêÆ03┬á┬▒┬á0.4┬á┬▒┬á0.1┬á┬▒┬á0.3	6.777eΓêÆ03┬á┬▒┬á0.3┬á┬▒┬á0.1┬á┬▒┬á0.2
37.0ΓÇô45.0	4.559eΓêÆ03┬á┬▒┬á0.5┬á┬▒┬á0.2┬á┬▒┬á0.6	4.568eΓêÆ03┬á┬▒┬á0.5┬á┬▒┬á0.2┬á┬▒┬á0.6	4.510eΓêÆ03┬á┬▒┬á0.5┬á┬▒┬á0.1┬á┬▒┬á0.3	4.527eΓêÆ03┬á┬▒┬á0.5┬á┬▒┬á0.1┬á┬▒┬á0.3	4.532eΓêÆ03┬á┬▒┬á0.5┬á┬▒┬á0.1┬á┬▒┬á0.3	4.544eΓêÆ03┬á┬▒┬á0.3┬á┬▒┬á0.1┬á┬▒┬á0.3
45.0ΓÇô55.0	2.962eΓêÆ03┬á┬▒┬á0.6┬á┬▒┬á0.3┬á┬▒┬á0.8	2.975eΓêÆ03┬á┬▒┬á0.6┬á┬▒┬á0.3┬á┬▒┬á0.8	2.890eΓêÆ03┬á┬▒┬á0.5┬á┬▒┬á0.2┬á┬▒┬á0.3	2.909eΓêÆ03┬á┬▒┬á0.5┬á┬▒┬á0.2┬á┬▒┬á0.3	2.924eΓêÆ03┬á┬▒┬á0.5┬á┬▒┬á0.2┬á┬▒┬á0.4	2.940eΓêÆ03┬á┬▒┬á0.4┬á┬▒┬á0.2┬á┬▒┬á0.3
55.0ΓÇô65.0	1.903eΓêÆ03┬á┬▒┬á0.7┬á┬▒┬á0.3┬á┬▒┬á1.0	1.916eΓêÆ03┬á┬▒┬á0.7┬á┬▒┬á0.3┬á┬▒┬á1.0	1.873eΓêÆ03┬á┬▒┬á0.6┬á┬▒┬á0.2┬á┬▒┬á0.4	1.893eΓêÆ03┬á┬▒┬á0.6┬á┬▒┬á0.2┬á┬▒┬á0.4	1.907eΓêÆ03┬á┬▒┬á0.6┬á┬▒┬á0.2┬á┬▒┬á0.5	1.907eΓêÆ03┬á┬▒┬á0.5┬á┬▒┬á0.2┬á┬▒┬á0.5
65.0ΓÇô75.0	1.238eΓêÆ03┬á┬▒┬á0.9┬á┬▒┬á0.4┬á┬▒┬á1.2	1.249eΓêÆ03┬á┬▒┬á0.9┬á┬▒┬á0.4┬á┬▒┬á1.2	1.228eΓêÆ03┬á┬▒┬á0.8┬á┬▒┬á0.3┬á┬▒┬á0.5	1.242eΓêÆ03┬á┬▒┬á0.8┬á┬▒┬á0.3┬á┬▒┬á0.5	1.253eΓêÆ03┬á┬▒┬á0.8┬á┬▒┬á0.3┬á┬▒┬á0.5	1.249eΓêÆ03┬á┬▒┬á0.6┬á┬▒┬á0.2┬á┬▒┬á0.5
75.0ΓÇô85.0	8.166eΓêÆ04┬á┬▒┬á1.1┬á┬▒┬á0.6┬á┬▒┬á1.2	8.235eΓêÆ04┬á┬▒┬á1.1┬á┬▒┬á0.6┬á┬▒┬á1.2	8.238eΓêÆ04┬á┬▒┬á1.0┬á┬▒┬á0.4┬á┬▒┬á0.5	8.348eΓêÆ04┬á┬▒┬á1.0┬á┬▒┬á0.4┬á┬▒┬á0.5	8.421eΓêÆ04┬á┬▒┬á1.0┬á┬▒┬á0.4┬á┬▒┬á0.5	8.337eΓêÆ04┬á┬▒┬á0.7┬á┬▒┬á0.3┬á┬▒┬á0.5
85.0ΓÇô105.0	4.798eΓêÆ04┬á┬▒┬á1.0┬á┬▒┬á0.4┬á┬▒┬á1.0	4.844eΓêÆ04┬á┬▒┬á1.0┬á┬▒┬á0.4┬á┬▒┬á1.0	4.718eΓêÆ04┬á┬▒┬á0.8┬á┬▒┬á0.3┬á┬▒┬á0.5	4.786eΓêÆ04┬á┬▒┬á0.8┬á┬▒┬á0.3┬á┬▒┬á0.5	4.829eΓêÆ04┬á┬▒┬á0.8┬á┬▒┬á0.3┬á┬▒┬á0.5	4.832eΓêÆ04┬á┬▒┬á0.6┬á┬▒┬á0.2┬á┬▒┬á0.4
105.0ΓÇô150.0	1.807eΓêÆ04┬á┬▒┬á1.0┬á┬▒┬á0.4┬á┬▒┬á0.9	1.821eΓêÆ04┬á┬▒┬á1.0┬á┬▒┬á0.4┬á┬▒┬á0.9	1.802eΓêÆ04┬á┬▒┬á0.9┬á┬▒┬á0.3┬á┬▒┬á0.5	1.829eΓêÆ04┬á┬▒┬á0.9┬á┬▒┬á0.3┬á┬▒┬á0.5	1.840eΓêÆ04┬á┬▒┬á0.9┬á┬▒┬á0.3┬á┬▒┬á0.5	1.831eΓêÆ04┬á┬▒┬á0.6┬á┬▒┬á0.3┬á┬▒┬á0.5
150.0ΓÇô200.0	5.136eΓêÆ05┬á┬▒┬á1.7┬á┬▒┬á0.7┬á┬▒┬á1.4	5.166eΓêÆ05┬á┬▒┬á1.7┬á┬▒┬á0.7┬á┬▒┬á1.4	5.053eΓêÆ05┬á┬▒┬á1.6┬á┬▒┬á0.5┬á┬▒┬á1.0	5.152eΓêÆ05┬á┬▒┬á1.6┬á┬▒┬á0.5┬á┬▒┬á1.0	5.186eΓêÆ05┬á┬▒┬á1.6┬á┬▒┬á0.5┬á┬▒┬á1.0	5.182eΓêÆ05┬á┬▒┬á1.2┬á┬▒┬á0.4┬á┬▒┬á0.7
200.0ΓÇô900.0	2.031eΓêÆ06┬á┬▒┬á2.2┬á┬▒┬á0.7┬á┬▒┬á1.6	2.040eΓêÆ06┬á┬▒┬á2.2┬á┬▒┬á0.7┬á┬▒┬á1.6	2.025eΓêÆ06┬á┬▒┬á2.0┬á┬▒┬á0.7┬á┬▒┬á1.3	2.067eΓêÆ06┬á┬▒┬á2.0┬á┬▒┬á0.7┬á┬▒┬á1.3	2.078eΓêÆ06┬á┬▒┬á2.0┬á┬▒┬á0.7┬á┬▒┬á1.3	2.061eΓêÆ06┬á┬▒┬á1.5┬á┬▒┬á0.5┬á┬▒┬á0.9

**Table 23:** The values of $(1/\sigma )\,d\sigma /dp_\mathrm {T}^{\ell \ell }$ in each bin of $p_\mathrm {T}^{\ell \ell }$ for the electron and muon channels separately (for various generator-level definitions) and for the Born-level combination in the kinematic region $66 \text { GeV} \le m_{\ell \ell }< 116 \text { GeV},\ 1.2 \le |y_{\ell \ell }| < 1.6$. The associated statistical and systematic (both uncorrelated and correlated between bins) are provided in percentage form

Bin	$(1/\sigma )\,d\sigma /dp_\mathrm {T}^{\ell \ell }$ ┬▒ Statistical [%] ┬▒ Uncorrelated systematic [%] ┬▒ Correlated systematic [%]					
	Electron channel		Muon channel			Combination
	Dressed	Born	Bare	Dressed	Born	Born
0.0ΓÇô2.0	2.584eΓêÆ02┬á┬▒┬á0.6┬á┬▒┬á0.3┬á┬▒┬á1.1	2.655eΓêÆ02┬á┬▒┬á0.6┬á┬▒┬á0.3┬á┬▒┬á1.1	2.512eΓêÆ02┬á┬▒┬á0.5┬á┬▒┬á0.2┬á┬▒┬á0.9	2.573eΓêÆ02┬á┬▒┬á0.5┬á┬▒┬á0.2┬á┬▒┬á0.9	2.645eΓêÆ02┬á┬▒┬á0.5┬á┬▒┬á0.2┬á┬▒┬á0.9	2.658eΓêÆ02┬á┬▒┬á0.4┬á┬▒┬á0.2┬á┬▒┬á0.7
2.0ΓÇô4.0	5.405eΓêÆ02┬á┬▒┬á0.4┬á┬▒┬á0.2┬á┬▒┬á0.8	5.522eΓêÆ02┬á┬▒┬á0.4┬á┬▒┬á0.2┬á┬▒┬á0.8	5.308eΓêÆ02┬á┬▒┬á0.3┬á┬▒┬á0.2┬á┬▒┬á0.5	5.405eΓêÆ02┬á┬▒┬á0.3┬á┬▒┬á0.2┬á┬▒┬á0.5	5.525eΓêÆ02┬á┬▒┬á0.3┬á┬▒┬á0.2┬á┬▒┬á0.5	5.536eΓêÆ02┬á┬▒┬á0.3┬á┬▒┬á0.1┬á┬▒┬á0.4
4.0ΓÇô6.0	5.436eΓêÆ02┬á┬▒┬á0.4┬á┬▒┬á0.2┬á┬▒┬á0.5	5.494eΓêÆ02┬á┬▒┬á0.4┬á┬▒┬á0.2┬á┬▒┬á0.5	5.453eΓêÆ02┬á┬▒┬á0.3┬á┬▒┬á0.2┬á┬▒┬á0.4	5.498eΓêÆ02┬á┬▒┬á0.3┬á┬▒┬á0.2┬á┬▒┬á0.4	5.554eΓêÆ02┬á┬▒┬á0.3┬á┬▒┬á0.2┬á┬▒┬á0.4	5.529eΓêÆ02┬á┬▒┬á0.3┬á┬▒┬á0.2┬á┬▒┬á0.4
6.0ΓÇô8.0	4.804eΓêÆ02┬á┬▒┬á0.5┬á┬▒┬á0.3┬á┬▒┬á0.6	4.803eΓêÆ02┬á┬▒┬á0.5┬á┬▒┬á0.3┬á┬▒┬á0.6	4.836eΓêÆ02┬á┬▒┬á0.4┬á┬▒┬á0.2┬á┬▒┬á0.3	4.831eΓêÆ02┬á┬▒┬á0.4┬á┬▒┬á0.2┬á┬▒┬á0.3	4.829eΓêÆ02┬á┬▒┬á0.4┬á┬▒┬á0.2┬á┬▒┬á0.6	4.816eΓêÆ02┬á┬▒┬á0.3┬á┬▒┬á0.1┬á┬▒┬á0.5
8.0ΓÇô10.0	4.096eΓêÆ02┬á┬▒┬á0.5┬á┬▒┬á0.2┬á┬▒┬á0.5	4.057eΓêÆ02┬á┬▒┬á0.5┬á┬▒┬á0.2┬á┬▒┬á0.6	4.138eΓêÆ02┬á┬▒┬á0.4┬á┬▒┬á0.2┬á┬▒┬á0.4	4.104eΓêÆ02┬á┬▒┬á0.4┬á┬▒┬á0.2┬á┬▒┬á0.4	4.068eΓêÆ02┬á┬▒┬á0.4┬á┬▒┬á0.2┬á┬▒┬á0.6	4.066eΓêÆ02┬á┬▒┬á0.3┬á┬▒┬á0.2┬á┬▒┬á0.5
10.0ΓÇô13.0	3.316eΓêÆ02┬á┬▒┬á0.4┬á┬▒┬á0.2┬á┬▒┬á0.3	3.271eΓêÆ02┬á┬▒┬á0.4┬á┬▒┬á0.2┬á┬▒┬á0.4	3.354eΓêÆ02┬á┬▒┬á0.4┬á┬▒┬á0.1┬á┬▒┬á0.2	3.313eΓêÆ02┬á┬▒┬á0.4┬á┬▒┬á0.1┬á┬▒┬á0.2	3.268eΓêÆ02┬á┬▒┬á0.4┬á┬▒┬á0.1┬á┬▒┬á0.3	3.270eΓêÆ02┬á┬▒┬á0.3┬á┬▒┬á0.1┬á┬▒┬á0.3
13.0ΓÇô16.0	2.573eΓêÆ02┬á┬▒┬á0.5┬á┬▒┬á0.3┬á┬▒┬á0.4	2.531eΓêÆ02┬á┬▒┬á0.5┬á┬▒┬á0.3┬á┬▒┬á0.4	2.620eΓêÆ02┬á┬▒┬á0.4┬á┬▒┬á0.2┬á┬▒┬á0.3	2.580eΓêÆ02┬á┬▒┬á0.4┬á┬▒┬á0.2┬á┬▒┬á0.3	2.536eΓêÆ02┬á┬▒┬á0.4┬á┬▒┬á0.2┬á┬▒┬á0.3	2.534eΓêÆ02┬á┬▒┬á0.3┬á┬▒┬á0.1┬á┬▒┬á0.2
16.0ΓÇô20.0	1.956eΓêÆ02┬á┬▒┬á0.5┬á┬▒┬á0.2┬á┬▒┬á0.4	1.922eΓêÆ02┬á┬▒┬á0.5┬á┬▒┬á0.2┬á┬▒┬á0.4	1.974eΓêÆ02┬á┬▒┬á0.4┬á┬▒┬á0.2┬á┬▒┬á0.2	1.947eΓêÆ02┬á┬▒┬á0.4┬á┬▒┬á0.2┬á┬▒┬á0.2	1.914eΓêÆ02┬á┬▒┬á0.4┬á┬▒┬á0.2┬á┬▒┬á0.3	1.917eΓêÆ02┬á┬▒┬á0.3┬á┬▒┬á0.1┬á┬▒┬á0.2
20.0ΓÇô25.0	1.390eΓêÆ02┬á┬▒┬á0.5┬á┬▒┬á0.3┬á┬▒┬á0.4	1.375eΓêÆ02┬á┬▒┬á0.5┬á┬▒┬á0.3┬á┬▒┬á0.4	1.405eΓêÆ02┬á┬▒┬á0.4┬á┬▒┬á0.2┬á┬▒┬á0.3	1.390eΓêÆ02┬á┬▒┬á0.4┬á┬▒┬á0.2┬á┬▒┬á0.3	1.374eΓêÆ02┬á┬▒┬á0.4┬á┬▒┬á0.2┬á┬▒┬á0.3	1.374eΓêÆ02┬á┬▒┬á0.3┬á┬▒┬á0.1┬á┬▒┬á0.2
25.0ΓÇô30.0	1.000eΓêÆ02┬á┬▒┬á0.6┬á┬▒┬á0.3┬á┬▒┬á0.6	9.947eΓêÆ03┬á┬▒┬á0.6┬á┬▒┬á0.3┬á┬▒┬á0.6	1.002eΓêÆ02┬á┬▒┬á0.5┬á┬▒┬á0.2┬á┬▒┬á0.3	9.973eΓêÆ03┬á┬▒┬á0.5┬á┬▒┬á0.2┬á┬▒┬á0.3	9.918eΓêÆ03┬á┬▒┬á0.5┬á┬▒┬á0.2┬á┬▒┬á0.3	9.915eΓêÆ03┬á┬▒┬á0.4┬á┬▒┬á0.1┬á┬▒┬á0.3
30.0ΓÇô37.0	6.933eΓêÆ03┬á┬▒┬á0.6┬á┬▒┬á0.3┬á┬▒┬á0.6	6.929eΓêÆ03┬á┬▒┬á0.6┬á┬▒┬á0.3┬á┬▒┬á0.6	6.924eΓêÆ03┬á┬▒┬á0.4┬á┬▒┬á0.1┬á┬▒┬á0.3	6.928eΓêÆ03┬á┬▒┬á0.4┬á┬▒┬á0.1┬á┬▒┬á0.3	6.921eΓêÆ03┬á┬▒┬á0.4┬á┬▒┬á0.1┬á┬▒┬á0.3	6.918eΓêÆ03┬á┬▒┬á0.4┬á┬▒┬á0.1┬á┬▒┬á0.2
37.0ΓÇô45.0	4.611eΓêÆ03┬á┬▒┬á0.6┬á┬▒┬á0.3┬á┬▒┬á0.8	4.628eΓêÆ03┬á┬▒┬á0.6┬á┬▒┬á0.3┬á┬▒┬á0.8	4.576eΓêÆ03┬á┬▒┬á0.5┬á┬▒┬á0.2┬á┬▒┬á0.3	4.594eΓêÆ03┬á┬▒┬á0.5┬á┬▒┬á0.2┬á┬▒┬á0.3	4.605eΓêÆ03┬á┬▒┬á0.5┬á┬▒┬á0.2┬á┬▒┬á0.3	4.606eΓêÆ03┬á┬▒┬á0.4┬á┬▒┬á0.2┬á┬▒┬á0.3
45.0ΓÇô55.0	2.995eΓêÆ03┬á┬▒┬á0.7┬á┬▒┬á0.3┬á┬▒┬á1.0	3.012eΓêÆ03┬á┬▒┬á0.7┬á┬▒┬á0.3┬á┬▒┬á1.0	2.928eΓêÆ03┬á┬▒┬á0.5┬á┬▒┬á0.2┬á┬▒┬á0.3	2.949eΓêÆ03┬á┬▒┬á0.5┬á┬▒┬á0.2┬á┬▒┬á0.3	2.967eΓêÆ03┬á┬▒┬á0.5┬á┬▒┬á0.2┬á┬▒┬á0.3	2.978eΓêÆ03┬á┬▒┬á0.4┬á┬▒┬á0.2┬á┬▒┬á0.3
55.0ΓÇô65.0	1.940eΓêÆ03┬á┬▒┬á0.9┬á┬▒┬á0.4┬á┬▒┬á1.1	1.953eΓêÆ03┬á┬▒┬á0.9┬á┬▒┬á0.4┬á┬▒┬á1.1	1.902eΓêÆ03┬á┬▒┬á0.7┬á┬▒┬á0.3┬á┬▒┬á0.5	1.921eΓêÆ03┬á┬▒┬á0.7┬á┬▒┬á0.3┬á┬▒┬á0.5	1.938eΓêÆ03┬á┬▒┬á0.7┬á┬▒┬á0.3┬á┬▒┬á0.5	1.939eΓêÆ03┬á┬▒┬á0.5┬á┬▒┬á0.2┬á┬▒┬á0.4
65.0ΓÇô75.0	1.290eΓêÆ03┬á┬▒┬á1.1┬á┬▒┬á0.4┬á┬▒┬á1.2	1.303eΓêÆ03┬á┬▒┬á1.1┬á┬▒┬á0.4┬á┬▒┬á1.2	1.247eΓêÆ03┬á┬▒┬á0.9┬á┬▒┬á0.3┬á┬▒┬á0.5	1.263eΓêÆ03┬á┬▒┬á0.9┬á┬▒┬á0.3┬á┬▒┬á0.5	1.276eΓêÆ03┬á┬▒┬á0.9┬á┬▒┬á0.3┬á┬▒┬á0.6	1.284eΓêÆ03┬á┬▒┬á0.7┬á┬▒┬á0.3┬á┬▒┬á0.5
75.0ΓÇô85.0	8.719eΓêÆ04┬á┬▒┬á1.3┬á┬▒┬á0.5┬á┬▒┬á1.1	8.814eΓêÆ04┬á┬▒┬á1.3┬á┬▒┬á0.5┬á┬▒┬á1.1	8.569eΓêÆ04┬á┬▒┬á1.1┬á┬▒┬á0.4┬á┬▒┬á0.6	8.689eΓêÆ04┬á┬▒┬á1.1┬á┬▒┬á0.4┬á┬▒┬á0.6	8.800eΓêÆ04┬á┬▒┬á1.1┬á┬▒┬á0.4┬á┬▒┬á0.6	8.786eΓêÆ04┬á┬▒┬á0.8┬á┬▒┬á0.3┬á┬▒┬á0.5
85.0ΓÇô105.0	4.966eΓêÆ04┬á┬▒┬á1.1┬á┬▒┬á0.5┬á┬▒┬á1.0	5.025eΓêÆ04┬á┬▒┬á1.1┬á┬▒┬á0.5┬á┬▒┬á1.1	4.805eΓêÆ04┬á┬▒┬á0.9┬á┬▒┬á0.3┬á┬▒┬á0.5	4.877eΓêÆ04┬á┬▒┬á0.9┬á┬▒┬á0.3┬á┬▒┬á0.5	4.928eΓêÆ04┬á┬▒┬á0.9┬á┬▒┬á0.3┬á┬▒┬á0.5	4.959eΓêÆ04┬á┬▒┬á0.7┬á┬▒┬á0.3┬á┬▒┬á0.5
105.0ΓÇô150.0	1.933eΓêÆ04┬á┬▒┬á1.1┬á┬▒┬á0.4┬á┬▒┬á1.2	1.950eΓêÆ04┬á┬▒┬á1.1┬á┬▒┬á0.4┬á┬▒┬á1.2	1.879eΓêÆ04┬á┬▒┬á0.9┬á┬▒┬á0.3┬á┬▒┬á0.5	1.909eΓêÆ04┬á┬▒┬á0.9┬á┬▒┬á0.3┬á┬▒┬á0.5	1.927eΓêÆ04┬á┬▒┬á0.9┬á┬▒┬á0.3┬á┬▒┬á0.6	1.936eΓêÆ04┬á┬▒┬á0.7┬á┬▒┬á0.2┬á┬▒┬á0.5
150.0ΓÇô200.0	5.229eΓêÆ05┬á┬▒┬á2.0┬á┬▒┬á0.7┬á┬▒┬á1.7	5.271eΓêÆ05┬á┬▒┬á2.0┬á┬▒┬á0.7┬á┬▒┬á1.7	5.304eΓêÆ05┬á┬▒┬á1.7┬á┬▒┬á0.5┬á┬▒┬á0.7	5.410eΓêÆ05┬á┬▒┬á1.7┬á┬▒┬á0.5┬á┬▒┬á0.7	5.445eΓêÆ05┬á┬▒┬á1.7┬á┬▒┬á0.5┬á┬▒┬á0.7	5.363eΓêÆ05┬á┬▒┬á1.3┬á┬▒┬á0.4┬á┬▒┬á0.7
200.0ΓÇô900.0	2.078eΓêÆ06┬á┬▒┬á2.7┬á┬▒┬á1.0┬á┬▒┬á3.3	2.084eΓêÆ06┬á┬▒┬á2.7┬á┬▒┬á1.0┬á┬▒┬á3.3	2.031eΓêÆ06┬á┬▒┬á2.2┬á┬▒┬á0.6┬á┬▒┬á0.8	2.095eΓêÆ06┬á┬▒┬á2.2┬á┬▒┬á0.6┬á┬▒┬á0.8	2.109eΓêÆ06┬á┬▒┬á2.2┬á┬▒┬á0.6┬á┬▒┬á0.9	2.099eΓêÆ06┬á┬▒┬á1.7┬á┬▒┬á0.5┬á┬▒┬á1.1

**Table 24:** The values of $(1/\sigma )\,d\sigma /dp_\mathrm {T}^{\ell \ell }$ in each bin of $p_\mathrm {T}^{\ell \ell }$ for the electron and muon channels separately (for various generator-level definitions) and for the Born-level combination in the kinematic region $66 \text { GeV} \le m_{\ell \ell }< 116 \text { GeV},\ 1.6 \le |y_{\ell \ell }| < 2.0$. The associated statistical and systematic (both uncorrelated and correlated between bins) are provided in percentage form

Bin	$(1/\sigma )\,d\sigma /dp_\mathrm {T}^{\ell \ell }$ ┬▒ Statistical [%] ┬▒ Uncorrelated systematic [%] ┬▒ Correlated systematic [%]					
	Electron channel		Muon channel			Combination
	Dressed	Born	Bare	Dressed	Born	Born
0.0ΓÇô2.0	2.536eΓêÆ02┬á┬▒┬á0.8┬á┬▒┬á0.5┬á┬▒┬á1.5	2.616eΓêÆ02┬á┬▒┬á0.8┬á┬▒┬á0.5┬á┬▒┬á1.5	2.466eΓêÆ02┬á┬▒┬á0.6┬á┬▒┬á0.3┬á┬▒┬á1.2	2.541eΓêÆ02┬á┬▒┬á0.6┬á┬▒┬á0.3┬á┬▒┬á1.2	2.618eΓêÆ02┬á┬▒┬á0.6┬á┬▒┬á0.3┬á┬▒┬á1.2	2.606eΓêÆ02┬á┬▒┬á0.5┬á┬▒┬á0.3┬á┬▒┬á0.9
2.0ΓÇô4.0	5.324eΓêÆ02┬á┬▒┬á0.5┬á┬▒┬á0.3┬á┬▒┬á1.1	5.454eΓêÆ02┬á┬▒┬á0.5┬á┬▒┬á0.3┬á┬▒┬á1.2	5.139eΓêÆ02┬á┬▒┬á0.4┬á┬▒┬á0.2┬á┬▒┬á0.6	5.254eΓêÆ02┬á┬▒┬á0.4┬á┬▒┬á0.2┬á┬▒┬á0.6	5.382eΓêÆ02┬á┬▒┬á0.4┬á┬▒┬á0.2┬á┬▒┬á0.7	5.394eΓêÆ02┬á┬▒┬á0.3┬á┬▒┬á0.2┬á┬▒┬á0.6
4.0ΓÇô6.0	5.345eΓêÆ02┬á┬▒┬á0.5┬á┬▒┬á0.3┬á┬▒┬á0.6	5.408eΓêÆ02┬á┬▒┬á0.5┬á┬▒┬á0.3┬á┬▒┬á0.6	5.307eΓêÆ02┬á┬▒┬á0.4┬á┬▒┬á0.2┬á┬▒┬á0.3	5.366eΓêÆ02┬á┬▒┬á0.4┬á┬▒┬á0.2┬á┬▒┬á0.3	5.433eΓêÆ02┬á┬▒┬á0.4┬á┬▒┬á0.2┬á┬▒┬á0.5	5.425eΓêÆ02┬á┬▒┬á0.3┬á┬▒┬á0.2┬á┬▒┬á0.4
6.0ΓÇô8.0	4.640eΓêÆ02┬á┬▒┬á0.6┬á┬▒┬á0.3┬á┬▒┬á0.9	4.646eΓêÆ02┬á┬▒┬á0.6┬á┬▒┬á0.3┬á┬▒┬á0.9	4.678eΓêÆ02┬á┬▒┬á0.4┬á┬▒┬á0.3┬á┬▒┬á0.5	4.674eΓêÆ02┬á┬▒┬á0.4┬á┬▒┬á0.3┬á┬▒┬á0.5	4.679eΓêÆ02┬á┬▒┬á0.4┬á┬▒┬á0.3┬á┬▒┬á0.9	4.667eΓêÆ02┬á┬▒┬á0.3┬á┬▒┬á0.2┬á┬▒┬á0.6
8.0ΓÇô10.0	3.960eΓêÆ02┬á┬▒┬á0.6┬á┬▒┬á0.3┬á┬▒┬á0.8	3.931eΓêÆ02┬á┬▒┬á0.6┬á┬▒┬á0.3┬á┬▒┬á0.9	3.978eΓêÆ02┬á┬▒┬á0.5┬á┬▒┬á0.2┬á┬▒┬á0.4	3.950eΓêÆ02┬á┬▒┬á0.5┬á┬▒┬á0.2┬á┬▒┬á0.4	3.922eΓêÆ02┬á┬▒┬á0.5┬á┬▒┬á0.2┬á┬▒┬á0.5	3.925eΓêÆ02┬á┬▒┬á0.4┬á┬▒┬á0.2┬á┬▒┬á0.4
10.0ΓÇô13.0	3.241eΓêÆ02┬á┬▒┬á0.6┬á┬▒┬á0.3┬á┬▒┬á0.5	3.194eΓêÆ02┬á┬▒┬á0.6┬á┬▒┬á0.3┬á┬▒┬á0.5	3.259eΓêÆ02┬á┬▒┬á0.4┬á┬▒┬á0.2┬á┬▒┬á0.3	3.216eΓêÆ02┬á┬▒┬á0.4┬á┬▒┬á0.2┬á┬▒┬á0.3	3.170eΓêÆ02┬á┬▒┬á0.4┬á┬▒┬á0.2┬á┬▒┬á0.3	3.180eΓêÆ02┬á┬▒┬á0.3┬á┬▒┬á0.2┬á┬▒┬á0.2
13.0ΓÇô16.0	2.554eΓêÆ02┬á┬▒┬á0.7┬á┬▒┬á0.3┬á┬▒┬á0.6	2.503eΓêÆ02┬á┬▒┬á0.7┬á┬▒┬á0.3┬á┬▒┬á0.6	2.580eΓêÆ02┬á┬▒┬á0.5┬á┬▒┬á0.2┬á┬▒┬á0.4	2.531eΓêÆ02┬á┬▒┬á0.5┬á┬▒┬á0.2┬á┬▒┬á0.4	2.480eΓêÆ02┬á┬▒┬á0.5┬á┬▒┬á0.2┬á┬▒┬á0.4	2.489eΓêÆ02┬á┬▒┬á0.4┬á┬▒┬á0.2┬á┬▒┬á0.3
16.0ΓÇô20.0	1.920eΓêÆ02┬á┬▒┬á0.6┬á┬▒┬á0.3┬á┬▒┬á0.6	1.882eΓêÆ02┬á┬▒┬á0.6┬á┬▒┬á0.3┬á┬▒┬á0.6	2.009eΓêÆ02┬á┬▒┬á0.5┬á┬▒┬á0.2┬á┬▒┬á0.3	1.968eΓêÆ02┬á┬▒┬á0.5┬á┬▒┬á0.2┬á┬▒┬á0.3	1.929eΓêÆ02┬á┬▒┬á0.5┬á┬▒┬á0.2┬á┬▒┬á0.4	1.914eΓêÆ02┬á┬▒┬á0.4┬á┬▒┬á0.2┬á┬▒┬á0.3
20.0ΓÇô25.0	1.431eΓêÆ02┬á┬▒┬á0.6┬á┬▒┬á0.3┬á┬▒┬á0.5	1.409eΓêÆ02┬á┬▒┬á0.6┬á┬▒┬á0.3┬á┬▒┬á0.5	1.436eΓêÆ02┬á┬▒┬á0.5┬á┬▒┬á0.2┬á┬▒┬á0.4	1.419eΓêÆ02┬á┬▒┬á0.5┬á┬▒┬á0.2┬á┬▒┬á0.4	1.398eΓêÆ02┬á┬▒┬á0.5┬á┬▒┬á0.2┬á┬▒┬á0.5	1.403eΓêÆ02┬á┬▒┬á0.4┬á┬▒┬á0.2┬á┬▒┬á0.3
25.0ΓÇô30.0	1.042eΓêÆ02┬á┬▒┬á0.8┬á┬▒┬á0.4┬á┬▒┬á0.7	1.033eΓêÆ02┬á┬▒┬á0.8┬á┬▒┬á0.4┬á┬▒┬á0.7	1.036eΓêÆ02┬á┬▒┬á0.6┬á┬▒┬á0.2┬á┬▒┬á0.4	1.029eΓêÆ02┬á┬▒┬á0.6┬á┬▒┬á0.2┬á┬▒┬á0.4	1.020eΓêÆ02┬á┬▒┬á0.6┬á┬▒┬á0.2┬á┬▒┬á0.5	1.025eΓêÆ02┬á┬▒┬á0.5┬á┬▒┬á0.2┬á┬▒┬á0.4
30.0ΓÇô37.0	7.322eΓêÆ03┬á┬▒┬á0.7┬á┬▒┬á0.3┬á┬▒┬á0.8	7.307eΓêÆ03┬á┬▒┬á0.7┬á┬▒┬á0.3┬á┬▒┬á0.8	7.463eΓêÆ03┬á┬▒┬á0.5┬á┬▒┬á0.2┬á┬▒┬á0.4	7.452eΓêÆ03┬á┬▒┬á0.5┬á┬▒┬á0.2┬á┬▒┬á0.4	7.429eΓêÆ03┬á┬▒┬á0.5┬á┬▒┬á0.2┬á┬▒┬á0.4	7.398eΓêÆ03┬á┬▒┬á0.4┬á┬▒┬á0.2┬á┬▒┬á0.3
37.0ΓÇô45.0	4.924eΓêÆ03┬á┬▒┬á0.8┬á┬▒┬á0.4┬á┬▒┬á1.0	4.937eΓêÆ03┬á┬▒┬á0.8┬á┬▒┬á0.4┬á┬▒┬á1.0	4.975eΓêÆ03┬á┬▒┬á0.6┬á┬▒┬á0.2┬á┬▒┬á0.4	4.998eΓêÆ03┬á┬▒┬á0.6┬á┬▒┬á0.2┬á┬▒┬á0.4	5.015eΓêÆ03┬á┬▒┬á0.6┬á┬▒┬á0.2┬á┬▒┬á0.4	4.994eΓêÆ03┬á┬▒┬á0.5┬á┬▒┬á0.2┬á┬▒┬á0.4
45.0ΓÇô55.0	3.207eΓêÆ03┬á┬▒┬á0.8┬á┬▒┬á0.4┬á┬▒┬á1.4	3.225eΓêÆ03┬á┬▒┬á0.8┬á┬▒┬á0.4┬á┬▒┬á1.4	3.196eΓêÆ03┬á┬▒┬á0.7┬á┬▒┬á0.2┬á┬▒┬á0.4	3.221eΓêÆ03┬á┬▒┬á0.7┬á┬▒┬á0.2┬á┬▒┬á0.4	3.241eΓêÆ03┬á┬▒┬á0.7┬á┬▒┬á0.2┬á┬▒┬á0.4	3.241eΓêÆ03┬á┬▒┬á0.5┬á┬▒┬á0.2┬á┬▒┬á0.4
55.0ΓÇô65.0	2.042eΓêÆ03┬á┬▒┬á1.0┬á┬▒┬á0.5┬á┬▒┬á1.6	2.062eΓêÆ03┬á┬▒┬á1.0┬á┬▒┬á0.5┬á┬▒┬á1.6	2.002eΓêÆ03┬á┬▒┬á0.9┬á┬▒┬á0.4┬á┬▒┬á0.6	2.022eΓêÆ03┬á┬▒┬á0.9┬á┬▒┬á0.4┬á┬▒┬á0.6	2.044eΓêÆ03┬á┬▒┬á0.9┬á┬▒┬á0.4┬á┬▒┬á0.6	2.052eΓêÆ03┬á┬▒┬á0.7┬á┬▒┬á0.3┬á┬▒┬á0.6
65.0ΓÇô75.0	1.333eΓêÆ03┬á┬▒┬á1.3┬á┬▒┬á0.6┬á┬▒┬á1.8	1.350eΓêÆ03┬á┬▒┬á1.3┬á┬▒┬á0.6┬á┬▒┬á1.8	1.311eΓêÆ03┬á┬▒┬á1.1┬á┬▒┬á0.4┬á┬▒┬á0.6	1.330eΓêÆ03┬á┬▒┬á1.1┬á┬▒┬á0.4┬á┬▒┬á0.6	1.344eΓêÆ03┬á┬▒┬á1.1┬á┬▒┬á0.4┬á┬▒┬á0.6	1.349eΓêÆ03┬á┬▒┬á0.8┬á┬▒┬á0.4┬á┬▒┬á0.6
75.0ΓÇô85.0	9.113eΓêÆ04┬á┬▒┬á1.5┬á┬▒┬á0.6┬á┬▒┬á2.0	9.246eΓêÆ04┬á┬▒┬á1.5┬á┬▒┬á0.6┬á┬▒┬á2.0	8.804eΓêÆ04┬á┬▒┬á1.4┬á┬▒┬á0.5┬á┬▒┬á0.9	8.952eΓêÆ04┬á┬▒┬á1.4┬á┬▒┬á0.5┬á┬▒┬á0.9	9.085eΓêÆ04┬á┬▒┬á1.4┬á┬▒┬á0.5┬á┬▒┬á0.9	9.165eΓêÆ04┬á┬▒┬á1.0┬á┬▒┬á0.4┬á┬▒┬á0.8
85.0ΓÇô105.0	5.245eΓêÆ04┬á┬▒┬á1.3┬á┬▒┬á0.6┬á┬▒┬á1.3	5.298eΓêÆ04┬á┬▒┬á1.3┬á┬▒┬á0.6┬á┬▒┬á1.3	5.142eΓêÆ04┬á┬▒┬á1.1┬á┬▒┬á0.4┬á┬▒┬á0.7	5.206eΓêÆ04┬á┬▒┬á1.1┬á┬▒┬á0.4┬á┬▒┬á0.7	5.271eΓêÆ04┬á┬▒┬á1.1┬á┬▒┬á0.4┬á┬▒┬á0.8	5.290eΓêÆ04┬á┬▒┬á0.8┬á┬▒┬á0.4┬á┬▒┬á0.6
105.0ΓÇô150.0	2.042eΓêÆ04┬á┬▒┬á1.3┬á┬▒┬á0.5┬á┬▒┬á1.3	2.064eΓêÆ04┬á┬▒┬á1.3┬á┬▒┬á0.5┬á┬▒┬á1.3	1.994eΓêÆ04┬á┬▒┬á1.1┬á┬▒┬á0.3┬á┬▒┬á0.6	2.030eΓêÆ04┬á┬▒┬á1.1┬á┬▒┬á0.3┬á┬▒┬á0.6	2.053eΓêÆ04┬á┬▒┬á1.1┬á┬▒┬á0.3┬á┬▒┬á0.6	2.056eΓêÆ04┬á┬▒┬á0.8┬á┬▒┬á0.3┬á┬▒┬á0.5
150.0ΓÇô200.0	5.886eΓêÆ05┬á┬▒┬á2.2┬á┬▒┬á0.9┬á┬▒┬á1.9	5.962eΓêÆ05┬á┬▒┬á2.2┬á┬▒┬á0.9┬á┬▒┬á1.9	5.851eΓêÆ05┬á┬▒┬á1.9┬á┬▒┬á0.6┬á┬▒┬á0.8	5.976eΓêÆ05┬á┬▒┬á1.9┬á┬▒┬á0.6┬á┬▒┬á0.8	6.050eΓêÆ05┬á┬▒┬á1.9┬á┬▒┬á0.6┬á┬▒┬á0.9	6.014eΓêÆ05┬á┬▒┬á1.5┬á┬▒┬á0.5┬á┬▒┬á0.8
200.0ΓÇô900.0	2.048eΓêÆ06┬á┬▒┬á3.2┬á┬▒┬á1.3┬á┬▒┬á3.4	2.078eΓêÆ06┬á┬▒┬á3.2┬á┬▒┬á1.3┬á┬▒┬á3.5	1.972eΓêÆ06┬á┬▒┬á2.8┬á┬▒┬á0.8┬á┬▒┬á1.1	2.036eΓêÆ06┬á┬▒┬á2.8┬á┬▒┬á0.8┬á┬▒┬á1.1	2.054eΓêÆ06┬á┬▒┬á2.8┬á┬▒┬á0.8┬á┬▒┬á1.2	2.060eΓêÆ06┬á┬▒┬á2.1┬á┬▒┬á0.7┬á┬▒┬á1.3

**Table 25:** The values of $(1/\sigma )\,d\sigma /dp_\mathrm {T}^{\ell \ell }$ in each bin of $p_\mathrm {T}^{\ell \ell }$ for the electron and muon channels separately (for various generator-level definitions) and for the Born-level combination in the kinematic region $66 ~\text {GeV} \le m_{\ell \ell }< 116 ~\text {GeV},\ 2.0 \le |y_{\ell \ell }| < 2.4$. The associated statistical and systematic (both uncorrelated and correlated between bins) are provided in percentage form

Bin	$(1/\sigma )\,d\sigma /dp_\mathrm {T}^{\ell \ell }$ ┬▒ Statistical [%] ┬▒ Uncorrelated systematic [%] ┬▒ Correlated systematic [%]					
	Electron channel		Muon channel			Combination
	Dressed	Born	Bare	Dressed	Born	Born
0.0ΓÇô2.0	2.582eΓêÆ02┬á┬▒┬á1.3┬á┬▒┬á0.6┬á┬▒┬á2.3	2.668eΓêÆ02┬á┬▒┬á1.3┬á┬▒┬á0.6┬á┬▒┬á2.3	2.483eΓêÆ02┬á┬▒┬á1.1┬á┬▒┬á0.5┬á┬▒┬á2.0	2.563eΓêÆ02┬á┬▒┬á1.1┬á┬▒┬á0.5┬á┬▒┬á2.0	2.646eΓêÆ02┬á┬▒┬á1.1┬á┬▒┬á0.5┬á┬▒┬á2.0	2.676eΓêÆ02┬á┬▒┬á0.8┬á┬▒┬á0.4┬á┬▒┬á1.5
2.0ΓÇô4.0	5.224eΓêÆ02┬á┬▒┬á0.8┬á┬▒┬á0.5┬á┬▒┬á1.5	5.354eΓêÆ02┬á┬▒┬á0.8┬á┬▒┬á0.5┬á┬▒┬á1.5	5.173eΓêÆ02┬á┬▒┬á0.7┬á┬▒┬á0.3┬á┬▒┬á1.1	5.296eΓêÆ02┬á┬▒┬á0.7┬á┬▒┬á0.3┬á┬▒┬á1.1	5.432eΓêÆ02┬á┬▒┬á0.7┬á┬▒┬á0.3┬á┬▒┬á1.2	5.429eΓêÆ02┬á┬▒┬á0.5┬á┬▒┬á0.3┬á┬▒┬á0.9
4.0ΓÇô6.0	5.276eΓêÆ02┬á┬▒┬á0.8┬á┬▒┬á0.5┬á┬▒┬á1.0	5.345eΓêÆ02┬á┬▒┬á0.8┬á┬▒┬á0.5┬á┬▒┬á1.0	5.203eΓêÆ02┬á┬▒┬á0.7┬á┬▒┬á0.4┬á┬▒┬á0.8	5.274eΓêÆ02┬á┬▒┬á0.7┬á┬▒┬á0.4┬á┬▒┬á0.8	5.337eΓêÆ02┬á┬▒┬á0.7┬á┬▒┬á0.4┬á┬▒┬á0.8	5.338eΓêÆ02┬á┬▒┬á0.6┬á┬▒┬á0.3┬á┬▒┬á0.6
6.0ΓÇô8.0	4.545eΓêÆ02┬á┬▒┬á0.9┬á┬▒┬á0.5┬á┬▒┬á0.9	4.558eΓêÆ02┬á┬▒┬á0.9┬á┬▒┬á0.5┬á┬▒┬á1.0	4.548eΓêÆ02┬á┬▒┬á0.8┬á┬▒┬á0.4┬á┬▒┬á1.0	4.556eΓêÆ02┬á┬▒┬á0.8┬á┬▒┬á0.4┬á┬▒┬á1.0	4.575eΓêÆ02┬á┬▒┬á0.8┬á┬▒┬á0.4┬á┬▒┬á1.0	4.567eΓêÆ02┬á┬▒┬á0.6┬á┬▒┬á0.3┬á┬▒┬á0.7
8.0ΓÇô10.0	3.928eΓêÆ02┬á┬▒┬á1.0┬á┬▒┬á0.5┬á┬▒┬á0.9	3.904eΓêÆ02┬á┬▒┬á1.0┬á┬▒┬á0.5┬á┬▒┬á0.9	3.903eΓêÆ02┬á┬▒┬á0.8┬á┬▒┬á0.4┬á┬▒┬á0.7	3.872eΓêÆ02┬á┬▒┬á0.8┬á┬▒┬á0.4┬á┬▒┬á0.7	3.850eΓêÆ02┬á┬▒┬á0.8┬á┬▒┬á0.4┬á┬▒┬á0.8	3.867eΓêÆ02┬á┬▒┬á0.6┬á┬▒┬á0.3┬á┬▒┬á0.6
10.0ΓÇô13.0	3.116eΓêÆ02┬á┬▒┬á0.9┬á┬▒┬á0.4┬á┬▒┬á0.7	3.075eΓêÆ02┬á┬▒┬á0.9┬á┬▒┬á0.4┬á┬▒┬á0.7	3.216eΓêÆ02┬á┬▒┬á0.7┬á┬▒┬á0.4┬á┬▒┬á0.7	3.175eΓêÆ02┬á┬▒┬á0.7┬á┬▒┬á0.4┬á┬▒┬á0.7	3.121eΓêÆ02┬á┬▒┬á0.7┬á┬▒┬á0.4┬á┬▒┬á0.8	3.100eΓêÆ02┬á┬▒┬á0.6┬á┬▒┬á0.3┬á┬▒┬á0.5
13.0ΓÇô16.0	2.418eΓêÆ02┬á┬▒┬á1.1┬á┬▒┬á0.6┬á┬▒┬á0.8	2.369eΓêÆ02┬á┬▒┬á1.1┬á┬▒┬á0.6┬á┬▒┬á0.8	2.523eΓêÆ02┬á┬▒┬á0.9┬á┬▒┬á0.4┬á┬▒┬á0.7	2.473eΓêÆ02┬á┬▒┬á0.9┬á┬▒┬á0.4┬á┬▒┬á0.7	2.423eΓêÆ02┬á┬▒┬á0.9┬á┬▒┬á0.4┬á┬▒┬á0.7	2.401eΓêÆ02┬á┬▒┬á0.7┬á┬▒┬á0.3┬á┬▒┬á0.5
16.0ΓÇô20.0	1.935eΓêÆ02┬á┬▒┬á1.0┬á┬▒┬á0.4┬á┬▒┬á0.6	1.890eΓêÆ02┬á┬▒┬á1.0┬á┬▒┬á0.4┬á┬▒┬á0.6	1.954eΓêÆ02┬á┬▒┬á0.8┬á┬▒┬á0.4┬á┬▒┬á0.5	1.909eΓêÆ02┬á┬▒┬á0.8┬á┬▒┬á0.4┬á┬▒┬á0.5	1.870eΓêÆ02┬á┬▒┬á0.8┬á┬▒┬á0.4┬á┬▒┬á0.6	1.877eΓêÆ02┬á┬▒┬á0.6┬á┬▒┬á0.3┬á┬▒┬á0.4
20.0ΓÇô25.0	1.407eΓêÆ02┬á┬▒┬á1.0┬á┬▒┬á0.6┬á┬▒┬á0.8	1.383eΓêÆ02┬á┬▒┬á1.0┬á┬▒┬á0.6┬á┬▒┬á0.8	1.438eΓêÆ02┬á┬▒┬á0.9┬á┬▒┬á0.3┬á┬▒┬á0.5	1.417eΓêÆ02┬á┬▒┬á0.9┬á┬▒┬á0.3┬á┬▒┬á0.5	1.390eΓêÆ02┬á┬▒┬á0.9┬á┬▒┬á0.3┬á┬▒┬á0.8	1.386eΓêÆ02┬á┬▒┬á0.7┬á┬▒┬á0.3┬á┬▒┬á0.5
25.0ΓÇô30.0	1.040eΓêÆ02┬á┬▒┬á1.1┬á┬▒┬á0.7┬á┬▒┬á0.9	1.032eΓêÆ02┬á┬▒┬á1.1┬á┬▒┬á0.7┬á┬▒┬á0.9	1.029eΓêÆ02┬á┬▒┬á1.0┬á┬▒┬á0.5┬á┬▒┬á0.7	1.020eΓêÆ02┬á┬▒┬á1.0┬á┬▒┬á0.5┬á┬▒┬á0.7	1.011eΓêÆ02┬á┬▒┬á1.0┬á┬▒┬á0.5┬á┬▒┬á0.9	1.019eΓêÆ02┬á┬▒┬á0.8┬á┬▒┬á0.4┬á┬▒┬á0.6
30.0ΓÇô37.0	7.412eΓêÆ03┬á┬▒┬á1.1┬á┬▒┬á0.7┬á┬▒┬á0.8	7.365eΓêÆ03┬á┬▒┬á1.1┬á┬▒┬á0.7┬á┬▒┬á0.8	7.470eΓêÆ03┬á┬▒┬á1.0┬á┬▒┬á0.3┬á┬▒┬á0.5	7.424eΓêÆ03┬á┬▒┬á1.0┬á┬▒┬á0.3┬á┬▒┬á0.5	7.404eΓêÆ03┬á┬▒┬á1.0┬á┬▒┬á0.3┬á┬▒┬á0.6	7.377eΓêÆ03┬á┬▒┬á0.7┬á┬▒┬á0.3┬á┬▒┬á0.5
37.0ΓÇô45.0	5.299eΓêÆ03┬á┬▒┬á1.1┬á┬▒┬á0.5┬á┬▒┬á0.9	5.288eΓêÆ03┬á┬▒┬á1.1┬á┬▒┬á0.5┬á┬▒┬á1.0	5.210eΓêÆ03┬á┬▒┬á1.1┬á┬▒┬á0.3┬á┬▒┬á0.5	5.215eΓêÆ03┬á┬▒┬á1.1┬á┬▒┬á0.3┬á┬▒┬á0.5	5.197eΓêÆ03┬á┬▒┬á1.1┬á┬▒┬á0.3┬á┬▒┬á0.5	5.226eΓêÆ03┬á┬▒┬á0.8┬á┬▒┬á0.3┬á┬▒┬á0.5
45.0ΓÇô55.0	3.549eΓêÆ03┬á┬▒┬á1.2┬á┬▒┬á0.7┬á┬▒┬á1.3	3.566eΓêÆ03┬á┬▒┬á1.2┬á┬▒┬á0.7┬á┬▒┬á1.3	3.461eΓêÆ03┬á┬▒┬á1.1┬á┬▒┬á0.4┬á┬▒┬á0.6	3.478eΓêÆ03┬á┬▒┬á1.1┬á┬▒┬á0.4┬á┬▒┬á0.6	3.502eΓêÆ03┬á┬▒┬á1.1┬á┬▒┬á0.4┬á┬▒┬á0.6	3.517eΓêÆ03┬á┬▒┬á0.8┬á┬▒┬á0.3┬á┬▒┬á0.6
55.0ΓÇô65.0	2.400eΓêÆ03┬á┬▒┬á1.5┬á┬▒┬á0.6┬á┬▒┬á1.5	2.423eΓêÆ03┬á┬▒┬á1.5┬á┬▒┬á0.6┬á┬▒┬á1.5	2.295eΓêÆ03┬á┬▒┬á1.4┬á┬▒┬á0.5┬á┬▒┬á0.7	2.325eΓêÆ03┬á┬▒┬á1.4┬á┬▒┬á0.5┬á┬▒┬á0.7	2.342eΓêÆ03┬á┬▒┬á1.4┬á┬▒┬á0.5┬á┬▒┬á0.7	2.368eΓêÆ03┬á┬▒┬á1.0┬á┬▒┬á0.4┬á┬▒┬á0.6
65.0ΓÇô75.0	1.534eΓêÆ03┬á┬▒┬á1.8┬á┬▒┬á0.9┬á┬▒┬á1.9	1.556eΓêÆ03┬á┬▒┬á1.8┬á┬▒┬á0.9┬á┬▒┬á2.0	1.499eΓêÆ03┬á┬▒┬á1.7┬á┬▒┬á0.7┬á┬▒┬á1.4	1.519eΓêÆ03┬á┬▒┬á1.7┬á┬▒┬á0.7┬á┬▒┬á1.4	1.541eΓêÆ03┬á┬▒┬á1.7┬á┬▒┬á0.7┬á┬▒┬á1.4	1.538eΓêÆ03┬á┬▒┬á1.3┬á┬▒┬á0.6┬á┬▒┬á1.0
75.0ΓÇô85.0	9.811eΓêÆ04┬á┬▒┬á2.4┬á┬▒┬á0.8┬á┬▒┬á1.8	9.940eΓêÆ04┬á┬▒┬á2.4┬á┬▒┬á0.8┬á┬▒┬á1.8	1.020eΓêÆ03┬á┬▒┬á2.2┬á┬▒┬á1.0┬á┬▒┬á1.5	1.037eΓêÆ03┬á┬▒┬á2.2┬á┬▒┬á1.0┬á┬▒┬á1.5	1.052eΓêÆ03┬á┬▒┬á2.2┬á┬▒┬á1.0┬á┬▒┬á1.6	1.021eΓêÆ03┬á┬▒┬á1.6┬á┬▒┬á0.6┬á┬▒┬á1.1
85.0ΓÇô105.0	5.455eΓêÆ04┬á┬▒┬á2.0┬á┬▒┬á0.6┬á┬▒┬á1.6	5.523eΓêÆ04┬á┬▒┬á2.0┬á┬▒┬á0.6┬á┬▒┬á1.6	5.396eΓêÆ04┬á┬▒┬á1.9┬á┬▒┬á0.7┬á┬▒┬á1.3	5.498eΓêÆ04┬á┬▒┬á1.9┬á┬▒┬á0.7┬á┬▒┬á1.3	5.603eΓêÆ04┬á┬▒┬á1.9┬á┬▒┬á0.7┬á┬▒┬á1.4	5.546eΓêÆ04┬á┬▒┬á1.4┬á┬▒┬á0.5┬á┬▒┬á1.0
105.0ΓÇô150.0	2.015eΓêÆ04┬á┬▒┬á2.1┬á┬▒┬á0.8┬á┬▒┬á1.4	2.045eΓêÆ04┬á┬▒┬á2.1┬á┬▒┬á0.8┬á┬▒┬á1.4	1.935eΓêÆ04┬á┬▒┬á1.9┬á┬▒┬á0.6┬á┬▒┬á1.3	1.975eΓêÆ04┬á┬▒┬á1.9┬á┬▒┬á0.6┬á┬▒┬á1.3	1.993eΓêÆ04┬á┬▒┬á1.9┬á┬▒┬á0.6┬á┬▒┬á1.4	2.005eΓêÆ04┬á┬▒┬á1.4┬á┬▒┬á0.5┬á┬▒┬á0.9
150.0ΓÇô200.0	5.896eΓêÆ05┬á┬▒┬á3.6┬á┬▒┬á1.6┬á┬▒┬á2.6	5.955eΓêÆ05┬á┬▒┬á3.6┬á┬▒┬á1.6┬á┬▒┬á2.6	5.369eΓêÆ05┬á┬▒┬á3.6┬á┬▒┬á1.2┬á┬▒┬á1.6	5.514eΓêÆ05┬á┬▒┬á3.6┬á┬▒┬á1.2┬á┬▒┬á1.6	5.579eΓêÆ05┬á┬▒┬á3.6┬á┬▒┬á1.2┬á┬▒┬á1.7	5.744eΓêÆ05┬á┬▒┬á2.6┬á┬▒┬á1.0┬á┬▒┬á1.5
200.0ΓÇô900.0	1.803eΓêÆ06┬á┬▒┬á5.4┬á┬▒┬á1.8┬á┬▒┬á3.7	1.818eΓêÆ06┬á┬▒┬á5.4┬á┬▒┬á1.8┬á┬▒┬á3.7	1.692eΓêÆ06┬á┬▒┬á5.2┬á┬▒┬á1.7┬á┬▒┬á2.4	1.745eΓêÆ06┬á┬▒┬á5.2┬á┬▒┬á1.7┬á┬▒┬á2.4	1.747eΓêÆ06┬á┬▒┬á5.2┬á┬▒┬á1.7┬á┬▒┬á2.5	1.765eΓêÆ06┬á┬▒┬á3.8┬á┬▒┬á1.2┬á┬▒┬á2.2

**Table 26:** The values of $(1/\sigma )\,d\sigma /dp_\mathrm {T}^{\ell \ell }$ in each bin of $p_\mathrm {T}^{\ell \ell }$ for the electron and muon channels separately (for various generator-level definitions) and for the Born-level combination in the kinematic region $12 \text { GeV} \le m_{\ell \ell }< 20 \text { GeV},\ 0.0 \le |y_{\ell \ell }| < 2.4$. The associated statistical and systematic (both uncorrelated and correlated between bins) are provided in percentage form

Bin	$(1/\sigma )\,d\sigma /dp_\mathrm {T}^{\ell \ell }$ ┬▒ Statistical [%] ┬▒ Uncorrelated systematic [%] ┬▒ Correlated systematic [%]					
	Electron channel		Muon channel			Combination
	Dressed	Born	Bare	Dressed	Born	Born
45.0ΓÇô55.0	2.720eΓêÆ02┬á┬▒┬á1.9┬á┬▒┬á1.4┬á┬▒┬á2.3	2.719eΓêÆ02┬á┬▒┬á1.9┬á┬▒┬á1.4┬á┬▒┬á2.3	2.820eΓêÆ02┬á┬▒┬á1.3┬á┬▒┬á0.3┬á┬▒┬á0.7	2.815eΓêÆ02┬á┬▒┬á1.3┬á┬▒┬á0.3┬á┬▒┬á0.7	2.807eΓêÆ02┬á┬▒┬á1.3┬á┬▒┬á0.3┬á┬▒┬á1.3	2.792eΓêÆ02┬á┬▒┬á1.1┬á┬▒┬á0.4┬á┬▒┬á1.1
55.0ΓÇô65.0	2.137eΓêÆ02┬á┬▒┬á2.3┬á┬▒┬á1.6┬á┬▒┬á1.6	2.140eΓêÆ02┬á┬▒┬á2.3┬á┬▒┬á1.6┬á┬▒┬á1.8	2.166eΓêÆ02┬á┬▒┬á1.6┬á┬▒┬á0.5┬á┬▒┬á0.5	2.156eΓêÆ02┬á┬▒┬á1.6┬á┬▒┬á0.5┬á┬▒┬á0.5	2.158eΓêÆ02┬á┬▒┬á1.6┬á┬▒┬á0.5┬á┬▒┬á0.7	2.150eΓêÆ02┬á┬▒┬á1.3┬á┬▒┬á0.5┬á┬▒┬á0.8
65.0ΓÇô75.0	1.526eΓêÆ02┬á┬▒┬á2.6┬á┬▒┬á2.7┬á┬▒┬á2.9	1.526eΓêÆ02┬á┬▒┬á2.6┬á┬▒┬á2.7┬á┬▒┬á3.0	1.534eΓêÆ02┬á┬▒┬á1.9┬á┬▒┬á0.5┬á┬▒┬á0.6	1.532eΓêÆ02┬á┬▒┬á1.9┬á┬▒┬á0.5┬á┬▒┬á0.6	1.538eΓêÆ02┬á┬▒┬á1.9┬á┬▒┬á0.5┬á┬▒┬á1.3	1.534eΓêÆ02┬á┬▒┬á1.6┬á┬▒┬á0.7┬á┬▒┬á1.2
75.0ΓÇô85.0	1.014eΓêÆ02┬á┬▒┬á3.2┬á┬▒┬á1.4┬á┬▒┬á1.9	1.014eΓêÆ02┬á┬▒┬á3.2┬á┬▒┬á1.4┬á┬▒┬á2.1	1.038eΓêÆ02┬á┬▒┬á2.3┬á┬▒┬á0.7┬á┬▒┬á1.1	1.042eΓêÆ02┬á┬▒┬á2.3┬á┬▒┬á0.7┬á┬▒┬á1.1	1.044eΓêÆ02┬á┬▒┬á2.3┬á┬▒┬á0.7┬á┬▒┬á1.1	1.030eΓêÆ02┬á┬▒┬á1.9┬á┬▒┬á0.7┬á┬▒┬á1.1
85.0ΓÇô105.0	6.223eΓêÆ03┬á┬▒┬á2.5┬á┬▒┬á1.1┬á┬▒┬á2.0	6.239eΓêÆ03┬á┬▒┬á2.5┬á┬▒┬á1.1┬á┬▒┬á2.1	5.958eΓêÆ03┬á┬▒┬á2.1┬á┬▒┬á0.6┬á┬▒┬á1.1	5.974eΓêÆ03┬á┬▒┬á2.1┬á┬▒┬á0.6┬á┬▒┬á1.1	5.974eΓêÆ03┬á┬▒┬á2.1┬á┬▒┬á0.6┬á┬▒┬á1.4	6.042eΓêÆ03┬á┬▒┬á1.6┬á┬▒┬á0.6┬á┬▒┬á1.3
105.0ΓÇô150.0	2.116eΓêÆ03┬á┬▒┬á2.7┬á┬▒┬á1.0┬á┬▒┬á2.2	2.106eΓêÆ03┬á┬▒┬á2.7┬á┬▒┬á1.0┬á┬▒┬á2.3	2.007eΓêÆ03┬á┬▒┬á2.4┬á┬▒┬á0.7┬á┬▒┬á1.4	2.023eΓêÆ03┬á┬▒┬á2.4┬á┬▒┬á0.7┬á┬▒┬á1.4	2.027eΓêÆ03┬á┬▒┬á2.4┬á┬▒┬á0.7┬á┬▒┬á1.4	2.044eΓêÆ03┬á┬▒┬á1.8┬á┬▒┬á0.6┬á┬▒┬á1.3
150.0ΓÇô200.0	5.489eΓêÆ04┬á┬▒┬á4.7┬á┬▒┬á1.9┬á┬▒┬á3.5	5.472eΓêÆ04┬á┬▒┬á4.7┬á┬▒┬á1.9┬á┬▒┬á3.7	4.873eΓêÆ04┬á┬▒┬á4.6┬á┬▒┬á1.7┬á┬▒┬á2.5	4.940eΓêÆ04┬á┬▒┬á4.6┬á┬▒┬á1.7┬á┬▒┬á2.5	4.897eΓêÆ04┬á┬▒┬á4.6┬á┬▒┬á1.7┬á┬▒┬á2.6	5.105eΓêÆ04┬á┬▒┬á3.3┬á┬▒┬á1.2┬á┬▒┬á2.3
200.0ΓÇô900.0	1.888eΓêÆ05┬á┬▒┬á9.6┬á┬▒┬á3.0┬á┬▒┬á4.6	1.880eΓêÆ05┬á┬▒┬á9.6┬á┬▒┬á3.0┬á┬▒┬á4.9	1.467eΓêÆ05┬á┬▒┬á7.1┬á┬▒┬á1.9┬á┬▒┬á3.4	1.460eΓêÆ05┬á┬▒┬á7.1┬á┬▒┬á1.9┬á┬▒┬á3.4	1.448eΓêÆ05┬á┬▒┬á7.1┬á┬▒┬á1.9┬á┬▒┬á4.3	1.574eΓêÆ05┬á┬▒┬á5.7┬á┬▒┬á1.6┬á┬▒┬á3.5

**Table 27:** The values of $(1/\sigma )\,d\sigma /dp_\mathrm {T}^{\ell \ell }$ in each bin of $p_\mathrm {T}^{\ell \ell }$ for the electron and muon channels separately (for various generator-level definitions) and for the Born-level combination in the kinematic region $20 ~\text {GeV} \le m_{\ell \ell }< 30 ~\text {GeV},\ 0.0 \le |y_{\ell \ell }| < 2.4$. The associated statistical and systematic (both uncorrelated and correlated between bins) are provided in percentage form

Bin	$(1/\sigma )\,d\sigma /dp_\mathrm {T}^{\ell \ell }$ ┬▒ Statistical [%] ┬▒ Uncorrelated systematic [%] ┬▒ Correlated systematic [%]					
	Electron channel		Muon channel			Combination
	Dressed	Born	Bare	Dressed	Born	Born
45.0ΓÇô55.0	2.771eΓêÆ02┬á┬▒┬á2.3┬á┬▒┬á0.9┬á┬▒┬á2.2	2.745eΓêÆ02┬á┬▒┬á2.3┬á┬▒┬á0.9┬á┬▒┬á2.4	2.775eΓêÆ02┬á┬▒┬á1.6┬á┬▒┬á0.6┬á┬▒┬á1.1	2.766eΓêÆ02┬á┬▒┬á1.6┬á┬▒┬á0.6┬á┬▒┬á1.1	2.750eΓêÆ02┬á┬▒┬á1.6┬á┬▒┬á0.6┬á┬▒┬á1.4	2.751eΓêÆ02┬á┬▒┬á1.3┬á┬▒┬á0.5┬á┬▒┬á1.3
55.0ΓÇô65.0	2.081eΓêÆ02┬á┬▒┬á2.7┬á┬▒┬á1.4┬á┬▒┬á1.8	2.085eΓêÆ02┬á┬▒┬á2.7┬á┬▒┬á1.4┬á┬▒┬á1.8	2.122eΓêÆ02┬á┬▒┬á1.9┬á┬▒┬á0.7┬á┬▒┬á0.9	2.114eΓêÆ02┬á┬▒┬á1.9┬á┬▒┬á0.7┬á┬▒┬á0.9	2.134eΓêÆ02┬á┬▒┬á1.9┬á┬▒┬á0.7┬á┬▒┬á1.3	2.122eΓêÆ02┬á┬▒┬á1.6┬á┬▒┬á0.6┬á┬▒┬á1.1
65.0ΓÇô75.0	1.441eΓêÆ02┬á┬▒┬á3.1┬á┬▒┬á1.3┬á┬▒┬á2.3	1.447eΓêÆ02┬á┬▒┬á3.1┬á┬▒┬á1.3┬á┬▒┬á2.3	1.475eΓêÆ02┬á┬▒┬á2.4┬á┬▒┬á1.0┬á┬▒┬á1.5	1.480eΓêÆ02┬á┬▒┬á2.4┬á┬▒┬á1.0┬á┬▒┬á1.5	1.479eΓêÆ02┬á┬▒┬á2.4┬á┬▒┬á1.0┬á┬▒┬á1.6	1.468eΓêÆ02┬á┬▒┬á1.9┬á┬▒┬á0.8┬á┬▒┬á1.3
75.0ΓÇô85.0	1.048eΓêÆ02┬á┬▒┬á3.7┬á┬▒┬á1.7┬á┬▒┬á3.1	1.052eΓêÆ02┬á┬▒┬á3.7┬á┬▒┬á1.7┬á┬▒┬á3.1	1.012eΓêÆ02┬á┬▒┬á2.9┬á┬▒┬á1.3┬á┬▒┬á2.0	1.010eΓêÆ02┬á┬▒┬á2.9┬á┬▒┬á1.3┬á┬▒┬á2.0	1.014eΓêÆ02┬á┬▒┬á2.9┬á┬▒┬á1.3┬á┬▒┬á2.0	1.027eΓêÆ02┬á┬▒┬á2.3┬á┬▒┬á1.0┬á┬▒┬á1.7
85.0ΓÇô105.0	6.007eΓêÆ03┬á┬▒┬á3.0┬á┬▒┬á1.4┬á┬▒┬á3.4	6.029eΓêÆ03┬á┬▒┬á3.0┬á┬▒┬á1.4┬á┬▒┬á3.4	6.245eΓêÆ03┬á┬▒┬á2.5┬á┬▒┬á1.2┬á┬▒┬á1.9	6.240eΓêÆ03┬á┬▒┬á2.5┬á┬▒┬á1.2┬á┬▒┬á1.9	6.245eΓêÆ03┬á┬▒┬á2.5┬á┬▒┬á1.2┬á┬▒┬á2.0	6.140eΓêÆ03┬á┬▒┬á1.9┬á┬▒┬á0.9┬á┬▒┬á1.9
105.0ΓÇô150.0	2.236eΓêÆ03┬á┬▒┬á3.0┬á┬▒┬á1.4┬á┬▒┬á3.9	2.247eΓêÆ03┬á┬▒┬á3.0┬á┬▒┬á1.4┬á┬▒┬á3.9	2.176eΓêÆ03┬á┬▒┬á2.7┬á┬▒┬á1.0┬á┬▒┬á2.1	2.201eΓêÆ03┬á┬▒┬á2.7┬á┬▒┬á1.0┬á┬▒┬á2.1	2.191eΓêÆ03┬á┬▒┬á2.7┬á┬▒┬á1.0┬á┬▒┬á2.2	2.199eΓêÆ03┬á┬▒┬á2.0┬á┬▒┬á0.8┬á┬▒┬á2.1
150.0ΓÇô200.0	5.916eΓêÆ04┬á┬▒┬á5.1┬á┬▒┬á2.4┬á┬▒┬á4.8	5.999eΓêÆ04┬á┬▒┬á5.1┬á┬▒┬á2.4┬á┬▒┬á5.6	5.085eΓêÆ04┬á┬▒┬á5.3┬á┬▒┬á2.3┬á┬▒┬á3.2	5.107eΓêÆ04┬á┬▒┬á5.3┬á┬▒┬á2.3┬á┬▒┬á3.2	5.077eΓêÆ04┬á┬▒┬á5.3┬á┬▒┬á2.3┬á┬▒┬á3.4	5.446eΓêÆ04┬á┬▒┬á3.7┬á┬▒┬á1.6┬á┬▒┬á3.2
200.0ΓÇô900.0	2.228eΓêÆ05┬á┬▒┬á7.0┬á┬▒┬á2.8┬á┬▒┬á5.4	2.208eΓêÆ05┬á┬▒┬á7.0┬á┬▒┬á2.8┬á┬▒┬á6.2	1.924eΓêÆ05┬á┬▒┬á6.9┬á┬▒┬á2.7┬á┬▒┬á3.4	1.947eΓêÆ05┬á┬▒┬á6.9┬á┬▒┬á2.7┬á┬▒┬á3.4	1.923eΓêÆ05┬á┬▒┬á6.9┬á┬▒┬á2.7┬á┬▒┬á3.4	2.023eΓêÆ05┬á┬▒┬á5.0┬á┬▒┬á1.9┬á┬▒┬á3.3

**Table 28:** The values of $(1/\sigma )\,d\sigma /dp_\mathrm {T}^{\ell \ell }$ in each bin of $p_\mathrm {T}^{\ell \ell }$ for the electron and muon channels separately (for various generator-level definitions) and for the Born-level combination in the kinematic region $30 \text { GeV} \le m_{\ell \ell }< 46 \text { GeV},\ 0.0 \le |y_{\ell \ell }| < 2.4$. The associated statistical and systematic (both uncorrelated and correlated between bins) are provided in percentage form

Bin	$(1/\sigma )\,d\sigma /dp_\mathrm {T}^{\ell \ell }$ ┬▒ Statistical [%] ┬▒ Uncorrelated systematic [%] ┬▒ Correlated systematic [%]					
	Electron channel		Muon channel			Combination
	Dressed	Born	Bare	Dressed	Born	Born
45.0ΓÇô55.0	2.853eΓêÆ02┬á┬▒┬á2.2┬á┬▒┬á0.6┬á┬▒┬á1.7	2.821eΓêÆ02┬á┬▒┬á2.2┬á┬▒┬á0.6┬á┬▒┬á2.0	2.974eΓêÆ02┬á┬▒┬á1.6┬á┬▒┬á0.5┬á┬▒┬á1.1	2.977eΓêÆ02┬á┬▒┬á1.6┬á┬▒┬á0.5┬á┬▒┬á1.1	2.953eΓêÆ02┬á┬▒┬á1.6┬á┬▒┬á0.5┬á┬▒┬á1.3	2.909eΓêÆ02┬á┬▒┬á1.3┬á┬▒┬á0.4┬á┬▒┬á1.2
55.0ΓÇô65.0	2.153eΓêÆ02┬á┬▒┬á2.6┬á┬▒┬á1.4┬á┬▒┬á1.4	2.165eΓêÆ02┬á┬▒┬á2.7┬á┬▒┬á1.4┬á┬▒┬á1.6	2.118eΓêÆ02┬á┬▒┬á2.0┬á┬▒┬á0.8┬á┬▒┬á1.0	2.119eΓêÆ02┬á┬▒┬á2.0┬á┬▒┬á0.8┬á┬▒┬á1.0	2.132eΓêÆ02┬á┬▒┬á2.0┬á┬▒┬á0.8┬á┬▒┬á1.3	2.146eΓêÆ02┬á┬▒┬á1.6┬á┬▒┬á0.7┬á┬▒┬á1.1
65.0ΓÇô75.0	1.517eΓêÆ02┬á┬▒┬á3.3┬á┬▒┬á1.1┬á┬▒┬á1.6	1.529eΓêÆ02┬á┬▒┬á3.3┬á┬▒┬á1.1┬á┬▒┬á1.7	1.412eΓêÆ02┬á┬▒┬á2.6┬á┬▒┬á1.1┬á┬▒┬á1.3	1.405eΓêÆ02┬á┬▒┬á2.6┬á┬▒┬á1.1┬á┬▒┬á1.3	1.410eΓêÆ02┬á┬▒┬á2.6┬á┬▒┬á1.1┬á┬▒┬á1.3	1.452eΓêÆ02┬á┬▒┬á2.1┬á┬▒┬á0.8┬á┬▒┬á1.1
75.0ΓÇô85.0	9.428eΓêÆ03┬á┬▒┬á4.2┬á┬▒┬á1.5┬á┬▒┬á2.3	9.462eΓêÆ03┬á┬▒┬á4.2┬á┬▒┬á1.5┬á┬▒┬á2.3	1.001eΓêÆ02┬á┬▒┬á3.2┬á┬▒┬á1.2┬á┬▒┬á1.6	9.895eΓêÆ03┬á┬▒┬á3.2┬á┬▒┬á1.2┬á┬▒┬á1.6	9.917eΓêÆ03┬á┬▒┬á3.2┬á┬▒┬á1.2┬á┬▒┬á2.8	9.763eΓêÆ03┬á┬▒┬á2.6┬á┬▒┬á0.9┬á┬▒┬á2.0
85.0ΓÇô105.0	5.793eΓêÆ03┬á┬▒┬á3.4┬á┬▒┬á1.4┬á┬▒┬á2.5	5.807eΓêÆ03┬á┬▒┬á3.4┬á┬▒┬á1.4┬á┬▒┬á2.6	5.772eΓêÆ03┬á┬▒┬á2.9┬á┬▒┬á1.2┬á┬▒┬á1.8	5.799eΓêÆ03┬á┬▒┬á2.9┬á┬▒┬á1.2┬á┬▒┬á1.8	5.808eΓêÆ03┬á┬▒┬á2.9┬á┬▒┬á1.2┬á┬▒┬á2.0	5.793eΓêÆ03┬á┬▒┬á2.2┬á┬▒┬á0.9┬á┬▒┬á1.7
105.0ΓÇô150.0	2.114eΓêÆ03┬á┬▒┬á3.3┬á┬▒┬á1.5┬á┬▒┬á3.0	2.118eΓêÆ03┬á┬▒┬á3.3┬á┬▒┬á1.5┬á┬▒┬á3.0	2.028eΓêÆ03┬á┬▒┬á3.0┬á┬▒┬á1.1┬á┬▒┬á2.4	2.042eΓêÆ03┬á┬▒┬á3.0┬á┬▒┬á1.1┬á┬▒┬á2.4	2.053eΓêÆ03┬á┬▒┬á3.0┬á┬▒┬á1.1┬á┬▒┬á2.4	2.076eΓêÆ03┬á┬▒┬á2.2┬á┬▒┬á0.9┬á┬▒┬á1.9
150.0ΓÇô200.0	5.464eΓêÆ04┬á┬▒┬á5.6┬á┬▒┬á1.7┬á┬▒┬á3.6	5.430eΓêÆ04┬á┬▒┬á5.6┬á┬▒┬á1.7┬á┬▒┬á4.1	5.930eΓêÆ04┬á┬▒┬á5.2┬á┬▒┬á2.3┬á┬▒┬á4.3	5.911eΓêÆ04┬á┬▒┬á5.2┬á┬▒┬á2.3┬á┬▒┬á4.3	5.853eΓêÆ04┬á┬▒┬á5.2┬á┬▒┬á2.3┬á┬▒┬á4.6	5.617eΓêÆ04┬á┬▒┬á3.8┬á┬▒┬á1.4┬á┬▒┬á3.5
200.0ΓÇô900.0	2.155eΓêÆ05┬á┬▒┬á7.7┬á┬▒┬á5.1┬á┬▒┬á6.0	2.182eΓêÆ05┬á┬▒┬á7.7┬á┬▒┬á5.1┬á┬▒┬á6.1	1.887eΓêÆ05┬á┬▒┬á7.7┬á┬▒┬á3.4┬á┬▒┬á7.4	1.923eΓêÆ05┬á┬▒┬á7.7┬á┬▒┬á3.4┬á┬▒┬á7.4	1.926eΓêÆ05┬á┬▒┬á7.7┬á┬▒┬á3.4┬á┬▒┬á7.5	2.037eΓêÆ05┬á┬▒┬á5.4┬á┬▒┬á2.9┬á┬▒┬á5.0

**Table 29:** The values of $(1/\sigma )\,d\sigma /dp_\mathrm {T}^{\ell \ell }$ in each bin of $p_\mathrm {T}^{\ell \ell }$ for the electron and muon channels separately (for various generator-level definitions) and for the Born-level combination in the kinematic region $46 \text { GeV} \le m_{\ell \ell }< 66 \text { GeV},\ 0.0 \le |y_{\ell \ell }| < 2.4$. The associated statistical and systematic (both uncorrelated and correlated between bins) are provided in percentage form

Bin	$(1/\sigma )\,d\sigma /dp_\mathrm {T}^{\ell \ell }$ ┬▒ Statistical [%] ┬▒ Uncorrelated systematic [%] ┬▒ Correlated systematic [%]					
	Electron channel		Muon channel			Combination
	Dressed	Born	Bare	Dressed	Born	Born
0.0ΓÇô2.0	3.886eΓêÆ02┬á┬▒┬á1.4┬á┬▒┬á0.7┬á┬▒┬á4.5	4.361eΓêÆ02┬á┬▒┬á1.4┬á┬▒┬á0.7┬á┬▒┬á5.2	3.680eΓêÆ02┬á┬▒┬á1.0┬á┬▒┬á0.3┬á┬▒┬á4.4	3.927eΓêÆ02┬á┬▒┬á1.0┬á┬▒┬á0.3┬á┬▒┬á4.4	4.420eΓêÆ02┬á┬▒┬á1.0┬á┬▒┬á0.3┬á┬▒┬á4.8	4.347eΓêÆ02┬á┬▒┬á0.8┬á┬▒┬á0.3┬á┬▒┬á4.4
2.0ΓÇô4.0	7.415eΓêÆ02┬á┬▒┬á1.0┬á┬▒┬á0.5┬á┬▒┬á1.3	8.265eΓêÆ02┬á┬▒┬á1.0┬á┬▒┬á0.5┬á┬▒┬á3.1	6.865eΓêÆ02┬á┬▒┬á0.7┬á┬▒┬á0.3┬á┬▒┬á0.8	7.302eΓêÆ02┬á┬▒┬á0.7┬á┬▒┬á0.3┬á┬▒┬á0.8	8.110eΓêÆ02┬á┬▒┬á0.7┬á┬▒┬á0.3┬á┬▒┬á2.1	8.148eΓêÆ02┬á┬▒┬á0.6┬á┬▒┬á0.3┬á┬▒┬á1.2
4.0ΓÇô6.0	6.674eΓêÆ02┬á┬▒┬á1.0┬á┬▒┬á0.5┬á┬▒┬á1.1	7.307eΓêÆ02┬á┬▒┬á1.0┬á┬▒┬á0.5┬á┬▒┬á2.7	6.451eΓêÆ02┬á┬▒┬á0.7┬á┬▒┬á0.3┬á┬▒┬á0.7	6.764eΓêÆ02┬á┬▒┬á0.7┬á┬▒┬á0.3┬á┬▒┬á0.7	7.414eΓêÆ02┬á┬▒┬á0.7┬á┬▒┬á0.3┬á┬▒┬á1.8	7.390eΓêÆ02┬á┬▒┬á0.6┬á┬▒┬á0.2┬á┬▒┬á1.1
6.0ΓÇô8.0	5.417eΓêÆ02┬á┬▒┬á1.1┬á┬▒┬á0.5┬á┬▒┬á0.9	5.822eΓêÆ02┬á┬▒┬á1.1┬á┬▒┬á0.5┬á┬▒┬á2.0	5.296eΓêÆ02┬á┬▒┬á0.8┬á┬▒┬á0.3┬á┬▒┬á0.8	5.484eΓêÆ02┬á┬▒┬á0.8┬á┬▒┬á0.3┬á┬▒┬á0.8	5.885eΓêÆ02┬á┬▒┬á0.8┬á┬▒┬á0.3┬á┬▒┬á1.5	5.877eΓêÆ02┬á┬▒┬á0.6┬á┬▒┬á0.2┬á┬▒┬á0.9
8.0ΓÇô10.0	4.119eΓêÆ02┬á┬▒┬á1.3┬á┬▒┬á0.5┬á┬▒┬á1.1	4.293eΓêÆ02┬á┬▒┬á1.3┬á┬▒┬á0.5┬á┬▒┬á1.6	4.097eΓêÆ02┬á┬▒┬á0.9┬á┬▒┬á0.4┬á┬▒┬á0.8	4.163eΓêÆ02┬á┬▒┬á0.9┬á┬▒┬á0.4┬á┬▒┬á0.8	4.361eΓêÆ02┬á┬▒┬á0.9┬á┬▒┬á0.4┬á┬▒┬á1.3	4.355eΓêÆ02┬á┬▒┬á0.8┬á┬▒┬á0.3┬á┬▒┬á0.9
10.0ΓÇô13.0	3.160eΓêÆ02┬á┬▒┬á1.1┬á┬▒┬á0.4┬á┬▒┬á1.4	3.175eΓêÆ02┬á┬▒┬á1.1┬á┬▒┬á0.4┬á┬▒┬á2.0	3.180eΓêÆ02┬á┬▒┬á0.8┬á┬▒┬á0.3┬á┬▒┬á0.9	3.166eΓêÆ02┬á┬▒┬á0.8┬á┬▒┬á0.3┬á┬▒┬á0.9	3.179eΓêÆ02┬á┬▒┬á0.8┬á┬▒┬á0.3┬á┬▒┬á1.3	3.187eΓêÆ02┬á┬▒┬á0.6┬á┬▒┬á0.2┬á┬▒┬á1.0
13.0ΓÇô16.0	2.289eΓêÆ02┬á┬▒┬á1.3┬á┬▒┬á0.5┬á┬▒┬á1.5	2.152eΓêÆ02┬á┬▒┬á1.3┬á┬▒┬á0.5┬á┬▒┬á1.9	2.374eΓêÆ02┬á┬▒┬á0.9┬á┬▒┬á0.3┬á┬▒┬á0.9	2.283eΓêÆ02┬á┬▒┬á0.9┬á┬▒┬á0.3┬á┬▒┬á0.9	2.137eΓêÆ02┬á┬▒┬á0.9┬á┬▒┬á0.3┬á┬▒┬á1.6	2.148eΓêÆ02┬á┬▒┬á0.7┬á┬▒┬á0.3┬á┬▒┬á1.0
16.0ΓÇô20.0	1.705eΓêÆ02┬á┬▒┬á1.3┬á┬▒┬á0.5┬á┬▒┬á1.3	1.438eΓêÆ02┬á┬▒┬á1.3┬á┬▒┬á0.5┬á┬▒┬á2.2	1.904eΓêÆ02┬á┬▒┬á0.9┬á┬▒┬á0.3┬á┬▒┬á0.9	1.738eΓêÆ02┬á┬▒┬á0.9┬á┬▒┬á0.3┬á┬▒┬á0.9	1.462eΓêÆ02┬á┬▒┬á0.9┬á┬▒┬á0.3┬á┬▒┬á2.0	1.456eΓêÆ02┬á┬▒┬á0.7┬á┬▒┬á0.2┬á┬▒┬á1.2
20.0ΓÇô25.0	1.254eΓêÆ02┬á┬▒┬á1.2┬á┬▒┬á0.5┬á┬▒┬á1.2	9.316eΓêÆ03┬á┬▒┬á1.2┬á┬▒┬á0.5┬á┬▒┬á1.7	1.432eΓêÆ02┬á┬▒┬á0.8┬á┬▒┬á0.4┬á┬▒┬á1.1	1.254eΓêÆ02┬á┬▒┬á0.8┬á┬▒┬á0.4┬á┬▒┬á1.1	9.367eΓêÆ03┬á┬▒┬á0.8┬á┬▒┬á0.4┬á┬▒┬á3.9	9.327eΓêÆ03┬á┬▒┬á0.7┬á┬▒┬á0.3┬á┬▒┬á1.7
25.0ΓÇô30.0	7.907eΓêÆ03┬á┬▒┬á1.5┬á┬▒┬á0.7┬á┬▒┬á1.5	6.033eΓêÆ03┬á┬▒┬á1.5┬á┬▒┬á0.7┬á┬▒┬á1.7	8.560eΓêÆ03┬á┬▒┬á1.1┬á┬▒┬á0.5┬á┬▒┬á1.2	7.862eΓêÆ03┬á┬▒┬á1.1┬á┬▒┬á0.5┬á┬▒┬á1.2	5.972eΓêÆ03┬á┬▒┬á1.1┬á┬▒┬á0.5┬á┬▒┬á1.5	5.999eΓêÆ03┬á┬▒┬á0.9┬á┬▒┬á0.4┬á┬▒┬á1.1
30.0ΓÇô37.0	4.606eΓêÆ03┬á┬▒┬á1.6┬á┬▒┬á0.8┬á┬▒┬á1.8	3.857eΓêÆ03┬á┬▒┬á1.6┬á┬▒┬á0.8┬á┬▒┬á2.6	4.580eΓêÆ03┬á┬▒┬á1.2┬á┬▒┬á0.6┬á┬▒┬á1.5	4.428eΓêÆ03┬á┬▒┬á1.2┬á┬▒┬á0.6┬á┬▒┬á1.5	3.711eΓêÆ03┬á┬▒┬á1.2┬á┬▒┬á0.6┬á┬▒┬á2.4	3.768eΓêÆ03┬á┬▒┬á1.0┬á┬▒┬á0.5┬á┬▒┬á1.5
37.0ΓÇô45.0	2.797eΓêÆ03┬á┬▒┬á2.0┬á┬▒┬á1.0┬á┬▒┬á2.1	2.515eΓêÆ03┬á┬▒┬á2.0┬á┬▒┬á1.0┬á┬▒┬á2.5	2.844eΓêÆ03┬á┬▒┬á1.5┬á┬▒┬á0.7┬á┬▒┬á2.0	2.784eΓêÆ03┬á┬▒┬á1.5┬á┬▒┬á0.7┬á┬▒┬á2.0	2.504eΓêÆ03┬á┬▒┬á1.5┬á┬▒┬á0.7┬á┬▒┬á2.4	2.520eΓêÆ03┬á┬▒┬á1.2┬á┬▒┬á0.6┬á┬▒┬á1.7
45.0ΓÇô55.0	1.915eΓêÆ03┬á┬▒┬á2.1┬á┬▒┬á1.0┬á┬▒┬á2.3	1.781eΓêÆ03┬á┬▒┬á2.1┬á┬▒┬á1.0┬á┬▒┬á2.7	1.859eΓêÆ03┬á┬▒┬á1.6┬á┬▒┬á0.6┬á┬▒┬á2.0	1.819eΓêÆ03┬á┬▒┬á1.6┬á┬▒┬á0.6┬á┬▒┬á2.0	1.670eΓêÆ03┬á┬▒┬á1.6┬á┬▒┬á0.6┬á┬▒┬á3.3	1.712eΓêÆ03┬á┬▒┬á1.3┬á┬▒┬á0.5┬á┬▒┬á1.9
55.0ΓÇô65.0	1.213eΓêÆ03┬á┬▒┬á2.8┬á┬▒┬á1.2┬á┬▒┬á2.8	1.139eΓêÆ03┬á┬▒┬á2.8┬á┬▒┬á1.2┬á┬▒┬á3.1	1.262eΓêÆ03┬á┬▒┬á2.2┬á┬▒┬á0.8┬á┬▒┬á2.1	1.236eΓêÆ03┬á┬▒┬á2.2┬á┬▒┬á0.8┬á┬▒┬á2.1	1.152eΓêÆ03┬á┬▒┬á2.2┬á┬▒┬á0.8┬á┬▒┬á3.1	1.146eΓêÆ03┬á┬▒┬á1.7┬á┬▒┬á0.7┬á┬▒┬á2.1
65.0ΓÇô75.0	8.436eΓêÆ04┬á┬▒┬á3.4┬á┬▒┬á1.5┬á┬▒┬á3.4	7.991eΓêÆ04┬á┬▒┬á3.4┬á┬▒┬á1.5┬á┬▒┬á3.5	8.045eΓêÆ04┬á┬▒┬á2.8┬á┬▒┬á1.0┬á┬▒┬á2.2	7.838eΓêÆ04┬á┬▒┬á2.8┬á┬▒┬á1.0┬á┬▒┬á2.2	7.582eΓêÆ04┬á┬▒┬á2.8┬á┬▒┬á1.0┬á┬▒┬á2.2	7.712eΓêÆ04┬á┬▒┬á2.2┬á┬▒┬á0.8┬á┬▒┬á2.1
75.0ΓÇô85.0	6.112eΓêÆ04┬á┬▒┬á4.0┬á┬▒┬á1.8┬á┬▒┬á3.8	5.812eΓêÆ04┬á┬▒┬á4.0┬á┬▒┬á1.8┬á┬▒┬á4.4	5.686eΓêÆ04┬á┬▒┬á3.4┬á┬▒┬á1.3┬á┬▒┬á2.5	5.533eΓêÆ04┬á┬▒┬á3.4┬á┬▒┬á1.3┬á┬▒┬á2.5	5.292eΓêÆ04┬á┬▒┬á3.4┬á┬▒┬á1.3┬á┬▒┬á2.9	5.473eΓêÆ04┬á┬▒┬á2.6┬á┬▒┬á1.1┬á┬▒┬á2.5
85.0ΓÇô105.0	3.534eΓêÆ04┬á┬▒┬á3.4┬á┬▒┬á0.9┬á┬▒┬á3.8	3.374eΓêÆ04┬á┬▒┬á3.4┬á┬▒┬á0.9┬á┬▒┬á4.0	3.460eΓêÆ04┬á┬▒┬á2.9┬á┬▒┬á1.0┬á┬▒┬á2.4	3.396eΓêÆ04┬á┬▒┬á2.9┬á┬▒┬á1.0┬á┬▒┬á2.4	3.246eΓêÆ04┬á┬▒┬á2.9┬á┬▒┬á1.0┬á┬▒┬á3.0	3.267eΓêÆ04┬á┬▒┬á2.2┬á┬▒┬á0.7┬á┬▒┬á2.5
105.0ΓÇô150.0	1.279eΓêÆ04┬á┬▒┬á3.5┬á┬▒┬á1.3┬á┬▒┬á4.3	1.225eΓêÆ04┬á┬▒┬á3.5┬á┬▒┬á1.3┬á┬▒┬á4.8	1.250eΓêÆ04┬á┬▒┬á3.1┬á┬▒┬á1.0┬á┬▒┬á2.1	1.229eΓêÆ04┬á┬▒┬á3.1┬á┬▒┬á1.0┬á┬▒┬á2.1	1.193eΓêÆ04┬á┬▒┬á3.1┬á┬▒┬á1.0┬á┬▒┬á2.3	1.191eΓêÆ04┬á┬▒┬á2.4┬á┬▒┬á0.8┬á┬▒┬á2.3
150.0ΓÇô200.0	3.507eΓêÆ05┬á┬▒┬á5.6┬á┬▒┬á1.6┬á┬▒┬á4.4	3.436eΓêÆ05┬á┬▒┬á5.6┬á┬▒┬á1.6┬á┬▒┬á4.4	3.548eΓêÆ05┬á┬▒┬á5.1┬á┬▒┬á1.8┬á┬▒┬á2.4	3.488eΓêÆ05┬á┬▒┬á5.1┬á┬▒┬á1.8┬á┬▒┬á2.4	3.385eΓêÆ05┬á┬▒┬á5.1┬á┬▒┬á1.8┬á┬▒┬á3.3	3.363eΓêÆ05┬á┬▒┬á3.8┬á┬▒┬á1.2┬á┬▒┬á2.3
200.0ΓÇô900.0	1.279eΓêÆ06┬á┬▒┬á7.5┬á┬▒┬á2.3┬á┬▒┬á6.0	1.240eΓêÆ06┬á┬▒┬á7.5┬á┬▒┬á2.3┬á┬▒┬á6.1	1.361eΓêÆ06┬á┬▒┬á6.7┬á┬▒┬á2.6┬á┬▒┬á3.2	1.315eΓêÆ06┬á┬▒┬á6.7┬á┬▒┬á2.6┬á┬▒┬á3.2	1.294eΓêÆ06┬á┬▒┬á6.7┬á┬▒┬á2.6┬á┬▒┬á3.8	1.247eΓêÆ06┬á┬▒┬á5.1┬á┬▒┬á1.8┬á┬▒┬á3.0

**Table 30:** The values of $(1/\sigma )\,d\sigma /dp_\mathrm {T}^{\ell \ell }$ in each bin of $p_\mathrm {T}^{\ell \ell }$ for the electron and muon channels separately (for various generator-level definitions) and for the Born-level combination in the kinematic region $66 ~\text {GeV} \le m_{\ell \ell }< 116 ~\text {GeV},\ 0.0 \le |y_{\ell \ell }| < 2.4$. The associated statistical and systematic (both uncorrelated and correlated between bins) are provided in percentage form

Bin	$(1/\sigma )\,d\sigma /dp_\mathrm {T}^{\ell \ell }$ ┬▒ Statistical [%] ┬▒ Uncorrelated systematic [%] ┬▒ Correlated systematic [%]					
	Electron channel		Muon channel			Combination
	Dressed	Born	Bare	Dressed	Born	Born
0.0ΓÇô2.0	2.608eΓêÆ02┬á┬▒┬á0.3┬á┬▒┬á0.1┬á┬▒┬á0.9	2.677eΓêÆ02┬á┬▒┬á0.3┬á┬▒┬á0.1┬á┬▒┬á0.9	2.568eΓêÆ02┬á┬▒┬á0.2┬á┬▒┬á0.1┬á┬▒┬á0.9	2.626eΓêÆ02┬á┬▒┬á0.2┬á┬▒┬á0.1┬á┬▒┬á0.9	2.696eΓêÆ02┬á┬▒┬á0.2┬á┬▒┬á0.1┬á┬▒┬á0.9	2.669eΓêÆ02┬á┬▒┬á0.2┬á┬▒┬á0.1┬á┬▒┬á0.5
2.0ΓÇô4.0	5.441eΓêÆ02┬á┬▒┬á0.2┬á┬▒┬á0.1┬á┬▒┬á0.5	5.553eΓêÆ02┬á┬▒┬á0.2┬á┬▒┬á0.1┬á┬▒┬á0.6	5.337eΓêÆ02┬á┬▒┬á0.1┬á┬▒┬á0.1┬á┬▒┬á0.3	5.428eΓêÆ02┬á┬▒┬á0.1┬á┬▒┬á0.1┬á┬▒┬á0.3	5.541eΓêÆ02┬á┬▒┬á0.2┬á┬▒┬á0.1┬á┬▒┬á0.4	5.545eΓêÆ02┬á┬▒┬á0.1┬á┬▒┬á0.1┬á┬▒┬á0.2
4.0ΓÇô6.0	5.505eΓêÆ02┬á┬▒┬á0.2┬á┬▒┬á0.1┬á┬▒┬á0.3	5.560eΓêÆ02┬á┬▒┬á0.2┬á┬▒┬á0.1┬á┬▒┬á0.3	5.483eΓêÆ02┬á┬▒┬á0.1┬á┬▒┬á0.1┬á┬▒┬á0.4	5.525eΓêÆ02┬á┬▒┬á0.1┬á┬▒┬á0.1┬á┬▒┬á0.4	5.579eΓêÆ02┬á┬▒┬á0.1┬á┬▒┬á0.1┬á┬▒┬á0.5	5.590eΓêÆ02┬á┬▒┬á0.1┬á┬▒┬á0.1┬á┬▒┬á0.3
6.0ΓÇô8.0	4.831eΓêÆ02┬á┬▒┬á0.2┬á┬▒┬á0.1┬á┬▒┬á0.4	4.831eΓêÆ02┬á┬▒┬á0.2┬á┬▒┬á0.1┬á┬▒┬á0.5	4.856eΓêÆ02┬á┬▒┬á0.2┬á┬▒┬á0.1┬á┬▒┬á0.2	4.851eΓêÆ02┬á┬▒┬á0.2┬á┬▒┬á0.1┬á┬▒┬á0.2	4.851eΓêÆ02┬á┬▒┬á0.2┬á┬▒┬á0.1┬á┬▒┬á0.6	4.847eΓêÆ02┬á┬▒┬á0.1┬á┬▒┬á0.1┬á┬▒┬á0.3
8.0ΓÇô10.0	4.084eΓêÆ02┬á┬▒┬á0.2┬á┬▒┬á0.1┬á┬▒┬á0.4	4.052eΓêÆ02┬á┬▒┬á0.2┬á┬▒┬á0.1┬á┬▒┬á0.5	4.115eΓêÆ02┬á┬▒┬á0.2┬á┬▒┬á0.1┬á┬▒┬á0.2	4.085eΓêÆ02┬á┬▒┬á0.2┬á┬▒┬á0.1┬á┬▒┬á0.2	4.055eΓêÆ02┬á┬▒┬á0.2┬á┬▒┬á0.1┬á┬▒┬á0.6	4.047eΓêÆ02┬á┬▒┬á0.1┬á┬▒┬á0.1┬á┬▒┬á0.3
10.0ΓÇô12.0	3.429eΓêÆ02┬á┬▒┬á0.2┬á┬▒┬á0.1┬á┬▒┬á0.2	3.389eΓêÆ02┬á┬▒┬á0.2┬á┬▒┬á0.1┬á┬▒┬á0.3	3.458eΓêÆ02┬á┬▒┬á0.2┬á┬▒┬á0.1┬á┬▒┬á0.2	3.420eΓêÆ02┬á┬▒┬á0.2┬á┬▒┬á0.1┬á┬▒┬á0.2	3.378eΓêÆ02┬á┬▒┬á0.2┬á┬▒┬á0.1┬á┬▒┬á0.4	3.384eΓêÆ02┬á┬▒┬á0.1┬á┬▒┬á0.1┬á┬▒┬á0.2
12.0ΓÇô14.0	2.881eΓêÆ02┬á┬▒┬á0.2┬á┬▒┬á0.1┬á┬▒┬á0.3	2.838eΓêÆ02┬á┬▒┬á0.2┬á┬▒┬á0.1┬á┬▒┬á0.3	2.919eΓêÆ02┬á┬▒┬á0.2┬á┬▒┬á0.1┬á┬▒┬á0.2	2.878eΓêÆ02┬á┬▒┬á0.2┬á┬▒┬á0.1┬á┬▒┬á0.2	2.835eΓêÆ02┬á┬▒┬á0.2┬á┬▒┬á0.1┬á┬▒┬á0.3	2.838eΓêÆ02┬á┬▒┬á0.2┬á┬▒┬á0.1┬á┬▒┬á0.2
14.0ΓÇô16.0	2.431eΓêÆ02┬á┬▒┬á0.3┬á┬▒┬á0.1┬á┬▒┬á0.3	2.393eΓêÆ02┬á┬▒┬á0.3┬á┬▒┬á0.1┬á┬▒┬á0.3	2.479eΓêÆ02┬á┬▒┬á0.2┬á┬▒┬á0.1┬á┬▒┬á0.2	2.443eΓêÆ02┬á┬▒┬á0.2┬á┬▒┬á0.1┬á┬▒┬á0.2	2.403eΓêÆ02┬á┬▒┬á0.2┬á┬▒┬á0.1┬á┬▒┬á0.2	2.404eΓêÆ02┬á┬▒┬á0.2┬á┬▒┬á0.1┬á┬▒┬á0.1
16.0ΓÇô18.0	2.076eΓêÆ02┬á┬▒┬á0.3┬á┬▒┬á0.1┬á┬▒┬á0.2	2.041eΓêÆ02┬á┬▒┬á0.3┬á┬▒┬á0.1┬á┬▒┬á0.2	2.109eΓêÆ02┬á┬▒┬á0.2┬á┬▒┬á0.1┬á┬▒┬á0.2	2.077eΓêÆ02┬á┬▒┬á0.2┬á┬▒┬á0.1┬á┬▒┬á0.2	2.042eΓêÆ02┬á┬▒┬á0.2┬á┬▒┬á0.1┬á┬▒┬á0.2	2.043eΓêÆ02┬á┬▒┬á0.2┬á┬▒┬á0.1┬á┬▒┬á0.1
18.0ΓÇô20.0	1.782eΓêÆ02┬á┬▒┬á0.3┬á┬▒┬á0.2┬á┬▒┬á0.3	1.755eΓêÆ02┬á┬▒┬á0.3┬á┬▒┬á0.2┬á┬▒┬á0.3	1.805eΓêÆ02┬á┬▒┬á0.3┬á┬▒┬á0.1┬á┬▒┬á0.2	1.781eΓêÆ02┬á┬▒┬á0.3┬á┬▒┬á0.1┬á┬▒┬á0.2	1.754eΓêÆ02┬á┬▒┬á0.3┬á┬▒┬á0.1┬á┬▒┬á0.2	1.756eΓêÆ02┬á┬▒┬á0.2┬á┬▒┬á0.1┬á┬▒┬á0.2
20.0ΓÇô22.5	1.510eΓêÆ02┬á┬▒┬á0.3┬á┬▒┬á0.1┬á┬▒┬á0.3	1.490eΓêÆ02┬á┬▒┬á0.3┬á┬▒┬á0.1┬á┬▒┬á0.3	1.520eΓêÆ02┬á┬▒┬á0.3┬á┬▒┬á0.1┬á┬▒┬á0.3	1.503eΓêÆ02┬á┬▒┬á0.3┬á┬▒┬á0.1┬á┬▒┬á0.3	1.485eΓêÆ02┬á┬▒┬á0.3┬á┬▒┬á0.1┬á┬▒┬á0.3	1.488eΓêÆ02┬á┬▒┬á0.2┬á┬▒┬á0.1┬á┬▒┬á0.2
22.5ΓÇô25.0	1.259eΓêÆ02┬á┬▒┬á0.3┬á┬▒┬á0.2┬á┬▒┬á0.4	1.247eΓêÆ02┬á┬▒┬á0.3┬á┬▒┬á0.2┬á┬▒┬á0.4	1.275eΓêÆ02┬á┬▒┬á0.3┬á┬▒┬á0.1┬á┬▒┬á0.2	1.266eΓêÆ02┬á┬▒┬á0.3┬á┬▒┬á0.1┬á┬▒┬á0.2	1.253eΓêÆ02┬á┬▒┬á0.3┬á┬▒┬á0.1┬á┬▒┬á0.2	1.251eΓêÆ02┬á┬▒┬á0.2┬á┬▒┬á0.1┬á┬▒┬á0.2
25.0ΓÇô27.5	1.072eΓêÆ02┬á┬▒┬á0.4┬á┬▒┬á0.2┬á┬▒┬á0.4	1.065eΓêÆ02┬á┬▒┬á0.4┬á┬▒┬á0.2┬á┬▒┬á0.4	1.074eΓêÆ02┬á┬▒┬á0.3┬á┬▒┬á0.1┬á┬▒┬á0.3	1.068eΓêÆ02┬á┬▒┬á0.3┬á┬▒┬á0.1┬á┬▒┬á0.3	1.061eΓêÆ02┬á┬▒┬á0.3┬á┬▒┬á0.1┬á┬▒┬á0.3	1.062eΓêÆ02┬á┬▒┬á0.2┬á┬▒┬á0.1┬á┬▒┬á0.2
27.5ΓÇô30.0	9.172eΓêÆ03┬á┬▒┬á0.4┬á┬▒┬á0.2┬á┬▒┬á0.5	9.124eΓêÆ03┬á┬▒┬á0.4┬á┬▒┬á0.2┬á┬▒┬á0.5	9.089eΓêÆ03┬á┬▒┬á0.3┬á┬▒┬á0.1┬á┬▒┬á0.3	9.064eΓêÆ03┬á┬▒┬á0.3┬á┬▒┬á0.1┬á┬▒┬á0.3	9.014eΓêÆ03┬á┬▒┬á0.3┬á┬▒┬á0.1┬á┬▒┬á0.3	9.062eΓêÆ03┬á┬▒┬á0.3┬á┬▒┬á0.1┬á┬▒┬á0.2
30.0ΓÇô33.0	7.677eΓêÆ03┬á┬▒┬á0.4┬á┬▒┬á0.2┬á┬▒┬á0.4	7.657eΓêÆ03┬á┬▒┬á0.4┬á┬▒┬á0.2┬á┬▒┬á0.4	7.721eΓêÆ03┬á┬▒┬á0.3┬á┬▒┬á0.1┬á┬▒┬á0.3	7.709eΓêÆ03┬á┬▒┬á0.3┬á┬▒┬á0.1┬á┬▒┬á0.3	7.687eΓêÆ03┬á┬▒┬á0.3┬á┬▒┬á0.1┬á┬▒┬á0.3	7.677eΓêÆ03┬á┬▒┬á0.2┬á┬▒┬á0.1┬á┬▒┬á0.2
33.0ΓÇô36.0	6.558eΓêÆ03┬á┬▒┬á0.4┬á┬▒┬á0.2┬á┬▒┬á0.5	6.552eΓêÆ03┬á┬▒┬á0.4┬á┬▒┬á0.2┬á┬▒┬á0.5	6.536eΓêÆ03┬á┬▒┬á0.3┬á┬▒┬á0.2┬á┬▒┬á0.3	6.541eΓêÆ03┬á┬▒┬á0.3┬á┬▒┬á0.2┬á┬▒┬á0.3	6.536eΓêÆ03┬á┬▒┬á0.3┬á┬▒┬á0.2┬á┬▒┬á0.4	6.534eΓêÆ03┬á┬▒┬á0.3┬á┬▒┬á0.1┬á┬▒┬á0.2
36.0ΓÇô39.0	5.537eΓêÆ03┬á┬▒┬á0.4┬á┬▒┬á0.2┬á┬▒┬á0.6	5.536eΓêÆ03┬á┬▒┬á0.4┬á┬▒┬á0.2┬á┬▒┬á0.6	5.476eΓêÆ03┬á┬▒┬á0.4┬á┬▒┬á0.2┬á┬▒┬á0.3	5.487eΓêÆ03┬á┬▒┬á0.4┬á┬▒┬á0.2┬á┬▒┬á0.3	5.491eΓêÆ03┬á┬▒┬á0.4┬á┬▒┬á0.2┬á┬▒┬á0.3	5.507eΓêÆ03┬á┬▒┬á0.3┬á┬▒┬á0.1┬á┬▒┬á0.2
39.0ΓÇô42.0	4.745eΓêÆ03┬á┬▒┬á0.5┬á┬▒┬á0.2┬á┬▒┬á0.6	4.754eΓêÆ03┬á┬▒┬á0.5┬á┬▒┬á0.2┬á┬▒┬á0.6	4.712eΓêÆ03┬á┬▒┬á0.4┬á┬▒┬á0.2┬á┬▒┬á0.3	4.728eΓêÆ03┬á┬▒┬á0.4┬á┬▒┬á0.2┬á┬▒┬á0.3	4.734eΓêÆ03┬á┬▒┬á0.4┬á┬▒┬á0.2┬á┬▒┬á0.3	4.738eΓêÆ03┬á┬▒┬á0.3┬á┬▒┬á0.1┬á┬▒┬á0.2
42.0ΓÇô45.0	4.085eΓêÆ03┬á┬▒┬á0.5┬á┬▒┬á0.3┬á┬▒┬á0.7	4.100eΓêÆ03┬á┬▒┬á0.5┬á┬▒┬á0.3┬á┬▒┬á0.7	4.066eΓêÆ03┬á┬▒┬á0.4┬á┬▒┬á0.2┬á┬▒┬á0.3	4.086eΓêÆ03┬á┬▒┬á0.4┬á┬▒┬á0.2┬á┬▒┬á0.3	4.098eΓêÆ03┬á┬▒┬á0.4┬á┬▒┬á0.2┬á┬▒┬á0.3	4.094eΓêÆ03┬á┬▒┬á0.3┬á┬▒┬á0.2┬á┬▒┬á0.3
45.0ΓÇô48.0	3.557eΓêÆ03┬á┬▒┬á0.6┬á┬▒┬á0.3┬á┬▒┬á0.7	3.570eΓêÆ03┬á┬▒┬á0.6┬á┬▒┬á0.3┬á┬▒┬á0.7	3.519eΓêÆ03┬á┬▒┬á0.5┬á┬▒┬á0.2┬á┬▒┬á0.4	3.541eΓêÆ03┬á┬▒┬á0.5┬á┬▒┬á0.2┬á┬▒┬á0.4	3.555eΓêÆ03┬á┬▒┬á0.5┬á┬▒┬á0.2┬á┬▒┬á0.4	3.558eΓêÆ03┬á┬▒┬á0.4┬á┬▒┬á0.2┬á┬▒┬á0.3
48.0ΓÇô51.0	3.060eΓêÆ03┬á┬▒┬á0.6┬á┬▒┬á0.3┬á┬▒┬á0.7	3.072eΓêÆ03┬á┬▒┬á0.6┬á┬▒┬á0.3┬á┬▒┬á0.8	3.009eΓêÆ03┬á┬▒┬á0.5┬á┬▒┬á0.2┬á┬▒┬á0.4	3.027eΓêÆ03┬á┬▒┬á0.5┬á┬▒┬á0.2┬á┬▒┬á0.4	3.041eΓêÆ03┬á┬▒┬á0.5┬á┬▒┬á0.2┬á┬▒┬á0.5	3.050eΓêÆ03┬á┬▒┬á0.4┬á┬▒┬á0.2┬á┬▒┬á0.3
51.0ΓÇô54.0	2.706eΓêÆ03┬á┬▒┬á0.6┬á┬▒┬á0.4┬á┬▒┬á0.9	2.720eΓêÆ03┬á┬▒┬á0.6┬á┬▒┬á0.4┬á┬▒┬á0.9	2.640eΓêÆ03┬á┬▒┬á0.6┬á┬▒┬á0.2┬á┬▒┬á0.5	2.660eΓêÆ03┬á┬▒┬á0.5┬á┬▒┬á0.2┬á┬▒┬á0.5	2.676eΓêÆ03┬á┬▒┬á0.5┬á┬▒┬á0.2┬á┬▒┬á0.5	2.690eΓêÆ03┬á┬▒┬á0.4┬á┬▒┬á0.2┬á┬▒┬á0.4
54.0ΓÇô57.0	2.358eΓêÆ03┬á┬▒┬á0.7┬á┬▒┬á0.4┬á┬▒┬á0.9	2.378eΓêÆ03┬á┬▒┬á0.7┬á┬▒┬á0.4┬á┬▒┬á0.9	2.302eΓêÆ03┬á┬▒┬á0.6┬á┬▒┬á0.2┬á┬▒┬á0.4	2.322eΓêÆ03┬á┬▒┬á0.6┬á┬▒┬á0.2┬á┬▒┬á0.4	2.339eΓêÆ03┬á┬▒┬á0.6┬á┬▒┬á0.2┬á┬▒┬á0.4	2.350eΓêÆ03┬á┬▒┬á0.5┬á┬▒┬á0.2┬á┬▒┬á0.3
57.0ΓÇô61.0	2.011eΓêÆ03┬á┬▒┬á0.6┬á┬▒┬á0.3┬á┬▒┬á0.8	2.025eΓêÆ03┬á┬▒┬á0.6┬á┬▒┬á0.3┬á┬▒┬á0.8	2.017eΓêÆ03┬á┬▒┬á0.5┬á┬▒┬á0.3┬á┬▒┬á0.4	2.036eΓêÆ03┬á┬▒┬á0.5┬á┬▒┬á0.3┬á┬▒┬á0.4	2.051eΓêÆ03┬á┬▒┬á0.5┬á┬▒┬á0.3┬á┬▒┬á0.4	2.038eΓêÆ03┬á┬▒┬á0.4┬á┬▒┬á0.2┬á┬▒┬á0.3
61.0ΓÇô65.0	1.702eΓêÆ03┬á┬▒┬á0.7┬á┬▒┬á0.3┬á┬▒┬á1.0	1.715eΓêÆ03┬á┬▒┬á0.7┬á┬▒┬á0.3┬á┬▒┬á1.0	1.665eΓêÆ03┬á┬▒┬á0.6┬á┬▒┬á0.2┬á┬▒┬á0.4	1.683eΓêÆ03┬á┬▒┬á0.6┬á┬▒┬á0.2┬á┬▒┬á0.4	1.698eΓêÆ03┬á┬▒┬á0.6┬á┬▒┬á0.2┬á┬▒┬á0.4	1.704eΓêÆ03┬á┬▒┬á0.5┬á┬▒┬á0.2┬á┬▒┬á0.4
65.0ΓÇô70.0	1.440eΓêÆ03┬á┬▒┬á0.6┬á┬▒┬á0.3┬á┬▒┬á1.0	1.454eΓêÆ03┬á┬▒┬á0.6┬á┬▒┬á0.3┬á┬▒┬á1.0	1.400eΓêÆ03┬á┬▒┬á0.6┬á┬▒┬á0.2┬á┬▒┬á0.4	1.417eΓêÆ03┬á┬▒┬á0.6┬á┬▒┬á0.2┬á┬▒┬á0.4	1.429eΓêÆ03┬á┬▒┬á0.6┬á┬▒┬á0.2┬á┬▒┬á0.4	1.438eΓêÆ03┬á┬▒┬á0.4┬á┬▒┬á0.2┬á┬▒┬á0.4
70.0ΓÇô75.0	1.138eΓêÆ03┬á┬▒┬á0.7┬á┬▒┬á0.3┬á┬▒┬á1.2	1.149eΓêÆ03┬á┬▒┬á0.7┬á┬▒┬á0.3┬á┬▒┬á1.2	1.131eΓêÆ03┬á┬▒┬á0.7┬á┬▒┬á0.3┬á┬▒┬á0.5	1.144eΓêÆ03┬á┬▒┬á0.7┬á┬▒┬á0.3┬á┬▒┬á0.5	1.156eΓêÆ03┬á┬▒┬á0.7┬á┬▒┬á0.3┬á┬▒┬á0.6	1.151eΓêÆ03┬á┬▒┬á0.5┬á┬▒┬á0.2┬á┬▒┬á0.5
75.0ΓÇô80.0	9.443eΓêÆ04┬á┬▒┬á0.8┬á┬▒┬á0.4┬á┬▒┬á1.1	9.550eΓêÆ04┬á┬▒┬á0.8┬á┬▒┬á0.4┬á┬▒┬á1.1	9.420eΓêÆ04┬á┬▒┬á0.7┬á┬▒┬á0.3┬á┬▒┬á0.6	9.545eΓêÆ04┬á┬▒┬á0.7┬á┬▒┬á0.3┬á┬▒┬á0.6	9.645eΓêÆ04┬á┬▒┬á0.7┬á┬▒┬á0.3┬á┬▒┬á0.6	9.582eΓêÆ04┬á┬▒┬á0.5┬á┬▒┬á0.3┬á┬▒┬á0.5
80.0ΓÇô85.0	7.761eΓêÆ04┬á┬▒┬á0.9┬á┬▒┬á0.5┬á┬▒┬á1.2	7.837eΓêÆ04┬á┬▒┬á0.9┬á┬▒┬á0.5┬á┬▒┬á1.2	7.617eΓêÆ04┬á┬▒┬á0.8┬á┬▒┬á0.4┬á┬▒┬á0.7	7.721eΓêÆ04┬á┬▒┬á0.8┬á┬▒┬á0.4┬á┬▒┬á0.7	7.808eΓêÆ04┬á┬▒┬á0.8┬á┬▒┬á0.4┬á┬▒┬á0.7	7.810eΓêÆ04┬á┬▒┬á0.6┬á┬▒┬á0.3┬á┬▒┬á0.5
85.0ΓÇô95.0	5.740eΓêÆ04┬á┬▒┬á0.6┬á┬▒┬á0.3┬á┬▒┬á0.9	5.794eΓêÆ04┬á┬▒┬á0.6┬á┬▒┬á0.3┬á┬▒┬á0.9	5.691eΓêÆ04┬á┬▒┬á0.6┬á┬▒┬á0.2┬á┬▒┬á0.5	5.771eΓêÆ04┬á┬▒┬á0.6┬á┬▒┬á0.2┬á┬▒┬á0.5	5.826eΓêÆ04┬á┬▒┬á0.6┬á┬▒┬á0.2┬á┬▒┬á0.5	5.811eΓêÆ04┬á┬▒┬á0.4┬á┬▒┬á0.2┬á┬▒┬á0.4
95.0ΓÇô105.0	4.122eΓêÆ04┬á┬▒┬á0.7┬á┬▒┬á0.3┬á┬▒┬á1.0	4.158eΓêÆ04┬á┬▒┬á0.7┬á┬▒┬á0.3┬á┬▒┬á1.0	3.992eΓêÆ04┬á┬▒┬á0.7┬á┬▒┬á0.2┬á┬▒┬á0.5	4.044eΓêÆ04┬á┬▒┬á0.7┬á┬▒┬á0.2┬á┬▒┬á0.5	4.082eΓêÆ04┬á┬▒┬á0.7┬á┬▒┬á0.2┬á┬▒┬á0.5	4.114eΓêÆ04┬á┬▒┬á0.5┬á┬▒┬á0.2┬á┬▒┬á0.4
105.0ΓÇô125.0	2.591eΓêÆ04┬á┬▒┬á0.6┬á┬▒┬á0.3┬á┬▒┬á0.9	2.612eΓêÆ04┬á┬▒┬á0.6┬á┬▒┬á0.3┬á┬▒┬á0.9	2.523eΓêÆ04┬á┬▒┬á0.6┬á┬▒┬á0.2┬á┬▒┬á0.4	2.562eΓêÆ04┬á┬▒┬á0.6┬á┬▒┬á0.2┬á┬▒┬á0.4	2.584eΓêÆ04┬á┬▒┬á0.6┬á┬▒┬á0.2┬á┬▒┬á0.4	2.593eΓêÆ04┬á┬▒┬á0.4┬á┬▒┬á0.2┬á┬▒┬á0.4
125.0ΓÇô150.0	1.337eΓêÆ04┬á┬▒┬á0.7┬á┬▒┬á0.3┬á┬▒┬á0.9	1.347eΓêÆ04┬á┬▒┬á0.7┬á┬▒┬á0.3┬á┬▒┬á0.9	1.316eΓêÆ04┬á┬▒┬á0.7┬á┬▒┬á0.2┬á┬▒┬á0.4	1.337eΓêÆ04┬á┬▒┬á0.7┬á┬▒┬á0.2┬á┬▒┬á0.4	1.347eΓêÆ04┬á┬▒┬á0.7┬á┬▒┬á0.2┬á┬▒┬á0.4	1.346eΓêÆ04┬á┬▒┬á0.5┬á┬▒┬á0.2┬á┬▒┬á0.4
150.0ΓÇô175.0	6.769eΓêÆ05┬á┬▒┬á1.0┬á┬▒┬á0.4┬á┬▒┬á1.0	6.816eΓêÆ05┬á┬▒┬á1.0┬á┬▒┬á0.4┬á┬▒┬á1.0	6.974eΓêÆ05┬á┬▒┬á1.0┬á┬▒┬á0.4┬á┬▒┬á0.5	7.115eΓêÆ05┬á┬▒┬á1.0┬á┬▒┬á0.4┬á┬▒┬á0.5	7.163eΓêÆ05┬á┬▒┬á1.0┬á┬▒┬á0.4┬á┬▒┬á0.6	6.980eΓêÆ05┬á┬▒┬á0.7┬á┬▒┬á0.3┬á┬▒┬á0.5
175.0ΓÇô200.0	3.840eΓêÆ05┬á┬▒┬á1.3┬á┬▒┬á0.5┬á┬▒┬á1.3	3.865eΓêÆ05┬á┬▒┬á1.3┬á┬▒┬á0.5┬á┬▒┬á1.3	3.668eΓêÆ05┬á┬▒┬á1.5┬á┬▒┬á0.7┬á┬▒┬á0.8	3.744eΓêÆ05┬á┬▒┬á1.5┬á┬▒┬á0.7┬á┬▒┬á0.8	3.769eΓêÆ05┬á┬▒┬á1.5┬á┬▒┬á0.7┬á┬▒┬á0.8	3.809eΓêÆ05┬á┬▒┬á1.0┬á┬▒┬á0.4┬á┬▒┬á0.6
200.0ΓÇô250.0	1.753eΓêÆ05┬á┬▒┬á1.3┬á┬▒┬á0.5┬á┬▒┬á1.5	1.766eΓêÆ05┬á┬▒┬á1.3┬á┬▒┬á0.5┬á┬▒┬á1.6	1.725eΓêÆ05┬á┬▒┬á1.4┬á┬▒┬á0.6┬á┬▒┬á0.8	1.762eΓêÆ05┬á┬▒┬á1.4┬á┬▒┬á0.6┬á┬▒┬á0.8	1.772eΓêÆ05┬á┬▒┬á1.4┬á┬▒┬á0.6┬á┬▒┬á0.8	1.769eΓêÆ05┬á┬▒┬á0.9┬á┬▒┬á0.4┬á┬▒┬á0.7
250.0ΓÇô300.0	6.578eΓêÆ06┬á┬▒┬á2.1┬á┬▒┬á0.7┬á┬▒┬á2.2	6.591eΓêÆ06┬á┬▒┬á2.1┬á┬▒┬á0.7┬á┬▒┬á2.2	6.569eΓêÆ06┬á┬▒┬á2.4┬á┬▒┬á1.1┬á┬▒┬á1.4	6.756eΓêÆ06┬á┬▒┬á2.4┬á┬▒┬á1.1┬á┬▒┬á1.4	6.793eΓêÆ06┬á┬▒┬á2.4┬á┬▒┬á1.1┬á┬▒┬á1.5	6.658eΓêÆ06┬á┬▒┬á1.6┬á┬▒┬á0.6┬á┬▒┬á1.0
300.0ΓÇô350.0	2.813eΓêÆ06┬á┬▒┬á3.3┬á┬▒┬á1.2┬á┬▒┬á2.8	2.814eΓêÆ06┬á┬▒┬á3.3┬á┬▒┬á1.2┬á┬▒┬á2.8	2.653eΓêÆ06┬á┬▒┬á4.2┬á┬▒┬á2.0┬á┬▒┬á3.1	2.735eΓêÆ06┬á┬▒┬á4.2┬á┬▒┬á2.0┬á┬▒┬á3.1	2.747eΓêÆ06┬á┬▒┬á4.2┬á┬▒┬á2.0┬á┬▒┬á3.1	2.789eΓêÆ06┬á┬▒┬á2.6┬á┬▒┬á1.1┬á┬▒┬á1.6
350.0ΓÇô400.0	1.194eΓêÆ06┬á┬▒┬á4.9┬á┬▒┬á1.7┬á┬▒┬á2.2	1.207eΓêÆ06┬á┬▒┬á4.9┬á┬▒┬á1.7┬á┬▒┬á2.3	1.172eΓêÆ06┬á┬▒┬á6.6┬á┬▒┬á3.1┬á┬▒┬á4.8	1.228eΓêÆ06┬á┬▒┬á6.6┬á┬▒┬á3.1┬á┬▒┬á4.8	1.227eΓêÆ06┬á┬▒┬á6.6┬á┬▒┬á3.1┬á┬▒┬á4.9	1.217eΓêÆ06┬á┬▒┬á4.0┬á┬▒┬á1.5┬á┬▒┬á2.0
400.0ΓÇô470.0	5.587eΓêÆ07┬á┬▒┬á6.2┬á┬▒┬á1.9┬á┬▒┬á4.6	5.537eΓêÆ07┬á┬▒┬á6.2┬á┬▒┬á1.9┬á┬▒┬á4.8	5.578eΓêÆ07┬á┬▒┬á8.0┬á┬▒┬á3.9┬á┬▒┬á4.6	5.711eΓêÆ07┬á┬▒┬á8.0┬á┬▒┬á3.9┬á┬▒┬á4.6	5.698eΓêÆ07┬á┬▒┬á8.0┬á┬▒┬á3.9┬á┬▒┬á4.6	5.565eΓêÆ07┬á┬▒┬á4.9┬á┬▒┬á1.8┬á┬▒┬á2.6
470.0ΓÇô550.0	1.882eΓêÆ07┬á┬▒┬á9.6┬á┬▒┬á2.6┬á┬▒┬á4.1	1.868eΓêÆ07┬á┬▒┬á9.6┬á┬▒┬á2.6┬á┬▒┬á4.1	2.055eΓêÆ07┬á┬▒┬á12.7┬á┬▒┬á7.5┬á┬▒┬á9.0	2.118eΓêÆ07┬á┬▒┬á12.7┬á┬▒┬á7.5┬á┬▒┬á9.0	2.113eΓêÆ07┬á┬▒┬á12.7┬á┬▒┬á7.5┬á┬▒┬á9.1	1.920eΓêÆ07┬á┬▒┬á7.7┬á┬▒┬á2.9┬á┬▒┬á3.5
550.0ΓÇô650.0	6.450eΓêÆ08┬á┬▒┬á14.1┬á┬▒┬á3.8┬á┬▒┬á6.6	6.470eΓêÆ08┬á┬▒┬á14.1┬á┬▒┬á3.8┬á┬▒┬á6.6	8.090eΓêÆ08┬á┬▒┬á17.4┬á┬▒┬á10.2┬á┬▒┬á16.6	8.540eΓêÆ08┬á┬▒┬á17.4┬á┬▒┬á10.2┬á┬▒┬á16.6	8.560eΓêÆ08┬á┬▒┬á17.4┬á┬▒┬á10.2┬á┬▒┬á16.9	7.065eΓêÆ08┬á┬▒┬á11.1┬á┬▒┬á4.1┬á┬▒┬á5.8
650.0ΓÇô900.0	1.500eΓêÆ08┬á┬▒┬á17.9┬á┬▒┬á4.8┬á┬▒┬á7.8	1.530eΓêÆ08┬á┬▒┬á17.9┬á┬▒┬á4.8┬á┬▒┬á8.3	6.800eΓêÆ09┬á┬▒┬á42.2┬á┬▒┬á20.3┬á┬▒┬á25.7	7.000eΓêÆ09┬á┬▒┬á42.2┬á┬▒┬á20.3┬á┬▒┬á25.7	6.900eΓêÆ09┬á┬▒┬á42.2┬á┬▒┬á20.3┬á┬▒┬á25.7	1.386eΓêÆ08┬á┬▒┬á16.0┬á┬▒┬á5.9┬á┬▒┬á7.3

**Table 31:** The values of $(1/\sigma )\,d\sigma /dp_\mathrm {T}^{\ell \ell }$ in each bin of $p_\mathrm {T}^{\ell \ell }$ for the electron and muon channels separately (for various generator-level definitions) and for the Born-level combination in the kinematic region $116~\text {GeV} \le m_{\ell \ell }< 150~\text {GeV},\ 0.0 \le |y_{\ell \ell }| < 2.4$. The associated statistical and systematic (both uncorrelated and correlated between bins) are provided in percentage form

Bin	$(1/\sigma )\,d\sigma /dp_\mathrm {T}^{\ell \ell }$ ┬▒ Statistical [%] ┬▒ Uncorrelated systematic [%] ┬▒ Correlated systematic [%]					
	Electron channel		Muon channel			Combination
	Dressed	Born	Bare	Dressed	Born	Born
0.0ΓÇô2.0	2.181eΓêÆ02┬á┬▒┬á3.0┬á┬▒┬á1.3┬á┬▒┬á4.4	2.228eΓêÆ02┬á┬▒┬á3.0┬á┬▒┬á1.3┬á┬▒┬á4.4	1.967eΓêÆ02┬á┬▒┬á2.7┬á┬▒┬á1.4┬á┬▒┬á5.0	2.023eΓêÆ02┬á┬▒┬á2.7┬á┬▒┬á1.4┬á┬▒┬á5.0	2.071eΓêÆ02┬á┬▒┬á2.7┬á┬▒┬á1.4┬á┬▒┬á5.1	2.130eΓêÆ02┬á┬▒┬á2.0┬á┬▒┬á1.0┬á┬▒┬á4.3
2.0ΓÇô4.0	4.492eΓêÆ02┬á┬▒┬á1.9┬á┬▒┬á0.9┬á┬▒┬á1.8	4.572eΓêÆ02┬á┬▒┬á1.9┬á┬▒┬á0.9┬á┬▒┬á1.8	4.413eΓêÆ02┬á┬▒┬á1.7┬á┬▒┬á0.8┬á┬▒┬á1.2	4.488eΓêÆ02┬á┬▒┬á1.7┬á┬▒┬á0.8┬á┬▒┬á1.2	4.597eΓêÆ02┬á┬▒┬á1.7┬á┬▒┬á0.8┬á┬▒┬á1.2	4.563eΓêÆ02┬á┬▒┬á1.2┬á┬▒┬á0.6┬á┬▒┬á1.1
4.0ΓÇô6.0	4.604eΓêÆ02┬á┬▒┬á1.9┬á┬▒┬á0.9┬á┬▒┬á1.4	4.659eΓêÆ02┬á┬▒┬á1.9┬á┬▒┬á0.9┬á┬▒┬á1.4	4.842eΓêÆ02┬á┬▒┬á1.6┬á┬▒┬á0.9┬á┬▒┬á1.1	4.882eΓêÆ02┬á┬▒┬á1.6┬á┬▒┬á0.9┬á┬▒┬á1.1	4.949eΓêÆ02┬á┬▒┬á1.6┬á┬▒┬á0.9┬á┬▒┬á1.5	4.826eΓêÆ02┬á┬▒┬á1.2┬á┬▒┬á0.6┬á┬▒┬á1.0
6.0ΓÇô8.0	4.296eΓêÆ02┬á┬▒┬á1.9┬á┬▒┬á0.9┬á┬▒┬á1.3	4.332eΓêÆ02┬á┬▒┬á1.9┬á┬▒┬á0.9┬á┬▒┬á1.3	4.296eΓêÆ02┬á┬▒┬á1.6┬á┬▒┬á0.8┬á┬▒┬á1.4	4.326eΓêÆ02┬á┬▒┬á1.6┬á┬▒┬á0.8┬á┬▒┬á1.4	4.336eΓêÆ02┬á┬▒┬á1.6┬á┬▒┬á0.8┬á┬▒┬á1.4	4.306eΓêÆ02┬á┬▒┬á1.2┬á┬▒┬á0.6┬á┬▒┬á1.0
8.0ΓÇô10.0	3.850eΓêÆ02┬á┬▒┬á2.1┬á┬▒┬á1.0┬á┬▒┬á1.4	3.849eΓêÆ02┬á┬▒┬á2.1┬á┬▒┬á1.0┬á┬▒┬á1.5	3.792eΓêÆ02┬á┬▒┬á1.9┬á┬▒┬á0.8┬á┬▒┬á1.6	3.804eΓêÆ02┬á┬▒┬á1.9┬á┬▒┬á0.8┬á┬▒┬á1.6	3.818eΓêÆ02┬á┬▒┬á1.9┬á┬▒┬á0.8┬á┬▒┬á1.6	3.838eΓêÆ02┬á┬▒┬á1.4┬á┬▒┬á0.6┬á┬▒┬á1.1
10.0ΓÇô13.0	3.035eΓêÆ02┬á┬▒┬á1.9┬á┬▒┬á0.9┬á┬▒┬á1.2	3.029eΓêÆ02┬á┬▒┬á1.9┬á┬▒┬á0.9┬á┬▒┬á1.3	3.097eΓêÆ02┬á┬▒┬á1.7┬á┬▒┬á0.8┬á┬▒┬á1.0	3.081eΓêÆ02┬á┬▒┬á1.7┬á┬▒┬á0.8┬á┬▒┬á1.0	3.067eΓêÆ02┬á┬▒┬á1.7┬á┬▒┬á0.8┬á┬▒┬á1.5	3.049eΓêÆ02┬á┬▒┬á1.3┬á┬▒┬á0.6┬á┬▒┬á1.1
13.0ΓÇô16.0	2.439eΓêÆ02┬á┬▒┬á2.2┬á┬▒┬á0.9┬á┬▒┬á1.4	2.430eΓêÆ02┬á┬▒┬á2.2┬á┬▒┬á0.9┬á┬▒┬á1.4	2.572eΓêÆ02┬á┬▒┬á1.8┬á┬▒┬á0.8┬á┬▒┬á1.1	2.552eΓêÆ02┬á┬▒┬á1.8┬á┬▒┬á0.8┬á┬▒┬á1.1	2.537eΓêÆ02┬á┬▒┬á1.8┬á┬▒┬á0.8┬á┬▒┬á1.1	2.479eΓêÆ02┬á┬▒┬á1.4┬á┬▒┬á0.6┬á┬▒┬á0.9
16.0ΓÇô20.0	1.889eΓêÆ02┬á┬▒┬á2.0┬á┬▒┬á0.9┬á┬▒┬á1.4	1.873eΓêÆ02┬á┬▒┬á2.0┬á┬▒┬á0.9┬á┬▒┬á1.4	1.974eΓêÆ02┬á┬▒┬á1.8┬á┬▒┬á0.8┬á┬▒┬á1.0	1.956eΓêÆ02┬á┬▒┬á1.8┬á┬▒┬á0.8┬á┬▒┬á1.0	1.941eΓêÆ02┬á┬▒┬á1.8┬á┬▒┬á0.8┬á┬▒┬á1.0	1.912eΓêÆ02┬á┬▒┬á1.3┬á┬▒┬á0.6┬á┬▒┬á0.9
20.0ΓÇô25.0	1.479eΓêÆ02┬á┬▒┬á1.9┬á┬▒┬á0.9┬á┬▒┬á1.3	1.468eΓêÆ02┬á┬▒┬á1.9┬á┬▒┬á0.9┬á┬▒┬á1.3	1.477eΓêÆ02┬á┬▒┬á1.7┬á┬▒┬á0.8┬á┬▒┬á1.1	1.463eΓêÆ02┬á┬▒┬á1.7┬á┬▒┬á0.8┬á┬▒┬á1.1	1.447eΓêÆ02┬á┬▒┬á1.7┬á┬▒┬á0.8┬á┬▒┬á1.1	1.453eΓêÆ02┬á┬▒┬á1.3┬á┬▒┬á0.6┬á┬▒┬á0.9
25.0ΓÇô30.0	1.076eΓêÆ02┬á┬▒┬á2.3┬á┬▒┬á1.0┬á┬▒┬á1.5	1.067eΓêÆ02┬á┬▒┬á2.3┬á┬▒┬á1.0┬á┬▒┬á1.7	1.096eΓêÆ02┬á┬▒┬á2.0┬á┬▒┬á0.9┬á┬▒┬á1.2	1.084eΓêÆ02┬á┬▒┬á2.0┬á┬▒┬á0.9┬á┬▒┬á1.2	1.074eΓêÆ02┬á┬▒┬á2.0┬á┬▒┬á0.9┬á┬▒┬á1.3	1.074eΓêÆ02┬á┬▒┬á1.5┬á┬▒┬á0.7┬á┬▒┬á1.1
30.0ΓÇô37.0	7.884eΓêÆ03┬á┬▒┬á2.3┬á┬▒┬á1.2┬á┬▒┬á1.6	7.767eΓêÆ03┬á┬▒┬á2.3┬á┬▒┬á1.2┬á┬▒┬á1.7	8.028eΓêÆ03┬á┬▒┬á1.9┬á┬▒┬á0.6┬á┬▒┬á1.1	7.948eΓêÆ03┬á┬▒┬á1.9┬á┬▒┬á0.6┬á┬▒┬á1.1	7.892eΓêÆ03┬á┬▒┬á1.9┬á┬▒┬á0.6┬á┬▒┬á1.2	7.864eΓêÆ03┬á┬▒┬á1.5┬á┬▒┬á0.6┬á┬▒┬á1.1
37.0ΓÇô45.0	5.685eΓêÆ03┬á┬▒┬á2.3┬á┬▒┬á1.2┬á┬▒┬á1.5	5.620eΓêÆ03┬á┬▒┬á2.3┬á┬▒┬á1.2┬á┬▒┬á1.5	5.688eΓêÆ03┬á┬▒┬á2.1┬á┬▒┬á0.8┬á┬▒┬á1.5	5.628eΓêÆ03┬á┬▒┬á2.1┬á┬▒┬á0.8┬á┬▒┬á1.5	5.560eΓêÆ03┬á┬▒┬á2.1┬á┬▒┬á0.8┬á┬▒┬á1.5	5.611eΓêÆ03┬á┬▒┬á1.6┬á┬▒┬á0.7┬á┬▒┬á1.2
45.0ΓÇô55.0	4.160eΓêÆ03┬á┬▒┬á2.4┬á┬▒┬á1.1┬á┬▒┬á1.8	4.130eΓêÆ03┬á┬▒┬á2.4┬á┬▒┬á1.1┬á┬▒┬á1.8	3.590eΓêÆ03┬á┬▒┬á2.5┬á┬▒┬á1.0┬á┬▒┬á2.3	3.573eΓêÆ03┬á┬▒┬á2.5┬á┬▒┬á1.0┬á┬▒┬á2.3	3.540eΓêÆ03┬á┬▒┬á2.5┬á┬▒┬á1.0┬á┬▒┬á2.3	3.842eΓêÆ03┬á┬▒┬á1.7┬á┬▒┬á0.7┬á┬▒┬á1.6
55.0ΓÇô65.0	2.592eΓêÆ03┬á┬▒┬á3.3┬á┬▒┬á1.2┬á┬▒┬á2.9	2.585eΓêÆ03┬á┬▒┬á3.3┬á┬▒┬á1.2┬á┬▒┬á2.9	2.614eΓêÆ03┬á┬▒┬á3.1┬á┬▒┬á1.2┬á┬▒┬á2.8	2.604eΓêÆ03┬á┬▒┬á3.1┬á┬▒┬á1.2┬á┬▒┬á2.8	2.576eΓêÆ03┬á┬▒┬á3.1┬á┬▒┬á1.2┬á┬▒┬á2.8	2.597eΓêÆ03┬á┬▒┬á2.2┬á┬▒┬á0.9┬á┬▒┬á2.2
65.0ΓÇô75.0	1.870eΓêÆ03┬á┬▒┬á3.9┬á┬▒┬á1.8┬á┬▒┬á3.2	1.858eΓêÆ03┬á┬▒┬á3.9┬á┬▒┬á1.8┬á┬▒┬á3.2	1.706eΓêÆ03┬á┬▒┬á4.2┬á┬▒┬á1.7┬á┬▒┬á4.0	1.691eΓêÆ03┬á┬▒┬á4.2┬á┬▒┬á1.7┬á┬▒┬á4.0	1.680eΓêÆ03┬á┬▒┬á4.2┬á┬▒┬á1.7┬á┬▒┬á4.1	1.777eΓêÆ03┬á┬▒┬á2.9┬á┬▒┬á1.2┬á┬▒┬á2.8
75.0ΓÇô85.0	1.267eΓêÆ03┬á┬▒┬á5.0┬á┬▒┬á2.4┬á┬▒┬á3.8	1.248eΓêÆ03┬á┬▒┬á5.0┬á┬▒┬á2.4┬á┬▒┬á3.9	1.322eΓêÆ03┬á┬▒┬á4.8┬á┬▒┬á2.1┬á┬▒┬á3.7	1.325eΓêÆ03┬á┬▒┬á4.8┬á┬▒┬á2.1┬á┬▒┬á3.7	1.309eΓêÆ03┬á┬▒┬á4.8┬á┬▒┬á2.1┬á┬▒┬á3.7	1.279eΓêÆ03┬á┬▒┬á3.5┬á┬▒┬á1.6┬á┬▒┬á3.2
85.0ΓÇô105.0	8.973eΓêÆ04┬á┬▒┬á3.9┬á┬▒┬á1.2┬á┬▒┬á3.1	8.907eΓêÆ04┬á┬▒┬á3.9┬á┬▒┬á1.2┬á┬▒┬á3.1	8.350eΓêÆ04┬á┬▒┬á3.9┬á┬▒┬á1.2┬á┬▒┬á3.1	8.311eΓêÆ04┬á┬▒┬á3.9┬á┬▒┬á1.2┬á┬▒┬á3.1	8.263eΓêÆ04┬á┬▒┬á3.9┬á┬▒┬á1.2┬á┬▒┬á3.2	8.626eΓêÆ04┬á┬▒┬á2.7┬á┬▒┬á0.9┬á┬▒┬á2.9
105.0ΓÇô150.0	3.827eΓêÆ04┬á┬▒┬á3.6┬á┬▒┬á0.9┬á┬▒┬á2.9	3.819eΓêÆ04┬á┬▒┬á3.6┬á┬▒┬á0.9┬á┬▒┬á2.9	3.687eΓêÆ04┬á┬▒┬á3.5┬á┬▒┬á1.1┬á┬▒┬á2.7	3.714eΓêÆ04┬á┬▒┬á3.5┬á┬▒┬á1.1┬á┬▒┬á2.7	3.686eΓêÆ04┬á┬▒┬á3.5┬á┬▒┬á1.1┬á┬▒┬á2.8	3.755eΓêÆ04┬á┬▒┬á2.5┬á┬▒┬á0.7┬á┬▒┬á2.7
150.0ΓÇô200.0	1.226eΓêÆ04┬á┬▒┬á5.4┬á┬▒┬á2.1┬á┬▒┬á3.1	1.222eΓêÆ04┬á┬▒┬á5.4┬á┬▒┬á2.1┬á┬▒┬á3.2	1.118eΓêÆ04┬á┬▒┬á5.9┬á┬▒┬á2.2┬á┬▒┬á2.8	1.139eΓêÆ04┬á┬▒┬á5.9┬á┬▒┬á2.2┬á┬▒┬á2.8	1.120eΓêÆ04┬á┬▒┬á5.9┬á┬▒┬á2.2┬á┬▒┬á2.8	1.168eΓêÆ04┬á┬▒┬á4.0┬á┬▒┬á1.5┬á┬▒┬á2.3
200.0ΓÇô900.0	4.541eΓêÆ06┬á┬▒┬á7.1┬á┬▒┬á2.0┬á┬▒┬á3.2	4.491eΓêÆ06┬á┬▒┬á7.1┬á┬▒┬á2.0┬á┬▒┬á3.2	4.602eΓêÆ06┬á┬▒┬á6.5┬á┬▒┬á1.7┬á┬▒┬á2.3	4.718eΓêÆ06┬á┬▒┬á6.5┬á┬▒┬á1.7┬á┬▒┬á2.3	4.681eΓêÆ06┬á┬▒┬á6.5┬á┬▒┬á1.7┬á┬▒┬á2.6	4.530eΓêÆ06┬á┬▒┬á4.8┬á┬▒┬á1.3┬á┬▒┬á2.2

## Footnotes

1 ATLAS uses a right-handed coordinate system with its origin at the nominal interaction point in the centre of the detector and the *z*-axis coinciding with the axis of the beam pipe. The *x*-axis points from the interaction point to the centre of the LHC ring, and the *y*-axis points upward. Cylindrical coordinates (*r*,$\phi$) are used in the transverse plane, $\phi$ being the azimuthal angle around the beam pipe. The pseudorapidity is defined in terms of the polar angle $\theta$ as $\eta =-\ln \tan (\theta /2)$. The rapidity of a system, *y*, is defined in terms of its energy, *E*, and its longitudinal momentum, $p_z$, as $y = (1/2)\ln [(E+p_z)/(E-p_z)]$. Angular separations between particles or reconstructed objects are measured in $\eta -\phi$ space using $\Delta R = \sqrt{(\Delta \eta )^2 + (\Delta \phi )^2}$.

2 We thank Dr M. Guzzi (University of Manchester, UK) for many useful discussions and for helping us to produce the predictions from ResBos to which we compare our measurements.

3 Following Ref. [49] the central value of the non-perturbative parameter $a_Z=1.1$ GeV$^2$ is chosen in ResBos. The central values of the QCD scale parameters of the CSS formalism used in ResBos are chosen to be $C_1=C_3=2b_0$ and $C_2=C_4=1/2$, where $b_0 = e^{-\gamma _\mathrm{E}}, \mathrm{where} \,\gamma _\mathrm{E} \approx 0.577$ is the EulerΓÇôMascheroni constant. In assigning uncertainties to the predictions of ResBos the value of $a_Z$ is varied over the range $1.05< a_Z < 1.19$ GeV$^2$. The QCD scale uncertainties for the ResBos predictions are evaluated by varying independently the scale parameters $C_1, C_2$ and $C_3$ up and down by a factor of two relative to the central values given above. The relationship $C_2=C_4$ is maintained throughout. The overall QCD scale uncertainty is taken as the quadrature sum of the changes in the predicted distribution resulting from the variations in $C_1, C_2$ and $C_3$. PDF uncertainties are evaluated using the CT14 NNLO PDF error sets.

4 The NLO electroweak virtual corrections are provided as fractional difference of calculations performed at the order $\mathcal{O}{(\alpha ^3\alpha _\mathrm{s}})$ compared to $\mathcal{O} (\alpha ^2\alpha _\mathrm{s})$. This fractional difference is then applied directly to the $\mathcal{O} (\alpha ^2\alpha _\mathrm{s }^2)$ QCD calculation from Dynnlo following the prescription of Ref.┬á[67]. The nominal renormalisation ($\mu _{\mathrm{R}}$) and factorisation ($\mu _{\mathrm{F}}$) scales are implemented to take dynamically the value of $\sqrt{{m_{\ell \ell }}^2 + p_T^{\ell \ell \ 2}}$. For the evaluation of scale uncertainties the scales $\mu _{\mathrm{R}}$ and $\mu _{\mathrm{F}}$ are varied simultaneously by a factor of two up and down.

5 For small values of $\phi ^*_{\eta }$ the following approximate relationship holds ${\phi ^*_{\eta }} \approx a_\mathrm{T}/{m_{\ell \ell }}$. Here $a_\mathrm{T }$┬á[68] is one of the two orthogonal components of $p_\mathrm{T} ^{\ell \ell }$, which explains the factor of $\sqrt{2}$ in scaling from $\phi ^*_{\eta }$ to $p_\mathrm{T} ^{\ell \ell }$. For events at the *Z*-boson mass peak we take ${m_{\ell \ell }}\approx m_Z$.
